# Lipopolysaccharide insertion and vesicle mediated turnover drive growth independent outer membrane adaptation in *Escherichia coli*

**DOI:** 10.1038/s41467-026-76886-6

**Published:** 2026-08-21

**Authors:** Joe Nabarro, Natasha E. Hatton, Bartosz L. Kowalski, Christopher D. Spicer, Dmitri O. Pushkin, Martin A. Fascione, Christoph G. Baumann

**Affiliations:** 1 Department of Chemistry, University of York, York, UK; 2 Department of Biology, University of York, York, UK; 3 Department of Mathematics, University of York, York, UK

**Keywords:** Bacterial structural biology, Glycobiology

## Abstract

The Gram-negative outer membrane is a load-bearing permeability barrier dependent on ordered lipopolysaccharide (LPS) packing in its outer leaflet. How this organisation is maintained after LPS insertion, and whether bacteria can remodel LPS independently of growth, remain unclear. Existing models attribute LPS turnover to passive dilution during elongation and division, limiting adaptation as growth slows. Here we show that, as *Escherichia coli* enters stationary phase and elongation slows, new LPS insertion continues while pre-existing LPS is preferentially removed through outer membrane vesicles, enabling growth-independent surface remodelling. Pulse–chase metabolic labelling and super-resolution microscopy reveal that newly inserted LPS localises to discrete sites and remains segregated from pre-existing LPS. Spatiotemporal analysis supports an insertion-trapping model in which localised insertion and restricted lateral diffusion maintain LPS-rich patches without coarsening into larger domains. Time-lapse imaging, biochemical fractionation and nanoparticle tracking identify vesicle release as a route that uncouples LPS turnover from cell growth.

## Abstract (web summary)

Lipopolysaccharide (LPS) organisation and turnover in *E. coli* is integral to outer membrane homeostasis. Here, pulse-chase labelling and imaging reveal sustained spatial segregation of pre-existing and newly inserted  LPS, together with growth-independent LPS turnover by vesiculation.

## Introduction

The asymmetric outer membrane (OM) of Gram-negative bacteria provides an innate permeability barrier that provides protection against toxic compounds, large antibiotics (typically >500 Da), and host immune defence molecules such as antimicrobial peptides and antibodies^1–4^. In addition to this protective role, the OM contributes to the structural integrity of the bacterial cell envelope, providing rigidity and mechanical stability^5,6^. These functions are dependent on OM molecular organisation, whose outer leaflet comprises a densely packed layer of lipopolysaccharide (LPS) molecules interspersed with a range of transmembrane β-barrel outer membrane proteins (OMPs). While OMPs enable selective permeability and impart key functionalities such as intercellular signalling and toxin secretion^7,8^, it is LPS, as the major constituent of the OM outer leaflet, that intrinsically underpins the barrier and load-bearing functional roles of the OM^4,9^. Extreme lateral confinement of LPS in the OM, governed by a network of cooperative intermolecular interactions^10,11^, reinforces its rigidity and creates a highly-ordered glycolipid matrix that restricts the lateral mobility of all OM components, supporting both permeability resistance and mechanical robustness^11,12^. A direct implication of this solid-like behaviour is that OM expansion during growth cannot be achieved by rapid lateral equilibration but instead must rely on spatially localised insertion of new material into an otherwise diffusion-limited outer leaflet. In line with this growth-and-displacement mechanism, using a covalently labelled LamB trimeric maltoporin derivative as a surface reporter, Ursell et al. combined time lapse fluorescence microscopy with mathematical modelling to infer that *Escherichia coli* (*E. coli*) OM expansion occurs through stochastic, spatially localised insertion of new material along the cylindrical cell surface^13^. Under restricted lateral mobility, growth driven displacement then predicts progressive enrichment of older OM material at the poles^13^.

Despite its accepted functional significance, little is known about how LPS organisation in the OM is maintained after insertion and the subsequent fate of LPS molecules after it is incorporated into the OM outer leaflet. Existing models largely assume that LPS remains in the OM indefinitely, diluted only by bacterial envelope area expansion during cell growth and partitioning during cell division^10,14^. Thus, whether LPS can be turned over independently of cell growth, or preferentially removed from the OM to enable adaptive remodelling of the primary interface of the cell with its environment, has yet to be established.

This represents a blind spot in our understanding of bacterial envelope homoeostasis and adaptation, particularly in non-dividing or slow-growing cells in stationary-phase that occupy pathologically relevant, nutrient-limited niches. These include, for example, the uroepithelium, the viscous, hypoxic mucus of cystic fibrosis airways, and membrane-bound intracellular compartments within host cells such as macrophage phagosomes/phagolysosomes^15–18^. These environments can impose strong OM stress by perturbing key stabilising interactions in the LPS-rich outer leaflet, for example, fluctuations in divalent cation availability and exposure to cationic antimicrobial peptides can weaken LPS–LPS cross-bridging^18–21^, while temperature shifts require rapid remodelling of lipid packing to preserve barrier function^22,23^. In growing cells, continuous envelope synthesis and dilution by growth/division can help buffer such perturbations; however, in growth-arrested cells, these routes are constrained, raising the question of how OM integrity is preserved or actively maintained.

Here, through combined pulse-chase metabolic labelling experiments and super-resolution microscopy (SRM) we demonstrate that *E. coli* cells can turn over their OM LPS content after entry into stationary phase, with outer membrane vesicle (OMV) biogenesis identified as a key route for preferential removal of pre-existing (background) LPS. We also re-examine the spatial distribution of LPS within the OM and present a revised hypothesis for OM organisation wherein outer leaflet architecture and organisation are governed by localised, stochastic insertion events and extremely restricted lateral diffusion, rather than thermodynamically-driven phase separation. Our results reveal that (1) newly inserted LPS is delivered to discrete sites distributed across the OM; (2) once inserted, new LPS remains spatially segregated from background LPS; (3) LPS-rich patches evolve in size and abundance without evidence of patch coalescence (domain coarsening); and (4) LPS insertion and turnover continue during stationary phase, with turnover mediated by OMV release that preferentially removes older background LPS.

## Results

### Dual metabolic and fluorescent labelling reveals discrete colocalisation of LptD and newly inserted LPS in the *E. coli* outer membrane

**Fig. 1:**
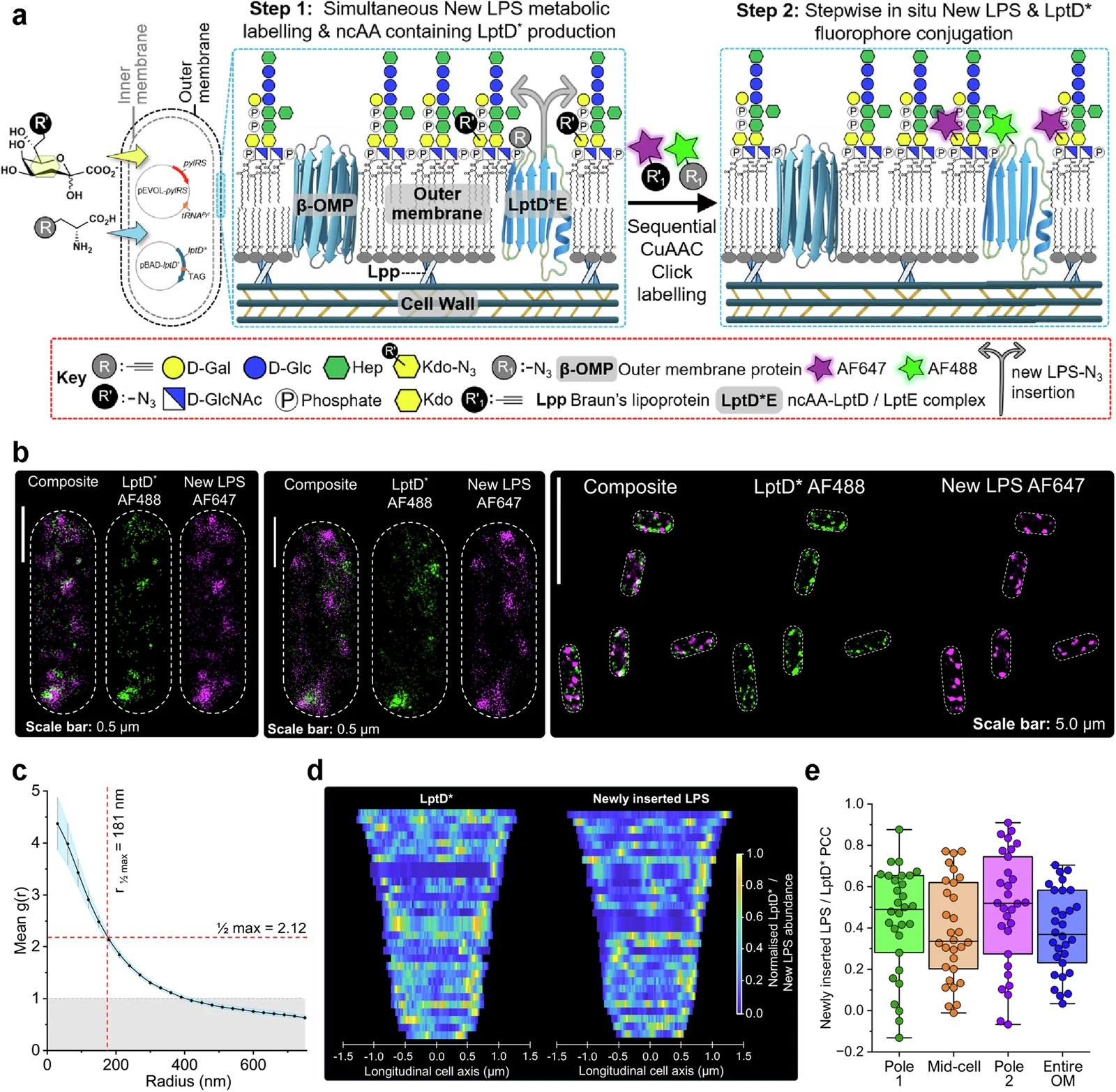
In situ bio-orthogonal dual labelling in combination with two-colour dSTORM revealed that LptD and newly inserted LPS colocalise at discrete sites across the OM in *E. coli.* **a** Schematic illustrating the dual LptD*/newly inserted LPS labelling strategy. Newly synthesised LPS was metabolically labelled via incorporation of 8-azido-3,8-dideoxy-D-manno-oct-2-ulosonic acid (Kdo-N_3_) into its inner core oligosaccharide domain, while LptD* was site-specifically labelled at position D600 with propargyl-L-lysine. Dual-colour differential fluorescent labelling was performed by sequential CuAAC reactions using appropriately functionalised dyes (see Supplementary Table 6). Glycan symbols follow the SNFG convention. **b** Representative two-colour dSTORM single-cell (left) and widefield (right) images showing co-localised punctate distributions of fluorescently labelled newly inserted LPS (AF647, magenta) and LptD* (AF488, green) across the OM of *E. coli* BW25113 cells. Newly inserted LPS appears in discrete patches that spatially coincide with LptD* puncta. Dashed outlines indicate cell boundaries. Single-cell scale bars: 0.5 µm. Widefield scale bars: 5 µm. **c** Mean ( ± S.E.M.) cross-correlation function (*g*(*r*)) calculated across 30 cells for newly inserted LPS and LptD* (*N* = 3 biological replicates, includes both dye combinations). A maximum *g*(*r*) value at short radii indicates nanoscale co-enrichment of newly inserted LPS and LptD*. **d** Demograph of LptD and newly inserted LPS distribution along the longitudinal cell axis. Heatmaps show normalised signal intensity of LptD* (left) and newly inserted LPS (right) along the longitudinal axis of individual *E. coli* cells (*n* = 30 cells). Each horizontal row corresponds to a single cell, aligned by PCA and sorted by cell length. Localisation intensity is normalised within each cell to visualise relative enrichment patterns. **e** PCC distributions in different regions (polar and mid-cell) of individual *E. coli* cells and for the entire OM surface. Consistent positive PCC values support LptD*/new LPS colocalisation (no significant statistical difference between distributions by two-tailed Mann-Whitney U-test, *p* = 0.07 – 0.97, median PCC = 0.34–0.52, see Supplementary Table 11). Each data point represents the PCC value for a single cell, with the box, horizontal line and whiskers representing upper/lower quartile, median and interquartile range of the data, respectively (*n* = 30 cells, *N *= 3 biological replicates).

To investigate the spatial dynamics of LPS insertion within the Gram-negative OM, we implemented a dual-labelling strategy in combination with two-colour SRM analysis enabling detection of newly inserted LPS and the OM LPS translocon LptD (LptDE), used here as a marker of putative LPS delivery/insertion sites^24^ (Fig. 1a and Supplementary Fig. 1). Newly synthesised LPS was metabolically tagged via the incorporation of 8-azido-3,8-dideoxy-D-manno-oct-2-ulosonic acid (Kdo-N_3_) within its inner core oligosaccharide domain, while LptD was site-specifically labelled at residue position D600 (LptD*, Fig. 1a and Supplementary Fig. 1b,c) using genetic code expansion^11,25–27^. We verified that the ncAA-labelled LptD* forms the expected LptD*/LptE complex and shows no evidence of gross assembly disruption (Supplementary Fig. 1c), supporting its use as a reporter for LptD-associated sites. As LptD is essential^28–31^, cells necessarily retain the endogenous chromosomal LptD pool; thus, direct stochastic reconstruction microscopy (dSTORM) localisations report the plasmid-expressed ncAA-containing LptD* subpopulation. To minimise perturbation of total LptD abundance and LPS transport dynamics, lptD* was expressed from a pBADcLIC vector under non-induced (leaky) conditions (without L-arabinose), since induction would drive total LptD levels far above native.

These two bio-orthogonal handles incorporated into LPS and LptD* within the cell OM were fluorescently labelled in two sequential CuAAC (click) reactions (one dye per reaction), using an alkyne-functionalised dye to label Kdo-N_3_–LPS and an azide (-N_3_)-functionalised dye to label propargyl-L-lysine-containing LptD*. Because CuAAC was performed after growth is arrested (cells on ice; pre-chilled tubes and buffers), sequential labelling does not permit appreciable redistribution of LptD/LPS between labelling steps. Furthermore, the dye labelling order was swapped in replicate experiments to exclude order-dependent artefacts (Supplementary Table 6). By coupling this dual-labelling approach with two-colour direct stochastic optical reconstruction microscopy (dSTORM), we were able to visualise newly inserted LPS and LptD distributions across the OM surface of individual cells with nanometre-scale resolution (Fig. 1a,b and Supplementary Fig. 1a)*.* Two-colour dSTORM revealed that both LptD and newly inserted LPS localised to discrete puncta scattered across the OM surface, rather than forming diffuse or uniform distributions (Fig. 1b and Supplementary Fig. 1a). These patches were consistently observed on cells irrespective of the dye combinations employed (Supplementary Fig. 1a,d), supporting the robustness of the dual-labelling and imaging strategy.

These LPS-rich patches of varying intensity colocalised with LptD* signals, suggesting that LPS insertion may be spatially restricted, and involve LptD-incorporating sites with different associated LPS insertion rates (Fig. 1b,d), in line with previous studies visualising new LPS insertion in the OM^10,12^. This co-patterning is in line with LptDE-mediated delivery but does not by itself establish that LptD activity is the sole determinant of the observed insertion pattern. The distribution of LptD*–LPS colocalisation sites across the OM indicates that LPS insertion occurs at discrete locations over the entire OM (Fig. 1d,e), rather than being concentrated within specific regions of the OM, such as the division septum, as is the case for β-barrel assembly machinery (BAM)-mediated OMP insertion^32^. Given that LptDE is the established OM translocon for LPS insertion, we interpret LptD* puncta as markers of discrete delivery sites.

To quantify colocalisation between newly inserted LPS and LptD*, we performed pairwise cross-correlation analysis across 30 cells from 3 biological replicates. The resulting mean cross-correlation function, *g*(*r*), showed a clear peak at short radii, confirming sub-diffraction co-enrichment of the two signals (Fig. 1c and Supplementary Fig. 1d,e). The *r*_1/2_ max value, representing the radius at which *g*(*r*) drops to half of its peak height, was 181 nm, indicating the typical size of co-enriched regions, which is in line with previous LPS patch and confinement diameter characterisations^10,12,33^. The half-max line value (*g*(*r*) = 2.12) further supports a strong local enrichment of new LPS near LptD*, significantly above the random expectation of 1.

To investigate whether LPS insertion follows any stereotyped spatial pattern equivalent to BAM-mediated OMP insertion in the OM^32^, we generated demographs of LptD* and newly inserted LPS along the longitudinal axis of individual cells (*n* = 30), aligning and sorting cells by length following principal component analysis (PCA)-based axis normalisation (Fig. 1d). While both signals were spatially confined, they exhibited heterogeneous distributions between cells with no consistent macrodomain localisation or polar enrichment. Instead, puncta appeared distributed yet spatially constrained, suggesting that LPS insertion occurs at discrete but stochastically positioned sites. However, not all LptD*-dense regions were accompanied by proportionally dense newly inserted LPS signal suggesting that not all LptD*/E complexes are simultaneously engaged in LPS insertion at once, aligned with the transient nature of some Lpt-mediated LPS transport bridges that form between the sites of LPS production at the inner membrane and its insertion into the OM (Fig. 1b,d)^10,32,34–36^.

We further quantified LptD*–LPS spatial relationships using their associated Pearson correlation coefficient (PCC) across the full OM, and within discrete polar and mid-cell regions of individual cells (Fig. 1e). Box plots revealed consistently positive PCC values across all regions, with no statistically significant differences between the regional PCC values (two-tailed Mann–Whitney U test, *p* > 0.05, Supplementary Table 11), indicating that the mechanism of LPS insertion and its spatial relationship with LptD is maintained across the OM surface (Fig. 1e), in agreement with the results of previous studies^10,12^. These analyses constrain the spatial scale of LptD*–new LPS co-enriched insertion regions (*r*_1/2_ max = 181 nm) and reveal heterogeneous site activity across the OM, supporting dynamic engagement of Lpt complexes. In keeping with this interpretation, recent single-molecule tracking of Lpt transport components supports highly dynamic bridge states that persist for a few seconds, providing a framework for future live-cell measurements of insertion-site dwell times and patch growth kinetics^36^.

### Spatial segregation of newly inserted and background LPS persists over time

**Fig. 2:**
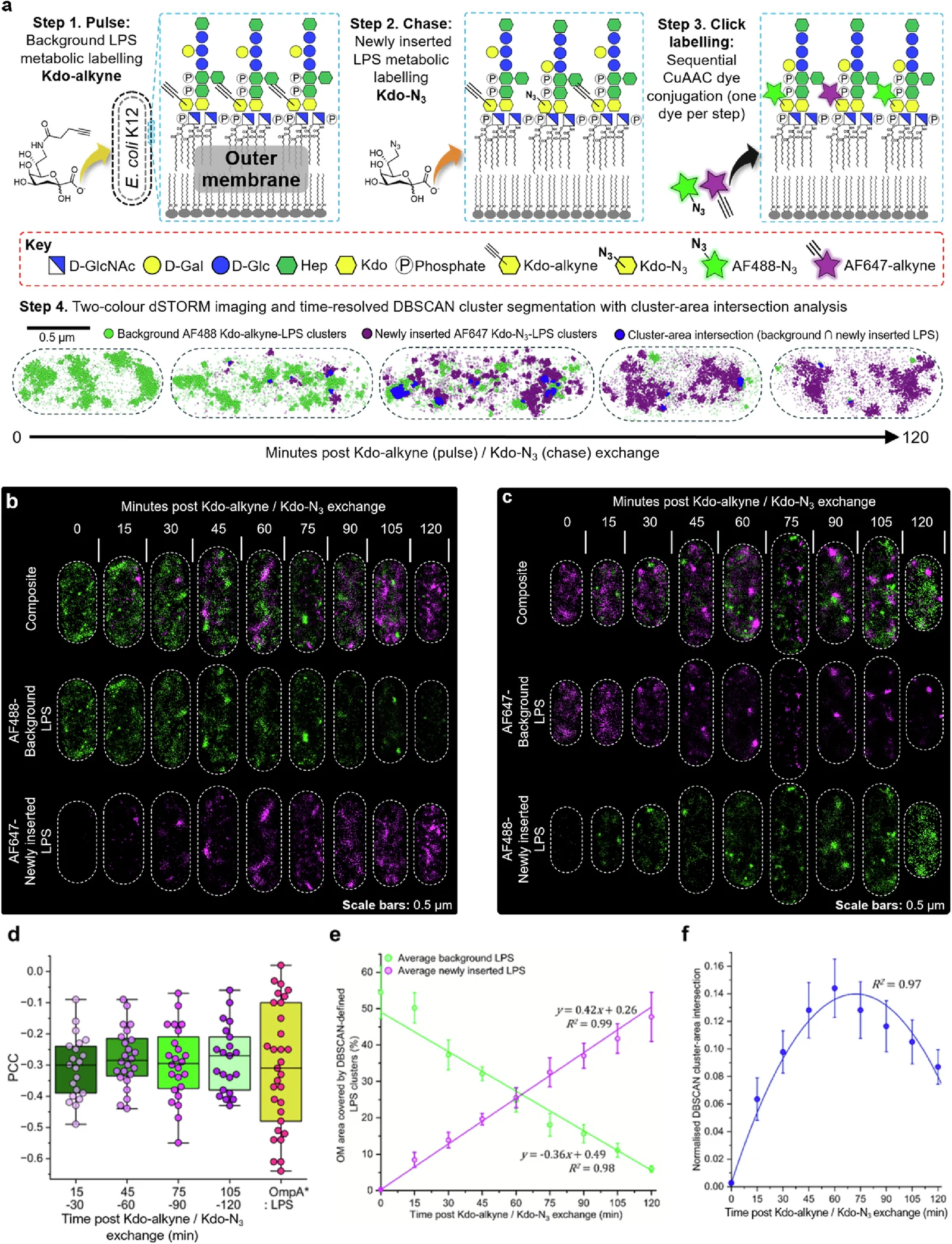
Pulse-chase metabolic labelling and two-colour dSTORM showed that newly inserted and pre-existing LPS form persistent, spatially discrete domains in OM of *E. coli* BW25113 cells. **a** Schematic overview of the experimental strategy. Kdo-alkyne probe (Step 1) was incorporated into the inner core of pre-existing (background/old) LPS. After removal of residual Kdo-alkyne, cells presenting background Kdo-alkyne-containing LPS at their surfaces are chased with Kdo-N_3_ probe (Step 2). Cells are sampled at 15 min intervals post Kdo-analogue exchange after which (Step 3) background/old Kdo-alkyne-LPS and newly inserted Kdo-N_3_-LPS are fluorescently labelled by sequential CuAAC reactions using appropriately functionalised dyes (see Supplementary Table 12). Two-colour dSTORM (Step 4) was used to visualise background and newly inserted LPS distributions at the single-cell level. Subsequent DBSCAN-based segmentation quantifies OM coverage by each LPS population. Blue indicates the intersection region between the background and newly inserted LPS cluster masks. Glycan symbols follow the SNFG convention. Scale bars: 0.5 µm. **b**, **c** Representative two-colour dSTORM images from Kdo-alkyne/Kdo-N_3_ pulse–chase experiments visualising the evolution of background LPS (middle rows) and newly inserted LPS (bottom rows) regions in OM at 15 min intervals post Kdo-analogue exchange. In (**b**), the dye identities were swapped for the sequential labelling reactions (see Supplementary Fig. 2d). Dashed outlines indicate cell boundaries. Scale bars: 0.5 µm. **d** Box plots showing the PCC values for background/old LPS and newly inserted LPS as a function of time. For comparison, PCC values were calculated for OmpA* protein and LPS-rich patches (yellow box), which are known to have anti-correlated distributions in OM. Each data point represents the PCC value for a single cell, with the box, horizontal line and whiskers representing upper/lower quartile, median and interquartile range of the data, respectively (*n* ≥ 21 cells, *N* = 3 biological replicates). **e** Percentage of OM area occupied by DBSCAN-defined background/old LPS-rich (green) and newly inserted LPS-rich (magenta) clusters over 120 min time course. Values represent mean ± S.E.M. from individual cells (*n* ≥ 10 cells per timepoint). **f** Normalised DBSCAN (with *ε* = 30 nm) cluster-mask intersection between background/old LPS and newly inserted LPS. Values represent mean ± S.E.M. from individual cells (*n *≥ 10 cells per timepoint).

To further investigate the spatiotemporal dynamics of LPS insertion and specific LPS subpopulations within the OM of *E. coli*, we developed a pulse-chase metabolic labelling strategy utilising differentially functionalised 3-deoxy-D-manno-oct-2-ulosonic acid (Kdo) sugar analogues, 8-azido-3,8-dideoxy-D-manno-oct-2-ulosonic acid (Kdo-N_3_) and 8-(4-pentynoylamido)-3-deoxy-D-manno-oct-2-ulosonic acid (Kdo-alkyne) that we combined with two-colour dSTORM^37^. A schematic overview of this approach is presented in Fig. 2a. Preliminary control experiments were performed to verify that both Kdo-analogues are incorporated in the inner core oligosaccharide domain of *E. coli* K12 LPS and enable equivalent levels of in situ fluorescent labelling (Supplementary Fig. 2a,c). To probe spatiotemporal LPS dynamics, cells were pulse-labelled with a Kdo-alkyne probe to generate what we designated as the pre-existing or background LPS population (Fig. 2a)^38^. After thorough washing to remove residual Kdo-alkyne, the cells were chased with Kdo-N_3_ to specifically label newly synthesised LPS molecules. Differential fluorescent labelling was performed by two sequential CuAAC reactions (one dye type per reaction, with washes between reactions) using distinct azide (-N_3_) and alkyne functionalised fluorescent dyes, enabling simultaneous two-colour visualisation of both LPS subpopulations in separate fluorescence channels. Control reactions in wild-type *E. coli* BW25113 cells cultured with native Kdo (no bio-orthogonal handle) and processed with the corresponding fluorescent azide(-N_3_)/alkyne reagents under identical CuAAC conditions showed minimal non-specific cell-surface staining when imaged via 3D-SIM^2^ (Supplementary Fig. 15). Two-colour 2D dSTORM imaging was performed on aliquots of the growing cells isolated at 15-min intervals over a 2-h period, which had been dual-labelled with the functionalized dyes to facilitate precise spatiotemporal tracking of the LPS subpopulations at the single-cell level.

Representative dSTORM images (Fig. 2b,c) illustrate the spatiotemporal evolution of the two LPS subpopulations. In agreement with results presented in Fig. 1, newly inserted LPS appeared predominantly as discrete, punctate clusters across the OM and had minimal spatial co-occurrence overlap with pre-existing background LPS patches (Fig. 2b,c). Identical patterning was observed irrespective of dye selection (Fig. 2b,c and Supplementary Fig. 2d). The discrete spatial arrangement of background and newly inserted LPS subpopulations in the OM of *E. coli* cells persisted throughout the entire experimental time course, indicating limited cluster-scale co-occurrence of newly inserted- and labelled background/old LPS-rich domains even as total membrane coverage by newly inserted LPS increased.

PCC values calculated from dSTORM data outputs were consistently negative (Fig. 2d), confirming sustained spatial segregation between background and newly inserted LPS. These anti-correlation values were comparable to those we calculated for labelled OmpA*-rich domains and LPS-dense patches in a previous study^11^, supporting a diffusion-limited organisation rather than an equilibrium-like compositional ordering regime in the *E. coli* OM over these timescales. Instead, our data support a distinct organisational model based on a stochastic distribution of LPS insertion sites (LptDE complexes in the OM) with variable insertion rates, combined with restricted lateral diffusion within the OM.

To quantify the spatial relationships between labelled background and newly inserted LPS-rich clusters, we applied a density-based clustering nonparametric algorithm (DBSCAN) to our dSTORM-derived datasets^39^ (Supplementary Fig. 2e). Quantitative analysis of OM coverage by DBSCAN-defined LPS clusters further reinforced our findings (Fig. 2e and Supplementary Fig. 2d). Background LPS cluster coverage gradually decreased over the 120 min time course, concomitant with a reciprocal increase in coverage by newly inserted LPS clusters (Fig. 2e and Supplementary Fig. 2d). To complement this coverage analysis with a cluster co-occurrence metric, we quantified the normalised DBSCAN cluster-area intersection (cluster-mask intersection fraction; geometric intersection of the two independently segmented cluster masks) for labelled background and newly inserted LPS (Fig. 2f and Supplementary Fig. 2e), calculated as the intersection area divided by the sum of the background- and newly inserted-cluster areas. Because this metric is defined at the cluster-mask/analysis scale (set by ε and localisation precision), it reports cluster-mask co-occurrence or proximity rather than molecular co-occupancy. This cluster-area intersection metric remained low throughout the time course, showing only a modest transient maximum around 60 min before decreasing again, indicating persistent spatial segregation and limited cluster-mask co-occurrence (Fig. 2f). Collectively, these results demonstrate that spatial segregation of newly inserted and background LPS domains persist over time. Therefore, once inserted into the OM, LPS molecules are effectively constrained with minimal lateral diffusion, matching prior findings that LPS mobility in the OM is tightly confined^10–12^, which we define as an insertion-trapping mechanism.

### Kdo-N_3_ pulse-chase experiments reveal continuous insertion of new LPS and removal of background LPS in a growth-independent manner

**Fig. 3:**
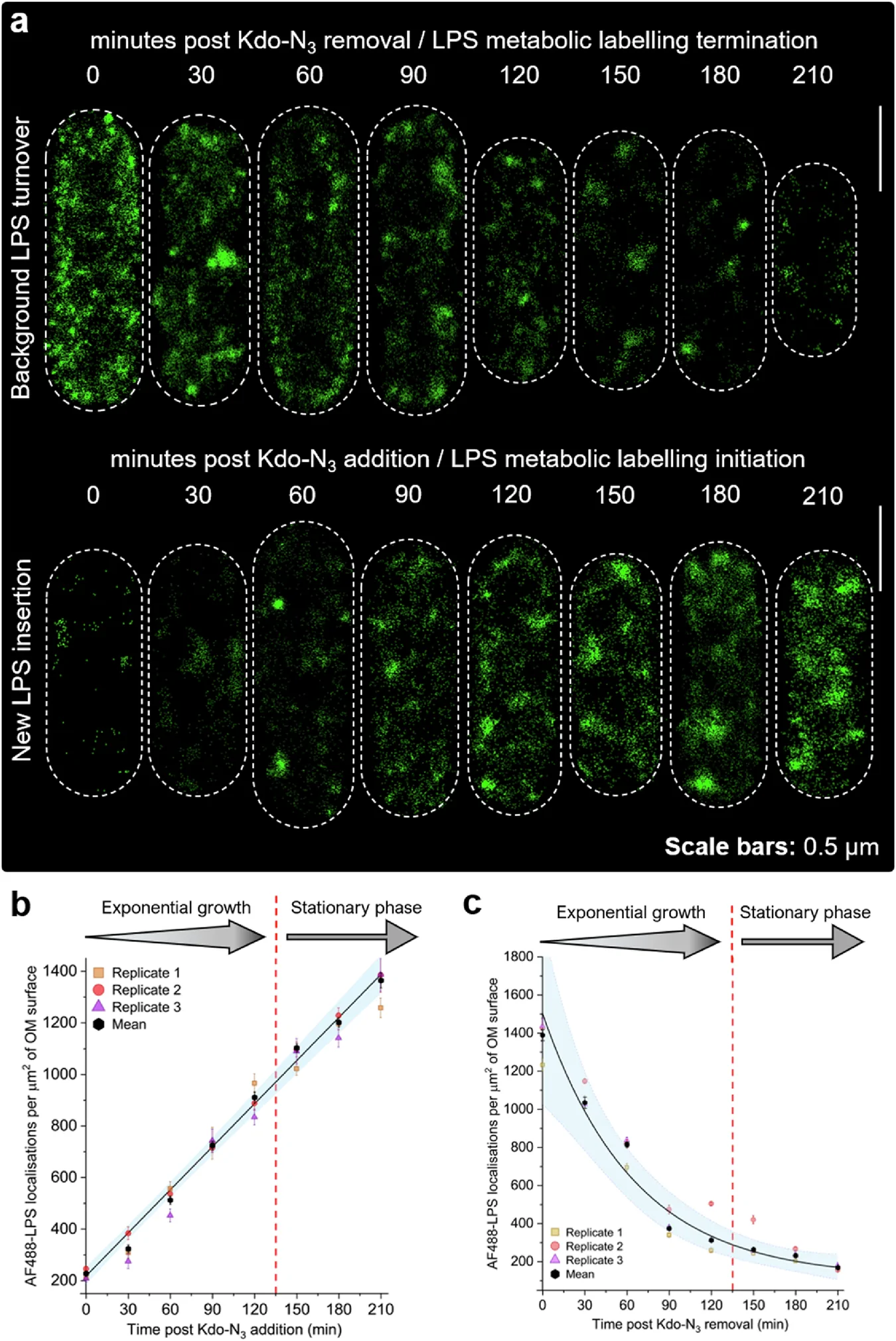
Pulse-chase metabolic labelling and dSTORM revealed continuous insertion of new LPS and removal of background LPS in a growth-independent manner. **a** Representative dSTORM images of AF488-labelled LPS in OM of single cells following termination (background LPS turnover; top row) and initiation (new LPS insertion; bottom row) of metabolic labelling with Kdo-N_3_. Newly inserted LPS appears as bright foci at discrete sites distributed across the OM, whereas pre-labelled background LPS remains in patchy domains that progressively diminish in intensity over time. Dashed outlines indicate cell boundaries. Scale bars: 0.5 μm. **b** Quantification of newly inserted LPS metabolically labelled with Kdo-N_3_ which is plotted as AF488-LPS localisation density per μm² of OM surface area for individual biological replicates (*N* = 3, coloured symbols) and the replicate mean (black hexagon) (*n* ≥ 30 cells per time point). The black line indicates the mean trendline (*R*^2^ = 0.99) with associated 95% confidence band (shaded), and the red dashed line marks the transition from exponential phase to stationary phase of growth (~135 min post Kdo-N_3_ addition). The transition point was derived from the growth curve where the OD_600_ begins to decrease towards an asymptote (see Supplementary Fig. 3c, d and Supplementary Tables 22, 23). **c** Quantification of background LPS loss following Kdo-N_3_ removal (chased with native Kdo), plotted as AF488-LPS localisation density per μm² of OM surface area for individual biological replicates (*N* = 3, coloured symbols) and the replicate mean (black hexagon) (*n* ≥ 30 cells per time point). The black line indicates the mean trendline (*R*^2^ = 0.94) with 95% confidence band (shaded), and the red dashed line marks the transition from exponential phase to stationary phase of growth (~135 min post Kdo-N_3_ removal) as described for (**b**).

**Fig. 4:**
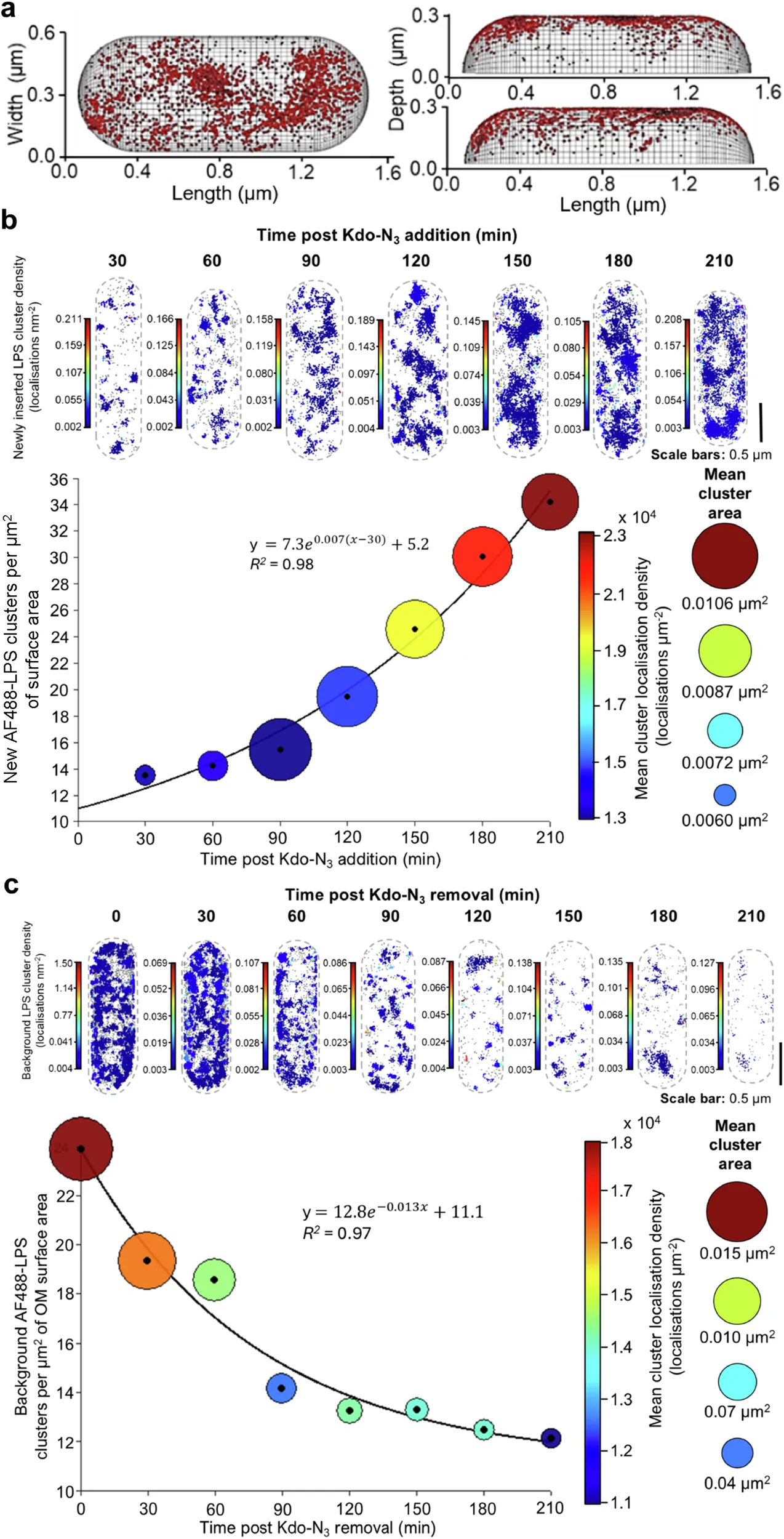
Cluster analysis of single-colour dSTORM data revealed that LPS-rich domains remain spatially discrete and do not coarsen over time in OM of *E. coli* cells. **a** Single-molecule localisations from dSTORM images were projected onto a half-rod cylindrical model of an *E. coli* cell to account for 3D curvature of OM surface and enable more accurate membrane-wide analysis of clustering. **b** Representative cluster maps (top row, scale bar: 0.5 µm) and quantification (bottom row) show that newly inserted Kdo-N_3_-containing, AF488-labelled LPS accumulates in discrete patches whose number, density and total area increase over time. Dashed outlines indicate cell boundaries. Each point on the graph represents the mean cluster value per cell (*n* ≥ 10 cells per time point, *N* = 3 biological replicates), while marker size and colour encode the mean cluster surface area and localisation density, respectively. The increase in cluster number was well described by an exponential growth-to-asymptote fit (*R*² = 0.98), consistent with continued LPS insertion after entry into stationary phase, without signatures of time-dependent domain coarsening. **c** Representative cluster maps (top row, scale bar: 0.5 µm) and quantification (bottom row) show that background Kdo-N_3_-containing, AF488-labelled LPS clusters steadily decrease in abundance, size and density over time. Data presented in the same way as (**b**) (*n* ≥ 10 cells per time point, *N* = 3 biological replicates). The decrease in cluster number followed an exponential decay-to-asymptote (*R*² = 0.97), consistent with dilution of the pre-labelled LPS pool during exponential phase followed by continued loss during stationary phase with no evidence of time-dependent domain coarsening.

Having shown that newly inserted LPS exists in discrete, spatially segregated patches located across the entire OM (Figs. 1 and 2), we adapted our pulse-chase methodology to examine the kinetics of LPS incorporation and turnover during exponential phase growth of the *E. coli* population and its transition to stationary phase. A related click-chemistry strategy was recently reported to estimate relative LPS synthesis rates in *E. coli* K-12 MG1655 by pulsing cells with Kdo-N_3_ followed by copper-free click labelling and in-gel fluorescence quantification^14^. In that study, fluorescence microscopy outputs suggested that newly synthesised LPS initially appears as discrete surface foci at short pulse times, becoming more evenly distributed with longer pulses^14^. Here, we build on this strategy by coupling a pulse-chase design to single-cell dSTORM (Fig. 3 and Supplementary Fig. 3a,b) and complementary population-scale biochemical readouts (Supplementary Fig. 6a,c) thereby enabling quantitative analysis of nanoscale new LPS insertion patterns and loss of preexisting (background/old) LPS across the exponential-to-stationary phase of growth transition (Fig. 4 and Supplementary Fig. 4).

We performed complementary pulse-chase experiments using Kdo-N_3_ and native Kdo combined with dSTORM imaging. One experiment quantified accumulation of newly inserted LPS in the OM over time by analysing individual cells sampled from the population at each 30 min time point, whilst the other quantified loss of pre-existing, background LPS from the OM of individual *E. coli* cells. Cultures were inoculated at an OD_600_ of 0.05 under standard shaking and aeration conditions; newly inserted LPS metabolic labelling was initiated by addition of Kdo-N_3_ when cultures reached OD_600_ = 0.5.

To track the accumulation of newly inserted LPS over time, cells were sampled at 30 min intervals over a 210 min time course following Kdo-N_3_ addition/LPS metabolic labelling initiation. After sampling and removal of excess Kdo-N_3_ newly inserted, metabolically labelled LPS (via Kdo-N_3_ incorporation within its inner core oligosaccharide domain) was fluorescently labelled via CuAAC in these samples and characterised in individual cells via dSTORM. For these time-course measurements, we used identical imaging and processing settings across a fixed acquisition window (5000 frames) at every time point, so that localisation density per OM area could be compared consistently as a relative proxy for Kdo-N_3_-containing, new LPS delivered to the OM. We note that a fixed 5000-frame acquisition may underrepresent absolute localisation yield, but this does not affect the comparability of localisation densities across the time course because every time point was acquired and processed under identical conditions. This fixed-window approach avoids time-dependent photophysical depletion and photobleaching effects that can compress dynamic range and bias comparisons across time points when acquisition length is varied or extended. Longer acquisitions were used elsewhere (Figs. 1 and 2) to maximise localisation yield for spatial patterning and colocalisation analyses, rather than for quantitative cross-condition density comparisons. Throughout, dSTORM localisation density and in-gel fluorescence are used as relative proxies for the abundance of fluorescently labelled LPS; accordingly, we describe change as an increase (new LPS) or decline (background/old) in metabolically- and fluorescently labelled LPS signal (newly inserted- or background LPS pool), rather than an absolute change in newly inserted/background LPS amount in the OM of cells.

Single-cell level dSTORM imaging over a 210 min chase period (Fig. 3a) again revealed ongoing incorporation of newly synthesised Kdo-N_3_ LPS at discrete punctate sites distributed across the OM consistent with new, metabolically labelled LPS distributions characterised in Figs. 1 and 2 (Supplementary Fig. 1a,d,e). Despite the culture having transitioned into stationary phase (Fig. 3b) these insertion events continued, and insertion foci remained spatially discrete rather than becoming more diffuse over time. Subsequent quantification of the number of discrete fluorescent LPS localisations (Supplementary Table 17) revealed a continuous increase in newly inserted, Kdo-N_3_-LPS throughout the experiment (Fig. 3b and Supplementary Fig. 3b). The rate of new, metabolically labelled LPS accumulation per cell did not slow, even between 135 and 210 min post Kdo-N_3_ addition (Fig. 3b and Supplementary Fig. 3b), when rates of cell growth and division began to slow markedly as the culture entered stationary phase (Fig. 3b and Supplementary Fig. 3b,d). This indicates that LPS insertion rates in the OM are maintained beyond the point of cell elongation, suggesting that LPS insertion in stationary phase *E. coli* cells becomes decoupled from rates of cell growth and division.

In a second set of experiments, we monitored the depletion of the Kdo-N_3_-containing, background (old) LPS pool from the OM of individual cells. Cells were pre-labelled with Kdo-N_3_ to metabolically label the preexisting, background LPS pool. After removal of excess Kdo-N_3_ cells were transferred to fresh medium containing native Kdo in place of Kdo-N_3_. The decline of the fluorescent signal from background LPS was similarly quantified by single-cell SRM imaging (Fig. 3b, Supplementary Fig. 3a and Supplementary Table 16), and also by bulk biochemical analysis of extracted LPS (using in-gel fluorescence as a proxy for the Kdo-N_3_-containing background/old pool; Supplementary Fig. 6a,c). At the single-cell level, dSTORM imaging revealed a progressive decline in fluorescently labelled LPS density across the entire OM (Fig. 3b, Supplementary Figs. 3a and 4a-c,g). At the bulk-population level fluorescence analysis of Tricine SDS-PAGE (TSDS-PAGE) LPS gels showed decline of the (fluorescent) background/old Kdo-N_3_ LPS signal over time (Supplementary Fig. 6a,c).

In both single-cell dSTORM and bulk biochemical assays fluorescent background/old LPS signal reduction persisted into stationary phase, despite cell elongation and division slowing significantly (Fig. 3b, Supplementary Figs. 3a, 6a and 16c). These results show that the decrease in Kdo-N_3_ background/old LPS fluorescent signal over time cannot be explained by the dilution alone that results from multiplying cells as previously postulated^10^ (Supplementary Fig. 16c). Notably, both single cell dSTORM and bulk biochemically derived rates of labelled background LPS fluorescent signal decline are faster than expected from dilution alone, consistent with growth-independent depletion or turnover of the background LPS pool (Supplementary Fig. 16c).

### LPS-rich clusters remain spatially discrete and heterogeneously distributed, inconsistent with phase ordering in the outer membrane

To investigate the observed nanoscopic clustered organisation in the OM of *E. coli* cells, we analysed the spatiotemporal behaviour of background/old and newly inserted LPS by projecting single-molecule localisations from the dSTORM pulse-chase experiments onto a half-rod cylindrical model of each cell (Fig. 4a). This enabled identification of LPS clusters using a DBSCAN algorithm, with improved accuracy by accounting for projection distortion caused by cell-surface curvature^40^. This surface-based projection enabled the evolution of labelled newly inserted and background LPS clusters to be compared across cells over time (Fig. 4b,c).

It has been proposed that organisation of the Gram-negative OM can involve phase separation or phase ordering, in which compositionally distinct regions (e.g., LPS-rich versus OMP-rich) emerge from thermodynamically favoured interactions under reduced membrane fluidity^33,41^. Here, we use phase separation and phase ordering in this compositional sense (rather than simply to denote restricted lateral diffusion). Because both LPS and OMPs exhibit very limited lateral mobility in vivo, any compositional ordering may be diffusion-limited and therefore slow to relax on experimental timescales^10,11,32^. Nevertheless, an equilibrium-like ordering regime makes the clear, testable prediction that once compositionally distinct regions form, classical models of phase-ordering kinetics predict coarsening, in which smaller regions disappear while larger regions grow through coalescence and Ostwald ripening, yielding fewer, larger regions at later time points^42–48^. Ursell et al. provided an early framework for interpreting growth-dependent patterning in the OM based on microscopy of an OMP reporter and modelling of this membrane as an incompressible 2D fluid^13^. More recent live-cell AFM and high-resolution imaging studies have described the *E. coli* OM as a spatially heterogeneous mixture of OMP-rich and LPS-rich regions and discussed this organisation in terms of phase separation or phase ordering^10,13,33,49^. We therefore tested for time-dependent coarsening signatures in both newly inserted and background LPS cluster populations (Fig. 4b,c and Supplementary Fig. 10). In our analysis of newly inserted LPS (Fig. 4b and Supplementary Fig. 10d,f), the number of clusters per cell increased steadily over the 210 min experiment, while the total area covered by LPS also expanded, yet the mean cluster size plateaued after approximately 90 min (Supplementary Fig. 10d,e).

Together with the persistent segregation of labelled new and background LPS (Fig. 2), these trends indicate stable, spatially discrete LPS-rich patches that persist without coarsening, inconsistent with equilibrium phase-ordering expectations^42–48^. Analysis of background LPS clusters supported this conclusion, with cluster number, mean cluster size, and density decreasing over the same period (Fig. 4c and Supplementary Fig. 10a-c). The 60 and 90 min time points lie on the fitted trajectory within the 95% confidence intervals, with no evidence of an abrupt step change in the observables (Fig. 4c and Supplementary Fig. 10a-c). Rather than fusing into larger domains, these pre-existing LPS-rich patches gradually dissipated, reflecting dilution during OM expansion in growing cells. We observed no evidence of coarsening (in which smaller domains vanish as larger domains grow), as expected for Ostwald ripening or domain fusion^45–47^. For example, Benn et al. reported fusion and coalescence of LPS-rich patches under low-fluidity conditions compatible with an equilibrium-like phase-ordering regime^33,41^. In contrast, across our conditions and time window, both localisation-density-defined background and newly inserted LPS pools lacked any coarsening signature, indicating that LPS lateral mixing is strongly constrained and domain coalescence is suppressed. This behaviour is compatible with a solid-like, diffusion-limited regime in which LPS-rich patches are stabilised rather than undergoing fusion-driven growth^10–13^, arguing against equilibrium phase ordering as the dominant mechanism in our system.

To test whether OM expansion during cell elongation-driven redistribution produces a polar-enriched pattern of older LPS, we categorised clusters in the OM of individual cells into pole 1, mid-cell, and pole 2 regions based on their position along the longitudinal cell axis (Supplementary Fig. 4). Using global area scaling for visualisation, per-cell plots were generated to examine clustering patterns over time for both background and newly inserted LPS (Supplementary Fig. 4g,h). Quantitative analysis showed no significant differences in cluster number, density, or size between polar and mid-cell regions at any time point, and these spatial patterns remained stable throughout both background LPS turnover and new LPS insertion time courses, without evidence of domain coalescence or regional convergence (Supplementary Fig. 4a-f).

We did not observe progressive enrichment of the pre-labelled background/old LPS pool at the poles over time, arguing against the specific binary-partition/polar-sequestration scenario in which new OM material is inserted preferentially away from the poles and pre-existing material is displaced poleward during elongation. However, we note that an absence of old-pool polar enrichment could also, in principle, arise if new LPS were inserted at the poles, locally diluting or replacing the labelled pool. Our two-colour pulse-chase data do not support preferential polar insertion of new LPS; instead, newly inserted LPS appears in discrete patches distributed along the longitudinal axis. This contrasts with growth driven redistribution frameworks derived from OMP reporters, in which spatially localised insertion together with restricted lateral mobility leads to growth-driven redistribution and progressive accumulation of older OMPs at the poles^13,32^. Taken together with restricted lateral mixing of LPS on these timescales^10–12^, this indicates that LPS organisation is not well described by a polar-sequestration/binary-partition model during growth under our conditions.

These results (Fig. 4 and Supplementary Fig. 4) argue against *E. coli* OM organisation being governed primarily by equilibrium phase ordering with domain coarsening. Instead, our data support a solid-like, diffusion-limited OM with persistent, insertion-patterned spatial heterogeneity. The Kdo-N_3_-labelled background LPS pool (as measured by fluorescence/localisation density) progressively decreases without large-scale mixing or coarsening of LPS-rich regions (Fig. 4c), while newly inserted LPS accumulates in discrete, stable patches (Fig. 4b). This behaviour is consistent with insertion trapping, in which newly inserted LPS becomes constrained upon incorporation rather than laterally redistributing to equilibrate the membrane. This restricted-mixing regime is directly observed for LPS^10–13,33^. While growth-driven redistribution frameworks inferred from OMP reporters similarly invoke restricted mobility, their predicted progressive polar enrichment additionally relies on spatially biased insertion^13,32^, which we do not detect for LPS under our conditions.

### Live-cell 3D-SIM² imaging reveals that background LPS turnover does not undergo binary partitioning or internal cytoplasmic recycling

**Fig. 5:**
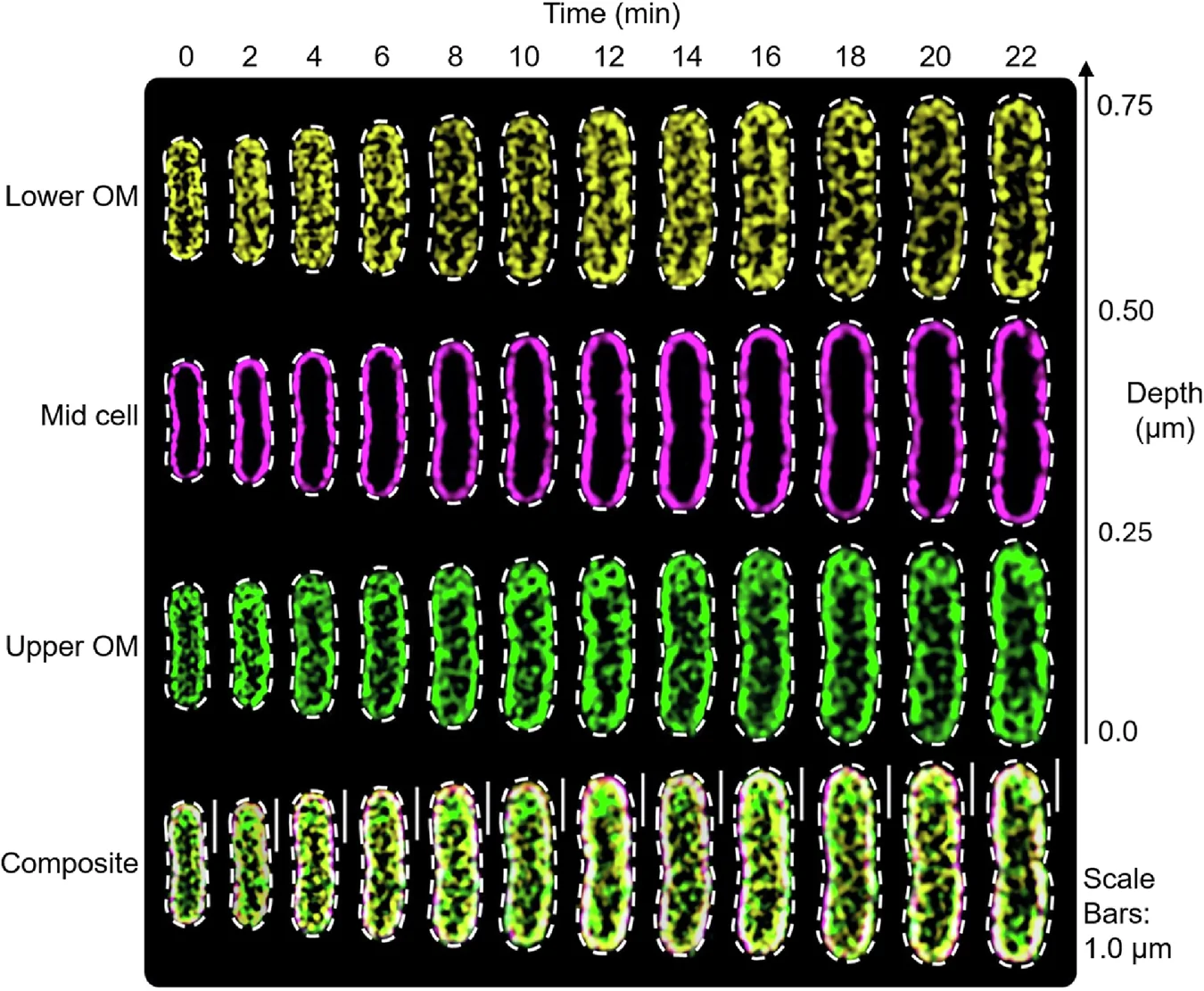
Live-cell 3D-SIM² imaging showed that background LPS was not partitioned to cell poles or internalised during cell growth. Optical sections through the upper and lower OM did not provide any evidence for accumulation of fluorescently labelled background LPS at the poles. Cytoplasmic (mid-cell) sections showed no intracellular accumulation of fluorescence over time. Background LPS distributions are maintained in OM during cell elongation, which is consistent with the absence of both binary partitioning and internal recycling. Dashed outlines indicate cell boundaries. Scale bars: 1.0 µm.

To identify the potential mechanisms responsible for LPS turnover in the OM of *E. coli*, we tested whether background LPS was recycled internally, or partitioned directionally during division, analogous to the behaviour of OMPs during cell elongation^32^. If LPS were partitioned in the same way as OMPs, one would expect its removal from the OM because of division via progressive polar enrichment of older material and dilution in progeny, without requiring any additional turnover mechanism such as OM shedding. Alternatively, old LPS could conceivably be internalised into the cell and recycled internally or redistributed within the envelope (for example, via envelope-spanning processes rather than direct cytoplasmic recycling)^50^. However, previous studies have suggested that buildup of LPS in the inner membrane is toxic to cells, making an active recycling pathway unlikely^51–56^. To investigate these possibilities, we performed time-lapse 3D-structured illumination super-resolution microscopy (3D-SIM) to track background LPS dynamics in the OM of growing cells after fluorescently labelling background Kdo-N_3_ containing LPS using copper-free click (SPAAC)^57,58^. After mounting on M9 CDM/agarose pads, viable cells with AF488-labelled LPS were imaged over multiple generations at 37 °C with 3D-SIM^2^, enabling optical sectioning in the z-direction and isolation of background fluorescent LPS signal in the upper cell envelope, cytoplasm and lower cell envelope (Fig. 5 and Supplementary Fig. 5). Rates of cell elongation and division were assessed (Supplementary Fig. 5d) to ensure that growth was unaffected by labelling or light exposure under the imaging conditions used. We found no evidence for intracellular accumulation or binary partitioning of background LPS during cell growth. Instead, fluorescently labelled LPS remained in discrete patches across the OM and was inherited uniformly by daughter cells without polar enrichment (Fig. 5, Supplementary Fig. 5 and Supplementary Movies 1-3). These live-cell observations are consistent with our fixed-cell dSTORM data (Figs. 1–3).

To test whether background LPS signal loss could be visualised directly in live cells over an extended time window, we acquired additional 3D-SIM^2^ time-course images under reduced-photobleaching conditions. Image acquisition was modified by reducing the number of SIM phases from 13 to 9 and lowering the 488 nm excitation power, thereby extending the imaging time window, while retaining sufficient OM-associated AF488 fluorescence signal. Under these conditions, AF488-labelled background LPS could be monitored in live cells for up to 120 min (Supplementary Fig. 19). Throughout these time course experiments, the labelled background LPS signal retained a punctate, clustered distribution, while the signal from these LPS-rich clusters decreased gradually over time. No time-dependent polar enrichment or coalescence of LPS-rich clusters was observed. These extended time course live-cell 3D-SIM^2^ data provide a direct visual confirmation of background LPS signal loss in individual dividing cells under reduced-photobleaching microscopy conditions, while fixed-cell dSTORM and biochemical membrane fractionation are used to directly quantify background LPS turnover rates.

To corroborate these imaging-based observations biochemically over the same period of growth (initiated at OD_600_ = 1.5 and sampled for 120 min thereafter) (Fig. 5, Supplementary Fig. 5 and 19), we fractionated identically labelled cultures into soluble, inner membrane and OM compartments. We observed AF488-labelled background LPS predominantly in the OM fraction, with no time-dependent enrichment in the soluble fraction (Supplementary Fig. 14). Together, these imaging and biochemical data indicate that the observed decline in background LPS signal is not explained by binary partitioning (as seen for OMPs) or by internal cytoplasmic recycling^32^

### Outer membrane vesicle biogenesis contributes to LPS turnover in stationary phase *E. coli* cells

Our data show that in stationary phase, newly synthesised LPS insertion and background LPS turnover in the OM are maintained despite a slowing in cell elongation and division (Fig. 2–4, Supplementary Figs. 2 and 3c). Over the time window analysed, CFU mL^−1^ remained approximately stable (Supplementary Fig. 11, Supplementary Tables 2 and 3), indicating minimal net loss of viability; thus, stationary phase here reflects negligible net population growth rather than population stagnation dominated by cell death. Accordingly, cells with slowed growth must actively remove LPS to maintain OM homoeostasis and permit continued LPS insertion in the absence of cell elongation and associated OM expansion. Having excluded binary partitioning and intracellular recycling of background LPS, we next investigated whether OMV biogenesis might mediate LPS turnover in stationary-phase cells^59–62^.

**Fig. 6:**
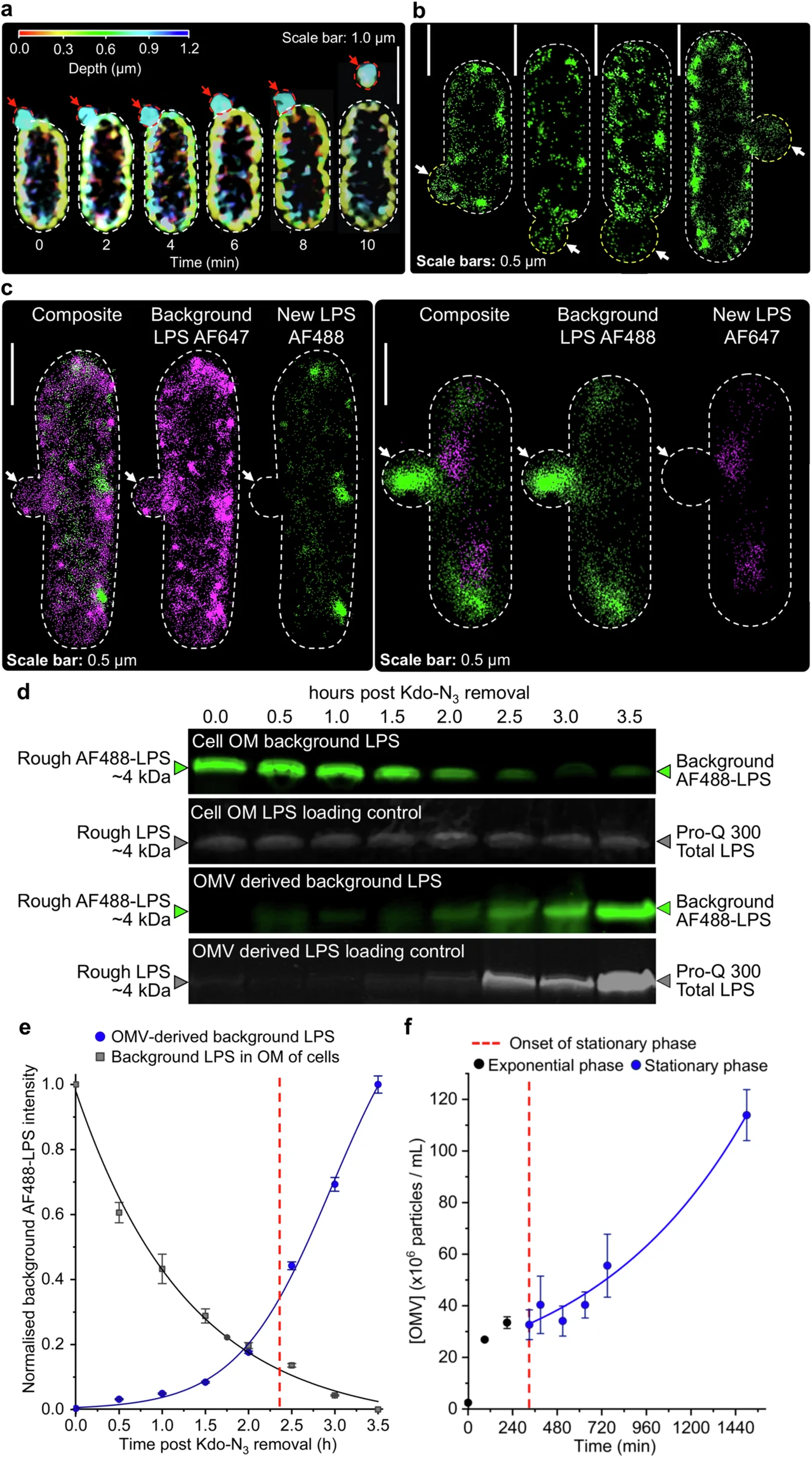
Preferential removal of background LPS from OM of stationary phase *E. coli* BW25113 cells by vesiculation. **a** Live-cell 3D-SIM² visualisation of OMV formation in a stationary phase cell. Depth-coded 3D projection shows an OMV budding and releasing from cell surface (red arrows). OMV is enriched in AF488-labelled background LPS. Dashed outlines indicate cell or OMV boundaries. Scale bar: 1.0 µm. **b** dSTORM images of stationary phase cells in the process of shedding background LPS via membrane protrusions. Dashed outlines indicate cell or OMV boundaries. Scale bars: 0.5 µm. **c** Representative two-colour dSTORM images showing the spatial distribution of fluorescently labelled background and newly inserted LPS in OM during OMV formation. Background and newly inserted LPS were metabolically labelled with Kdo-alkyne and Kdo-N_3_, respectively, in a pulse–chase experiment. Dual-colour differential fluorescent labelling was performed by sequential CuAAC reactions using appropriately functionalised dyes. Background LPS (magenta in lefthand images, green in righthand images) was enriched in the vesicle-like membrane protrusions (white arrows), while newly inserted LPS (green in lefthand images, magenta in righthand images) was depleted. Dashed outlines indicate cell boundaries. Scale bars: 0.5 μm. **d** Biochemical characterisation of background LPS in OM of intact cells and in released OMVs as a function of time by TSDS-PAGE. LPS extracted from an equivalent number of intact cells was loaded in each lane. For the OMV-derived LPS fractions, an equal volume of culture was centrifuged to remove intact cells and LPS was extracted from the resulting supernatant (equivalent volume of supernatant was loaded in each lane). The position and apparent molecular weight of rough LPS are indicated on each gel image. Pro-Q Emerald 300-stained rough LPS loading controls confirm the total amount of LPS loaded in each lane. **e** Quantification of background LPS levels in OM (filled squares) and in released OMVs (filled circles) as a function of time in pulse-chase experiment (mean ± S.E.M., *N* = 3 biological replicates). Red vertical dashed line indicates onset of stationary phase. **f** OMV concentration in bacterial culture during exponential and stationary phases of growth measured using nanoparticle tracking analysis. Each data point represents the mean OMV concentration ( ± S.D.) measured for technical replicates (*N* = 3) on a representative sample. The red vertical dashed line indicates onset of stationary phase. Absolute time-to-stationary phase differs from (**e**) because this growth curve was initiated from a lower starting OD_600_ ( ≈ 0.1) compared to the pulse-chase experiment (OD_600_ ≈ 0.5). OMV concentration increase during stationary phase was well described by an exponential function (*R*^2^ = 0.97).

Using live-cell 3D-SIM^2^, we directly visualised OMV biogenesis in stationary-phase cells presenting fluorescently labelled background LPS (Fig. 6a and Supplementary Fig. 12a,b). Across independent live-cell imaging replicates (≥30 min time courses), we observed the emergence of discrete, intensely fluorescent extracellular puncta and OMV-like structures adjacent to intact cells that exhibited higher fluorescence than the contiguous OM signal, consistent with enrichment of labelled background LPS in vesicle-like structures (Supplementary Fig. 12a,b). In a representative widefield view, fourteen visible OMV-like structures were observed in the vicinity of 72 intact cells (0.194 visible OMV-like structures per cell; Supplementary Fig. 12b), noting that this fluorescence-based count is a lower limit because the smallest vesicles fall below the practical resolution and sensitivity limits of fluorescence microscopy. We captured a complete budding-and-release sequence in which a dynamic OM buckle or nucleation progressively expanded and subsequently detached as a discrete vesicle over a 10 min period (Fig. 6a). Capturing full scission events in 3D by live-cell super-resolution microscopy is technically challenging because vesiculation is transient and stochastic, and sufficient spatiotemporal resolution requires balancing illumination, acquisition speed and photophysics while maintaining cell viability.

dSTORM imaging provided complementary support for OMV biogenesis and background-LPS shedding in stationary phase (Fig. 6b,c and Supplementary Fig. 12c,d). Unlike 3D-SIM^2^, OMV-producing cells cannot be reliably identified during dSTORM acquisition, and vesicle-like protrusions and OMV-like structures are typically confirmed only after localisation and reconstruction, necessitating screening of multiple cells and fields of view to capture vesiculation events. Despite this constraint, single-colour dSTORM frequently revealed LPS-rich puncta and vesicle-like protrusions consistent with nascent or recently released OMVs on stationary-phase cells (Fig. 6b and Supplementary Fig. 12c). In two-colour dSTORM experiments where pre-existing background LPS and newly inserted LPS were differentially labelled, OMV-like protrusions were consistently enriched in labelled background LPS and depleted in labelled newly inserted LPS relative to the surrounding OM. This enrichment of background LPS was observed even after swapping the dye labels (Fig. 6c and Supplementary Fig. 12d). Supplementary Fig. 12d shows representative two-colour reconstructions (including with dye labels swapped), and Supplementary Fig. 13 presents region of interest (ROI)-based localisation-density quantification of OMV/bleb protrusions versus intact-cell ROIs across a larger dataset (*n* = 30 cells, *N* = 3 biological replicates) which confirmed strong enrichment of background/old LPS in OMV ROIs (median log_2_(*S*_*old LPS*_) = +2.07; 4.2-fold enrichment) and depletion of newly inserted LPS (median log_2_(*S*_*new LPS*_) = –0.55; 0.68-fold depletion) relative to the surrounding OM (Supplementary Fig. 13 and Supplementary Table 24). Together, these data support a model in which stationary phase cells preferentially shed OM regions that are enriched in older background LPS via OMVs as part of growth-independent OM remodelling. Similar preferential incorporation of older LPS into OMVs has been previously observed in *E. coli*^63^ and in *Salmonella spp*. responding to antimicrobial peptide stress^62^, suggestive of a conserved OMV-based mechanism among Gram-negative bacteria in which OMV-blebbing is used for remodelling of OM LPS content.

To quantitatively assess OMV-mediated LPS turnover, we measured changes in the distribution of fluorescently labelled, background LPS between outer membranes isolated from intact cells and an OMV fraction isolated from culture supernatants (Fig. 6d and Supplementary Fig. 6c). Biochemical analysis of matched OM and OMV isolates confirmed OMV-associated LPS via TSDS-PAGE analysis followed by silver staining, whereas OMV-associated bulk protein signal was below the Coomassie detection limit at cell density-equivalent loadings (Supplementary Fig. 18). As cultures entered stationary phase and cell growth ceased, OM-associated background LPS fluorescence continued to decrease and was mirrored by a corresponding increase in OMV-associated fluorescence (Fig. 6e). In parallel, nanoparticle tracking analysis measurements revealed an increase in OMV abundance as cultures transitioned into stationary phase (Fig. 6f, Supplementary Figs. 11c,d and 16a,b).

To test whether the measured vesiculation rates can quantitatively account for the observed decline in background-labelled LPS fluorescence, we compared TSDS-PAGE–derived background LPS fluorescence decline with a dilution-only expectation calculated from population-level growth and OM expansion, and then utilised nanoparticle tracking analysis-derived OMV abundance and size distributions to estimate OMV-associated export of the background LPS pool (Supplementary Fig. 16c,d). This analysis shows that the background LPS fluorescence signal decline measured by TSDS-PAGE is significantly faster than expected from dilution alone and that OMV export can account for a considerable component of the excess decline beyond cell multiplication-driven dilution (Supplementary Fig. 16c,d). Because OMV recovery and particle counts can be reduced during supernatant processing and OMV isolation (e.g. resulting from filtration, high-speed prolonged centrifugation and adsorption losses), this estimate should be interpreted as a conservative lower bound on OMV-mediated export of the pre-labelled background LPS pool. Thus, our imaging and biochemical data provide direct evidence that stationary-phase wild-type *E. coli* can shed background LPS via OMV release under growth-limited conditions, thereby creating space in the OM that could permit continued incorporation of newly synthesised LPS when cell elongation has slowed.

Native OMV production was also assessed by growing the *E. coli* BW25113 strain in the same M9 CDM used for LPS metabolic labelling, removing aliquots of the culture as a function of time, and measuring particle concentrations in these processed supernatants by nanoparticle tracking analysis (NTA)^64–66^. OMV particle concentrations per mL are shown in Supplementary Fig. 11c, and normalisation to viable cell concentration (CFU equivalents) enables per-cell quantification of extracellular OMV accumulation during stationary phase (Supplementary Fig. 11b,d)*.* From these data, an apparent net accumulation rate per viable cell can be estimated from the slope of the OMVs/CFU trace (Supplementary Fig. 11d). Because this growth/NTA time course was initiated at a lower starting OD_600_ ( ≈ 0.1) than the pulse–chase experiments (initiated at OD_600_ ≈ 0.5, Figs. 2–4 and 6d,e), the absolute time to stationary phase onset differs between assays. The OMV concentration increased by ~14-fold during exponential growth (Fig. 6f), reflecting accumulation of vesicles in the culture as the population expands. This increase in OMV concentration coincided with the initial appearance of AF488-labelled background-LPS signal in the OMV fraction analysed by TSDS-PAGE (Fig. 6d,e). OMV release during exponential phase growth has been observed in wild-type Gram-negative bacterial strains^67^. However, during stationary phase the OMV particle concentration continued to rise by ~1.7-fold between 390 and 750 min post-inoculation and by a further ~2-fold between 750 and 1500 min (Fig. 6f), even though the bacterial cell density (OD_600_) and viable cell concentration in the culture did not change (Supplementary Table 23 and Supplementary Fig. 11b). Consistent with this, CFU mL^−1^ remained stable (within experimental variation) across stationary phase (390–1500 min post-inoculation) (Supplementary Table 23 and Supplementary Fig. 11b).

These results confirm that OMV production begins during mid-to-late exponential growth but also reveal that OMV production occurs continuously during stationary phase even when the cells are not multiplying. Furthermore, these results support our observation that bacterial cells continue to incorporate LPS metabolically labelled with Kdo-N_3_ in the OM during stationary phase even though the viable cells present are not elongating (Supplementary Figs. 7 and 11a). We propose that, during stationary phase, OMVs form preferentially from OM regions enriched in the pre-existing (background) LPS pool. This is supported by  single-cell measurements (Fig. 6c, Supplementary Fig. 13 and Supplementary Table 24), together with its accumulation in isolated OMV fractions at the population level (Fig. 6d). This enrichment reflects preferential partitioning of the labelled background pool into OM buckle/bleb ROIs and does not, by itself, imply a dedicated old-versus-new sorting mechanism.

This OMV-mediated LPS turnover would allow stationary phase *E. coli* to continually remodel and rejuvenate their OM composition, independently of cell growth. Preferential export of the older, pre-labelled LPS pool through OMV shedding supports a mechanism of OM homoeostasis, likely crucial for adaptation and survival under diverse environmental stresses that affect OM integrity (e.g. changes in temperature, local divalent cation concentration or exposure to cationic antimicrobial peptides)^11,68,69^.

### Outer membrane buckling model predicts the critical OMV size

The mean diameter of the OMVs produced during the different phases of bacterial growth was also determined by NTA (Supplementary Fig. 8a) and the size did not change appreciably across growth phases (≈80–95 nm, Supplementary Fig. 8b). This lack of dependence on growth phase for OMV size has been previously observed in *Neisseria meningitidis*^66^. The OMV size distribution obtained for stationary phase *E. coli* BW25113 cultures (Supplementary Fig. 8b, time = 1500 min) was also consistent with previous work on the same strain^70^. We have interpreted this observation as evidence that OMV biogenesis is governed by a characteristic (critical) vesicle size.

**Fig. 7:**
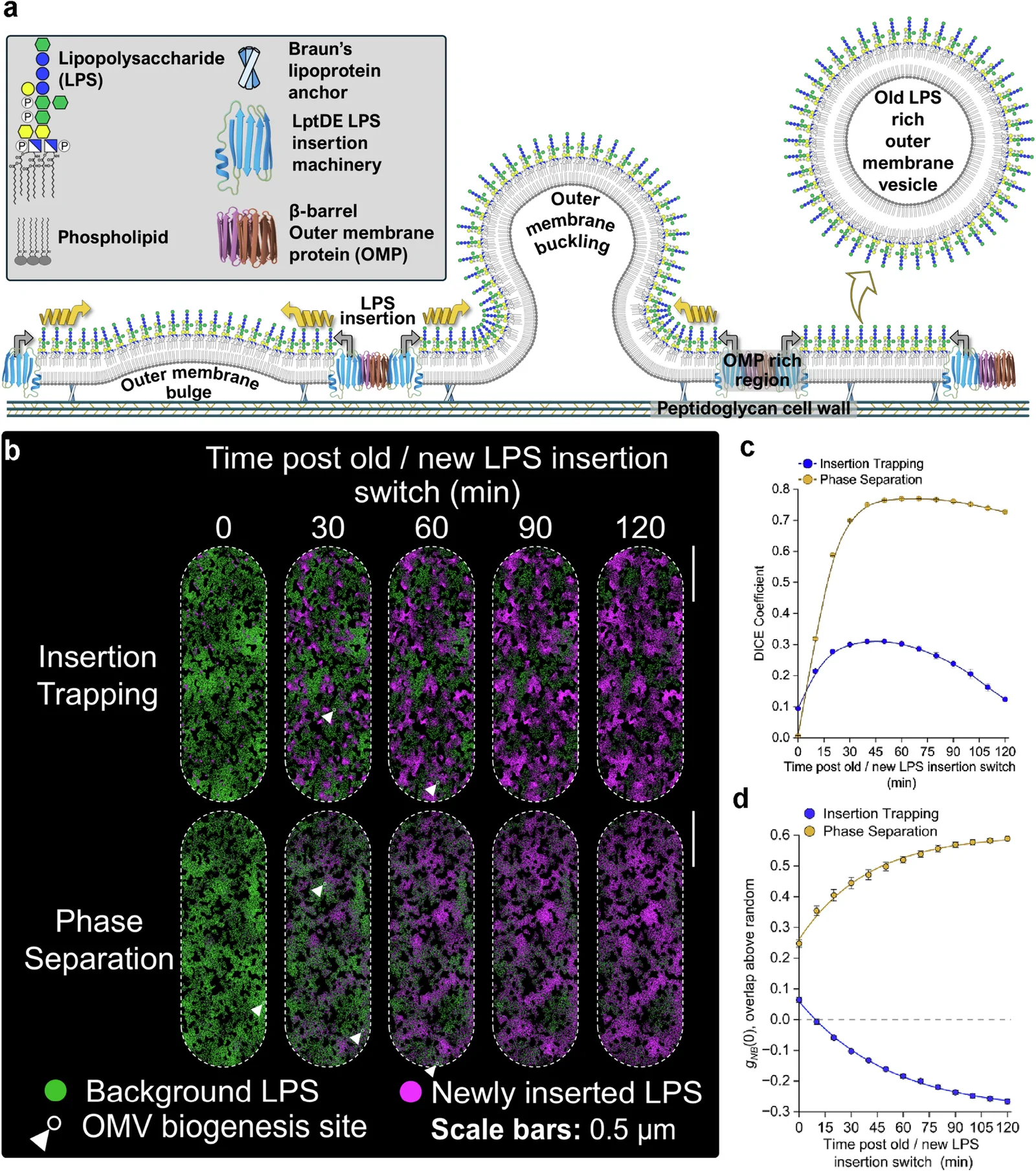
Modelling OMV formation and LPS turnover in the bacterial outer membrane. **a** Model for OMV formation in OM of a stationary phase *E. coli* cell. **1)** Continuous insertion of new LPS (grey arrows) by LPS insertion machinery (LptDE) into LPS-rich region increases local packing density while PG expansion lags due to slow rate of cell elongation in stationary phase. This asymmetric expansion of OM relative to PG induces in-plane compressive stress in OM (orange arrows) resulting in a bulge in the old LPS-rich regions. LPS patch rims are pinned by OMP-rich regions and PG-anchored zones (e.g. by Braun’s lipoprotein). **2)** Bulge grows into a bleb forming a neck between the surrounding OM and the protruding membrane. **3)** Bleb pinches off to produce an OMV, thereby removing background (old) LPS locally and releasing compressive stress in OM. This stress-release results in flattening of the OM around the vesiculation site. **b** Particle-based simulations of OM under two different organisation models: insertion-trapping model (top) and phase-separation model (bottom). Newly inserted LPS (magenta) and background LPS (green) regions are colour coded as in Fig. 2b. Dashed outlines indicate cell boundaries. LPS-inaccessible, OMP-rich regions (black) and OMV biogenesis events (white arrowheads/circles) are also indicated. Scale bars: 0.5 µm. **c** DICE analysis of newly inserted and background LPS co-clustering in both OM organisation models: phase-separation model (orange) and insertion-trapping model (blue). DICE coefficient values for each time point are a mean ( ± S.E.M.) calculated from five independent simulation runs per model. DICE is unitless (ranging from 0 to 1) and denotes the normalised old-new overlap. **d** Zero-lag coverage-normalised excess overlap of cluster masks (*g*_*NB*_(0)) quantifying colocalisation of newly inserted and background LPS relative to random placement in OM (horizontal dashed line at 0). Phase-separation model (orange) yields positive *g*_*NB*_(0) values that increase and saturate, reflecting growing co-location. *g*_*NB*_(0) values for insertion-trapping model (blue) remain near or below zero and become more negative with time, indicating anti-association. *g*_*NB*_(0) values for each time point are a mean ( ± S.E.M.) calculated from five independent simulation runs per model.

Asymmetric growth of the OM relative to the peptidoglycan (PG) cell wall has previously been postulated to favour local outward bulging of the OM, perhaps biased towards regions where a paucity of bonds exists between the PG and Braun’s lipoprotein (Lpp)^67^. In-line with this framework, OMVs purified under our conditions were depleted in Coomassie-detectable proteins while remaining LPS-rich relative to matched OM fractions (Supplementary Fig. 18). We developed an OM buckling model (Fig. 7a) to test whether accumulation of compressive stress in these membrane regions due to asymmetric growth of the OM relative to the cell wall was sufficient to cause buckling given the intrinsic curvature and physical stiffness of the OM (see Methods). Because cell elongation slows on entry into stationary phase while LPS incorporation continues (Figs. 2, 3 and 6, Supplementary Fig. 11a,b), this compressive stress is expected to become more widespread. Continued LPS insertion elevates in-plane compressive stress within LPS-dense OM patches mechanically constrained at their boundaries by OMP-rich domains and OM–PG anchoring by Braun’s lipoprotein (Fig. 7a), aligning with the load-bearing role of the OM and known mechanics of OM-PG tethers^5,71^. Increased vesiculation is known to occur with weakened OM-PG tethers^72,73^.

The OM buckling model (Fig. 7a) was used to predict the critical size of the OMV released in background LPS-rich regions (see Methods for model calculations), which can be compared to our experimentally determined diameters (Supplementary Fig. 8b, see Methods for model calculations). When a bulging LPS-rich OM patch exceeds a characteristic radius (*R*_*b*_), a bleb forms and is released as an OMV with a critical diameter (*d*). We estimated *d* ≈ 73-122 nm for OMVs released by this membrane buckling mechanism which agrees with our experimental results (Supplementary Fig. 8b, grey shaded region). This compressive stress-release by OM blebbing is consistent with bilayer-coupled, leaflet-asymmetry routes to OMV formation^74,75^ and with broad OMV biogenesis frameworks^59,60^.

### Particle-based simulations of LPS spatiotemporal dynamics distinguish the insertion-trapping mechanism from phase separation in the OM

To test whether the spatiotemporal LPS behaviour observed by pulse-chase dSTORM (Figs. 2 and 4, Supplementary Fig. 2) requires equilibrium phase separation, we compared two particle-based simulations of the *E. coli* OM that share identical, experimentally-derived LPS turnover rates (Figs. 2, 3 and 6) but differ in their organising rules (Fig. 7). The simulated field covers half of the OM surface of the cell (2.6 µm × 1.1 µm) and contains a static allowed mask (75% of the area) representing LPS-accessible membrane^76–78^. The remaining area (25%) is treated as OMP-rich, LPS-inaccessible voids (black regions in schematics), thereby recapitulating the heterogeneous, clustered OMP/LPS organisation observed in previous experimental studies^10,11,33^. The total LPS in the accessible area is held approximately constant (target *N* ≈ 8 × 10^4^ LPS simulation particles, ~9-fold coarse-graining of the expected biological copy number^77–79^). LPS turnover and insertion rates were informed by experimental data (Figs. 2, 3 and 6). Background (old) LPS decays with a half-life of ~90 min, newly inserted (new) LPS rises to ~65% by 120 min; and OMV events are placed stochastically at LPS-density maxima with realistic footprints (80–90 nm diameters) and at frequencies matched to experimental data (Fig. 6 and 7a,b). These constraints reproduce the measured turnover (reciprocal changes in old and new LPS coverage, and weak old-new co-clustering around ~60 min, Fig. 2f) and ensure that any divergence between models reflects organisation rather than bulk turnover/insertion rates. To facilitate robust quantitative comparison with the imaging analyses, cluster masks were defined by DBSCAN using identical cluster-defining parameters (*ε* = 30 nm; *minPts* = 7; core points only). We tracked the normalised old–new overlap (DICE, Sørensen–Dice coefficient) and zero-lag coverage-normalised excess overlap of cluster masks (*g*_*NB*_(0)), where values near 0 indicate overlap expected under random colocalisation, negative values indicate segregation (anti-association), and positive values indicate co-localisation (Fig. 7c,d).

The insertion-trapping model was designed to reproduce our experimental observations and incorporated the restricted diffusion of LPS in the OM (Fig. 7b and Supplementary Fig. 9a). The organising rule is conceptually consistent with the stochastic, spatially localised insertion and growth-dependent patterning previously observed for LamB dynamics by Ursell et al.^13^. Here, it is constrained and tested using direct nanoscale measurements of LPS turnover and old/new LPS segregation, and it incorporates growth-independent loss via OMV shedding. New LPS is inserted in short bursts at a changing subset of pre-sampled hotspots located across the entire OM surface (consistent with the mapped LptD-linked LPS delivery sites characterised in Fig. 1d)^10,36^, while both background and newly inserted LPS undergo strongly confined lateral diffusion in-line with previous observations^10,11^.

Background LPS loss is biased away from newly inserted LPS-rich locations at the edges of OMP-rich, LPS-inaccessible voids, and includes growth-independent loss via OMV shedding. OMV budding sites are drawn from regions with the highest LPS density. This model reproduces the following three key experimental observations. Despite substantial turnover, the model preserves spatial segregation (Fig. 7b-d), the normalised old–new overlap or co-clustering stays low with a weak maximum at ~60 min (Figs. 2 and 7c, Supplementary Fig. 9 and Supplementary Movie 4), and *g*_*NB*_(0) remains ≤0 from 10 to 120 min (Fig. 7d). Cluster sizes of new LPS patches broaden modestly as coverage grows, as was observed experimentally (Fig. 4), but there is no late stage, OM wide coarsening*.*

The phase separation model was used to emulate old and new LPS co-clustering in the OM due to phase separation while keeping all bulk rates and the restricted lateral diffusion of LPS particles identical to the insertion-trapping model (Fig. 7b, Supplementary Fig. 9 and Supplementary Movie 4). We introduced a short-range like–like coupling constant (Ising/Cahn–Hilliard-style drift up the density gradient of each LPS type)^43^ with strength tuned so that global densities and OMV statistics match the output from the insertion-trapping model. In contrast to the insertion-trapping model and our experimental data, this model predicts the opposite time dependencies. The simulation shows a monotonic increase in the overlap of background and newly inserted LPS (Fig. 7c) a rise in *g*_*NB*_(0) above zero (Fig. 7d) and a gradual right-shift in domain-size distribution indicative of coarsening, which are hallmarks of phase ordering. These behaviours were not observed in our experimental data (Figs. 2 and 4)^43^.

We further connected spatiotemporal LPS dynamics to vesicle biogenesis by implementing a membrane buckling-based OMV biogenesis module in our simulations. In the simulation, this is captured phenomenologically by Poisson-distributed OMV formation events at LPS-density maxima that remove local background LPS while conserving total membrane area (i.e. without creating holes), reproducing the observed depletion of background LPS at vesiculation sites while maintaining overall membrane coverage (Figs. 6a,c and 7a,b, Supplementary Fig. 9). In this framework, buckling-driven vesiculation provides a mechanistic link between continued LPS insertion and preferential release of background-LPS-rich regions as OMVs. With experimental LPS turnover rates, the insertion-trapping model yielded persistent old-new LPS segregation, low, transient old–new LPS co-clustering, and an absence of coarsening, all consistent with our in vivo experimental data (Figs. 2, 4 and 6). This contrasts with the phase separation model, which, despite identical, experimentally derived turnover constraints predicts increased co-clustering of old and new LPS and progressive domain coarsening (Fig. 7b-d). These particle-based simulations support a kinetically trapped OM organisation maintained in a non-equilibrated state by spatially localised insertion and restricted lateral diffusion. OMV biogenesis driven by continuous LPS insertion contributes to preferential background LPS loss, assisting stationary phase *E. coli* cells in the remodelling of their OM LPS content in a growth-independent manner.

## Discussion

Our experiments reveal a dynamic, yet tightly constrained organisation of the LPS content in the *E. coli* OM. We found that LPS turnover in the OM is not static, even in stationary phase cells, where new LPS continues to be inserted and old LPS is preferentially removed (Figs. 2–4 and 6). This turnover occurs without any large-scale mixing of LPS within the membrane (Figs. 2 and 4). Instead, the OM maintains a solid-like, diffusion-limited organisation in which insertion-patterned LPS-rich patches are preserved over time. These findings support a refined model for OM organisation and homoeostasis in Gram-negative bacteria. In this model, the spatial arrangement of LPS is governed not by equilibrium-like phase ordering with domain coarsening into larger regions but by localised insertion events coupled with limited lateral diffusion that trap LPS molecules near their point of insertion (insertion-trapping mechanism) and preserve their organisation relative to other OM components (e.g. OMPs). Meanwhile, the removal of pre-existing (background) LPS from the OM in stationary phase cells, where passive dilution is hindered due to the absence of growth-dependent OM expansion, is assisted through vesiculation.

This work reshapes the way we consider OM organisation, while reinforcing the limited lateral mobility of LPS in the OM^10,11^. Using two-colour SRM, we directly visualised that newly inserted LPS molecules remain spatially segregated from older LPS-rich patches even after hours of incubation (Fig. 2). The persistence of distinct, newly inserted and background LPS patches in our pulse-chase experiments indicate minimal mixing between the two populations (Figs. 2–4). This provides a visual confirmation of what earlier single-particle tracking data had detected: LPS lateral diffusion in the OM is very slow (~10^−2^ μm²/s or lower)^10,11^ and constrained such that a LPS molecule essentially remains near its point of insertion. Our data show that even across multiple generations, LPS does not redistribute to homogeneity in the OM. Instead, each cohort of LPS inserted at a given time retains its identity as a cluster, frozen in place. Consequently, LPS does not undergo binary partitioning in the manner of OMPs^13,32^, which are inserted at mid-cell and become apportioned to new cell poles during cell elongation and division^32^. This means that in the absence of growth and division, old LPS could be expected to accumulate progressively in the OM, limiting the ability of the bacterium to remodel its lipid composition in response to environmental change. Our data indicate that *E. coli* can mitigate this by shedding older, background LPS via OMVs, enabling OM renewal and adaptation even in growth-limited cells. This process appears to be a fundamental aspect of OM maintenance in non-elongating *E. coli* cells. By generating OMVs, the bacterium can shed portions of its OM that are enriched in background or old LPS (Fig. 6c, Supplementary Fig. 13 and Supplementary Table 24), in line with prior observations in enteric bacteria^62,63,74,80^.

Our data indicate that OMVs are enriched for the pre-labelled (background/old) LPS pool, consistent with vesiculation contributing to background LPS turnover (Fig. 6a-d and Supplementary Fig. 12c,d for representative reconstructions, Supplementary Fig. 13 for ROI-based quantification, and Supplementary Table 24), but this enrichment does not imply exclusive packaging or an active old-versus-new sorting mechanism. At present, we have no evidence for the existence of cellular mechanisms capable of distinguishing between LPS cohorts based on their relative age; instead, an explanation consistent with our data is that this background LPS enrichment in OMVs emerges from OM spatial organisation and envelope mechanics. Newly inserted LPS is delivered at discrete LptD-associated insertion sites (Fig. 1b-e) and remains spatially confined and segregated from the pre-existing LPS pool (Figs. 2, 3a and 4b), which would bias the background LPS population to become relatively concentrated over time within LPS-rich regions that are spatially separated from highly tethered OMP-rich zones. In the context of our OM buckling model (Fig. 7a), such LPS-rich regions being comparatively less constrained would make them prone to membrane deformation and budding, providing a physical basis for preferential enrichment of the background LPS pool in released vesicles without requiring a means to actively discriminate between different LPS subpopulations (Fig. 7a)^5,6,32^. Consistent with this view, OMV production increases as stationary phase ensues (Fig. 6f and Supplementary Fig. 11c,d), coincident with continued new LPS insertion and decrease of the background-labelled pool (Figs. 3 and 4, Supplementary Fig. 10). This behaviour aligns with prior work linking vesiculation with envelope remodelling and stress responses, including LPS remodelling-associated vesiculation in enteric bacteria^60–62^. Moreover, our simulations, incorporating buckling-driven vesiculation reproduce key qualitative features of our experimental data; loss of the background-labelled LPS without domain coalescence and preservation of spatially discrete patches, supporting a model in which OMV shedding, driven by compressive stress, can contribute to background LPS turnover in non-growing stationary-phase cells (Fig. 7, Supplementary Figs. 9 and 12, Supplementary Movie 4).

Our time-lapse imaging (Fig. 6a-c and Supplementary Fig. 12) captured nascent vesicles with old LPS budding off cells, and our bulk fractionation experiments showed a clear transfer of background LPS from the OM of cells to vesicles, with both events occurring as stationary phase ensued (Fig. 6d and Supplementary Fig. 6c). We observed a substantial increase in OMV production as bacterial growth slowed (Fig. 6f and Supplementary Fig. 11c,d), which suggests that vesicle shedding is increased in response to entry into stationary phase, consistent with prior reports of elevated OMV formation under stress or nutrient limitation^81–83^. This vesicle-mediated LPS turnover allows the cell to maintain OM homoeostasis by removing background LPS and replacing it with newly synthesised molecules, even when the cell is not dividing. Such a mechanism is likely important for preserving OM integrity and functionality during a prolonged period in the stationary phase, and for facilitating adaptation in situations where growth is arrested due to nutrient-limited conditions when the environment may still be challenging (for example, in host tissues, acidic lysosomes post-phagocytosis or in biofilms). By discarding a portion of its endotoxin via OMVs, a bacterial cell might also modulate its interactions with the host immune system or neighbouring microbes^84^, though the specific consequences of shedding background-enriched LPS remain to be explored.

To test whether the observed spatiotemporal dynamics arise from phase separation or kinetic-trapping, we built simulations of OM dynamics constrained by experimental rates of LPS turnover (Fig. 7, Supplementary Fig. 9 and Supplementary Movie 4). Ursell et al. combined time-lapse fluorescence microscopy of an OMP reporter with mathematical modelling to infer that stochastic, spatially localised OMP insertion and growth-driven displacement can generate polar enrichment of older material when lateral mobility is restricted^13^. Our insertion-trapping model is conceptually consistent with localised insertion and constrained redistribution^11,13,32^, but here it is constrained by experimentally quantified parameters and evaluated using two-colour single-molecule nanoscopic measurements of LPS turnover and old/new LPS segregation in the outer leaflet, incorporating growth-independent LPS loss through OMV shedding. The insertion-trapping model (Fig. 7b and Supplementary Fig. 9) yielded stable, discrete patches of newly inserted and background LPS (Figs. 1, 2 and 7, Supplementary Figs. 9 and 10, Supplementary Movie 4), loss of background LPS without coarsening, and sustained spatial segregation, quantified by a weak maximum in the old-new LPS overlap, and *g*_*NB*_(0) ≤ 0 across 0–120 min (Figs. 2, 4 and 7c,d)*.* Therefore, this model closely reproduced the hallmark features seen in the outputs of the pulse-chase / dSTORM experiments (Figs. 2–4)*.*

In sharp contrast, a phase separation model with identical bulk LPS insertion rates (Fig. 7b and Supplementary Fig. 9a) showed contrasting LPS spatiotemporal dynamics, yielding instead characteristics typical of phase ordering and liquid–liquid demixing in model membranes^85,86^. These included a monotonic rise in colocalisation (*g*_*NB*_(0) > 0)^43,44^, and growth of the LPS domain size (Fig. 7c,d). This phase separation model was implemented with the same restricted lateral membrane diffusion and matched LPS turnover constraints as the insertion-trapping model, differing only in the organising rules. Incorporation of bleb-driven OM vesiculation into the simulations (Fig. 7a) provided a biophysical mechanism for how an increase in local compressive membrane stress due to continued LPS insertion could drive OMV release. Poisson distributed vesiculation events nucleated at LPS particle density maxima, away from OMP-rich patches (black areas) where LPS-rich patch centres are weakly anchored to PG, resulting in the removal of old LPS content from the OM whilst relieving the compressive stress associated with continuous LPS insertion into the OM of a non-elongating cell without leaving persistent voids. The simulation outputs from the kinetic trapping model mirrored our cell-by-cell dSTORM image analyses and population scale biochemical experimental outputs (Figs. 2, 3 and 6), and aligned with established OM mechanics and OMV biogenesis paradigms^59,60,72–75^. Together, these particle-based simulations support a kinetically-trapped, non-equilibrium OM organisation, the properties of which are dictated by site-specific insertion of LPS, restricted lateral diffusion of OM components^10,11,32^ and OMV-mediated preferential removal of older LPS. The concept of a kinetically-trapped OM organisation, recapitulated by the simulations, also explains the stable coexistence of LPS-rich and OMP-rich domains without the need for equilibrium (thermodynamically-driven) phase separation^10,11^. In classical phase separation, one would expect coarsening over time with small domains merging into fewer, larger domains to minimise interfacial energy.

We see no evidence of such coarsening (Figs. 2 and 4, Supplementary Fig. 10). Instead, the patterns we observed (many small LPS patches that persist and do not fuse) align with a scenario where membrane components are inserted in certain areas and then constrained from moving throughout the OM (Figs. 2 and 4). Our clustering analysis showed that new LPS clusters proliferate in number but not in size, and that old clusters dissipate without merging into others (Fig. 4 and Supplementary Fig. 10). This is inconsistent with Ostwald ripening and other phase separation phenomena, and is better described by a diffusion-limited, far-from-equilibrium process.

In essence, the crowded, rugged energy landscape of the OM (shaped by strong LPS–LPS, OMP-OMP and OMP-LPS interactions, peptidoglycan tethers, and restricted lateral mobility) means that whatever arrangement of molecules is established through insertion is essentially trapped in place, giving rise to a patchwork of persistent nanodomains. Our findings thus bridge mechanistic insights at the molecular level (LPS transport and diffusion) with the mesoscopic organisation of the membrane.

In summary, this study highlights a previously unappreciated dynamism in the *E. coli* OM. We show that, far from being a static barrier, the OM actively undergoes renewal even in stationary phase. New LPS continues to be incorporated, and background LPS is continuously shed via OMVs (Fig. 6, Supplementary Figs. 12 and 13, Supplementary Table 24). This mode of LPS turnover ensures that the OM can be remodelled and adapt to changing conditions without requiring cell growth or division. The advanced imaging and labelling strategies we employed were crucial for uncovering these phenomena, enabling us to directly observe LPS being inserted and removed in vivo. In future studies, the high spatial precision methodologies employed here for studying LPS turnover, might also be applied in the exploration of turnover (or reorganisation) of other OM components (such as lipoproteins or certain OMPs) in non-dividing cells, and to determine whether similar vesicle-mediated processes occur in other Gram-negative species. More broadly, our work emphasises the importance of OM vesiculation as not only a means of secreting molecules but also a mechanism for turnover and renewal of its fundamental glycolipid component. Understanding how bacteria manage the composition and organisation of their envelopes in different growth states could inform new strategies to target their vulnerabilities, especially in contexts like chronic infections where many invading pathogenic Gram-negative cells are in a growth-limited state.

## Methods

### Bacterial strains and plasmids

Details of strains used in this study are provided in Supplementary Table 1. The Keio collection mutant of *Escherichia coli* K12 subsp. BW25113 used in this work (Δ*ompA*) was validated previously via PCR, confirming the presence of a kanamycin resistance cassette within the open-reading frame of interest^32,87,88^.

pBAD-lptD* and pBAD-ompA* plasmids were produced via Gibson isothermal assembly^89^. PCR was used to amplify the *lptD* and ompA gene inserts (see Supplementary Table 2 for primers) from purified *E. coli* K12 subsp. MG1655 genomic DNA. The amplified genes were cloned into pBADcLIC vectors using a commercial Gibson isothermal assembly kit (NEBuilder® HiFi DNA Assembly). The pBADcLIC vector system was selected because its *araBAD* promoter provides tight regulation yet permits low-level basal (leaky) expression in the absence of inducer (L-arabinose), allowing sufficient outer membrane protein production while minimising toxicity and misfolding that can occur under the strong induction conditions typical of IPTG-inducible recombinant expression systems; no L-arabinose was added in any experiment, lptD* and ompA* expression relied on basal *araBAD* promoter activity (leaky expression). PCR site-directed mutagenesis (Supplementary Table 2) was then used to incorporate the amber stop codon TAG in place of the codon for the D600 residue in *lptD* (Supplementary Fig. 1b) and E89 residue in *ompA*. Successful cloning and mutagenesis were verified via DNA sequencing (Eurofins Genomics LLC). The pEVOL-pylRS plasmid, encoding the pyrrolysyl-tRNA synthetase/pyrrolysyl-tRNA pair from *Methanosarcina mazei* was obtained from Dr. Edward Lemke (EMBL Heidelberg) under MTA^25,90^.

pBAD-lptD* and pEVOL-pylRS were co-transformed into *E. coli* BW25113 via electroporation (MicroPulser, Bio-Rad Laboratories) (1.8 kV, 25 µF, time constant: 3.5 ms). *E. coli* BW25113 cells co-transformed with pBAD-lptD^*^ and pEVOL-pylRS were selected by spreading some of the electroporated culture on an Lysogeny Broth (Miller formulation, LB-Miller) agar plate containing 100 µg mL^−1^ ampicillin and 35 µg mL^−1^ chloramphenicol and incubating the plate overnight at 37 °C. Unless otherwise specified, the incubation of bacterial cultures was done at 37 °C with shaking (220 rpm) in a 50 mL screw-cap polypropylene tube. Liquid pre-cultures were prepared by inoculating 5 mL of supplemented M9 chemically defined medium (M9 CDM, Supplementary Table 3) with appropriate antibiotics (Supplementary Table 1) using a well-isolated single colony of the desired strain picked from a freshly streaked LB-Miller agar plate. Post-inoculation cultures were incubated for 6–8 h or until cell densities reached a minimum optical density at 600 nm (OD_600_) of 1.5.

Following plasmid purification (Qiagen^©^ Midi plasmid purification kit) and DNA sequencing (Eurofins Genomics LLC), the TAG-containing pBAD-ompA vector (pBAD-ompA*) and pEVOL-pyIRS were co-transformed into *E. coli* ∆*ompA* cells via electroporation as above. Co-transformed cells were selected using LB-Miller agar plates with 30 µg mL^−1^ kanamycin, 100 µg mL^−1^ ampicillin and 35 µg mL^−1^ chloramphenicol. A single colony from this plate was used to inoculate a volume of supplemented M9 CDM with kanamycin, ampicillin and chloramphenicol antibiotics. Following prolonged pre-culture incubation, the appropriate volume of pre-culture was used to inoculate a fresh batch of supplemented M9 CDM with ampicillin and chloramphenicol antibiotics to an OD_600_ of 0.05. The cultures were then incubated for 2–3 h.

### Production of 8-azido-3,8-dideoxy-D-manno-oct-2-ulosonic acid (Kdo-N_3_)

Kdo-N_3_ was synthesised and purified following established protocols^26,38^. The synthesised Kdo-N_3_ was utilised for all super-resolution fluorescence microscopy experiments (dSTORM and 3D-SIM^2^) and yielded the same results as commercially available Kdo-N_3_ (Click Chemistry Tools, Vector Laboratories, Inc.) used in more recent work. Freeze-dried stocks of the synthesised compound and the purchased compound were resuspended to a concentration of 400 mM in sterile, deionised water and stored at −20 °C until required.

### Production of 8-(4-pentynoylamido)-3-deoxy-D-manno-oct-2-ulosonic acid (Kdo-alkyne)

Kdo-alkyne was synthesised and purified following established protocols from commercially available Kdo-N_3_ and synthesised Kdo-N_3_^26,38^. The synthesised Kdo-N_3_ was utilised for all super-resolution fluorescence microscopy experiments (dSTORM and 3D-SIM^2^). Freeze-dried stocks of the synthesised compound were resuspended to a concentration of 400 mM in sterile, deionised water and stored at −20 °C until required.

### LPS metabolic labelling with Kdo-N_3_ or Kdo-alkyne (Kdo-analogues)

Pre-cultures were used to inoculate fresh supplemented M9 CDM charged with 4 mM of the Kdo-analogue (Kdo-N_3_ or Kdo-alkyne) to a starting OD_600_ of 0.05. Post-inoculation these metabolic labelling cultures were incubated for 12 – 14 h. Cultures were subsequently harvested upon reaching late stationary phase. Cells were isolated via centrifugation (8000 × *g*, 4ºC, 3 min) and washed three times with fresh volumes of supplemented M9 CDM to remove non-specifically bound Kdo-analogue from cell surfaces prior to fluorescent labelling of the LPS.

### In situ fluorescent labelling of Kdo-analogue-containing LPS inner core domains via Cu(I) catalysed azide(-N_3_)-alkyne cycloaddition (CuAAC)

Cells with Kdo-analogue-containing LPS in the OM were pelleted via centrifugation (10,000 × *g*, 4 °C, 3 min) and resuspended to an OD_600_ of 1.0 in Click-iT reaction mix prepared according to the manufacturer’s instructions (Click-iT Cell Reaction Buffer Kit, Molecular Probes^®^, Invitrogen) supplemented with 4 mM *N*-acetylneuraminic acid (Neu5Ac) and charged with the alkyne- (for Kdo-N_3_-containing LPS) or N_3_- (for Kdo-alkyne-containing LPS) functionalised fluorescent dye (concentrations are specified in Supplementary Table 4). The cell suspensions were transferred to sterile 2 mL microcentrifuge tubes and incubated on a rotary wheel (12 rpm, 80° incline relative to bench top) for 30 min at room temperature protected from light. Post-labelling suspensions were transferred to fresh sterile 2 mL microcentrifuge tubes and cells bearing fluorescent LPS were pelleted via centrifugation (10,000 × *g*, 4 °C, 3 min). The labelled cell pellets were washed a further three times with supplemented M9 CDM to ensure removal of residual fluorophore and Click-iT mix components.

### Live cell Kdo-N_3_-containing LPS in situ fluorescent labelling via Cu(I)-free strain promoted azide(-N_3_)-alkyne cycloaddition (SPAAC)

Washed cell samples with Kdo-N_3_-labelled LPS were diluted in supplemented M9 CDM to an OD_600_ of 1.0 and transferred to a sterile 2 mL microcentrifuge tube. The appropriate volume of a dibenzocyclooctyne-amine (DBCO)-functionalised AF488 dye was then added directly to the cell suspensions (Supplementary Table 5). The SPAAC labelling reactions were then incubated for 45 min at 30 °C on a rotary wheel (12 rpm, 80° incline relative to benchtop) protected from light. The cell suspensions were then each transferred to a fresh, sterile 2 mL microcentrifuge tube and pelleted via centrifugation (8000 × *g*, 4 °C, 2 min,). The labelled cell pellets were then gently washed three times in supplemented M9 CDM. Extra care was taken during resuspension of cell pellets to ensure cells were exposed to minimal levels of shear stress, thereby ensuring minimal loss of cell viability.

### LptD fluorescent labelling via ncAA incorporation followed by CuAAC

Sites for ncAA incorporation into LptD in *E. coli* BW25113 were selected based on in silico modelling of *E. coli* LptD crystal structure deposited in the PDB database [PDB: 4RHB] (Supplementary Fig. 1b). Four sites for propargyl-L-Lysine ncAA incorporation were selected based on criteria designed to minimise the impact of ncAA incorporation on LptD function and structure, whilst maximising the probability of ncAA incorporation at an accessible site to enable efficient downstream bio-orthogonal fluorophore coupling and minimise dynamic quenching of the coupled extrinsic probe by certain amino acids (e.g. tryptophan, histidine, methionine and tyrosine)^91^. We selected two lysine (K473 and K602) and two aspartic acid (D592 and D600) residues located on two unstructured, exposed extracellular loops of LptD. These selections were informed by results from published structural, computational and biochemical studies of LptD^24,92,93^. By avoiding the extracellular loops, residues and regions implicated in LptD function by these previous studies, we were able to minimise the probability that ncAA incorporation would have a deleterious impact on LptD structure and function.

In the absence of L-arabinose, basal araBAD promoter activity drove low-level expression of the recombinant amber-stop codon-containing *lptD* gene (lptD*); addition of propargyl-L-Lysine enabled amber suppression by the *pylRS* and tRNA^Pyl^ pair and incorporation of the ncAA into full-length LptD with the ncAA integrated at one of four positions in its extracellular surface loops (LptD*). Because LptD is essential, cells retain an endogenous chromosomal LptD pool; therefore, dSTORM localisations reported only the plasmid-expressed ncAA-containing LptD* subpopulation. The accessible alkyne groups in the surface loops of LptD* enabled conjugation of an azide(-N_3_)-functionalised, extrinsic fluorescent organic dye molecule via CuAAC^94,95^. LptD* fluorescent labelling enabled in vitro characterisation of the recombinant protein by fluorescence imaging of LptD-containing protein bands after SDS-PAGE (Supplementary Fig. 1c).

Direct LptD* fluorophore conjugation via CuAAC allowed us to carry out experiments without the use of immunoblotting for LptD and LptDE visualisation. Validation of ncAA-LptD production and subsequent fluorescent labelling was done for all four ncAA-containing *lptD* variants along with SDS-PAGE screens of recombinant protein production levels and ability to form a complex with LptE. Based on this screening process, the LptD^D600^ mutant was selected and used in all dual-labelling experiments for LptD* and newly inserted LPS followed by visualisation via two-colour dSTORM super resolution microscopy (Fig. 1 and Supplementary Fig. 1).

### OmpA fluorescent labelling via ncAA incorporation followed by CuAAC

To investigate the spatial distribution and organisation of LPS and OMPs relative to one another in the outer leaflet of the OM, OmpA was selected for labelling via adaptation of the strategy used for LptD having demonstrated in a previous study that OmpA and LPS are organised in discrete clusters in the OM of *E. coli* BW25113 cells^11^. OmpA is one of the most abundant OMPs in *E. coli* with approximately 100,000 molecules per cell^96,97^, and lateral confinement of OmpA in the OM does not depend on its peptidoglycan-binding domain^98^. A three-dimensional model of the *E. coli* OmpA structure was produced using AlphaFold2^99^. We then identified regions of the OmpA primary sequence predicted to form unstructured extracellular loops. We selected an accessible glutamic acid (E89) residue in extracellular loop 2 for propargyl-L-Lysine incorporation via mutation of the respective codon in the *ompA* gene sequence to the amber stop codon (TAG)^100–102^. After isolation and amplification of the *E. coli* BW25113 *ompA* gene sequence from the purified bacterial chromosomal DNA via PCR (Supplementary Table 2), we incorporated the wild-type *ompA* gene into a pBADcLIC expression vector. The target codon in the *ompA* gene was mutated to TAG via PCR mutagenesis (Supplementary Table 2). Under standard laboratory culture conditions, OmpA is a non-essential OMP. Therefore, we were able to co-transform the TAG-containing *ompA* expression vector into an *E. coli* ∆*ompA* strain (Supplementary Table 1) along with the pEVOL-pylRS vector^88^. Full-length ncAA-containing OmpA (OmpA*) production via leaky expression was initiated by charging cell cultures with 1 mM *N*-propargyl-L-Lysine-OH. OmpA* fluorescent labelling with a functionalised small extrinsic organic dye molecule was done in situ via CuAAC.

### Dual differential fluorescent labelling of LptD* and newly inserted Kdo-N_3_-containing LPS

*E. coli* BW25113 cells were co-transformed with pBADcLIC-lptD* and pEVOL-pylRS via electroporation as described above. A 5 mL volume of supplemented M9 CDM with 100 µg mL^−1^ ampicillin and 35 µg mL^−1^ chloramphenicol was inoculated with a single colony of freshly co-transformed cells and incubated at 37 °C with shaking (220 rpm) for approximately 7 h (to an OD_600_ of 1.5–2.0). This pre-culture was used to inoculate a fresh volume of supplemented M9 CDM plus selection antibiotics to a starting OD_600_ of 0.05. The culture was incubated as before for approximately 3 h, until it reached an OD_600_ of 0.5. Uninduced recombinant LptD^*^ production and LPS Kdo-N_3_ metabolic labelling were initiated simultaneously via the addition of propargyl-L-Lysine and Kdo-N_3_ to final concentrations of 1.0 mM and 4.0 mM, respectively. The recombinant LptD^*^ production/LPS metabolic labelling culture was then re-incubated under standard conditions for a further 30 min. The culture was then removed and placed immediately on ice to halt further growth and minimise further LPS insertion/LptD* production. The culture was diluted with pre-chilled, supplemented M9 CDM plus selection antibiotics to an OD_600_ of 1.0 and transferred to a sterile pre-chilled 2.0 mL microcentrifuge tube on ice.

Cells were then harvested via centrifugation (10,000 × *g*, 4 °C, 3 min) and the supernatant was removed. The cell pellet was then resuspended to an OD_600_ of 1.0 in fresh, pre-chilled supplemented M9 CDM plus selection antibiotics via repeated, gentle pipetting. The resuspended culture was transferred to a sterile pre-chilled 2.0 mL microcentrifuge tube on ice, and the cells were re-pelleted via centrifugation. This washing step was repeated three times in total to ensure removal of residual propargyl-L-Lysine and Kdo-N_3_. The washed cell pellet was then labelled by two sequential CuAAC reactions (one dye per step). In the first step, cells were resuspended to an OD_600_ of 1.0 in Click-iT mix charged with a single complementary fluorophore (either an azide(-N_3_) or alkyne-functionalised AF488/AF647 dye, matched to the relevant bio-orthogonal handle; Kdo-N_3_-labelled LPS was labelled with an alkyne-functionalised dye and LptD* (propargyl-L-Lysine) was labelled with an azide(-N_3_) functionalised dye), transferred to a 2 mL microcentrifuge tube, and incubated at room temperature for 30 min to ensure efficient CuAAC labelling on a rotary wheel (12 rpm, 80° incline relative to benchtop) protected from light. Cells were then pelleted and washed to remove unreacted dye before performing a second CuAAC reaction with the complementary dye to label the remaining handle. Cells were kept on ice in pre-chilled tubes/buffers except during the CuAAC incubations. In replicate experiments, the newly inserted LPS and LptD* labelling order was alternated, as were the dye combinations (Supplementary Table 6). This labelling strategy was implemented to confirm that the order in which species were labelled and the dye combinations used did not influence the apparent spatial distributions and relative concentrations of LptD* and newly inserted LPS (Fig. 1b and Supplementary Fig. 1a). Cells were pelleted via centrifugation (10,000 × *g*, 4 °C, 3 min), and the spent Click-iT mix supernatant was removed. The cell pellet was then resuspended to an OD_600_ of 1.0 in 0.2 µm-filtered, pre-chilled phosphate-buffered saline (PBS, 137 mM NaCl, 2.7 mM KCl, 4.3 mM Na_2_HPO_4_, Sigma-Aldrich) pH 7.4 via gentle repeated pipetting.

### Preparation of labelled cell samples for direct stochastic optical reconstruction microscopy (dSTORM)

Immediately after the Click-iT labelling step, the cell suspension was transferred to a fresh pre-chilled 2 mL microcentrifuge tube and pelleted via centrifugation (10,000 × *g*, 4 °C, 2 min,). Labelled cell pellets were re-suspended to an OD_600_ of 1.0 in 0.2 µm-filtered PBS pH 7.4 supplemented with 2 mM MgSO_4_ and 0.5 mM CaCl_2_, and transferred to a sterile, pre-chilled 2 mL microcentrifuge tube. Cells were re-pelleted via centrifugation. This washing step was carried out a total of three times. The washed and labelled cell pellet was fixed via resuspension to an OD_600_ of 1.5 in 4% (v/v) ultrapure, methanol-free paraformaldehyde (PFA, Polysciences Inc.) in PBS pH 7.4. This solution was incubated for 30 min on a rotary wheel (12 rpm, 80° incline relative to benchtop) at room temperature protected from light. The fixed cell suspension was then transferred to a sterile 1.5 mL microcentrifuge tube and pelleted via centrifugation. The pellet of fixed and labelled cells was resuspended to an OD_600_ of 1.0 in PBS pH 7.4 with 0.5 mM MgSO_4_ and 0.15 mM CaCl_2_, transferred to a fresh microcentrifuge tube and pelleted via centrifugation. This washing step was carried out three times in total.

### Mounting of fixed fluorescently labelled cell samples using poly-D-Lysine-coated coverslips for imaging by dSTORM

The washed, fixed, and labelled cell pellet in a microcentrifuge tube was placed on ice to cool to 4 °C and resuspended in degassed, pre-chilled glucose oxidase (GluOx) (Sigma Aldrich)/catalase (Cat) (Sigma-Aldrich) O_2_ scavenging buffer to an OD_600_ of 1.5 via repeated gentle pipetting (Supplementary Table 7)^103,104^. 45.5 µL of the suspension was transferred to a pre-chilled, sterile 500 µL microcentrifuge tube on ice. 2.5 µL of 1 M β-mercaptoethylamine hydrochloride (β-MEA) (50 mM final concentration), 2 µL of 0.5% (w/v) 5 µm diameter silica bead slurry (final bead concentration of 0.02% (w/v)) and 0.5 µL TetraSpeck^TM^ 0.2 µm microsphere standard solution (stock concentration ~1.5 × 10^9^ particles mL^−1^, final concentration ~ 1.5 × 10^7^ particles mL^−1^, Invitrogen Ltd.) were added. TetraSpeck^TM^ beads were added to enable accurate AF488 and AZ647 channel alignment during two-colour dSTORM image processing. 10 µL of the suspension was then pipetted onto the centre of a pre-chilled (to minimise O_2_ scavenging buffer enzyme activity), clean 1.0–1.2 mm thick glass slide. The slide was covered with a poly-D-lysine-coated, 18 mm × 18 mm high precision (Zeiss), no. 1.5 glass coverslip, ensuring no bubbles were present in the slide chamber. The chamber was sealed with clear nail varnish. The slide was then incubated at 4 °C for 20 min while protected from light to allow the nail varnish to dry and the cells to adhere to the poly-D-lysine-coated coverslip surface.

### Two-colour dSTORM data acquisition with AF488- and AF647-labelled samples

dSTORM imaging was done on a Zeiss Elyra 7 super-resolution imaging platform in laser widefield beam path mode using ZEN Black 3.0 SR FP2 software (Biosciences Technology Facility, University of York). Experiments were carried out using a Plan-Apochromat 63×/1.46 NA Korr oil immersion objective Var 2 lens together with TIRF uHP (ultra-high power) laser power density setting. Image areas were set to 128 pixels × 128 pixels for two cells or 64 pixels × 64 pixels for a single cell and saved in 16-bit format. Initial excitation and intersystem crossing of all fluorophores in the target cell(s) from their ground singlet state to an excited triplet dark state was achieved via application of a short (200–300 frames, 50 ms exposure time), high intensity laser pulse (488 nm: 20-25% laser power, 642 nm: 10–15% laser power) with the microscope in epifluorescence (EPI) mode. dSTORM time series image sequences were then collected using highly inclined and laminated optical sheet (HILO) illumination for 10,000 frames (50 ms exposure time) using reduced laser powers (488 nm: starting at 1% increasing to 10% over the time series, 642 nm: starting at 0.7% and increasing to 5% over the time series). To propagate fluorophore blinking in the latter 5000 frames, 405 nm light was introduced during the transfer phase of individual image collection to reduce fluorophore photobleaching resulting from simultaneous exposure to both 488 nm/642 nm and 405 nm light^104^. HILO illumination mode was used to enable excitation of fluorophores in the outer membrane furthest from the coverslip thereby maximising the signal to noise (S:N) ratio. Additional dSTORM image acquisition settings are summarised in Supplementary Table 8.

Two-colour dSTORM imaging of AF488- and AF647-labelled species in the OM of in-frame, in-focus cells was done sequentially using the Zeiss Elyra 7 super-resolution imaging platform with 488 nm and 642 nm excitation lasers. The BP420-480 + BP490-550 emission filter (Supplementary Table 8) was used to prevent cross-channel bleeding of fluorescence emission from the two dyes. dSTORM data for AF647-labelled species was collected first using the second sCMOS camera due to the lower photostability of the dye compared to AF488. dSTORM data for AF488-labelled species were then collected using the first sCMOS camera. After the initial high-intensity burst to trigger fluorophore intersystem crossing from ground singlet states to a dark triplet state, the fluorescent emission blinking events were collected over 10,000 frames using a 50 ms exposure for each channel. This two-colour acquisition workflow was also used to capture OMV-associated dual-colour examples comparing background versus newly inserted LPS (Fig. 6c and Supplementary Fig. 12d).

### Processing of dSTORM data

dSTORM raw data were processed using the SMLM (single-molecule localisation microscopy) processing facility found within the ZEN Black 3.0 SR software. Time series image sequences were converted to a crude single dSTORM image discarding overlapping molecules using a *x,y* Gauss fit model, with a peak mask size of 9 pixels and a peak intensity to noise ratio of 7.0. Typical filtering settings are described in Supplementary Table 9. SMLM-grouping settings were adjusted to minimise double counting of individual dye molecules (5 frames for the maximum ON time, 50 frames for maximum OFF gap, 1.7 pixel for capture radius). Pixel resolution was set at 10 nm pixel^−1^ with localised peaks displayed in Gauss mode reflecting the degree of emission localisation precision. dSTORM image data was converted into images (.czi format) using the ZEN 3.0 SR software. Processed dSTORM data for individual channels was exported (.csv table format) for further processing and analysis. Images were exported to FIJI/ImageJ for final image production. Representative final images were processed in FIJI/ImageJ and saved in both .TIFF and .PNG formats. For OMV imaging by dSTORM, vesicle-like protrusions and extracellular puncta consistent with OMVs could not be reliably identified during acquisition and were typically confirmed only after localisation and reconstruction. OMV-containing examples were therefore obtained by screening multiple cells/fields, and the number of analysed cells (*n*) and biological replicates (*N*) are reported in the legends for Fig. 6b,c and Supplementary Fig. 12c, d.

### Spatial assessment of LptD*/newly inserted LPS via cross-correlation analysis of two-colour dSTORM data outputs

To quantify the spatial relationship between newly inserted LPS and the essential outer membrane protein LptD, two-dimensional spatial cross-correlation analysis was performed on two-colour dSTORM localisation data (exported from ZEN Black 3.0 SR FP2 software in .csv table format) from the OM of individual *E. coli* cells using MATLAB R2024b (64-bit) software. Localisation coordinates for labelled LptD* and corresponding newly inserted LPS were first realigned using principal component analysis (PCA) to correct for rotational variance and align the long axis of the cell with the *x*-dimension, enabling geometrically consistent estimation of cell length and width. Cell area was approximated by multiplying these dimensions.

To extract biologically meaningful radial correlation profiles, symmetric square grid of pairwise spatial displacements between all LptD* (*reference*) and newly inserted LPS (*target*) localisations was constructed. Each pairwise distance vector was binned radially in increments defined by the localisation precision. The bin width was set to 1.5× the median localisation precision across both channels (rounded to the nearest 10 nm), to ensure biologically resolvable separation of localisations into discrete shells without over-fragmentation of signal. The maximum radial displacement range was defined as 50% of the larger cell dimension (typically the long axis), capturing pairwise interactions across the full cell body while avoiding spurious correlations driven by distant points beyond the OM envelope. A null reference distribution was computed by randomising the LPS coordinates within the same cell envelope boundary, maintaining point density and cell geometry while eliminating true co-localisation. The final *g*(*r*) values represent the ratio of observed LptD*—newly inserted LPS pairwise counts to those expected under this null model, at each radial distance.

Radial cross-correlation values were exported as *g*(*r*) profiles (Fig. 1c and Supplementary Fig. 1d,e) and further summarised using biologically interpretable parameters, including the radial distance at which the correlation drops to half-maximal (*r_half-max*) and the corresponding *g*(*r*) value (*half-max*), as measures of the strength and spatial extent of association between LptD and newly inserted LPS. All pairwise data and *g*(*r*) outputs were exported per cell for population-level summary construction and figure generation using OriginPro 2024b (64-bit) software. Two-colour dSTORM input files and analysis outputs are provided in Supplementary Fig. 10 and in the accompanying indexed Figure Source Data and Analysis Workbook. The custom MATLAB scripts used for these analyses, together with the associated README files, are available through the Persistent_LPS_insertion_OM_OMVs GitHub repository at https://github.com/joenabarro-OM/Persistent_LPS_insertion_OM_OMVs/releases/tag/v1.0.2 and have been permanently archived on Zenodo at https://doi.org/10.5281/zenodo.21326870^105^.

### LptD* and newly inserted LPS demograph generator from two-colour dSTORM coordinates

To visualise the spatial distribution of LptD and newly inserted LPS across individual *E. coli* cells (*n* = 30), demographs were generated from two-colour dSTORM localisation data using MATLAB R2024b (64-bit) software (Fig. 1d). Localisation co-ordinates for LptD* and newly inserted LPS channels exported from processed two-colour dSTORM data in .csv table formats were grouped by *Cell_ID* ( = 1–30) and rotated using PCA such that the long axis of each cell was aligned with the *x*-axis. This geometric standardisation ensured consistent longitudinal orientation. Coordinates were then binned along the x-axis using a fine bin width of 25 nm, chosen to balance spatial resolution with noise reduction based on typical bacterial cell length scales and localisation precision (10–40 nm). Each binned intensity profile was normalised to its own maximum, enabling relative comparison of spatial enrichment patterns between cells of varying brightness or size. Demographs were centre-aligned by aligning the mid-point of the localisation distribution for each cell to a common reference. A missing value (*NaN*) due to uneven cell lengths or edge effects was rendered black to distinguish absent data from low signal. This approach was used to highlight population-wide trends while preserving per-cell spatial heterogeneity. dSTORM input and analysis output data are provided in the accompanying indexed Figure Source Data and Analysis Workbook, MATLAB script and associated README file are available through the Persistent_LPS_insertion_OM_OMVs GitHub repository at https://github.com/joenabarro-OM/Persistent_LPS_insertion_OM_OMVs/releases/tag/v1.0.2 and have been permanently archived on Zenodo at https://doi.org/10.5281/zenodo.21326870^105^.

### Pearson correlation coefficient calculation from processed two-colour LptD^*^/newly inserted LPS dSTORM data

To quantify spatial correlation between LptD* and newly inserted LPS in the OM of individual *E. coli* cells, a photon-weighted Pearson correlation coefficient (PCC) was calculated. Two-colour dSTORM localisations were segmented by cell and assigned to either the LptD* or newly inserted LPS channel, according to the channel in which they were observed. Coordinates were mean-centred and aligned along the principal cell axis using PCA. Localisations were binned along the longitudinal axis in 25 nm intervals, and photon counts per bin were used to construct channel-specific intensity profiles. These were smoothed using a Gaussian kernel (*σ* = 1.5 bins) to reduce noise. Each cell was divided into three equal-length regions (two poles and mid-cell), and the PCC was calculated for each region and across the full cell length using MATLAB 2024b (64-bit). This approach enabled region-specific quantification of LptD–LPS co-localisation dynamics (Fig. 1e). The input data and analysis output source data are provided in the accompanying indexed Figure Source Data and Analysis Workbook, MATLAB scripts and associated README files are available through the Persistent_LPS_insertion_OM_OMVs GitHub repository at https://github.com/joenabarro-OM/Persistent_LPS_insertion_OM_OMVs/releases/tag/v1.0.2 and have been permanently archived on Zenodo at https://doi.org/10.5281/zenodo.21326870^105^.

### Monitoring time-dependent changes in background and newly inserted LPS distributions in the OM of *E. coli* BW25113 cells via Kdo-alkyne/Kdo-N3 pulse-chase experiments followed by two-colour dSTORM

A 5 mL volume of supplemented M9 CDM was inoculated with a single colony of *E. coli* BW25113 cells from a freshly streaked LB/agar plate. Post-inoculation, this pre-culture was incubated at 37 °C with shaking (220 rpm) for approximately 7–8 h. This pre-culture was used to inoculate a separate volume of supplemented M9 CDM charged with 4.0 mM Kdo-alkyne to a starting OD_600_ of 0.05. This background LPS metabolic labelling culture was then incubated for 14 h at 37 °C with shaking (220 rpm). The following day the culture containing cells with background, Kdo-alkyne-containing LPS in the OM was transferred to ice to halt growth and further insertion of LPS. The culture was diluted to an OD_600_ of 1.0 via addition of pre-chilled supplemented M9 CDM and transferred to a sterile, pre-chilled 2.0 mL microcentrifuge tube. Extra care was taken when handling and manipulating the culture to ensure minimal loss of viability resulting from processing steps. Cells were pelleted via centrifugation (8000 × *g*, 3 min, 4 °C). After careful supernatant aspiration the pellet was resuspended to an OD_600_ of 1.0 in pre-chilled supplemented M9 CDM via gentle pipetting and intermittent tube swirling (thereby minimising cell viability loss resulting from excessive shear stress). The suspension was transferred to a sterile, pre-chilled 2.0 mL microcentrifuge tube and cells re-pelleted via centrifugation. This washing step was repeated three times in total to ensure complete removal of residual Kdo-alkyne.

Cells were then resuspended in pre-warmed (to 37 °C) supplemented M9 CDM to an OD_600_ of 0.5 and transferred to a sterile 50 mL screw-cap polypropylene tube. An initial sample (time = 0 min) was removed immediately upon resuspension and transferred to a pre-chilled, sterile 1.5 mL microcentrifuge tube on ice. Immediately after removal of this sample, metabolic labelling of newly inserted LPS was initiated by adding Kdo-N_3_ to the remaining culture (final concentration 4.0 mM). Following this addition the culture was incubated as before and samples removed at 15 min intervals over the 2 h monitoring window. Cells were pelleted via centrifugation (10,000 × *g*, 3 min, 4 °C). Pellets were washed three times with supplemented M9 CDM. Washed pellets from each time point were labelled by two sequential CuAAC reactions (one dye per step). For the first step, cells were resuspended to an OD_600_ of 1.0 in Click-iT mix containing a single complementary fluorophore (either an alkyne-functionalised AF488/AF647 dye to label Kdo-N_3_ (newly inserted LPS), or an azide (-N_3_)-functionalised AF488/AF647 dye to label Kdo-alkyne (background LPS)), transferred to a 2 mL microcentrifuge tube, and incubated at room temperature for 30 min on a rotary wheel (12 rpm, 80° incline) protected from light. In replicate experiments, labelling order and dye orientation were alternated (Supplementary Table 12). This alternative labelling strategy was implemented in the experimental replicates to ensure that the order in which species were labelled and dye combination used did not influence the spatial distributions and relative concentrations of background and newly inserted LPS (Fig. 2b,c).

Cells were then pelleted, washed to remove unreacted dye, and resuspended to an OD_600_ of 1.0 in Click-iT mix containing the second (complementary) fluorophore to label the remaining handle, followed by incubation at room temperature for 30 min on a rotary wheel protected from light. Cells were then pelleted via centrifugation (10,000 x g, 3 min, 4 °C). The spent Click-iT mix was carefully removed, and the pellet resuspended in pre-chilled, filtered PBS pH 7.4 to an OD_600_ of 1.0. The suspension was transferred to a sterile, pre-chilled 1.5 mL microcentrifuge tube and cells re-pelleted via centrifugation (10,000 × *g*, 3 min, 4 °C). This washing step was repeated three times in total. Cells were then fixed by re-suspension to an OD_600_ of 1.0 in 4% (v/v) paraformaldehyde in PBS pH 7.4 followed by incubation at room temperature for 30 min on a rotary wheel (12 rpm, 80° incline relative to benchtop) protected from light. The suspension was then transferred to a sterile, pre-chilled 1.5 mL microcentrifuge tube and cells were pelleted via centrifugation (10,000 x g, 3 min, 4 °C). The pellet consisting of dual-labelled, fixed cells was washed three more times in pre-chilled, 0.2 µm-filtered PBS pH 7.4 as before. After the final wash step, the cell pellets were resuspended to an OD_600_ of 1.0 in PBS pH 7.4 and stored at 4 °C or on ice, protected from light until imaging was completed.

### DBSCAN cluster segmentation and cluster-area intersection analysis of two-colour dSTORM localisation data

Two-colour dSTORM localisation tables (background LPS and newly inserted LPS) were exported from ZEN Black v3.3 (Carl Zeiss Microscopy) as .csv files and analysed in Python 3.10.12 (NumPy 1.26.0, pandas 2.2.1, shapely 2.0.3, scikit-learn 1.5.0). Both labelled newly inserted and background LPS localisations for each individual *E. coli* cell were merged, and a convex hull was expanded by 20 nm to define the OM outline. The polygon was simplified (2 nm tolerance) to remove sub-pixel kinks. PCA rotated each cell, so its long axis lay horizontally, ensuring a common reference frame throughout the 120 min pulse–chase.

Spatial domains of background, (pre-existing) and newly inserted LPS were identified independently using a tailored Density-Based Spatial Clustering of Applications with Noise (DBSCAN) algorithm (ε = 30 nm, *min_samples* = 7)^106^, widely adopted for co-localisation analysis of multi-channel SMLM data^107^. The ε value equals 1.5× the median nearest-neighbour distance between LPS localisations under our imaging conditions (median = 22 nm) and reflects the average precision associated with individual localisations ( ~ 30 nm), balancing sensitivity to genuine nanoscale domains with robustness to photon shot noise. A *min_samples* threshold of seven exceeds the upper quartile localisation count for single fluorophore bursts, limiting false positive clusters. Cluster polygons were clipped to the OM boundary and combined to obtain total old and new LPS cluster areas; their geometric intersection (*background LPS* ∩ *newly inserted LPS*) defined the cluster-area intersection (mask-intersection) region. Because ε is set on the order of the effective localisation precision, this mask-intersection metric reports cluster-mask co-occurrence/proximity at the analysis scale, rather than molecular co-occupancy. The script produces several output files. LPS_output_summary.csv contains numerical values for the OM area, individual and combined LPS areas, localisation counts within the mask-intersection region, and normalised coverage metrics; these underpin the quantitative time course displayed in Fig. 2e,f and are reproduced in Supplementary Table 13. A high resolution composite image (combined_overlap_map.png) was produced showing all localisation points, their DBSCAN assigned clusters and the spatial overlap; the map provides morphological context for the representative cells provided in Supplementary Fig. 2e (note that overlap in these outputs refers to geometric overlap of independently segmented cluster masks at the analysis scale, not physical co-occupancy of LPS molecules). Cluster size distributions are exported as separate files (.csv format), enabling independent verification that old and new clusters are sampled from comparable size ranges.

Examples of individual channel input dSTORM data for each time point, individual cell OM output data files and summary data output tables are provided in the Figure Source Data and Analysis Workbook. Python script and associated README file are available through the Persistent_LPS_insertion_OM_OMVs GitHub repository at https://github.com/joenabarro-OM/Persistent_LPS_insertion_OM_OMVs/releases/tag/v1.0.2 and have been permanently archived on Zenodo at https://doi.org/10.5281/zenodo.21326870^105^.

#### LPS extraction

LPS was extracted via adaption of the hot phenol – aqueous method developed by Westphal (1965)^108^. The cell pellet was re-suspended to an OD_600_ of 0.5 in PBS pH 7.4 plus 0.5 mM MgCl_2_ and 0.15 mM CaCl_2_ and pelleted via centrifugation (10,000 x g, 10 min, 4 °C). The washed cell pellets were then resuspended to the same OD_600_ in ultrapure deionised water (resistivity = 18.2 MΩ cm) and transferred to a glass dram with Teflon-coated lid. An equal volume of 90% (w/v) phenol, pre-warmed to 65 °C was added and the emulsion stirred vigorously at 65 °C for 15 min. Drams were then transferred to ice for 15 min to cool. Emulsions were then transferred to microcentrifuge tubes and centrifuged (8,500 x g, 15 °C 10 min). The LPS-containing aqueous fraction was transferred to a 50 mL screw-cap polypropylene tube. A second aqueous extraction was then carried out on the remaining phenol layer and the aqueous fractions were pooled in a 50 mL screw-cap polypropylene tube. Sodium acetate was added to the pooled aqueous fractions to a final concentration of 0.5 M to ensure LPS precipitation. 10 volumes of 95% (v/v) ethanol, pre-chilled to -20 °C was then added and after mixing via repeated inversions the suspension was incubated overnight at -20 °C to maximise LPS precipitation. The following day LPS was pelleted via centrifugation (2,000 x g, 4 °C, 10 min,). After supernatant aspiration, the LPS pellet was dried under a steady stream of nitrogen gas. The dried LPS pellet was resuspended in 100 µL ultrapure deionised water and transferred to a 1.5 mL microcentrifuge tube. Sodium acetate was added to a final concentration of 0.5 M and 1.1 mL pre-chilled 95% (v/v) ethanol was added. After mixing the tube contents via repeated inversion, the LPS was re-precipitated via overnight incubation at -20 °C. Precipitated LPS was pelleted via centrifugation. The pellet was dried again under nitrogen gas and finally resuspended in 50 µL PBS pH 7.4.

### LPS tricine-sodium dodecyl sulfate-polyacrylamide gel electrophoresis (TSDS-PAGE)

A volume (15 µL) of the purified LPS was mixed with a volume (5 µL) of 4x loading buffer (Supplementary Table 14) plus 2% (v/v) β-mercaptoethanol (β-ME). After vortex mixing and a brief centrifugation, the samples were incubated for 1 h at 40 °C. The samples were then re-vortexed and centrifuged briefly before loading the entire sample volume into the well of a 10 cm×10 cm x 1 mm 15% (w/v) acrylamide resolving/6% (w/v) acrylamide stacking Tricine-SDS gel prepared according to the protocol published by Schägger (2006)^109^.

Commercial smooth LPS standards (Thermo Scientific^®^) were analysed alongside extracted LPS samples to facilitate classification of sample LPS bands. O55:B5-AF488 LPS conjugate samples were prepared for TSDS-PAGE by adding 1 µL of 1 mg mL^-1^ LPS conjugate stock solution to 9 µL ultrapure deionised water followed by 3.3 µL of 4 x loading buffer plus 2% (v/v) β-ME (Supplementary Table 14)*.* Samples were vortexed and briefly centrifuged before being incubated at 40 °C for 1 h, then re-vortexed and briefly centrifuged again before loading the entire sample volume into the well of a Tricine gel. Electrophoresis was done at 4 °C using pre-chilled buffers with the gel tank protected from light to prevent fluorophore (AF488) photobleaching. Electrophoresis was done in constant current mode for 45 min at 30 mA until a dye front formed as a fine horizontal line at the stacking / resolving gel interface. The current was then increased to 60 mA for approximately 3 h until the dye front was approximately 1 cm from the base of the gel.

### Processing and visualisation of LPS TSDS-PAGE gels

Upon completion of electrophoresis, the gels were fixed via immersion in 150 mL 50% (v/v) methanol / 3% (v/v) glacial acetic acid and incubated overnight at room temperature on a slow-moving rocker, protected from light. The following day gels were washed via immersion in 150 mL 3% (v/v) glacial acetic acid and incubated for 20 min at room temperature on a slow-moving rocker. This step was repeated three times in total. AF488-LPS bands were then visualised using a Typhoon 5 bioimaging system (Amersham) equipped with a 488 nm argon laser and 525 BP filter. To visualise total LPS bands, gels were stained using a Pro-Q^TM^ Emerald 300 LPS gel stain kit according to the manufacturer’s instructions (Thermofisher Scientific^®^). Briefly, after fixation and washing steps, LPS carbohydrates were oxidised via immersion in 30 mL 1% (w/v) H_5_IO_6_ followed by incubation for 45 min on a slow-moving rocker at room temperature. Gels were washed three times in 150 mL 3% (v/v) glacial acetic acid for 20 min. Gels were then stained via immersion in 25 mL dilute (1:50) Pro-Q Emerald 300 stain and incubated for 90 min at room temperature on a slow-moving rocker. After post-staining washing steps, Pro-Q stained LPS bands were visualised under UV illumination using a GeneGenius gel imaging system.

### Quantifying relative rates of background LPS turnover on a cell-by-cell basis via Kdo-N_3_ / native Kdo pulse-chase experiments

A 5 mL volume of supplemented M9 CDM was inoculated with a single colony of the *E. coli* BW25113 strain picked from a freshly streaked LB / agar plate. Post-inoculation the culture was incubated at 37 °C with shaking (220 rpm) for approximately 7 hours. This pre-culture was used to inoculate a fresh volume of supplemented M9 CDM containing 4.0 mM Kdo-N_3_ to an OD_600_ of 0.05. This background LPS Kdo-N_3_ metabolic labelling culture was then incubated for 12 hours at 37 °C with shaking (220 rpm). The culture was then transferred to ice and diluted with pre-chilled M9 CDM without glucose or casamino acids (Supplementary Table 3) to an OD_600_ of 1.0 and cells were pelleted via centrifugation (8,000 x g, 3 min, 4 °C). After careful supernatant aspiration, the pellet was resuspended to an OD_600_ of 1.0 in pre-chilled supplemented M9 CDM via gentle pipetting and intermittent tube swirling (thereby minimising cell viability loss resulting from excessive shear stress). The suspension was transferred to a sterile, pre-chilled 2.0 mL microcentrifuge tube and cells re-pelleted via centrifugation. This washing step was repeated three times in total to ensure complete removal of all residual Kdo-N_3_.

The washed pellet of cells with Kdo-N_3_-labelled, background LPS in the OM were resuspended to an OD_600_ of 0.5 in pre-warmed supplemented M9 CDM charged with 4 mM native Kdo and incubated under standard conditions. Samples were taken at 30 min intervals over a 3.5 h period and OD_600_ measurements were done at time of sampling in triplicate to ensure accuracy and reliability. Samples were transferred to pre-chilled 2.0 mL sterile microcentrifuge tubes on ice and diluted with pre-chilled M9 CDM without glucose and casamino acids to ensure cell growth, division and LPS insertion were arrested. Cells were then pelleted via centrifugation (10,000 x g, 3 min, 4 °C). After careful supernatant aspiration, the pellets were resuspended to an OD_600_ of 1.0 in pre-chilled M9 CDM without glucose and casamino acids on ice. Suspensions were transferred to fresh sterile 2.0 mL microcentrifuge tubes and cells re-pelleted via centrifugation. This washing step was repeated three times in total.

Washed pellets of *E. coli* BW25113 cells containing background, pre-existing Kdo-N_3_-LPS in the OM were resuspended to an OD_600_ of 1.0 in Click-IT mix charged with AF488-alkyne. Suspensions were transferred to sterile 2 mL microcentrifuge tubes and incubated at room temperature on a slow-moving rotary wheel for 30 min (12 rpm, 80 ° incline relative to benchtop) protected from light. Following this labelling step, the suspensions were transferred to sterile 1.5 mL microcentrifuge tubes and cells pelleted via centrifugation (10,000 x g, 3 min, 4 °C). After aspiration of the spent Click-iT mix supernatants, the labelled cell pellets were resuspended to an OD_600_ of 1.0 in pre-chilled, 0.2 µm-filtered PBS pH 7.4. Suspensions were transferred to fresh, sterile 1.5 mL microcentrifuge tubes and the cells were re-pelleted via centrifugation. This washing step was repeated three times in total. Cells were then fixed via re-suspension to an OD_600_ of 1.0 in 4% (v/v) paraformaldehyde in PBS pH 7.4 followed by incubation at room temperature for 30 min on a rotary wheel (12 rpm, 80° incline relative to benchtop) protected from light. The suspension was then transferred to a fresh, pre-chilled 1.5 mL microcentrifuge tube and cells were pelleted via centrifugation (10,000 x g, 3 min, 4 °C). The pellet consisting of dual-labelled, fixed cells was washed three more times in pre-chilled, 0.2 µm-filtered PBS pH 7.4 as before. After the final wash step, the cell pellets were resuspended to an OD_600_ of 1.0 in PBS and stored at 4 °C or on ice, protected from light until imaging was completed.

### Quantifying relative rates of newly inserted LPS insertion on a cell-by-cell basis via native Kdo / Kdo-N_3_ pulse-chase experiments

A 5 mL volume of supplemented M9 CDM was inoculated with a single colony of *E. coli* BW25113 cells picked from a freshly streaked LB / agar plate. Post-inoculation the culture was incubated at 37 °C with shaking (220 rpm) for approximately 7 hours. This pre-culture was used to inoculate a fresh volume of supplemented M9 CDM containing 4.0 mM native Kdo to an OD_600_ of 0.05. This culture was then incubated for 12 hours. The culture was then transferred to ice and dilute with pre-chilled supplemented M9 CDM without glucose or casamino acids to an OD_600_ of 1.0 and cells were pelleted via centrifugation (8,000 x g, 3 min, 4 °C). After careful supernatant aspiration, the pellet was resuspended to an OD_600_ of 1.0 in pre-chilled supplemented M9 CDM via gentle pipetting and intermittent tube swirling (thereby minimising cell viability loss resulting from excessive shear stress). The suspension was transferred to a sterile, pre-chilled 2.0 mL microcentrifuge tube and cells re-pelleted via centrifugation. This washing step was repeated three times in total to ensure complete removal of all residual native Kdo. The washed pellet of cells was resuspended to an OD_600_ of 0.5 in pre-warmed supplemented M9 CDM charged with 4 mM Kdo-N_3_ and incubated under standard conditions.

Samples were taken at 30 min intervals over a 3.5 h period and OD_600_ measurements were done at the time of sampling in triplicate to ensure accuracy and reliability. After removal, the samples were transferred to sterile pre-chilled 2.0 mL microcentrifuge tubes on ice and diluted with pre-chilled M9 CDM without glucose and casamino acids. Cells were then pelleted via centrifugation (10,000 x g, 3 min, 4 °C). After careful supernatant aspiration, the pellets were resuspended to an OD_600_ of 1.0 in pre-chilled M9 CDM without glucose and casamino acids. Suspensions were transferred to fresh 2.0 mL sterile microcentrifuge tubes and cells re-pelleted via centrifugation. This washing step was repeated three times in total.

Washed pellets of *E. coli* BW25113 cells containing newly inserted Kdo-N_3_-labelled LPS in the OM were resuspended to an OD_600_ of 1.0 in Click-IT mix charged with AF488-alkyne. Suspensions were transferred to sterile 2 mL microcentrifuge tubes and incubated at room temperature on a slow-moving rotary wheel for 30 min (12 rpm, 80 ° incline relative to benchtop) protected from light. Following this labelling step, the suspensions were transferred to sterile 1.5 mL microcentrifuge tubes and cells pelleted via centrifugation (10,000 x g, 3 min, 4 °C). After aspiration of the spent Click-iT mix supernatants, the labelled cell pellets were resuspended to an OD_600_ of 1.0 in pre-chilled, 0.2 µm-filtered PBS pH 7.4. Suspensions were transferred to fresh, sterile 1.5 mL microcentrifuge tubes and the cells were re-pelleted via centrifugation. This washing step was repeated three times in total. Cells were then fixed via re-suspension to an OD_600_ of 1.0 in 4% (v/v) paraformaldehyde in PBS pH 7.4 followed by incubation at room temperature for 30 min on a rotary wheel (12 rpm, 80° incline relative to benchtop) protected from light. The suspension was then transferred to a fresh, pre-chilled 1.5 mL microcentrifuge tube and cells were pelleted via centrifugation (10,000 x g, 3 min, 4 °C). The pellet consisting of dual-labelled, fixed cells was washed three more times in pre-chilled, 0.2 µm-filtered 1 x PBS pH 7.4 as before. After the final wash step, the cell pellets were resuspended to an OD_600_ of 1.0 in PBS and stored at 4 °C or on ice, protected from light until imaging was completed.

### Single-colour dSTORM imaging

dSTORM imaging was done on a Zeiss Elyra 7 microscope in the laser widefield beam path mode using ZEN Black 3.0 SR FP2 software (Biosciences Technology Facility, University of York). Experiments were carried out using a Plan-Apochromat 63 x / 1.46 NA Korr oil immersion objective Var 2 lens together with TIRF uHP (ultra-high power) laser power density setting. Image areas were set to 128 pixels x 128 pixels for two cells or 64 pixels x 64 pixels for a single cell and saved in 16-bit format. Initial excitation and intersystem crossing of all fluorophores in the target cell(s) from their ground singlet state to an excited triplet dark state was achieved via application of a short (200 – 300 frames, 50 ms exposure time), high intensity 488 nm laser pulse (15% laser power) with the microscope in epifluorescence (EPI) mode. dSTORM time series image sequences were then collected using highly inclined and laminated optical sheet (HILO) illumination for 5,000 frames (50 ms exposure time) in total. For the first 1000 frames a laser power of 1.5% was applied, this was increased to 2.5% for frames 1000 – 2000, 3.5% for frames 2000 – 3000, 4.5% for frames 3000 – 4000 and finally to 5.5% for frames 4000 - 5000. To propagate fluorophore blinking in the latter 3000 frames, 405 nm light was introduced during the transfer phase of individual image collection to reduce fluorophore photobleaching resulting from simultaneous exposure to both 488 nm and 405 nm light^104^. HILO illumination mode was used to enable excitation of fluorophores in the outer membrane furthest from the coverslip thereby maximising the S:N ratio. Additional dSTORM image acquisition settings are summarised in Supplementary Table 8.

### Single-colour dSTORM data processing and measurement of discrete AF488 emission abundance in the outer membrane of *E. coli* BW25113

To ensure the compatibility of data derived from different cells, all dSTORM images were processed using a standard protocol with identical filter settings. dSTORM raw data were processed using the SMLM processing facility found within the ZEN Black 3.0 SR software. Time series image sequences were converted to a crude single dSTORM image discarding overlapping molecules using a *x,y* Gauss fit model, with a peak mask size of 9 pixels and a peak intensity to noise ratio of 6.5. Standard filtering settings used for all images are described in Supplementary Table 15. SMLM grouping settings were adjusted to minimise double counting of individual dye molecules (5 frames for the maximum ON time, 50 frames for maximum OFF gap, 1.7 pixel for capture radius). Pixel resolution was set at 9 nm pixel^-1^ with localised peaks displayed in Gauss mode reflecting the degree of localisation precision. The number of filtered spots was recorded and used to calculate AF488 emission abundance per µm^2^ on the OM surface after measurement of the OM surface area in ImageJ.

### Fluorescence recovery after photobleaching (FRAP) and confocal fluorescence microscopy

Fluorescence confocal microscopy analyses and FRAP experiments were conducted using either an upright Zeiss LSM710 confocal fluorescence microscope or an inverted Zeiss LSM 780 multiphoton confocal fluorescence microscope (Biosciences Technology Facility, University of York). Confocal imaging and FRAP quantitative and statistical analyses of FRAP data were done according to protocols used in a previous study^11^.

### DBSCAN analysis of single-colour dSTORM data using half-rod surface projection in LPS pulse–chase experiments

To quantify spatial features of newly inserted and background LPS-rich regions in the OM of individual *E. coli*, we developed a MATLAB pipeline that performed DBSCAN-based clustering on single-colour dSTORM datasets from pulse-chase experiments projected onto a three-dimensional (3D) half-rod geometry. Raw localisations were first pre-processed to remove missing values and aligned along the long axis of each cell using PCA. Data was then filtered to match a canonical *E. coli* rod geometry comprising a central cylindrical body and hemispherical polar caps. To account for projection distortion due to curvature of the bacterial surface, localisations were radially mapped onto a 3D half-rod surface defined by the visible hemisphere and top half of the cylindrical outer membrane. This strategy improved the geometric accuracy in density calculations, particularly at the poles. Unlike commonly used 2D clustering approaches, this method embeds localisation data into a biologically realistic 3D surface model, enabling more accurate quantification of cluster density and position across the full cell envelope.

Clustering was performed using DBSCAN parameters (ε = 30 nm, *minPts* = 7) chosen to match those used previously for two-colour overlap analyses, and to reflect the spatial resolution and labelling density typical of our dSTORM datasets. For each identified cluster, 2D convex hulls were computed in the aligned rod frame to calculate area and per-cluster localisation density (points per nm²). A z-height threshold was used to exclude clusters located near the edge of the projected rod surface (z < 20% of cell radius), which are more susceptible to projection artefacts. Global cluster density was calculated as the total number of clusters divided by the estimated surface area of the half-rod model, accounting for both cylindrical and hemispherical components. The pipeline was designed to be both modular and robust, with adjustable geometric and clustering parameters. It was designed for reproducible batch processing of individual cell datasets. The outputs of the pipeline were used to quantify temporal trends in LPS insertion and loss at both whole-cell and subcellular levels over the experimental time course, and summary output tables (Supplementary Tables 18 and 19) are provided in the Figure Source and Analysis Workbook. MATLAB scripts and associated README files are available through the Persistent_LPS_insertion_OM_OMVs GitHub repository at https://github.com/joenabarro-OM/Persistent_LPS_insertion_OM_OMVs/releases/tag/v1.0.2 and have been permanently archived on Zenodo at https://doi.org/10.5281/zenodo.21326870^105^.

### Region-resolved visualisation of LPS clusters across OM of single cells

To investigate whether cluster properties for newly inserted or background LPS varied between distinct spatial domains in the OM of *E. coli* cells, we implemented a MATLAB script to generate globally scaled, region-coloured cluster maps for individual *E. coli* cells. DBSCAN-derived cluster centroids identified in the previous analyses were classified as mid-cell or polar based on their *x*-coordinate relative to the cell midpoint (with poles defined as ±1.5× the cell radius). Cluster marker sizes were scaled globally by the square root of cluster area, enabling direct visual comparison across cells and timepoints. This visualisation allowed interrogation of spatial patterning in both background LPS and newly inserted LPS clusters following Kdo-N_3_ / native Kdo pulse–chase labelling. By applying a consistent classification scheme across all timepoints, the script enabled robust per-cell assessment of whether cluster distribution or size varied systematically between mid-cell and polar regions, and whether this organisation changed over time. The resulting maps provided an essential complement to global metrics, clarifying that distinct regional distributions of LPS clusters were maintained over time despite OM turnover. The MATLAB script and associated README files are available through the Persistent_LPS_insertion_OM_OMVs GitHub repository at https://github.com/joenabarro-OM/Persistent_LPS_insertion_OM_OMVs/releases/tag/v1.0.2 and have been permanently archived on Zenodo at https://doi.org/10.5281/zenodo.21326870^105^.

### Quantification of LPS enrichment in OMV nucleation regions from reconstructed localisation maps

Dual-colour dSTORM localisations for background (old) and newly inserted (new) LPS were obtained as described above. To control for fluorophore-dependent detection bias, dye assignments (AF488 vs AF647) for the old and new LPS pools were reversed across independent biological replicates (dye-swap), and the same enrichment analysis was applied. Individual cells displaying a clear vesicle-like protrusion were identified in reconstructed localisation maps. For each blebbing event, two regions of interest were defined: (i) an OMV bleb region of interest (ROI) encompassing the protrusion and (ii) an intact-cell outer membrane ROI encompassing the remaining cell outline while excluding the OMV bleb ROI. ROI areas were measured in µm² on 2D reconstructed localisation maps in ZEN Black 3.0 SR and reported in µm^2^ (Supplementary Fig. 13a,b). A total of *n* = 30 cells were analysed across the biological replicates (*N* = 3).

For each channel (c ϵ {*old LPS*, *new LPS*}), the localisation densities were calculated as follows:

1 $${\rho }_{c,{OMV}}=\frac{{N}_{c,{OMV}}}{{A}_{{OMV}}}$$

2 $${\rho }_{c,{cell}}=\frac{{N}_{c,{cell}}}{{A}_{{cell}}}$$

where *ρ*_*c,OMV*_ is localisation density in the OMV bleb ROI (for channel, *c*), *ρ*_*c,cell*_ is localisation density in the intact cell ROI (for channel, *c*), *N*_*c,OMV*_ is number of localisations in the OMV bleb ROI (for channel, *c*), *N*_*c,cell*_ is number of localisations in the intact cell ROI (for channel, *c*), *A*_*OMV*_ is OMV bleb ROI area (µm^2^), and *A*_*cell*_ is intact cell ROI area (µm^2^).

Individual cell / OMV bleb data are reported in Supplementary Table 24 and original dSTORM SMLM tables are provided in the Figure Source Data and Analysis Workbook. An enrichment score for each channel (*S*_*c*_) was calculated per protrusion ROI versus intact cell ROI as follows:

3 $${S}_{c}=\frac{{\rho }_{c,{OMV}}}{{\rho }_{c,{cell}}}$$

and reported as log_2_(*S*_*c*_), where log_2_(*S*_*c*_) = 0 indicates no enrichment in the OMV protrusion ROI, positive values indicate enrichment in the OMV protrusion ROI and negative values indicate depletion. For display purposes, per-cell log_2_(*S*_*c*_) values were rank ordered by log_2_(*S*_*old LPS*_) (descending) and plotted for individual cells (Supplementary Fig. 13c) with box plots (median and interquartile range, Supplementary Fig. 13d) using OriginPro 2024b (64-bit) software. Statistical comparisons used paired non-parametric tests on log_2_(*S*_*c*_) values: (i) two-sided Wilcoxon signed-rank tests against 0 for old and new LPS separately and (ii) a paired comparison of log_2_(*S*_*old LPS*_) versus log_2_(*S*_*new LPS*_) within the same cell. Localisation counts are interpreted as a proportional readout obtained under matched imaging and SMLM analysis settings, and therefore report relative partitioning between ROIs rather than absolute densities.

### Preparation of unsupplemented M9 pH 7.2 / agarose pads

The optimised protocol was based on a method published previously^110^. Slides were cleaned without exposure to any type of detergent. Briefly, slides were washed twice in acetone, washed twice in propan-2-ol and rinsed for 10 minutes under slow flowing deionised water. They were then placed in a drying oven, protected from dust overnight. The following day a 125 µL gene frame (Thermo Fisher Scientific) was placed flat at the centre of the slide and left overnight to set.

The following day the slide plus adhered gene frame was placed in a drying oven and heated to approximately 40 °C. 1 mL of sterile 2% (w/v) ultrapure low melting point (LMP) agarose (Thermo Fisher Scientific) in unsupplemented M9 CDM was prepared in a 50 mL screw-cap polypropylene tube via repeated, short duration, low power heating in a microwave (five10 – 15 s heating cycles at 5% power). Once the agarose had dissolved the tube was transferred to an ultrasonic water bath pre-heated to 50 °C and incubated for ~ 5 minutes to degas. 150 µL of the degassed, molten 2% (w/v) agarose / M9 solution was pipetted into the gene frame cavity in a laminar flow hood and covered with a clean glass slide. A weight was then placed on the top slide and the agarose / M9 pad was left to set for 60 min. After 60 min, the weight was removed and the agarose / M9 pad sealed in Parafilm^®^ and stored at 4 °C until required.

### Mounting of live *E. coli* BW25113 cells with fluorescently labelled background LPS for time-lapse 3D-SIM^2^ imaging

Post SPAAC labelling, the cells were resuspended to an OD_600_ of 2.0 in pre-warmed (to 37 °C) supplemented M9 CDM and incubated for 5 – 10 min at 37 °C. The agarose / M9 pad was incubated at 37 °C for 30 min after which a 1.5 cm×1.5 cm slice was cut using an ethanol cleaned scalpel and placed flat at the centre of a cleaned, pre-warmed glass slide. 10 µL of the labelled cell suspension was pipetted onto the centre of the pad and left for one minute in a laminar flow hood for the cells to adsorb. An additional 5 µL pre-warmed, supplemented M9 was deposited around the edges of the pad. A pre-warmed, cleaned (without detergent) glass, high-precision 18 mm×18 mm no. 1.5 glass coverslip (Zeiss) was then gently placed over the pad. The slide was sealed with a molten VALAP (1: 1: 1 mixture of Vaseline, lanolin, and paraffin) bead to prevent agarose pad desiccation during time course imaging^111^.

### Time-lapse 3D-SIM^2^ imaging of live *E. coli* BW25113 cells with fluorescently labelled background LPS

Slides were mounted onto a Zeiss Elyra 7 microscope in lattice SIM mode with a fitted incubator that had been prewarmed to 37 °C (for at least 12 h prior to the experiment to allow all components to equilibrate fully to the elevated temperature). Individual image acquisition settings are summarised in Supplementary Table 20. *Z*-stack images were collected at 2 to 5 minute intervals using low laser powers (0.5 – 1.0%) to minimise AF488 dye photobleaching thus extending the duration of time-lapse experiments whilst care was taken not to compress agarose pads by over-extending *z*-stack depth. *z*-stacks were collected over the largest possible cross-sectional areas (2560 pixels x 2560 pixels / 80.14 µm x 80.14 µm) to maximise data collection since increasing the field of view did not affect *xy* resolution (0.03 µm pixel^-1^). Individual experiments were terminated once the signal intensity:noise ratio for the AF488 fluorescence signal was deemed inadequate for reliable analysis (*i.e*. at the point where fluorescence signal in the OM region could not be reliably assigned to fluorescently labelled LPS rather than resulting from background noise). The duration of each experiment was usually 25 – 45 minutes depending on initial labelling efficiency and AF488-LPS signal strength.

For the reduced-photobleaching live-cell time-course experiments shown in Supplementary Fig. 19, the acquisition settings were modified to extend the imaging window while retaining sufficient OM-associated AF488-LPS fluorescence signal for the subsequent image processing. The number of SIM phases was reduced from 13 to 9, and the 488 nm excitation laser power was decreased relative to the standard live-cell 3D-SIM^2^ acquisition settings. Under these conditions, fluorescently labelled background LPS could be monitored for up to 120 min. SIM images were acquired at the time intervals indicated in Supplementary Fig. 19.

During live time-course imaging of stationary-phase cells presenting fluorescently labelled background Kdo-N_3_-LPS, we repeatedly observed discrete, intensely fluorescent extracellular puncta/OMV-like structures adjacent to intact cells, consistent with OMV-mediated preferential background-LPS clearance (Supplementary Fig. 12a,b). Imaging was performed across N ≥ 3 biological replicates with n ≥ 30 cells imaged per replicate. Because many of the OMV dimensions fall below the effective diffraction-limited detection threshold in live-cell fluorescence imaging, we additionally provide a conservative, resolvable OMV: cell ratio from a representative wide-field field-of-view (14 OMV structures adjacent to 72 intact cells; a ratio of 0.194 OMVs per cell; Supplementary Fig. 12b).

### 3D-SIM^2^ z-stack image processing and re-construction

3D SIM z-stack images were processed using the SIM^2^ programme within ZEN Black 3.0 SR software using default settings to minimise loss of detail during image processing. Final 2D depth coded images were produced in ZEN 3.1 (blue edition) in .CZI (for further processing) and .PNG (for presentation) formats. 2D image cross-sections (lower cell surface, mid-cell and upper cell surface) were produced in FIJI / ImageJ (version 1.54 h, with BioFormats plug-in installed) by summing the two slices from the 3D-SIM^2^ z-stacks that corresponded to each of these image cross-sections. 3D depth coded images were produced in Zen Blue 3.1 software, and were saved in both .TIFF and .PNG formats. As a control, to demonstrate standard rates of cell elongation and division, the lengths of randomly selected cells were measured at each time point and their normalised cell length (to the maximum observed length for each cell) were plotted against time (Supplementary Fig. 5d).

### Biochemical fractionation to assess cytoplasmic internalisation of fluorescently labelled background LPS

To verify that fluorescently labelled background LPS does not undergo intracellular internalisation and cytoplasmic recycling during the microscopy time course, *E. coli* BW25113 LPS was metabolically labelled with Kdo-N_3_ and fluorescently labelled in vivo with DBCO-AF488 via SPAAC using the same labelling and washing workflow used to prepare cells for live-cell imaging (Fig. 5, Supplementary Figs. 5 and 19, Supplementary Table 5). The starting time point (t = 0) was defined as the point after completion of AF488-labelling and removal of free DBCO-AF488 via repeated washing steps with pre-chilled M9 CDM as described above for live-cell labelling. After the final washing step, the cell pellet was resuspended to an OD_600_ = 1.5 in fresh, pre-warmed (to 37 °C) CDM and incubated at 37 °C with agitation at 180 rpm. Samples were collected at 30 min intervals over a 2 h period thus completely spanning the live-cell imaging window and the OD_600_ was recorded at each sampling time point. Samples were immediately transferred to pre-chilled containers and placed on ice to arrest cell growth. Cells were then pelleted via centrifugation (6000 x g, 10 min, 4 °C) and washed three times with ice-cold, 0.2 µm filtered PBS (pH 7.4) supplemented with 0.5 mM MgCl_2_ and 0.15 mM CaCl_2_.

Washed cell pellets were resuspended in ice-cold, 0.2 µm filtered lysis buffer (20 mM Tris-Cl pH 7.5, 150 mM NaCl, 5 mM MgCl_2_) supplemented immediately before use with DNase/RNase and lysed carefully via probe sonication on ice 10x (5-7 sec pulses: 30 sec recovery), 25% power, taking care to ensure minimal membrane fragmentation. Unbroken cells and large debris were removed via centrifugation (10,000 x g, 20 min, 4 °C). Clarified lysates were transferred to ice-cold polycarbonate ultracentrifugation tubes and centrifuged (100,000 x g, 60 min, 4 °C, Beckman Coulter TLX Optima benchtop ultracentrifuge, TLA 100.4 fixed angle rotor). The supernatant was retained as the soluble fraction (cytoplasmic/periplasmic) and the crude total membrane pellet was washed by resuspending gently (to minimise membrane fractionation) in ice-cold, 0.2 µm filtered lysis buffer and repeating the ultracentrifugation step.

After aspiration of the supernatant, the total membrane fraction was resuspended in ice-cold lysis buffer plus 1% (w/v) *N*-lauryl sarcosinate (Sarkosyl) (Sigma-Aldrich) and incubated on a slow moving rotary wheel (12 rpm, 80° incline relative to benchtop) for 60 min at room temperature. Samples were then transferred to ice-cold polycarbonate ultracentrifugation tubes and ultracentrifuged (100,000 x g, 45 min, 4 °C) yielding a Sarkosyl-soluble inner membrane (IM) fraction (supernatant) and a Sarkosyl-insoluble outer membrane (OM) fraction (pellet). The OM pellet was washed once via resuspension in ice-cold lysis buffer (no detergent) followed by ultracentrifugation (100,000 x g, 30 min, 4 °C) and subsequently resuspended in ice-cold lysis buffer. Material was then concentrated via cold acetone precipitation by adding 4x volumes of ice-cold acetone and incubation at -20 °C for 4 h followed by pelleting via centrifugation (10,000 x g, 30 min, 4 °C). Fractions were then resuspended in 0.2 µm filtered Buffer EB (Qiagen) and prepared for TSDS-PAGE as described above (including proteinase K digestion where appropriate) (Supplementary Table 14). TSDS-PAGE was carried out as described above with equal amounts of each sample added based on sampling volume and culture density (OD_600_) at the point of sampling. AF488-labelled material was visualised by in-gel fluorescence imaging using a Typhoon 5 bioimaging system (488 nm laser excitation and 525 BP emission filter). The resulting in-gel fluorescence images were processed using ImageJ / FIJI software.

### TSDS-PAGE analysis of background LPS turnover in intact *E. coli* BW25113 cells and its corresponding accumulation in culture supernatants due to OMV release

Labelled intact cell samples were prepared via the same Kdo-N_3_ / native pulse-chase protocol used to monitor background LPS turnover on a cell-by-cell basis using dSTORM analysis, but without the final paraformaldehyde fixation step. LPS was extracted from cell pellets using the protocol described above. The volume of LPS sample loaded was normalised based on OD_600_ measurements (Supplementary Fig. 3c,d) done at the point of sampling by diluting the sample with the appropriate volume of HPLC-quality water to ensure equal amounts of total LPS were loaded in each lane. 6.67 µL aliquot of 4 x loading buffer (Supplementary Table 14) with 3% (v/v) β-mercaptoethanol was added to each 20 µL cell pellet-derived LPS sample in a 500 µL microcentrifuge tube. Samples were then mixed via repeated pipetting and gentle vortexing, and then briefly centrifuged. Suspensions were then incubated at 40 °C for 1 h in a heated water bath. After incubation, the samples were re-vortexed and briefly centrifuged before loading 20 µL of each sample per well in a Tricine gel.

### OMV isolation, background LPS fluorescent labelling and OMV-derived LPS extraction

After intact cell pellet and culture supernatant isolation via centrifugation (10,000 x g, 4 °C, 20 min,) from Kdo-N_3_ / native Kdo pulse-chase samples, the supernatants were transferred to sterile pre-chilled 2.0 mL microcentrifuge tubes and immediately re-centrifuged to remove any remaining residual debris (10,000 x g, 4 °C, 20 min,). Double centrifuged supernatants were then carefully aspirated into new, sterile pre-chilled 2.0 mL microcentrifuge tubes and then passed slowly through 0.45 µm and 0.22 µm pore size hydrophilic (PES) syringe filters into pre-chilled, polycarbonate tubes. OMVs in the culture supernatant were then pelleted via ultracentrifugation (Beckman Coulter TLX Optima benchtop ultracentrifuge, TLA 100.4 fixed angle rotor, 110,000 x g, 4 °C, 12 hr,). Supernatants were aspirated and OMV pellets resuspended in 200 µL pre-chilled 0.2 µm-filtered PBS pH 7.4 (with 2 mM MgSO_4_ and 0.5 mM CaCl_2_ added) and transferred to sterile 2 mL microcentrifuge tubes. Background Kdo-N_3_-containing LPS in OMVs was then fluorescently labelled via CuAAC using the identical protocol used to label background LPS in the OM of intact cells, thereby ensuring no variations in labelling efficiency arose from differences in labelling protocols. OMV Click-iT labelling suspensions in 2.0 mL microcentrifuge tubes were incubated on a slow-moving rotary wheel at room temperature for 30 min (12 rpm, 80 ° incline relative to benchtop) protected from light.

Suspensions were then transferred to 3.5 kDa D-Tube Midi dialysers (Merck) and suspensions dialysed twice for 6 to 8 hours into 3 litres of unsupplemented M9 CDM (with 2 mM MgSO_4_ and 0.5 mM CaCl_2_ added) at 4 °C, protected from light to remove spent Click-iT mix components. Dialysed suspensions were transferred to 250 µL pre-chilled polycarbonate ultracentrifugation tubes and OMVs bearing AF488-labelled background LPS were pelleted via ultracentrifugation (110,000 x *g*, 4 °C, 12 h,). Supernatants were aspirated and OMV pellets dried for 5 – 10 min under a steady stream of nitrogen gas. OMV pellets were then resuspended in 30 µL LPS lysis buffer with 2% (v/v) β-ME (Supplementary Table 21) via repeated pipetting and transferred to 500 µL microcentrifuge tubes. Tubes were incubated in an ultrasonic water bath for 5 minutes at 40 °C to lyse OMVs. Suspensions were stored at 4 °C, protected from light prior to TSDS-PAGE analysis. OMV-derived LPS samples were prepared for TSDS-PAGE using the same method as for LPS extracted from bacterial cell pellets.

### Quantifying background AF488-labelled LPS in bacterial OM and OMV-derived fractions using TSDS-PAGE and densitometry

To verify AF488-LPS signal intensities were not influenced by unexpected differences in total LPS loading, control experiments were done using non-Kdo-N_3_ labelled samples (since Kdo-N_3_ incorporation into LPS affects Pro-Q LPS staining efficiency). The samples were loaded into the wells of a Tricine gel based on OD_600_ measurements at the point of sampling and total LPS visualised via Pro-Q 300 staining (according to the protocol described above, Fig. 6d and Supplementary Fig. 6c)*.* Densitometric analyses were done using ImageJ / FIJI software. The average raw AF488-LPS band intensities were measured and recorded. The average AF488-LPS band intensity (minus background signal) was normalised to enable the calculation of average relative changes in background AF488-LPS content across multiple experimental replicates and to facilitate comparison between bacterial OM and OMV-derived LPS. Normalised values ranged from 1 at 0 h post-Kdo-N_3_ removal to 0 at 3.5 h post-Kdo-N_3_ removal. Normalised background AZ488-LPS values for individual replicates and mean AZ488-LPS values for cell pellets (Fig. 6d and Supplementary Fig. 6a) and OMV-derived samples (Fig. 6d and Supplementary Fig. 6b) were plotted against time post-Kdo-N_3_ removal.

### Assessing Kdo-N_3_ incorporation into the OM of exponential and stationary phase *E. coli* BW25113 cells

A starter culture was prepared by inoculating 5 mL of supplemented M9 CDM with a single colony of *E. coli* BW25113 cells from a freshly streaked LB/agar plate. This culture was incubated post-inoculation for 10 h at 37 °C with shaking (220 rpm). Aliquots of this culture were used to inoculate two fresh 5 ml volumes of supplemented M9 CDM to a starting OD_600_ value of 0.05. One of the two cultures was immediately charged with Kdo-N_3_ to a final concentration of 4 mM, and both cultures were incubated for 10 h at 37 °C with shaking (220 rpm). The OD_600_ of the two cultures was measured (2.8 and 2.75, respectively) to check for equivalent rates of growth and to ensure both cultures had reached stationary phase. The culture charged with Kdo-N_3_ in exponential phase was harvested via centrifugation and LPS was labelled with AF488-alkyne via CuAAC. The second culture was charged with 4 mM Kdo-N_3_ and re-incubated for a further 10 h at 37 °C with shaking (220 rpm). Cells were then harvested via centrifugation and the LPS was fluorescently labelled with AF488-alkyne via CuAAC using the standard protocol described above. Labelling efficiency was assessed via confocal fluorescence microscopy ensuring standard 488 nm laser powers and detector gain settings were employed to enable a quantitative comparison of the two cultures.

### OMV isolation and purification

OMVs were isolated from the *E. coli* BW25113 strain cultured in nanoparticle-free supplemented M9 CDM. A volume of nanoparticle-free supplemented M9 CDM was inoculated using washed bacterial cells obtained from a stationary phase culture grown in the same nanoparticle-free medium. The bacterial cells were first isolated from the stationary phase culture by centrifugation at 3,400 x g for 20 min at 20 °C using a temperature-controlled bench-top centrifuge. After centrifugation, the supernatant was carefully removed under sterile conditions. The cell pellet was resuspended in 10 ml of room temperature, sterile, nanoparticle-free supplemented M9 CDM and the cell suspension was centrifuged as above. After centrifugation, the supernatant was removed, and the resulting bacterial cell pellet was resuspended as above. This wash step was repeated once more, and the final bacterial cell pellet was resuspended in 3 ml of sterile, nanoparticle-free supplemented M9 CDM. This cell suspension was used to inoculate a 100 ml volume of nanoparticle-free supplemented M9 CDM to an OD_600_ of ~0.1 and the inoculated culture was incubated at 37 °C with shaking (165 rpm). An exact volume (5-6 ml) was removed from the bacterial culture for the purification of OMVs at designated time points. The volume removed was immediately placed on wet ice to suspend bacterial growth and then stored for no longer than 24 h at 4 °C prior to OMV purification.

Bacterial cells were removed from the OMV-containing culture volume by centrifuging at 5,000 x g for 20 min at 4 °C. The supernatant containing the OMVs was carefully removed without disturbing the cell pellet after each centrifugation step. After centrifuging twice in this manner, the OMV-containing supernatant was immediately purified using one of the following procedures: (i) the supernatant was filtered through a 0.45 µm (Filtropur S, Sarstedt) pore size hydrophilic (PES) syringe filter, or (ii) the supernatant was sequentially filtered through 0.45 µm and 0.22 µm (Appleton Woods) pore size hydrophilic syringe filters. Both filtration procedures yielded similar OMV size distributions. Procedure (i) retained a larger number of OMVs in the supernatant; thus, samples purified in this way better represented the native OMV composition of the bacterial culture. Aliquots (10-20 µl) of the purified OMVs were spotted onto LB-Miller agar plates and the plates were incubated at 37 °C for 24 h to confirm the absence of intact, viable bacteria. The purified OMVs were stored at 4 °C prior to measuring their concentration by nanoparticle tracking analysis.

### Nanoparticle tracking analysis (NTA) of OMVs

NTA was done using a ZetaView® Quatt instrument (Particle Metrix) within 48 h of purifying the OMVs from bacterial culture. All OMV samples were centrifuged at 21,000 x g at 4 °C in a 2 ml micro-centrifuge tube for 30 min just prior to NTA, then transferred to a fresh micro-centrifuge tube and allowed to reach room temperature (21 °C) before the measurement. Ideal OMV concentrations for NTA (yielding 50-200 particles/frame) were determined by pre-testing each OMV sample after dilution with nanoparticle-free unsupplemented M9 CDM (with <10 particles/frame) to a final volume of 1 ml. The NTA instrument was calibrated daily using certified 100 nm diameter polymer nanospheres (NanoStandards, Applied Microspheres). Triplicate technical replicates were measured for each OMV sample. For each sample measurement, the cell was scanned at 11 different positions while capturing 60 frames of video at each position (with 488 nm laser illumination, video frame rate = 30 frames per second, video setting = high, shutter = 100, camera sensitivity = 85).

After capture, the videos were analysed using the ZetaView® software (version 8.06.01 SP1) and standardised analysis parameters (*i.e*. minimum particle area = 10, maximum particle area = 1000, minimum particle brightness = 30, tracking radius = 100, minimum trace length = 15 frames). Only videos where 7 or more positions met the selection criteria were used to determine the OMV concentration. Particles up to 1000 nm in diameter could be tracked using this NTA procedure. We routinely observed that > 90% of the OMVs purified by procedure (ii) had a diameter ≤ 200 nm, and that > 99% of the OMVs purified using procedure (i) had a diameter ≤ 500 nm. Non-linear regression of the skewed OMV size distribution for each set of technical replicates (*N* = 3) was done for particles using GraphPad Prism (v10.3.1) assuming a log-normal distribution of particle sizes.

### Growth phase assignment

For all experiments, exponential, late exponential, and stationary phases were assigned from the growth curves constructed from OD_600_ measurements of the sample culture, with stationary phase onset defined operationally as the transition point at which the growth curve first departed from exponential increase and the OD_600_ value begins to decrease towards an asymptote, as determined from successive OD_600_ measurements of the same culture. Because some assays were initiated at different starting optical densities for practical reasons (*e.g*. OD_600_ ≈ 0.5 for pulse–chase labelling/imaging versus OD_600_ ≈ 0.05 for NTA), the absolute time-to-stationary differs between assays, but the growth phase assignment uses the same OD_600_-plateau criterion (see Supplementary Figs. 3c,d and 11b, Supplementary Tables 22 and 23). Over the analysed stationary-phase time window, viable cell counts (CFU mL⁻^1^) remained approximately stable (Supplementary Fig. 11 and Supplementary Table 23), indicating minimal loss of viability under these conditions. Accordingly, net population growth is negligible over this window (OD_600_ asymptote), and we treat growth-associated dilution as minimal.

### Determining the viable cell concentration of bacterial cultures (CFU equivalents)

CFU-equivalent measurements were used to (i) corroborate the growth-phase assignments defined from growth curves (above) and (ii) derive time-dependent OD_600_-to-viable-cell conversion factors for normalising OMV counts on a per-viable-cell basis. Viable cell concentration was determined by serial dilution and colony enumeration (plate count method). Viable cell concentration is reported as colony forming units per millilitre (CFU mL^-1^). For each plated dilution, CFU mL^-1^ was calculated as follows:

4 $${\rm{CFU}}{{\rm{mL}}}^{-1}=\left(\frac{{N}_{{colonies}}}{{V}_{{plated}}}\right)\times D$$

where *N*_*colonies*_ is the number of colonies counted, *V*_*plated*_ is the plated volume (mL), and *D* is the dilution factor (reciprocal of the plated dilution).

CFU mL^-1^ values were calculated for each time point and paired with measured OD_600_ values obtained from the same cultures. From these paired measurements, an OD-to-viable-cell conversion factor, *k*(*t*) was determined at each CFU time point (*t*) as follows:

5 $$k\left(t\right)=\,\frac{{\rm{CFU}}(t)}{{{\rm{OD}}}_{600}(t)}$$

with units of CFU equivalents mL^-1^ OD_600_^-1^. This yielded representative conversion factors of 2.00 ×10^8^ CFU equivalents mL^-1^ OD_600_^-1^ during exponential phase growth and 3.44 ×10^8^ CFU equivalents mL^-1^ OD_600_^-1^ after entry into stationary phase (Supplementary Table 23). *k*(*t*) remained stable during late stationary phase, consistent with minimal loss of viability over this interval.

To obtain viable cell concentrations at additional OD_600_-only time points (for time-matched normalisation of OMV NTA measurements, Supplementary Fig. 11 d), *k*(*t*) was linearly interpolated between CFU sampling time points to obtain *k*_*interp*_(*t*) and OD_600_ values converted to viable cell concentrations as follows:

6 $${{CFU}}_{{eq}}\left(t\right)={{OD}}_{600}\left(t\right)\,\times \,{k}_{{interp}}(t)$$

### Normalisation of outer membrane vesicle concentration to viable cell concentration (CFU equivalents)

To express extracellular OMV abundance on a per-cell basis, OMV concentrations determined by NTA (particles mL^-1^) were normalised to the viable cell concentration (CFU equivalents mL^-1^) at the matched time points. Viable cell concentration was determined by CFU plate counts and OD_600_ calibration as described above. For each time point *t*, OMVs per viable cell were calculated as follows:

7 $${OMVs}\,{per}\,{viable}\,{cell}\left(t\right)=\,\frac{{OMVs}(t)}{{{CFU}}_{{eq}}\left(t\right)}$$

where *OMVs*(*t*) is the extracellular OMV concentration measured by NTA at time *t* (particles mL^-1^) and *CFU*_*eq*_(*t*) is the viable cell concentration at time *t* (CFU equivalents mL^-1^).

This normalisation yields a dimensionless per cell metric (particles per CFU-equivalent cell) that enables comparison across stationary phase time points despite changes in culture density. Because NTA quantifies extracellular particle concentration in clarified, processed supernatants, *OMVs per viable cell*(*t*) reflects net extracellular OMV accumulation (production minus losses due to sample handling and purification), rather than an instantaneous single-cell OMV production rate. Removal of intact cells by repeated centrifugation and subsequent passage through 0.45 µm and 0.22 µm filters, together with storage and transfer steps, can reduce particle recovery due to adsorption to plastics, vesicle aggregation, or retention on filters. However, because the OMV isolation and NTA workflows were stringently standardised and applied identically at each time point, the resulting per-cell metric supports robust relative comparisons of extracellular OMV accumulation across stationary phase and should be interpreted as a conservative estimate (lower bound) of the absolute number of OMVs released per cell.

### Modelling background LPS dilution and OMV mediated export

To quantify the contribution of OMV production to observed rates of background (old) LPS turnover beyond dilution resulting from cell growth and division, we implemented a population mass-balance model aligned to the pulse-chase time origin; *t* = 0 was defined as the time of Kdo-N_3_ removal (termination of LPS metabolic labelling, OD_600_ = 0.5). Under this scheme, no Kdo-N_3_-containing LPS was inserted after *t* = 0 and all LPS inserted after this point is classified as newly inserted (new) LPS. The newly inserted LPS population was unable to incorporate Kdo-N_3_ into its inner core oligosaccharide (due to its prior removal from the culture medium) and therefore was not fluorescently labelled.

Background, fluorescently labelled LPS associated with intact cell OM fractions was extracted and quantified via TSDS-PAGE and densitometry (as described above, Fig. 6d,e and Supplementary Fig. 6a,c). For each time point *t*, the TSDS-PAGE signal was converted to the background LPS density term ${\rho }_{{old}}(t)$ by normalising the background-labelled LPS signal to the total LPS-accessible OM area present in the culture at that time point. The normalised background LPS fraction remaining at time *t*, ${f}_{{\rm{obs}}}(t)$ plotted in Supplementary Fig. 16c, was then calculated as follows:

8 $$f_{\rm{obs}}(t)=\frac{{\rho }_{{\rm{old}}}(t)}{{\rho }_{{\rm{old}}}(0)}$$

where$\,{f}_{{\rm{obs}}}\left(t\right)$ is the normalised background LPS fraction remaining at time *t*, ${\rho }_{{\rm{old}}}\left(t\right)$ is the TSDS-PAGE fluorescent background LPS signal at time *t*, and ${\rho }_{{\rm{old}}}\left(0\right)$ is the TSDS-PAGE fluorescence background LPS signal at *t* = 0 (point of Kdo-N_3_ metabolic labelling termination).

The dilution-only expectation assumes that the number of background-labelled molecules present at *t* = 0 is conserved (i.e no active removal) and that the background LPS signal decreases solely because the total LPS accessible OM area in the cell population increases as cells elongate and divide while new LPS is inserted. Total LPS accessible OM area per mL was estimated as follows:

9 $${A}_{{\rm{cell}}}\left(t\right)={N}_{{cell}}\left(t\right)\cdot {A}_{{cell},{per}\,{cell}}(t)$$

where ${N}_{{cell}}\left(t\right)$ is the intact viable cells mL^-1^ at time *t*, and ${A}_{{cell},{per\; cell}}(t)$ is the LPS accessible OM surface area per cell. The dilution-only fraction (${f}_{{\rm{dil}}}(t)$) was calculated as follows:

10 $$f_{{\rm{dil}}}(t)=\frac{{A}_{{\rm{cell}}}(0)}{{A}_{{\rm{cell}}}(t)}$$

OMV concentration at time *t* (${C}_{{\rm{OMV}}}(t)$, particles mL^-1^) and size distributions were measured by NTA on culture supernatants prepared using 0.45 µm filter clarification only as described above. To incorporate the resulting long-tailed OMV diameter distributions (Supplementary Fig. 17), the mean OMV surface area per particle at time *t* ($\langle {A}_{{\rm{OMV}}}\rangle (t)$) was computed from the full diameter distribution as follows:

11 $${{\langle }}{A}_{{\rm{OMV}}}{{\rangle }}(t)=\pi {{\langle }}{d}^{2}{{\rangle }}$$

where 〈*d*^2^〉 is the mean of squared diameters (converted to µm^2^) across the distribution at time *t*.

The cumulative background LPS pool exported via OMVs was estimated by converting OMV surface area to an equivalent number of LPS molecules using an assumed LPS molecular footprint, ${a}_{{\rm{LPS}}}\,$(µm^2^ molecule^-1^), and then expressing this as a fraction of the initial background LPS pool at *t* = 0 as follows:

12 $$f_{{\rm{OMV}}}(t)=\frac{{C}_{{\rm{OMV}}}(t)\,\langle {A}_{{\rm{OMV}}}\rangle (t)}{{A}_{{\rm{cell}}}(0)}$$

This formulation corresponds to a scenario in which the outer leaflet of the OMV membrane is treated as LPS-dominated and the OMV-exported LPS is assigned to the pre-existing (background-labelled) pool, in line with our OM buckling model described below and supported by our analysis demonstrating significant background LPS enrichment in OMVs (Supplementary Fig. 13 and Supplementary Table 24). The predicted background fraction remaining under dilution plus OMV export was calculated as follows:

13 $$f_{\rm{model}}(t)=f_{\rm{dil}}(t)-f_{{\rm{OMV}}}(t)$$

To compare observed background loss with dilution and OMV export on the same scale, the excess loss beyond dilution was defined as follows:

14 $$\Delta f(t)=f_{{\rm{dil}}}(t)-f_{{\rm{obs}}}(t)$$

and the time-dependent values of Δf(t) and ${f}_{{\rm{OMV}}}(t)$ are plotted in Supplementary Fig. 16d.

### Derivation of outer membrane buckling model for OMV formation

To derive a general expression for the critical size of an OMV formed by membrane blebbing from an LPS-rich region in the bacterial OM, we assume these regions or patches are comprised solely of LPS and are pinned at their periphery to the cell wall by relatively immobile OMP-rich regions (Fig. 7a). These patches will tend to bulge outwards due to the intrinsic curvature of an asymmetric membrane bilayer with inner and outer leaflets comprised of phospholipid and LPS, respectively. However, the patches remain stable — resist blebbing — due to the intrinsic bending stiffness of the OM and anchoring by Braun’s lipoprotein, provided the characteristic radius of the LPS patch is below a critical value. For the isotropic case, the critical radius is given by the following:

15 $${R}_{c}=\sqrt{\frac{3{\kappa }_{b}}{2\sigma }}$$

where *κ*_*b*_ is the membrane bending stiffness and *σ* is the compressive stress in the membrane.

The load bearing properties of the OM, including its ability to sustain compressive stress, have been demonstrated previously^5^. When the patch radius exceeds *R*_*c*_, the buckled state becomes unstable, leading to membrane blebbing. The equation for *R*_*c*_ is consistent with the published literature^112^. However, using this expression to predict critical OMV size is challenging because the value of *σ* is generally unknown.

It has been shown that during exponential phase growth, the OM bears little or no load, and consequently the value of *σ* is relatively small^5^. Based on the equation for *R*_*c*_, we would expect membrane blebbing to be a relatively rare event during early to mid-exponential phase growth, and this conclusion is consistent with our time-dependent measurements of OMV concentration for wild-type *E. coli* BW25113 cells (Fig. 6f). To understand the dynamics of *σ* during stationary phase, we note that while the membrane area (*A*_*s*_) anchored to the peptidoglycan cell wall by OmpA and Braun’s lipoprotein remains constant, LPS continues to be inserted at approximately a constant rate. Continued insertion of LPS leads to a time-dependent increase in *σ* as follows:

16 $$\sigma \left(t\right)={\kappa }_{a}\frac{{Qt}}{{A}_{s}}$$

where *κ*_*a*_ is the membrane stretch modulus, *Q* is the inserted membrane area flux, and *t* is time.

The characteristic radius of the blebbing membrane (*R*_*b*_) in an LPS patch is obtained by evaluating the equation for *R*_*c*_ using a time-dependent value of *σ* corresponding to a characteristic membrane blebbing time (*t*_*b*_) as follows:

17 $${R}_{b}={R}_{c}\left(\sigma \left({t}_{b}\right)\right).$$

After a membrane blebbing event, the LPS patch is removed from the OM which leads to relaxation of the compressive stress. Afterwards, the continued insertion of new LPS leads to a gradual build-up of compressive stress as described by the equation for $\sigma \left(t\right)$ until another membrane blebbing event in an LPS patch. In a steady state, the rate of LPS insertion must balance the rate of membrane area loss due to membrane blebbing and OMV release. This steady state condition yields the following:

18 $$Q=\frac{\pi {R}_{b}^{2}}{{t}_{b}}.$$

Solving the system of equations ${R}_{c}$, ${R}_{b}$ and *Q* leads to the following expression for the characteristic radius of the blebbing membrane:

19 $${R}_{b}=\sqrt{w\,l}$$

where

20 $$w=\sqrt{\frac{3{\kappa }_{b}}{2{\kappa }_{a}}}$$

and

21 $$l=\sqrt{\frac{{A}_{s}}{\pi }}.$$

The resulting equation for ${R}_{b}$ is independent of *Q*, and this fortuitous circumstance makes this scaling law robust against the natural variability of LPS insertion rates observed in vivo. Since the diameter (*d*) of a vesicle formed from a single LPS patch is equal to ${R}_{b}$, we can predict the critical OMV size with the following expression:

22 $$d=\sqrt{w\,l}.$$

During late exponential phase and the stationary phase growth, asymmetric expansion of the OM relative to the cell wall would arise and lead to a time-dependent increase in *σ*. In a steady state, the rate of LPS insertion must balance the rate of OM area loss due to blebbing. The radius of a blebbing LPS patch (*R*_*b*_) at steady state was estimated using literature values of *κ*_a_ (=0.03–0.24 N/m^113^) and our experimentally determined value of *A*_*s*_ (=12.6 µm^2^ for a typical cell size). Estimates of *κ*_b_ and *κ*_a_ were made by fitting atomic force microscopy measurements of membrane stiffness to a thin-shell membrane model^5,114^ where the characteristic length scale (*w*) is related to the shell thickness (*h*) by *w* = *h*/2^3/2^^115^. The characteristic diameter (*d*) of the OMV formed from the membrane bleb would be equal to *R*_*b*_. We obtain *d* = 73–122 nm for an OMV released by this blebbing mechanism assuming *h* is equivalent to the OM thickness and using a range of experimentally determined OM thicknesses ( = 7.5–21 nm^116,117^) and *l* = 2 µm as measured for a typical cell. This estimate of *d* is in good agreement with our experimental results (Supplementary Fig. 8b, shaded region), considering the simplicity of the model and the absence of fitting parameters.

### Computational modelling of OM organisation, turnover, and OMV formation

#### Simulation domain, mask and coarse-graining

We simulated a half-cell field (2.6 µm × 1.1 µm; total area, ${A}_{{field}}$ = 2.86 µm^2^) mapped to a 780 pixels × 330 pixels grid (pixel size = 3.33 nm). A static allowed-area mask that covered a fraction (${f}_{{allow}}$ = 75%) of the field was defined to approximate the LPS-accessible outer leaflet (allowed area, ${A}_{{allow}}=0.75{x}2.86=2.145\,$µm²)^76–78^. The remaining 25% represented OMP-occupied, LPS-inaccessible excluded/obstacle regions (rendered as black in figures; sometimes referred to as voids in the simulation mask) consistent with OMP islands and the confined lateral mobility of OMPs and LPS in the OM of *E. coli*^11,32^. The number of simulated LPS molecules in the allowed area was held approximately constant at *N*_*sim*_ = 8 × 10^4^ per frame. This *N*_*sim*_ is a coarse-grained representation of the biological copy number expected in this field. In the insertion-trapping simulation (Model 1), the allowed-area mask is static. In the phase-separation simulation (Model 2), the mask undergoes a gentle, area-preserving annealing at discrete 10 min intervals that increases its correlation length and produces the slow coalescence of voids, simulating the slow OMP-domain merging expected in a membrane system where the evolution of component organisation is driven principally by phase separation^33,41,42^.

To relate simulated counts to an estimate of the numbers of LPS molecules within ${A}_{{allow}}$, we estimated the area density of LPS as follows:

23 $${\rho }_{{LPS}}\approx \frac{{N}_{{LPS}}}{{f}_{{LPS}}\,{A}_{{OM}}}$$

where ${{N}}_{{LPS}}$ is the total number of LPS molecules per cell (with ${N}_{{LPS}}\in \left[1.4,\,2.0\right]\,\times {10}^{6}$ molecules per cell^77–79,118^), ${A}_{{OM}}$ is the OM surface area per cell ( = 4.31 µm^2^^119^), ${\rho }_{{LPS}}$ is the area density of LPS molecules (LPS µm^−2^), and ${f}_{{LPS}}$ is the fraction of the OM surface occupied by LPS (${f}_{{LPS}}$ = 0.75^76–78^). Numerically, this yields the following:

24 $${\rho }_{{LPS}}\in \left[4.33,\,6.19\right]\,\times {10}^{5}{\rm{LPS}}\; {\rm{\mu }}{{{m}}}^{-2}$$

and the simulation field is as follows:

25 $${A}_{{field}}=2.6{\rm{\mu }}m\,\times 1.1{\rm{\mu }}m=2.86\,{\rm{\mu }}{m}^{2}.$$

The allowed (LPS-accessible) area is calculated as follows:

26 $${A}_{{allow}}={f}_{{\!allow}}{A}_{{field}}=0.75\,x\,2.86=2.145{\rm{\mu }}{m}^{2}$$

and the expected number of LPS molecules within ${A}_{{allow}}$ ($\left\langle {N}_{{field}}\right\rangle$) is calculated as follows:

27 $$\left\langle {N}_{{field}}\right\rangle=\,{\rho }_{{LPS}}{A}_{{allow}}\Rightarrow \left\langle {N}_{{field}}\right\rangle \in \left[9.29,13.27\right]\times {10}^{5}{\rm{LPS\; molecules}}.$$

Given the number of simulated particles restricted to the allowed area *N*_sim_ = 8 × 10^4^, the corresponding coarse-graining factor (*γ*) relative to the biological count is given by:

28 $$\gamma=\frac{\left\langle {N}_{{field}}\right\rangle }{{N}_{{sim}}}\in \left[11.6,16.6\right]$$

thus one simulated LPS particle represents between 12 and 17 LPS molecules.

#### LPS turnover kinetics (common parameters for both models)

Simulations advanced in discrete steps of Δ*t* = 5 min from *t* = 0 to 120 min (plots and .csv files were exported every 10 min). Background (old) LPS decayed with per-step probability where:

29 $${p}_{{decay}}=1-{2}^{-\Delta t/{t}_{{1}/_2}}$$

with ${t}_{1/2}$ = 90 min, the single-exponential half-life obtained by fitting the decline of old LPS coverage in the pulse-chase dSTORM experiments (Figs. 2e,f, 3) and consistent with the average rate of background LPS loss from the OM of cells over the first two hours (Fig. 6d,e).

The fraction of newly inserted LPS increased linearly to ~0.65 at 120 min, reproducing the measured composition shift (reciprocal rise of newly inserted LPS vs decline of background LPS) observed experimentally (Fig. 2e,f, Supplementary Tables 16 and 17). To minimise discretisation artefacts, we implemented this as a smooth, late-weighted specifies a target newly inserted LPS fraction at each step (not an absolute number).

Total LPS coverage in the field was held approximately constant at the target ${N}_{{sim}}=8\,\times \,{10}^{4}$ simulated particles (background + newly inserted) across the allowed area. At each 5 min simulation step, we applied updates in the following order: (i) background LPS decay (using ${p}_{{decay}}$) and OMV-associated removals, (ii) computation of the target newly inserted LPS fraction, and (iii) new LPS insertion up to a per-step cap so that the running composition follows the experimental trajectory and the combined count respects the following rule:

30 $$\left({Background}\; {LPS}+{Newly}\; {inserted}\; {LPS}\right)\le {N}_{{sim}}$$

When removals exceeded insertions in a given step, the total number of particles was allowed to transiently fall slightly below ${N}_{{sim}}$ and was not renormalised upward.

#### Burst-like new LPS insertion and restricted lateral diffusion of LPS (common settings for both models)

Within the allowed area, we pre-sampled 300 candidate insertion hotspots and, at each 5 min simulation step, randomly activated 110–150 sites. Newly synthesised LPS was inserted as compact two-dimensional Gaussian (typical *σ* ≈ 20–40 nm, with light ellipticity jitter), consistent with discrete LptDE-linked delivery to the outer leaflet^24,36,120^.

Between updates, both LPS particle types attempted highly restricted lateral displacements (*σ* ≈ 40 nm per 5 min) in-line with experimental observations that have shown LPS in *E. coli* BW25113 undergoes tightly restricted lateral diffusion^10,11^. Any LPS particle proposed movements that resulted in their entry into voids or crossing boundaries were rejected, reflecting the very low lateral mobility in the outer leaflet of the OM and confinement by OMP assemblies^10,11,32^. Diffusive step sizes were matched across both models (per-step standard deviation on the order of a few nm at 5 min cadence); only Model 2 adds an interaction-driven drift term. Since turnover, insertion, lateral diffusion and OMV biogenesis were identical in both the insertion-trapping and phase separation models, the observed variations in newly inserted versus background LPS distributions arose from differences in the principles underpinning organisation (i.e. insertion-trapping versus phase separation).

#### OMV biogenesis via OM buckling (release of OM compressive stress by vesiculation)

We modelled OMV biogenesis identically in both models, as a means of releasing compressive stress in LPS-rich patches whose rims are pinned by OM–peptidoglycan (PG) anchoring (for example, by Braun’s lipoprotein), and where patch centres are comparatively weakly anchored (Fig. 7a). Continued new LPS insertion elevates in-plane compressive stress until a patch bulges and blebs to release an OMV. At each 5 min simulation step, we drew a Poisson number of events corresponding to ~3 per 30 min on average and placed them at peaks of total LPS density (top 10%) at locations away from OMP voids. Each event removed only a fraction of the local LPS within a radius of 80–90 nm footprint and re-packed remaining LPS particles with a small jitter (re-packing jitter ~20 nm), so no persistent hole remained thereby mimicking OMV pinching (Fig. 7a) and membrane reshaping, rather than perforation. This implementation aligns with the load-bearing role of the OM and OM–PG tethering mechanics^5,71^ along with the observed hyper-vesiculating phenotypes observed in *E. coli* mutant strains (Δ*tol-pal* and Δ*lpp)* with weakened OM-PG tethers^72^. The white circles used in the figures are visual markers only and do not reflect OMV dimensions.

#### Insertion-trapping model

Model 1 enforced persistent segregation of background and newly inserted LPS by application of two local spatial rules while maintaining identical global insertion and turnover kinetics to Model 2. The two spatial rules were as follows: (i) At active hotspots, candidate new LPS particle insertions were accepted only where the smoothed OLD density is below a fixed threshold. This biases NEW to fill OLD-sparse patches, producing spatial anti-coincidence without altering the total number of new LPS particles inserted. (ii) Background LPS removal was locally increased in regions already enriched for new LPS insertion (probability scaled by the local unit-normalised new LPS particle density) and globally normalised at each step so that the population half-life of LPS particles remained at 90 min.

To emulate the compressive stress at highly active insertion sites without creating artefacts, recent newly inserted LPS bursts nudge background LPS slightly outward within a 50–60 nm neighbourhood in small, isotropic displacements, preserving local LPS packing density in background LPS-rich patches. The Python script and associated README file are available through the Persistent_LPS_insertion_OM_OMVs GitHub repository and have been permanently archived on Zenodo at https://doi.org/10.5281/zenodo.21326870^105^.

#### Phase separation model

Model 2 incorporated the same LPS insertion and turnover schedule, confined lateral diffusion and OMV biogenesis module as Model 1, but replaced the insertion-trapping rules with like-like interactions and slow OMP void coalescence. The phase separation-specific rules were as follows: (i) At each step, we computed smooth density fields and added a weak drift up the gradient of each particle’s own density (Metropolis/Cahn–Hilliard-style), increasing co-locating (co-clustering) and promoting domain coarsening while conserving particle numbers. No explicit newly inserted versus background LPS avoidance rules were applied. (ii) The allowed-area mask was lightly annealed at each 5 min simulation step (diffuse-threshold cycle) to increase the mask’s correlation length whilst preserving ${f}_{{allow}}$, yielding the slow merging of OMP-rich island voids expected in a membrane system undergoing demixing^43,44,85,86^.

Since all global kinetics matched those applied in Model 1, the distinctive signatures of this model (increasing DICE coefficient and *g*_*NB*_(0) > 0) arise from its intrinsic phase separation characteristics and not from differences in lateral diffusion, or LPS insertion and turnover rates. The Python script and associated README file are available through the Persistent_LPS_insertion_OM_OMVs GitHub repository at https://github.com/joenabarro-OM/Persistent_LPS_insertion_OM_OMVs/releases/tag/v1.0.2 and have been permanently archived on Zenodo at https://doi.org/10.5281/zenodo.21326870^105^.

### Calculation of zero-lag spatial cross-covariance for background and newly inserted LPS clusters in the model simulations

To quantify co-location of background and newly inserted LPS clusters independent of marginal coverage, we computed a zero-lag, coverage-normalised overlap statistic, *g*_*NB*_(0) on binary cluster masks using DBSCAN parameters equivalent to those used in the dSTORM analyses (Figs. 2e and 4, Supplementary Figs. 2e and 4). For each time point in the simulation, background and newly inserted (x, y) particle co-ordinates (in nm) were clustered via the DBSCAN application (ε = 30 nm; *minPts* = 7; core points only). Only core points were used to define robust cluster interiors. These points were converted to the image grid (780 pixels × 330 pixels, 3.33 nm pixel^−1^) on the allowed-area mask. This produced two Boolean masks *A* (background) and *B* (newly inserted) indicating pixels that belonged to the DBSCAN clusters. The DBSCAN parameters were fixed and held constant across models and simulation replicates (*N* = 5 for each model).

|*A*| and |*B*| are the number of true pixels in the allowed area of the OM, |*A* ⋀ *B*| is the count of the co-occupied pixels, and *N* is the number of allowed pixels. The fraction of pixels in the mask classified as background (*p*_*A*_), newly inserted (*p*_*B*_), and co-occupied (*p*_*AB*_) was calculated as follows:

31 $${p}_{A}=\frac{\left|A\right|}{N}$$

32 $${p}_{B}=\frac{\left|B\right|}{N}$$

33 $${p}_{{AB}}=\frac{{|A}\wedge {B|}}{N}.$$

The relative-to-random co-location is given by the following:

34 $${g}_{NB}\left(0\right)=\,{p}_{AB}-\,{p}_{A}{p}_{B}.$$

This subtracts the expected random overlap ${p}_{A}{p}_{B}$ from the observed overlap. Thus ${g}_{{NB}}\left(0\right)$ is dimensionless and interpretable with *g*_NB_(0) > 0 indicating greater co-location compared to random, ${g}_{{NB}}\left(0\right)=0$ indicating random localisation, and ${g}_{{NB}}\left(0\right) < 0$ indicating anti-association with less co-location compared to random. ${g}_{{NB}}\left(0\right)$ values were computed per time point for each run (*N* = 5) in both model simulations, and mean run values with standard errors were calculated and reported (Fig. 7d). Edge cases (with no clusters in *A* or *B*) yielding undefined overlap were marked *NaN* and excluded from reported summaries.

### Calculation of co-clustering metric (DICE coefficient) for background and newly inserted LPS clusters in the model simulations

As a complementary, bounded similarity index, a DICE coefficient was computed between background and newly inserted LPS particle cluster masks at each time point in every run for both model simulations. Masks were constructed via DBSCAN (*ε *= 30 nm; *minPts* = 7; core points only), with identical *g*_*NB*_(0) settings. The DICE coefficient was defined as follows for the two channels (*A* and *B*):

35 $${DICE}(A,\,B)=\frac{2{|A}\wedge B|}{\left|A\right|+\left|B\right|\,}$$

The resulting unitless DICE coefficient value range was [0,1], where 0 indicates disjointed masks, and 1 indicates identical masks (perfect colocalisation of pixels for background and newly inserted LPS clusters). If $\left|A\right|+\left|B\right|=0$ (no clusters detected in either channel) then the DICE coefficient was undefined and recorded as *NaN* and excluded from downstream mean calculations. DICE is sensitive to the spatial overlap of DBSCAN-defined cluster cores (i.e. the densest cluster pixels) and penalises cases where clusters are present in one channel but absent in the other channel for the same region. We reported DICE values (mean ± S.E.M.) calculated for *N* = 5 independent runs per model at each time point.

### Reporting summary

Further information on research design is available in the Nature Portfolio Reporting Summary linked to this article.

## Supplementary information

Supplementary Information

Description of Additional Supplementary Files

Supplementary Movie 1

Supplementary Movie 2

Supplementary Movie 3

Supplementary Movie 4

Reporting Summary

Transparent Peer Review file

## Source data

Source Data

## Data Availability

Unless otherwise stated, all data supporting the results of this study can be found in the article, supplementary and source data files. The accompanying Figure Source Data and Analysis Workbook contains numerical data for the main and Supplementary Figs., single cell and replicate biochemical data, analysis and simulation input and output files, and uncropped gel and blot images. Time-lapse imaging data are provided as Supplementary Movies. Requests for additional information or materials should be directed to the corresponding authors. Source data are provided with this paper.

## Code availability

The custom MATLAB and Python codes used for dSTORM image analysis, data interrogation and particle-based simulations are available in the Persistent_LPS_insertion_OM_OMVs GitHub repository at https://github.com/joenabarro-OM/Persistent_LPS_insertion_OM_OMVs/releases/tag/v1.0.2. This version, release v1.0.2, has been permanently archived on Zenodo at https://doi.org/10.5281/zenodo.21326870^105^. The code is released under the MIT License.
