# Supplementary material for: Lipopolysaccharide insertion and vesicle mediated turnover drive growth independent outer membrane adaptation in Escherichia coli
Source: Nat Commun. 2026 Aug 21;17:9983. doi: 10.1038/s41467-026-76886-6 (PMC13590524; doi:10.1038/s41467-026-76886-6)
Supplement: Supplementary file 1 [file 41467_2026_76886_MOESM1_ESM.pdf]

Lipopolysaccharide insertion and vesicle mediated turnover drive growth independent outer membrane adaptation in *Escherichia coli*

Supplementary Information

Author list

Joe Nabarro[1,2,*], Natasha E. Hatton[1], Bartosz L. Kowalski[1], Christopher D. Spicer[1], Dmitri O. Pushkin[3], Martin A. Fascione[1,*], and Christoph G. Baumann[2,*]

Affiliations

[1] Department of Chemistry, University of York, York YO10 5DD, United Kingdom

[2] Department of Biology, University of York, York YO10 5DD, United Kingdom

[3] Department of Mathematics, University of York, York YO10 5DD, United Kingdom

[*] Co-corresponding authors

Corresponding author list and correspondence details

Dr. Joe Nabarro

joe.nabarro@york.ac.uk

Dr. Martin A. Fascione

martin.fascione@york.ac.uk

Dr. Christoph G. Baumann

christoph.baumann@york.ac.uk

Table of Contents

Item	Description	Page
Supplementary Figure 1	Two-colour validation of site-specific LptD* labelling and colocalisation with newly inserted LPS, including reciprocal dye labelling, LptD structure, SDS-PAGE and cross-correlation analyses	4
Supplementary Figure 2	Comparison of Kdo-N_3 and Kdo-alkyne incorporation, FRAP controls, fluorescent TSDS-PAGE, reciprocal dye controls and two-colour DBSCAN outputs	5-6
Supplementary Figure 3	Biological replicate trajectories for background LPS loss, new LPS insertion and culture growth during the 210 min pulse-chase	7
Supplementary Figure 4	Spatial and temporal DBSCAN analysis of background and newly inserted LPS clusters across polar and mid-cell OM regions	8
Supplementary Figure 5	Cell length changes and division during live-cell 3D-SIM^2 imaging	9
Supplementary Figure 6	Replicate biochemical analyses supporting OMV-mediated clearance of background LPS, including uncropped TSDS-PAGE scans	10
Supplementary Figure 7	Kdo-N_3 incorporation during exponential and stationary phase growth	11
Supplementary Figure 8	NTA characterisation of OMV size after 0.22 μm filtration	12
Supplementary Figure 9	Extended simulation snapshots comparing insertion trapping and phase separation models	13
Supplementary Figure 10	Exponential fits to background- and newly inserted-LPS DBSCAN cluster statistics	14
Supplementary Figure 11	Cell dimensions, viability, OMV accumulation and OMV production per viable cell during stationary phase	15
Supplementary Figure 12	Live-cell 3D-SIM^2 and dSTORM examples of background LPS-enriched OMV-like structures	16-18
Supplementary Figure 13	Per-cell quantification of background and newly inserted LPS enrichment or depletion in OMV nucleation regions	19
Supplementary Figure 14	Biochemical fractionation excluding soluble intracellular accumulation of labelled background LPS	20
Supplementary Figure 15	Kdo-analogue and fluorophore conjugation specificity controls	21-22
Supplementary Figure 16	NTA-calibrated modelling of OMV-mediated background LPS turnover beyond dilution by cell elongation and division	23
Supplementary Figure 17	NTA characterisation of OMV size after 0.45 μm filtration	24
Supplementary Figure 18	Protein and LPS composition of OM and OMV fractions, including Coomassie and silver-stained gels	25
Supplementary Figure 19	Extended live-cell 3D-SIM^2 time courses visualising gradual loss of background LPS fluorescence	26-29
Supplementary Table 1	Bacterial strains	30
Supplementary Table 2	DNA primer sequences	30

Item	Description	Page
Supplementary Table 3	Supplemented M9 chemically defined medium composition	31
Supplementary Table 4	Alkyne-functionalised fluorophore concentrations for CuAAC	31
Supplementary Table 5	AF488-DBCO concentration for SPAAC	31
Supplementary Table 6	LptD* and newly inserted LPS dual-colour labelling orders	31
Supplementary Table 7	Glucose oxidase / catalase oxygen-scavenging buffer	31
Supplementary Table 8	Zeiss Elyra 7 dSTORM acquisition settings	32
Supplementary Table 9	Typical dSTORM localisation filtering settings	32
Supplementary Table 10	Mean LptD*–new LPS cross-correlation data	32
Supplementary Table 11	Regional LptD*–new LPS PCC statistical comparisons	33
Supplementary Table 12	Background and new LPS reciprocal dye-labelling orders	33
Supplementary Table 13	OM coverage and cluster area intersection for background and newly inserted LPS	33
Supplementary Table 14	4× TSDS-PAGE loading-buffer composition	34
Supplementary Table 15	AF488 dSTORM filtering settings	34
Supplementary Table 16	Biological replicate background LPS turnover rate values	34
Supplementary Table 17	Biological replicate new LPS insertion rate values	35
Supplementary Table 18	Background LPS DBSCAN summary statistics	35
Supplementary Table 19	Newly inserted LPS DBSCAN summary statistics	35
Supplementary Table 20	Standard and extended duration 3D-SIM[2] settings	36
Supplementary Table 21	OMV lysis buffer composition	36
Supplementary Table 22	OD_{600} values during the pulse-chase time course	36
Supplementary Table 23	Viable cell concentrations and CFU / OD_{600} values	37
Supplementary Table 24	Per-cell background and new LPS enrichment in OMV nucleation regions	38
Supplementary References	Literature sources cited in the supplementary information	39

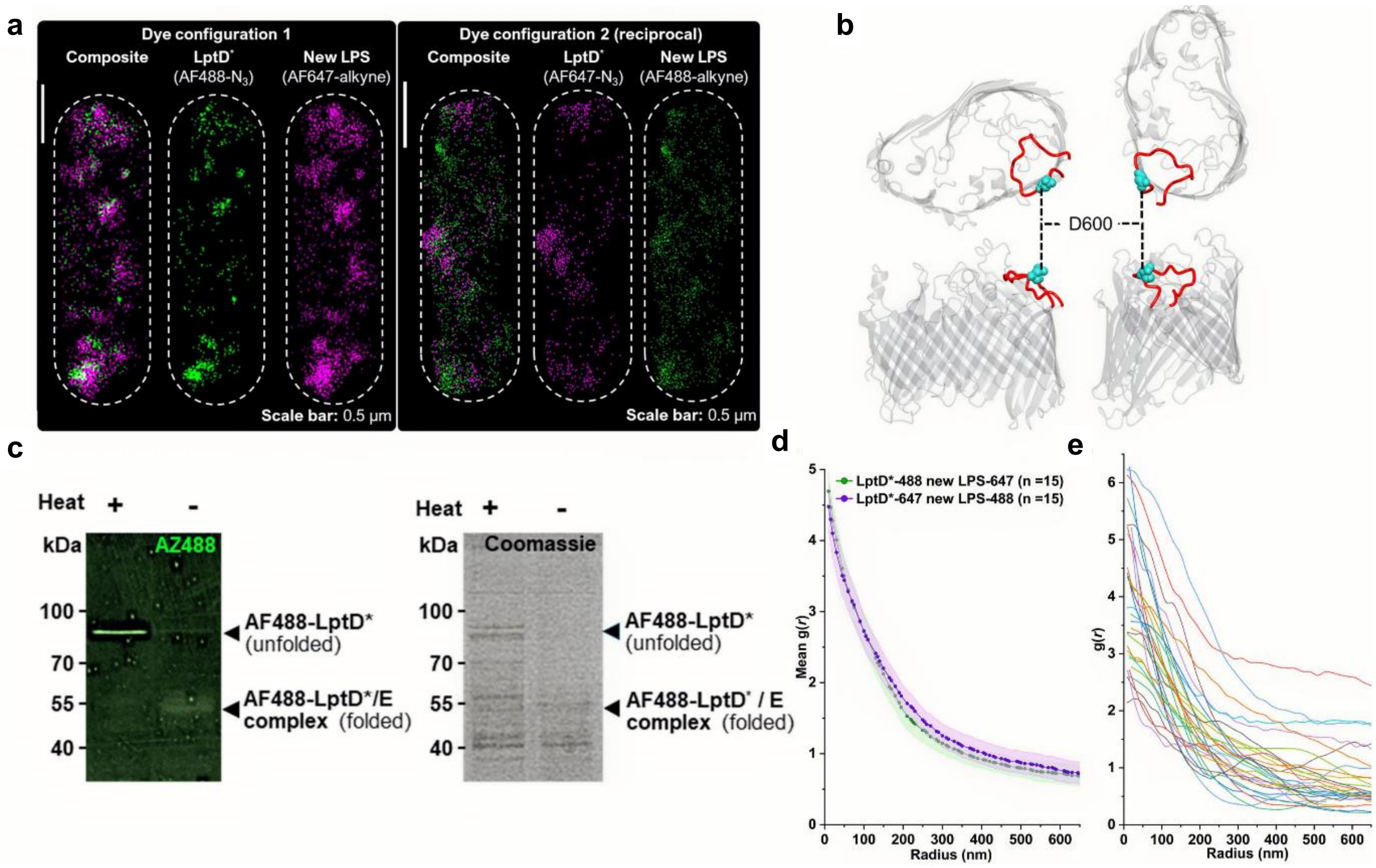

Supplementary Figure 1. Two colour labelling confirms site-specific incorporation and co-localisation of functional LptD* and newly inserted LPS in the OM of *E. coli* K12.

a. **Reciprocal dye labelling confirms robust LptD–newly inserted LPS puncta independent of fluorophore identity.** Two-colour dSTORM images of *E. coli* outer membranes labelled for recombinant LptD* and newly inserted LPS 30 minutes after co-addition of N-propargyl-L-lysine and Kdo-N_3. LptD* was mutated with an amber stop codon replacing D600 in extracellular loop 9 and expressed via genetic code expansion, enabling site-specific conjugation to an azide(N_3)-functionalised fluorophore via CuAAC. Newly inserted LPS was metabolically labelled with Kdo-N_3, enabling site-specific conjugation to an alkyne-functionalised fluorophore via CuAAC. In one dye configuration, LptD was labelled with AF488-N_3 and newly inserted LPS with AF647-alkyne ***(left)***; in the reciprocal configuration, LptD was labelled with AF647-N_3 and newly inserted LPS with AF488-alkyne ***(right)*** (dye–target assignments are indicated above each panel). Discrete, co-localised puncta are observed across the OM, independent of dye combination**.** **Scale bars:** 0.5 μm.
b. **LptD crystal structure (PDB: 4RHB) highlighting loop 9 (red) and D600 mutation site used for ncAA incorporation.**
c. **In-gel AF488 fluorescence (*left*) and Coomassie-stained (*right*) SDS-PAGE analysis of cell lysates containing AF488-labelled LptD*.** Fluorescent LptD* and native LptD*/LptE complex were visualised in unheated samples (–), while heat-denatured samples (+) show dissociated LptD* with reduced mobility, confirming that ncAA labelling does not disrupt LptD*/LptE complex formation.
d. **Mean cross-correlation profiles for LptD*–AF488 / new LPS-AF647 (n = 15 cells) and LptD*-AF647 / new LPS–AF488 (n = 15 cells) reciprocal dye configurations show near identical, strong sub-diffraction co-localisation, indicating spatial correlation was independent of dye selection.**
e. **Single-cell cross-correlation profiles (n = 30 cells) showing consistent strong sub-diffraction co-localisation between LptD* and newly inserted LPS across the population of screened *E. coli* OMs.**

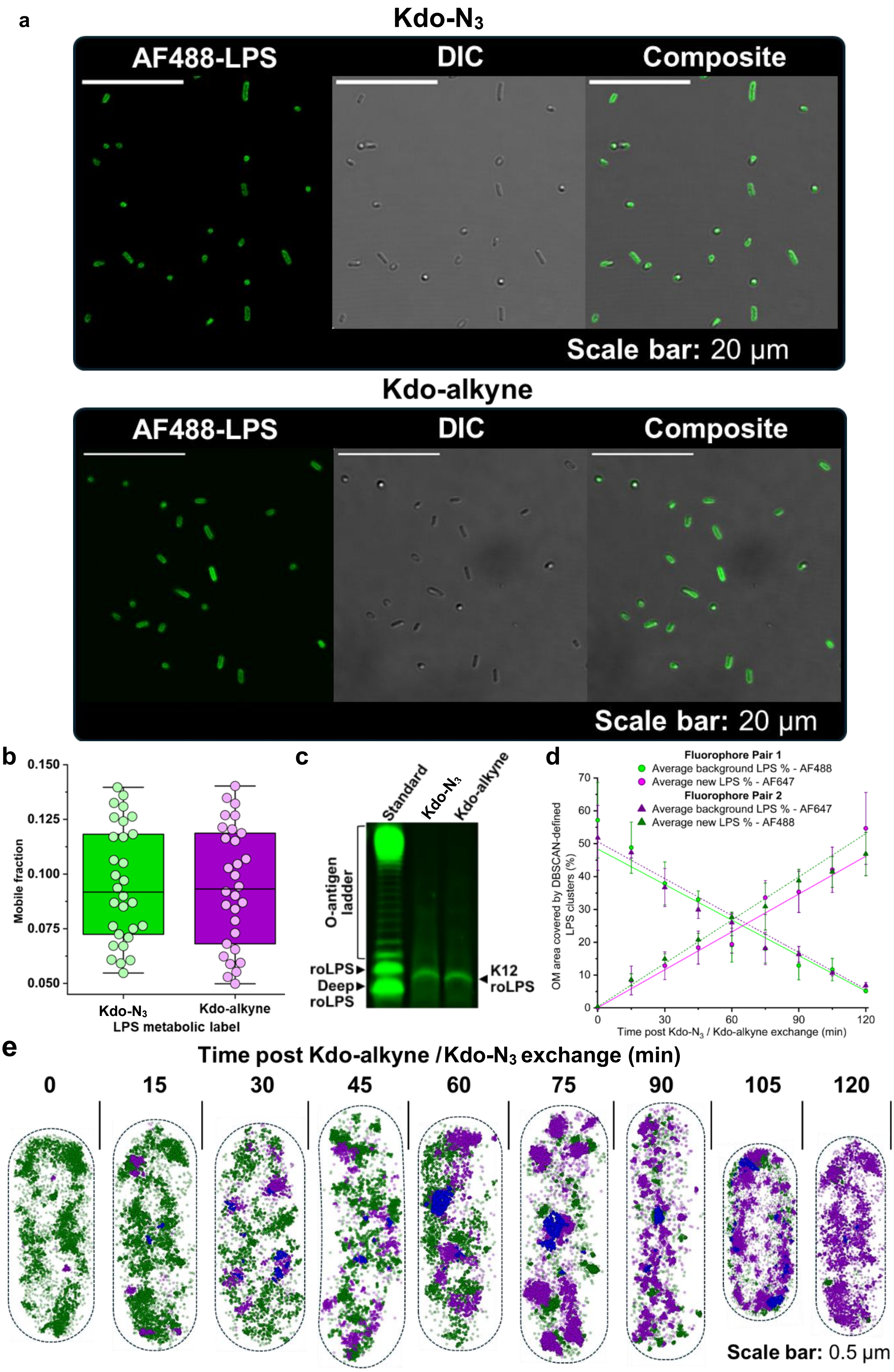
a
Kdo-N3
AF488-LPS
DIC
Composite
Scale bar: 20 µm
Kdo-alkyne
AF488-LPS
DIC
Composite
Scale bar: 20 µm
b
Mobile fraction
0.150
0.125
0.100
0.075
0.050
Kdo-N3
Kdo-alkyne
LPS metabolic label
c
Standard
Kdo-N3
Kdo-alkyne
O-antigen ladder
roLPS
Deep roLPS
K12 roLPS
d
Fluorophore Pair 1
Average background LPS % - AF488
Average new LPS % - AF647
Fluorophore Pair 2
Average background LPS % - AF647
Average new LPS % - AF488
OM area covered by DBSCAN-defined LPS clusters (%)
Time post Kdo-N3 / Kdo-alkyne exchange (min)
e
Time post Kdo-alkyne / Kdo-N3 exchange (min)
0
15
30
45
60
75
90
105
120
Scale bar: 0.5 µm

Supplementary Figure 2. Kdo-alkyne and Kdo-N_3 analogues were incorporated into the LPS of *E. coli* BW25113 cells at equivalent levels. Representative DBSCAN outputs derived from Kdo-alkyne / Kdo-N_3 pulse–chase experiments in combination with two-colour 2D dSTORM super-resolution imaging demonstrate persistent spatial separation of newly inserted and background LPS-rich regions in the OM.

- **a. Confocal microscopy of *E. coli* BW25113 cells metabolically labelled in vivo with either Kdo-N_3 (top row) or Kdo-alkyne (bottom row) reveals robust, equivalent levels of LPS fluorescent labelling achieved via Cu^+-catalysed azide(-N_3)-alkyne cycloaddition.** Representative widefield (AF488 fluorescence – left), brightfield (middle), and composite (right) images are shown. **Scale bars:** 20 µm.
- **b. Quantitative FRAP analysis shows no significant differences in lateral mobility of LPS labelled with Kdo-N_3 (green, n = 30 cells, median mobile fraction = 0.092) or Kdo-alkyne (purple, n = 30 cells, median mobile fraction = 0.0877).** Mobile fraction distributions are statistically indistinguishable (two-tailed Mann–Whitney U = 461, p = 0.877), indicating that incorporation of the functionalised Kdo-analogues does not affect LPS lateral mobility in vivo. Each data point represents the mobile fraction derived from a FRAP experiment carried out on an individual cell. Boxes show interquartile ranges and midline denotes median mobile fraction value.
- **c. Fluorescent TSDS-PAGE analysis of AF488-labelled LPS confirms comparable click labelling levels of Kdo-N_3 (lane 2) and Kdo-alkyne (lane 3).** A commercially purchased O55:B5 AF488-LPS conjugate standard (lane 1) served as a molecular weight reference.
  roLPS: Rough LPS; **deep roLPS:** Deep rough LPS; **K12 roLPS:** rough LPS extracted from Kdo-analogue labelled *E. coli* K-12.
- **d. Dye selection does not influence outer-membrane coverage by background and newly inserted LPS clusters in *E. coli* BW25113 cells**. Quantification of OM coverage by DBSCAN-defined background and newly inserted LPS clusters for both dye orientations (AF488-background/AF647-newly inserted and AF647-background/AF488-newly inserted). Values represent mean ± S.E.M. from individual cells ($n \geq 5$ cells per time point per dye orientation; $n \geq 10$ cells per time point when dye orientations are combined). Near-identical linear trends across dye orientations indicate reciprocal loss of labelled background LPS and accumulation of labelled newly inserted LPS with rates that are not measurably affected by fluorophore assignment.
- **e. Representative DBSCAN clustering outputs from Kdo-alkyne / Kdo-N_3 pulse-chase experiments visualised via two-colour dSTORM showing spatial organisation of background (green) and newly inserted (purple) LPS in the OM of *E. coli* cells over a 120-minute monitoring period post Kdo-analogue exchange**. Background and newly inserted LPS-rich regions remain largely discrete across time points, with only limited cluster-area intersection (blue; defined as the geometric intersection of independently segmented DBSCAN cluster areas). This supports a model of minimal mixing between old and newly synthesised LPS domains at the effective nanoscale resolution of the analysis.

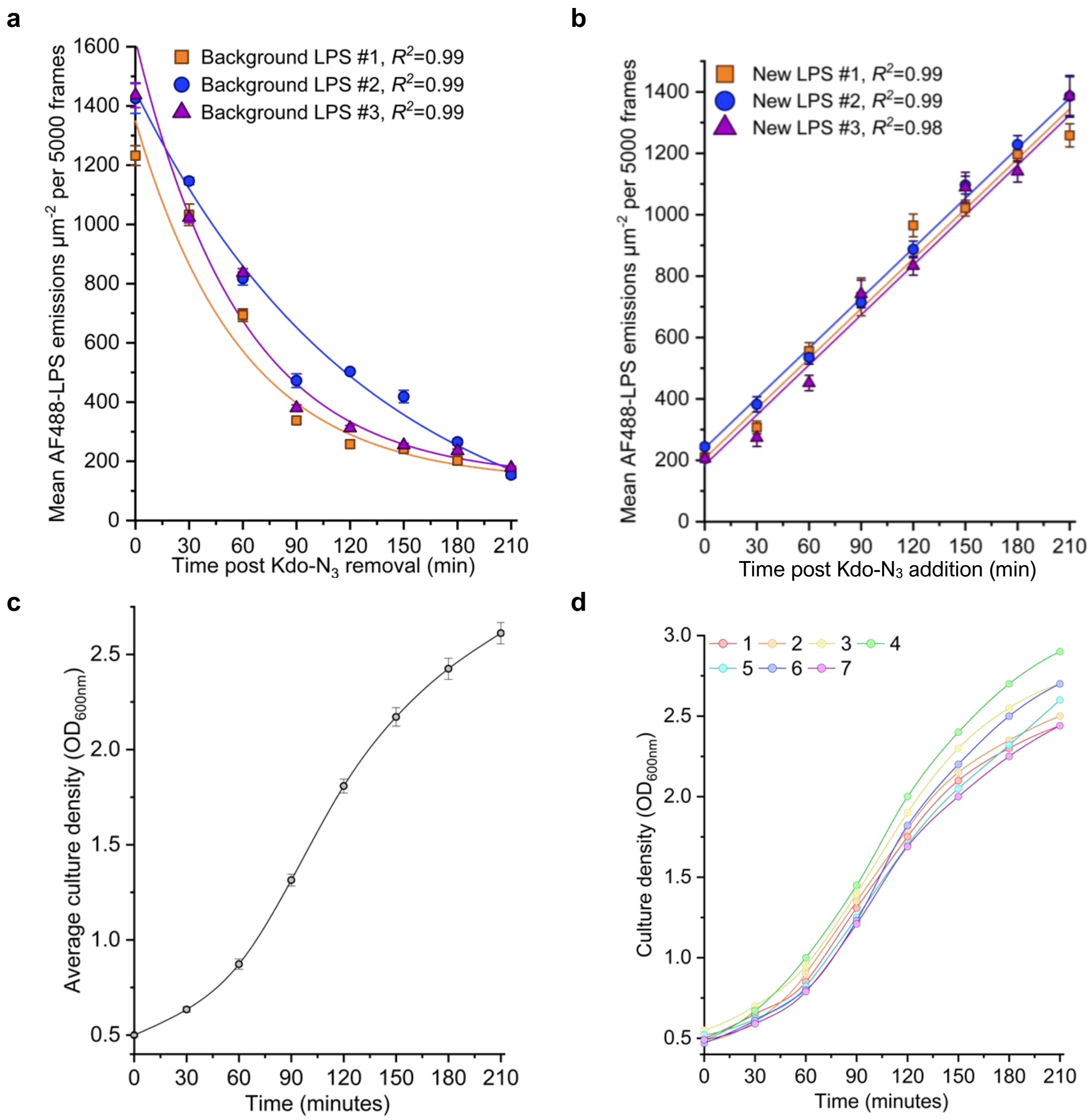

Supplementary Figure 3. Quantitative analysis of background LPS loss, new LPS insertion, and growth rate during individual 210-minute Kdo-N_3 / native Kdo pulse–chase dSTORM experimental replicates.

a. Per-cell background LPS abundance over time for each of three independent *E. coli* BW25113 replicates (N = 3 biological replicates; n ≥ 10 cells per time point). The number of discrete AF488–LPS localisations per outer membrane (OM), derived from dSTORM, is used as a proxy for background LPS content. Loss of background LPS continues throughout the 210-minute chase, even as cell division slows, and follows an exponential decay ($R^2 \geq 0.99$).

b. Per-cell abundance of newly inserted LPS over time for each of three independent *E. coli* BW25113 replicates (N = 3 biological replicates; n ≥ 10 cells per time point). Newly inserted LPS was labelled using native Kdo / Kdo–N_3 metabolic pulse–chase and detected via AF488 fluorescence. Insertion proceeds linearly ($R^2 \geq 0.98$) over the full chase period, despite the onset of stationary phase between 120- and 150-minute time points.

c. Mean growth curve (N = 7 biological replicates) showing culture OD_{600} over the 210-minute chase periods. A decrease in growth rate is evident beyond 120-min time point, consistent with transition from late exponential to stationary phase.

Individual OD_{600} growth curves for all biological replicates used for the mean growth curve in panel C. The source data for these growth curves are provided in Supplementary Table 22.

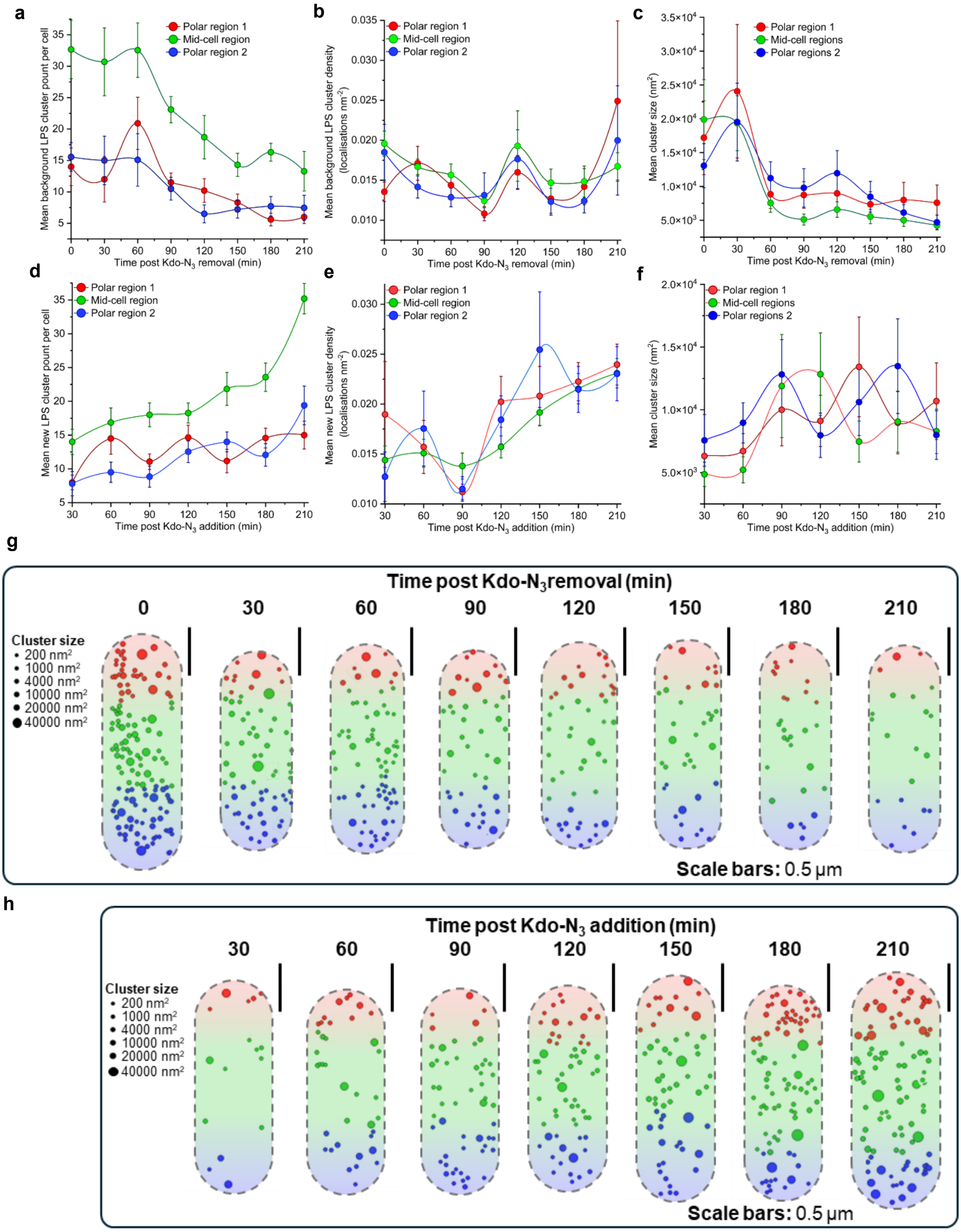

Supplementary Figure 4. Spatio-temporal analysis of LPS-rich clusters across outer membrane regions of *E. coli* revealed stable organisation of LPS-rich domains.

a-c. Mean number, density, and size of background (AF488-labelled) LPS-rich clusters detected in polar (red and blue) vs. mid-cell (green) regions over time following Kdo-N_3 labelling.

d-f. Mean number, density, and size of newly inserted (AF488-labelled) LPS-rich clusters across the same regions over time following Kdo-N_3 pulse-labelling. For all plots, n ≥ 10 cells per time point.

g, h. Representative single-cell DBSCAN cluster maps visualised from dSTORM data for individual *E. coli* BW25113 cells. Clusters are colour-coded by OM region (**polar region 1,** blue; **mid-cell:** green **polar region 2,** red) and scaled globally by area. Images represent selected time points from background LPS turnover **(panel G)** and new LPS insertion **(panel H)** experiments. **Scale bars:** 0.5 µm

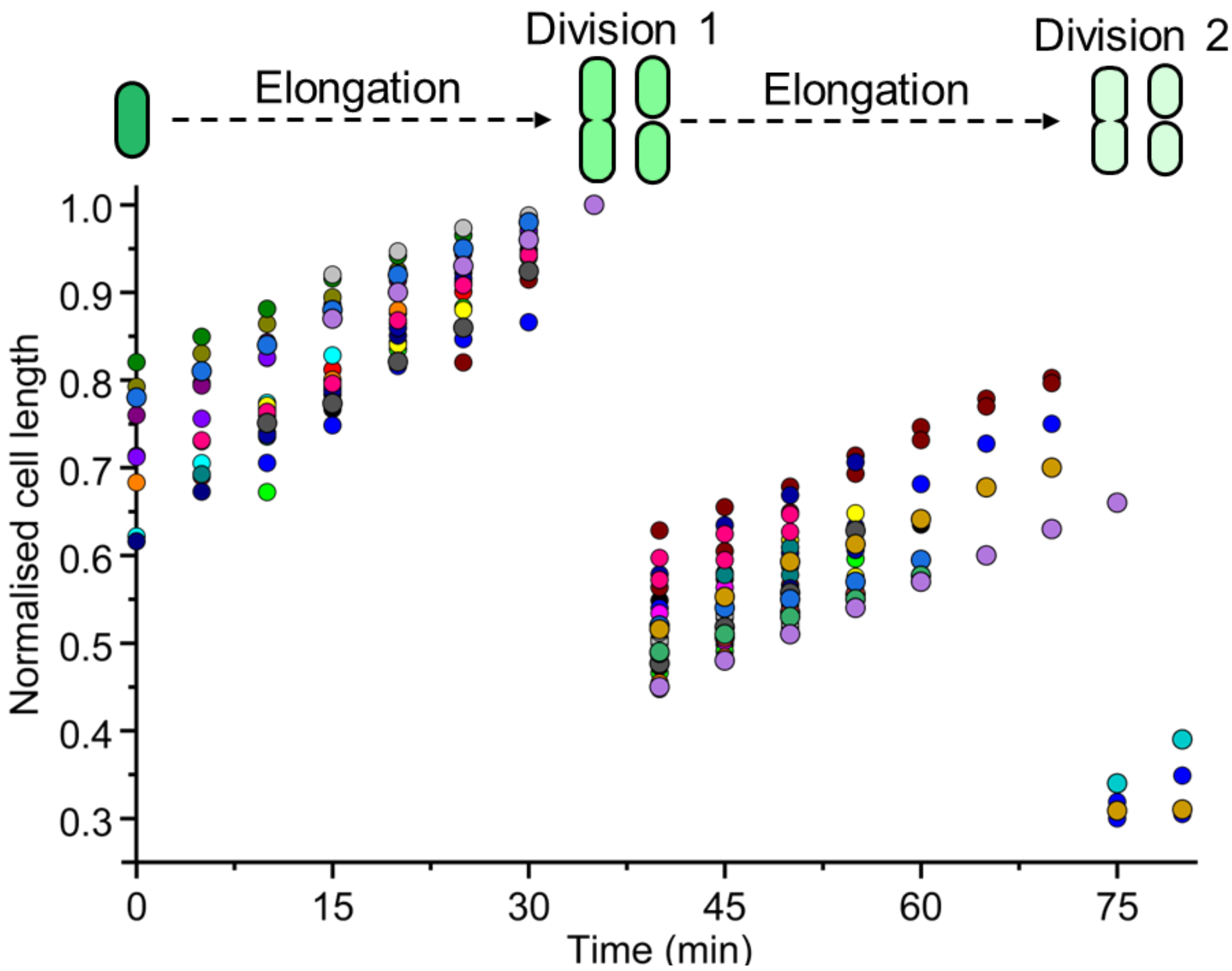

Supplementary Figure 5. Graph showing normalised changes in cell lengths (n = 44 cells) observed over the course of the 3D SIM[2] experiment. Cell lengths were normalised for each individual cell with the maximum length set to 1. An average doubling time of approximately 35 minutes was determined over the course of the time-lapse imaging experiment which matches the value measured in bulk bacterial cultures. This confirms cell elongation and division were not affected by Kdo-N_3 incorporation, SPAAC conjugation of DBCO-AF488, or exposure to the 488 nm excitation laser.

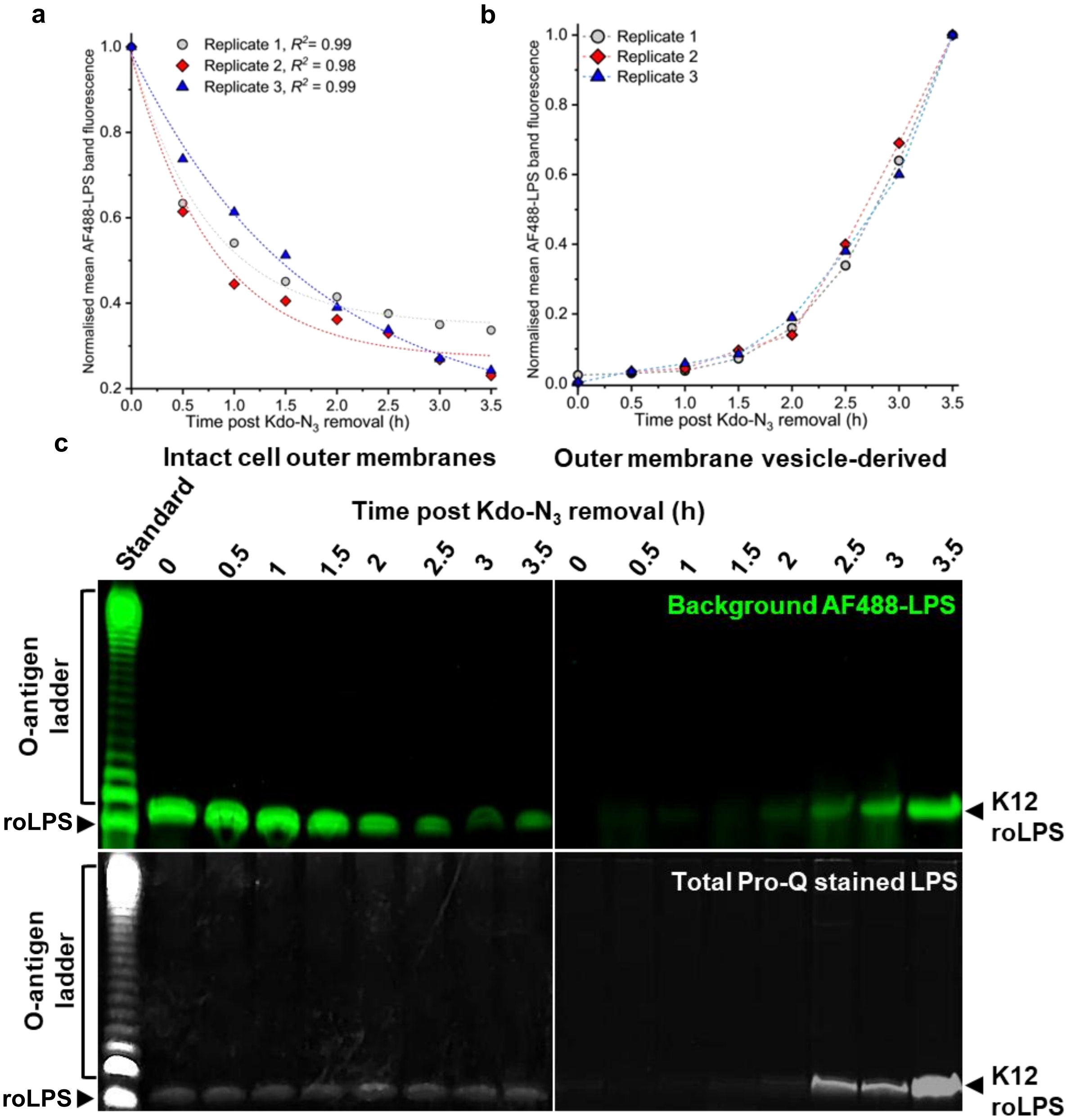

Supplementary Figure 6. Outer membrane vesicles (OMVs) mediate clearance of background (pre-existing) LPS from the OM in non-growing, stationary phase *E. coli* BW25113 cells.

a, b. Quantification of background AF488-labelled LPS over time in **(A)** intact-cell OM fractions and **(B)** OMV-derived fractions for individual biological replicates. For intact-cell OM fractions, lanes contain a standardised mass of total LPS so that each lane corresponds to the LPS recovered from an equivalent number of cells at that time point. For OMV fractions, lanes contain the complete LPS extracted from the cell-free supernatant collected at each time point (standard culture volumes). Replicate datasets are well described by single-exponential functions ($R^2 \geq 0.98$), consistent with analogue partitioning of background LPS as expected during exponential growth but that continues into stationary growth phase.

c. TSDS-PAGE analysis showing decrease in background AF488-LPS (top) concentration in intact-cell OM fractions (left) and reciprocal increase in background AF488-LPS in OMV-derived fractions (right) with associated total Pro-Q stained total LPS loading controls (bottom). A commercially purchased O55:B5 AF488-LPS conjugate standard (lane 1, labelled standard) served as a molecular weight reference.

roLPS: Rough LPS. **K12 roLPS:** Rough LPS extracted from *E. coli* BW25113 cells cultured in the presence of Kdo-N_3 or OMVs released by these cells and purified from cell-free culture supernatants.

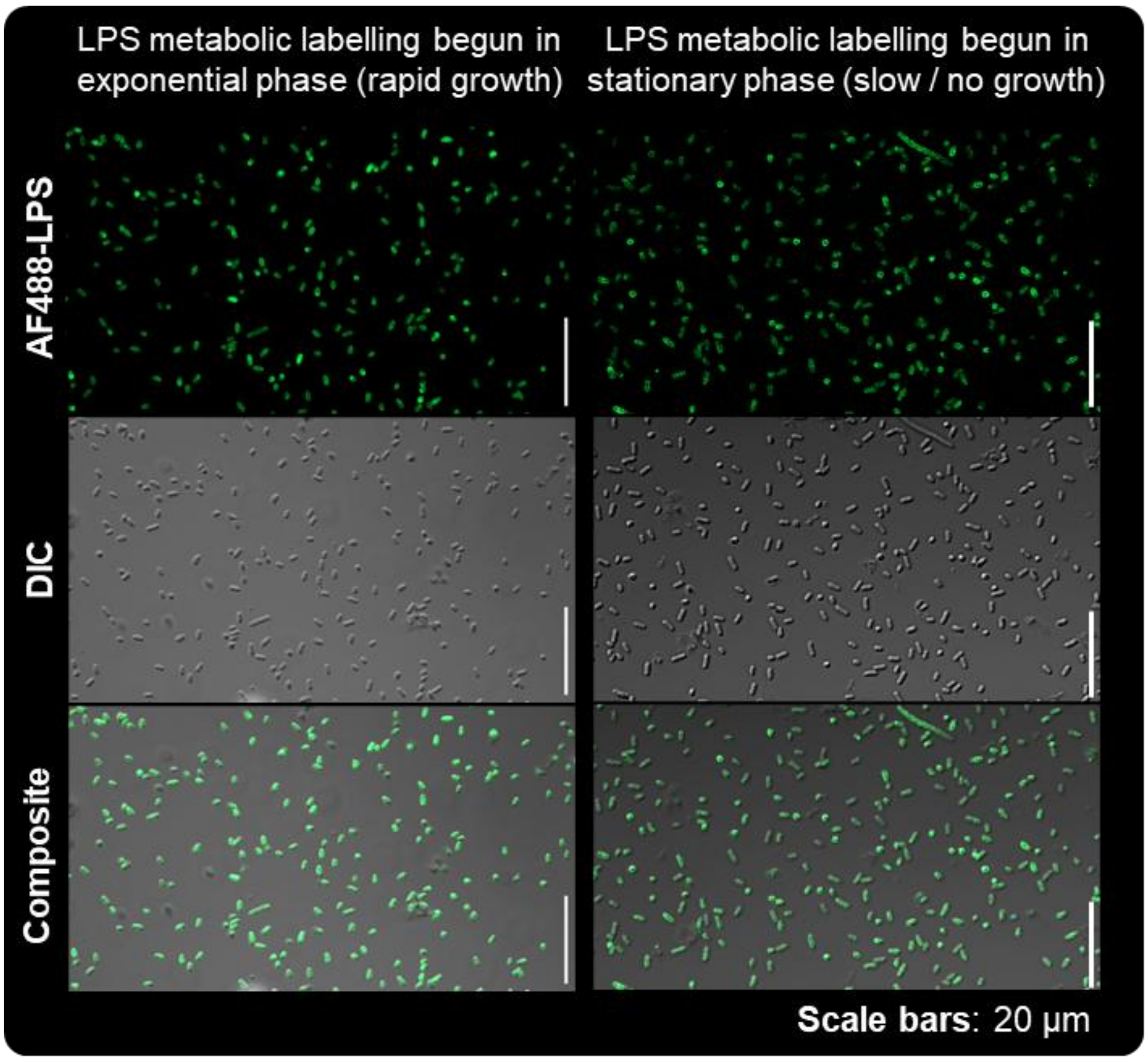

Supplementary Figure 7. Kdo-N_3 incorporation in LPS of *E. coli* BW25113 cells is independent of cell growth phase and does not require cell elongation. Equivalent levels of cell fluorescence were observed via fluorescence confocal microscopy in cells where Kdo-N_3 was added to the culture during the early stages of exponential growth (OD_{600} = 0.05, left) or during early stationary phase (OD_{600} = 2.5, right). After incubation for 12 h in M9 CDM charged with 4 mM Kdo-N_3, the LPS was labelled via CuAAC with AF488-alkyne. **Scale bars:** 20 µm.

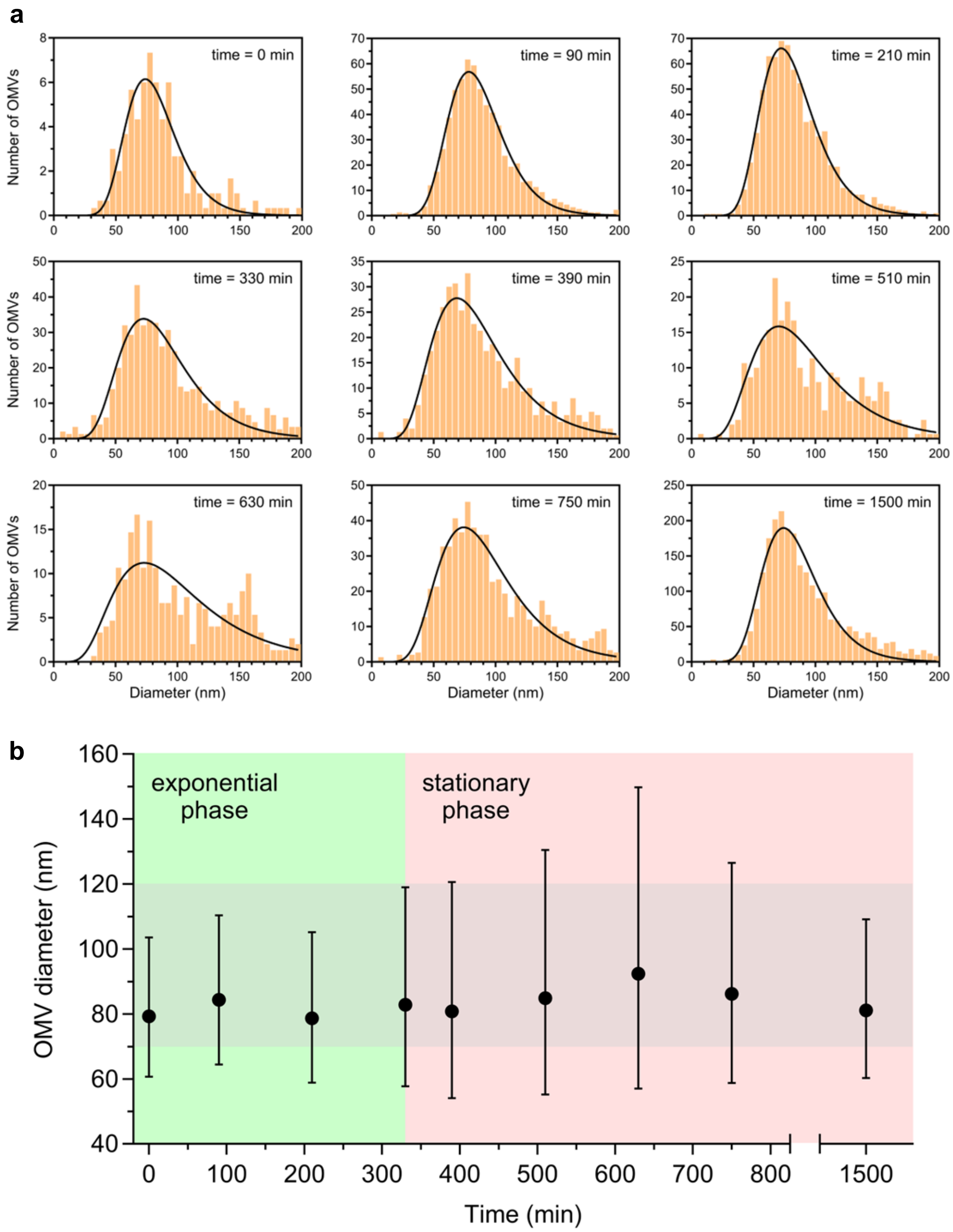

Supplementary Figure 8. Characterisation of OMVs produced by *E. coli* during exponential and stationary phases of growth in chemically defined medium (CDM) and purified using 0.22 μm pore size filtration.

a. Histograms of the diameters measured by nanoparticle tracking (NTA) for OMVs released by *E. coli* BW25113 cells cultured in M9 CDM at 37 °C for different growth times (see top right corner of each histogram for growth time). OMVs were purified by sequential filtration through 0.45 μm and 0.22 μm pore size hydrophilic syringe filters (see Methods section for description of the purification procedure). Each histogram was compiled using data from technical replicates (N = 3) done on a representative sample. The skewed size histograms at each time point were best described by a log-normal distribution function with mostly smaller vesicle sizes observed at each time point. Non-linear regression of the histograms with this distribution function yielded the following R^2 values (listed in chronological order): 0.69, 0.96, 0.96, 0.78, 0.60, 0.58, 0.50, 0.75 and 0.90.

b. The mean OMV diameters (± SD) obtained from the analysis of the size histogram for each time point. The grey shaded region indicates the predicted range of OMV diameters (d = 73-122 nm) from the OM buckling model for a range of experimentally determined OM thicknesses (= 7.5-21 nm, see Methods section for mathematical description of the OM buckling model).

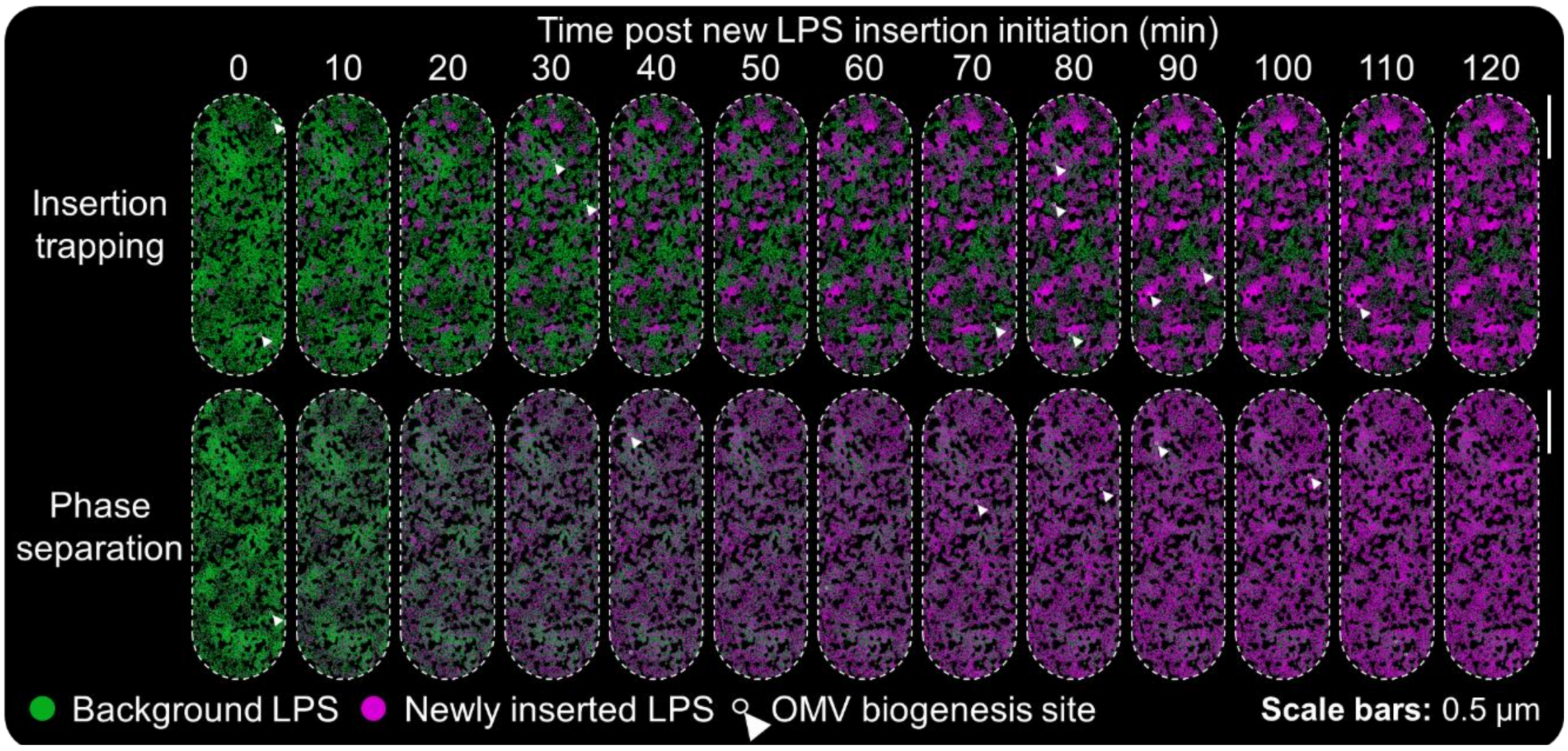

Supplementary Figure 9. Particle-based simulations of the outer leaflet in the *E. coli* OM for two different organisation models; extended snapshot sequences (0-120 min, 10 min intervals) from the two organisation models.

Insertion-trapping model (top): New LPS is delivered in bursts at discrete hotspots (LptD-like sites) and lateral motion of both new and background LPS is strongly confined, resulting in new patches remaining localised whilst background LPS is depleted locally by OMV formation.

Phase separation model (bottom): A short-range like–like coupling constant favours LPS-LPS contacts irrespective of old or new LPS type and drives domain growth under otherwise identical LPS turnover rates. This short-range favourable interaction tests whether old-new LPS co-clustering could reproduce the experimental observations. **Magenta:** Newly inserted LPS. **Green:** Background LPS. **Black / vacant spaces:** LPS-inaccessible OMP-occupied regions (~25% area). **White arrowhead / circles:** OMV biogenesis events arising in LPS-dense regions according to the OMV blebbing model. **Scale bars:** 0.5 µm**.**

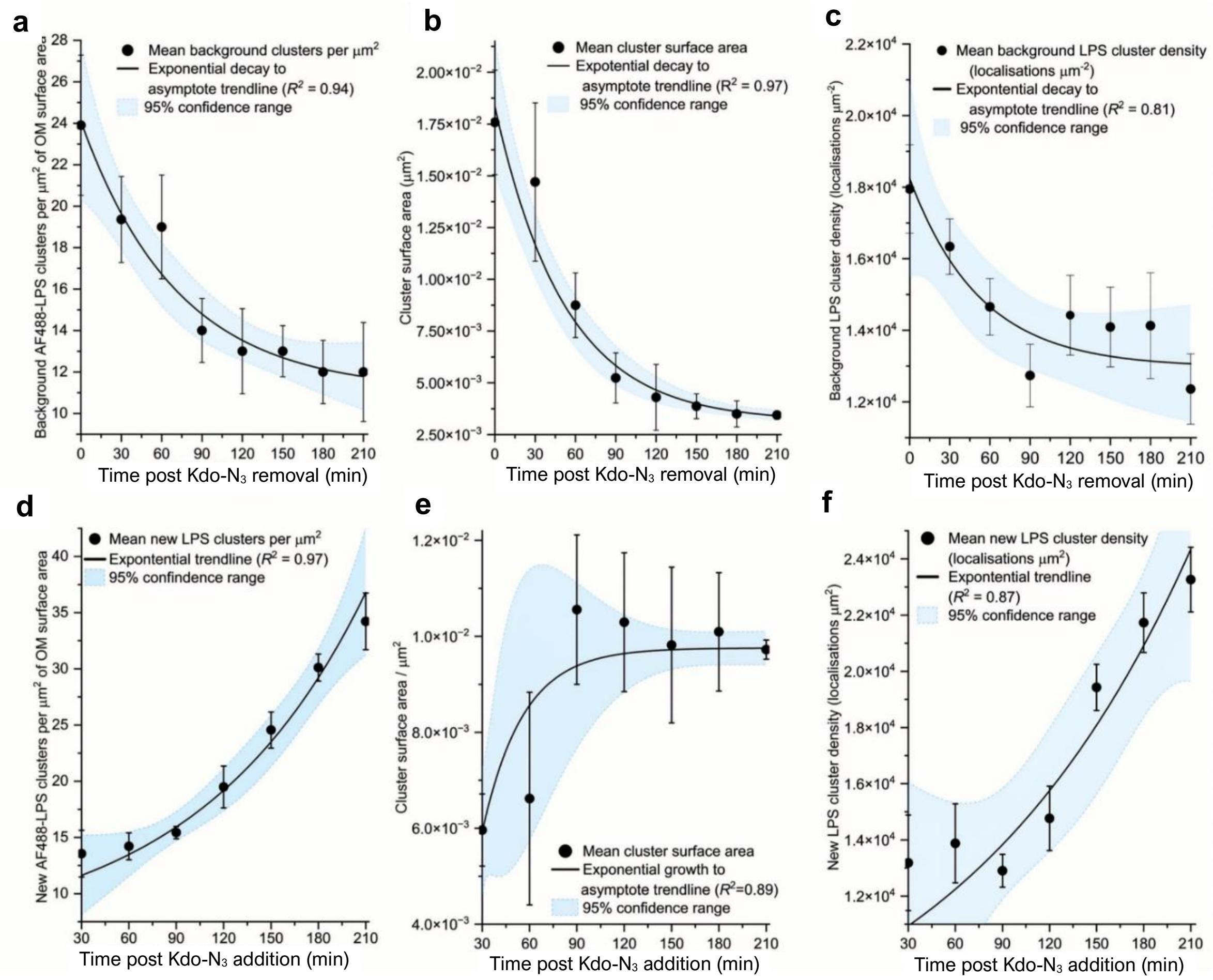

Supplementary Figure 10. Exponential fits to temporal changes in background and newly inserted LPS clusters from Kdo-N_3 pulse–chase dSTORM and DBSCAN analysis. Quantitative summary of cluster statistics underlying Figure 4b,c. Mean values ± standard errors are plotted for each time point (n ≥ 30 cells per time point, N = 3 biological replicates), with exponential fits (black lines) and 95% confidence intervals (blue shaded regions).

a-c: DBSCAN cluster analysis of background LPS (Kdo-N_3-labelled, after Kdo-N_3 removal)

- **a.** Mean number of background AF488–LPS clusters per μm² of OM surface area as a function of time after Kdo-N_3 removal. The data are well described by an exponential decay-to-asymptote fit (R^2 = 0.94).
- **b.** Mean background LPS cluster surface area as a function of time after Kdo-N_3 removal. The data are well described by an exponential decay-to-asymptote fit (R^2 = 0.97).
- **c.** Mean background LPS cluster localisation density (localisations μm^{-2}) over time, following an exponential decay-to-asymptote (R^2 = 0.81).

d-f: DBSCAN cluster analysis of newly inserted LPS (Kdo-N_3-labelled, after Kdo-N_3 addition)

- **d.** Mean number of newly inserted AF488–LPS clusters per μm² of OM surface area as a function of time after Kdo-N_3 addition, following an exponential growth trend *(R^2* = 0.97).
- **e.** Mean new LPS cluster surface area as a function of time, following an exponential growth-to-asymptote fit (R^2 = 0.89). Mean cluster size increases modestly at early time points and then plateaus despite continued increases in cluster number and total LPS coverage, arguing against domain coarsening.
- **f.** Mean new LPS cluster localisation density (localisations μm^{-2}) over time, described by an exponential growth fit (R^2 = 0.87).

Across all metrics, there is no evidence for abrupt step-like changes between time points; instead, the data follow smooth exponential trajectories consistent with gradual dilution/loss of background clusters and progressive accumulation of new clusters

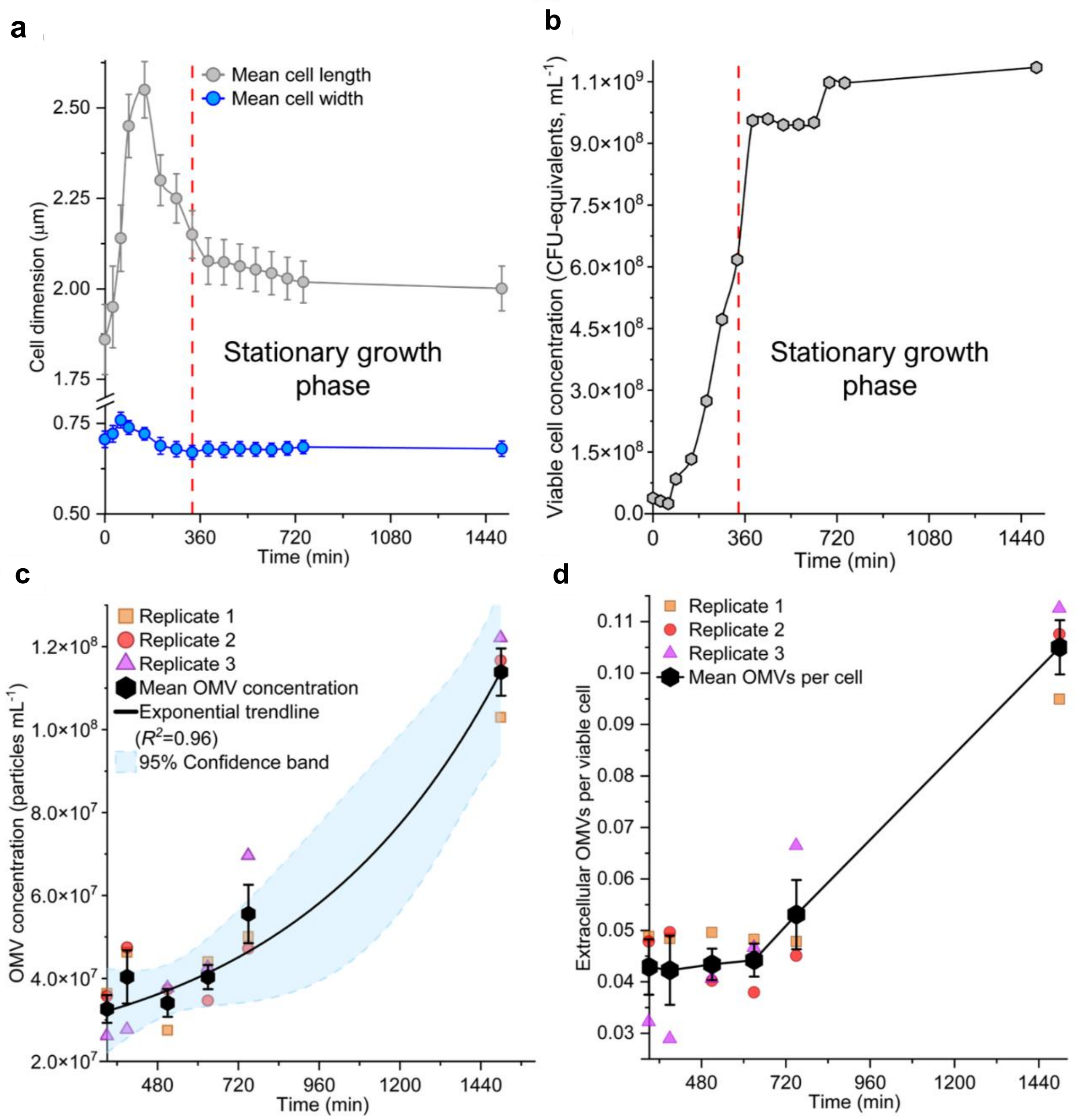

Supplementary Figure 11. Cell size, viability and OMV accumulation during stationary phase enable per-cell quantification of vesiculation, highlighting OMV production rates per cell increases significantly during stationary phase.

- **a. Mean cell length (grey) and width (blue) measured from microscopy images (n > 50 cells per time point, N = 3 biological replicates).** Points show means and error bars show standard error. The dashed red vertical line marks the onset of stationary growth phase (330 min).
- **b. Viable cell concentration (CFU equivalents mL^{-1})**. Points indicate CFU-derived estimates (used to normalise NTA particle counts) and show that viability remains high after stationary phase onset (dashed red vertical line, 330 min).
- **c. Extracellular OMV concentration measured by nanoparticle tracking analysis (NTA) during stationary phase (330 min onwards).** Coloured symbols show individual biological replicates (orange square; replicate 1, red circle; replicate 2 and magenta triangle; replicate 3) black hexagons show the mean across replicates. The black curve shows an exponential fit to the mean OMV concentration (R^2 = 0.96) with associated shaded 95% confidence band.
- **d. Extracellular OMV produced per viable cell (CFU equivalents) calculated by normalising NTA particle concentrations (c) to viable cell concentrations (b).** Coloured symbols show individual replicates, black symbols show the mean. OMV production increases significantly during late stationary phase.

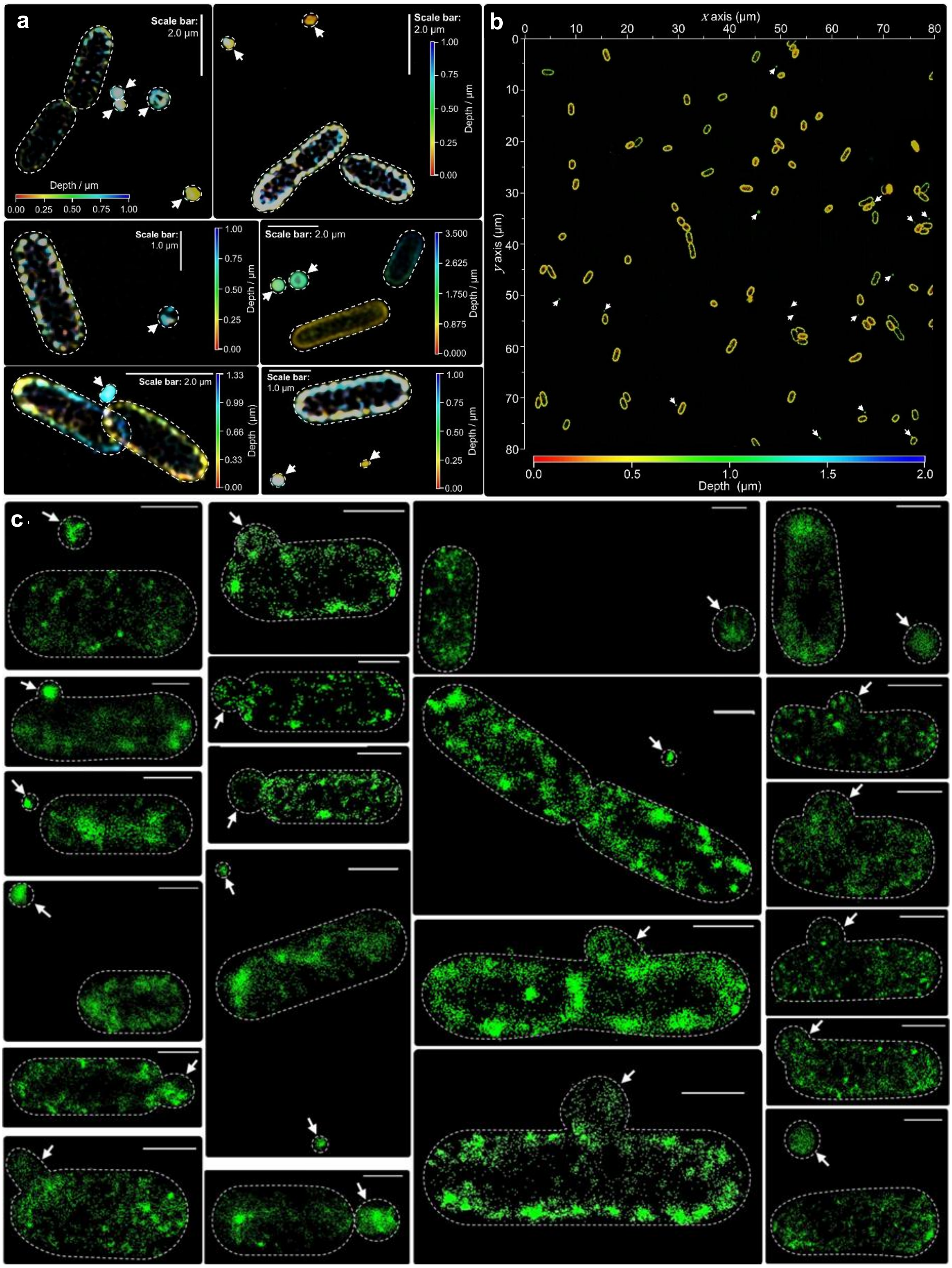
a
Scale bar: 2.0 μm
Depth / μm
0.00 0.25 0.50 0.75 1.00
Scale bar: 1.0 μm
Depth / μm
Scale bar: 2.0 μm
0.000 0.875 1.750 2.625 3.500
Depth (μm)
0.00 0.33 0.66 0.99 1.33
b
x axis (μm)
y axis (μm)
0 10 20 30 40 50 60 70 80
Depth (μm)
0.0 0.5 1.0 1.5 2.0
c

d

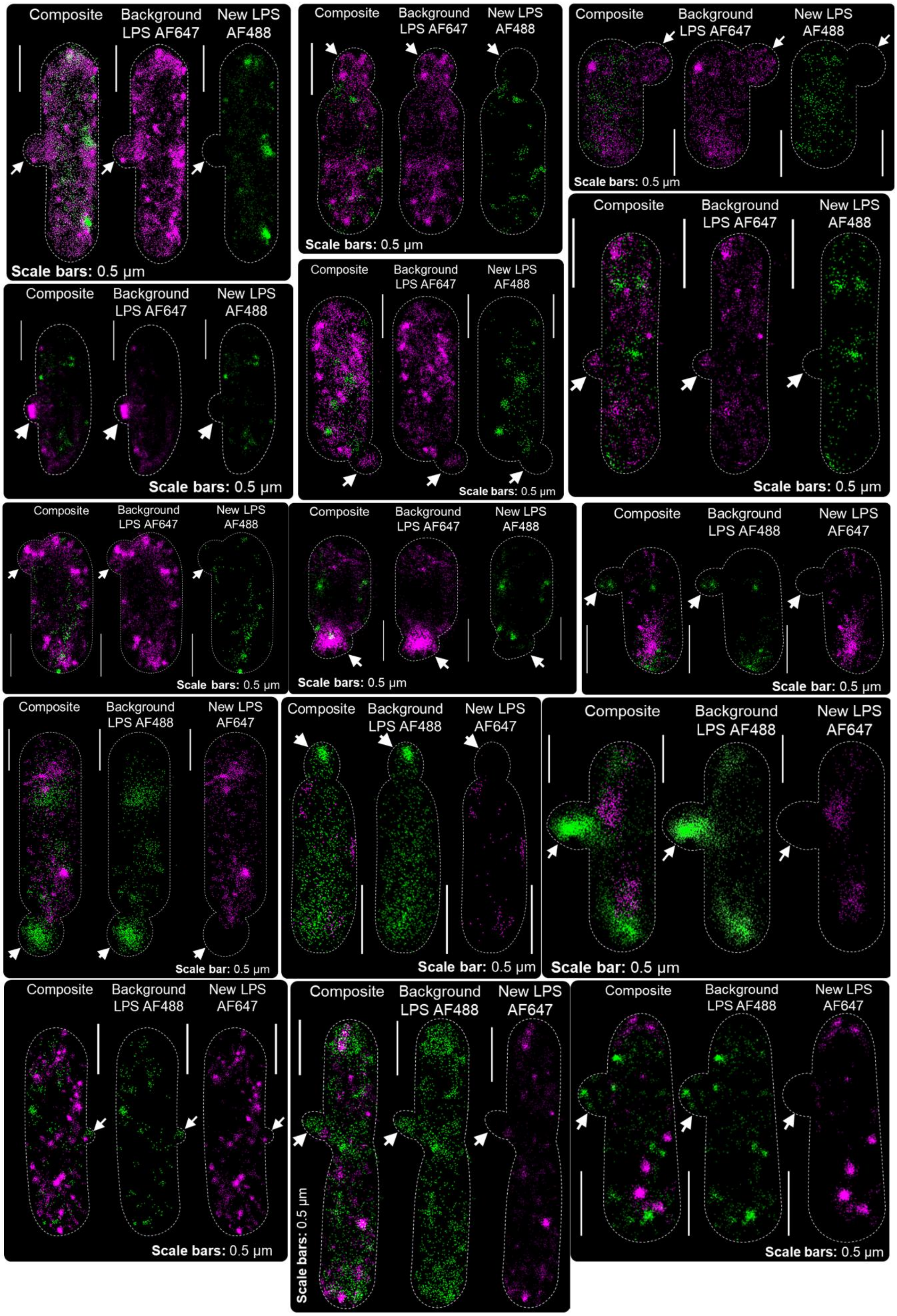
Composite
Background LPS AF647
New LPS AF488
Scale bars: 0.5 µm
Composite
Background LPS AF488
New LPS AF647
Scale bar: 0.5 µm

Supplementary Figure 12. Live-cell 3D SIM^2 and dSTORM images supporting OMV-associated shedding of background, fluorescently labelled LPS by stationary phase *E. coli* BW25113 cells.

a. Zoomed depth-coded 3D SIM^2 projections showing representative examples of live stationary-phase cells fluorescently labelled, background Kdo-N_3-LPS (labelled via SPAAC with DBCO-AF488) and imaged at 37 °C in the vicinity of OMV-like structures (white arrowheads) that exhibit substantially higher fluorescence compared to the cell OMs, consistent with enrichment of background LPS in vesicles. Depth is colour coded as indicated, scale bars as indicated.

b. Depth-coded widefield 3D SIM^2 projection of live stationary phase *E. coli* BW25113 presenting fluorescently labelled background LPS (metabolically labelled with Kdo-N_3 in vivo followed by in situ fluorescent LPS labelling with DBCO-AF488 via SPAAC). In this representative field of view 14 OMV-like structures (identified with white arrowheads) in the vicinity of 72 intact cells (0.194 visible OMV-like structures per cell). Note that this is likely a lower bound given the smallest OMVs are below the practical resolution / sensitivity limit for fluorescence detection. (Live-cell 3D SIM^2 imaging was performed across N = 3 biological replicates, n ≥ 30 cells imaged per experiment). Scale bars as indicated.

c. Single-colour dSTORM images of stationary phase cells presenting AF488-labelled, Kdo-N_3-LPS, showing OMV-like membrane protrusions and/or nearby vesicles enriched in background, labelled LPS (n = 21 cells from N = 3 biological replicates). **Scale bars:** 0.5 μm.

d. Two colour dSTORM examples from Kdo-alkyne / Kdo-N_3 pulse-chase experiments showing OMV-like protrusions/vesicles enriched in background, labelled LPS and depleted in newly inserted, labelled LPS relative to the cell OMs. Examples were observed with both dye combinations (n = 15 cells from N = 3 biological replicates). **Scale bars:** 0.5 μm.

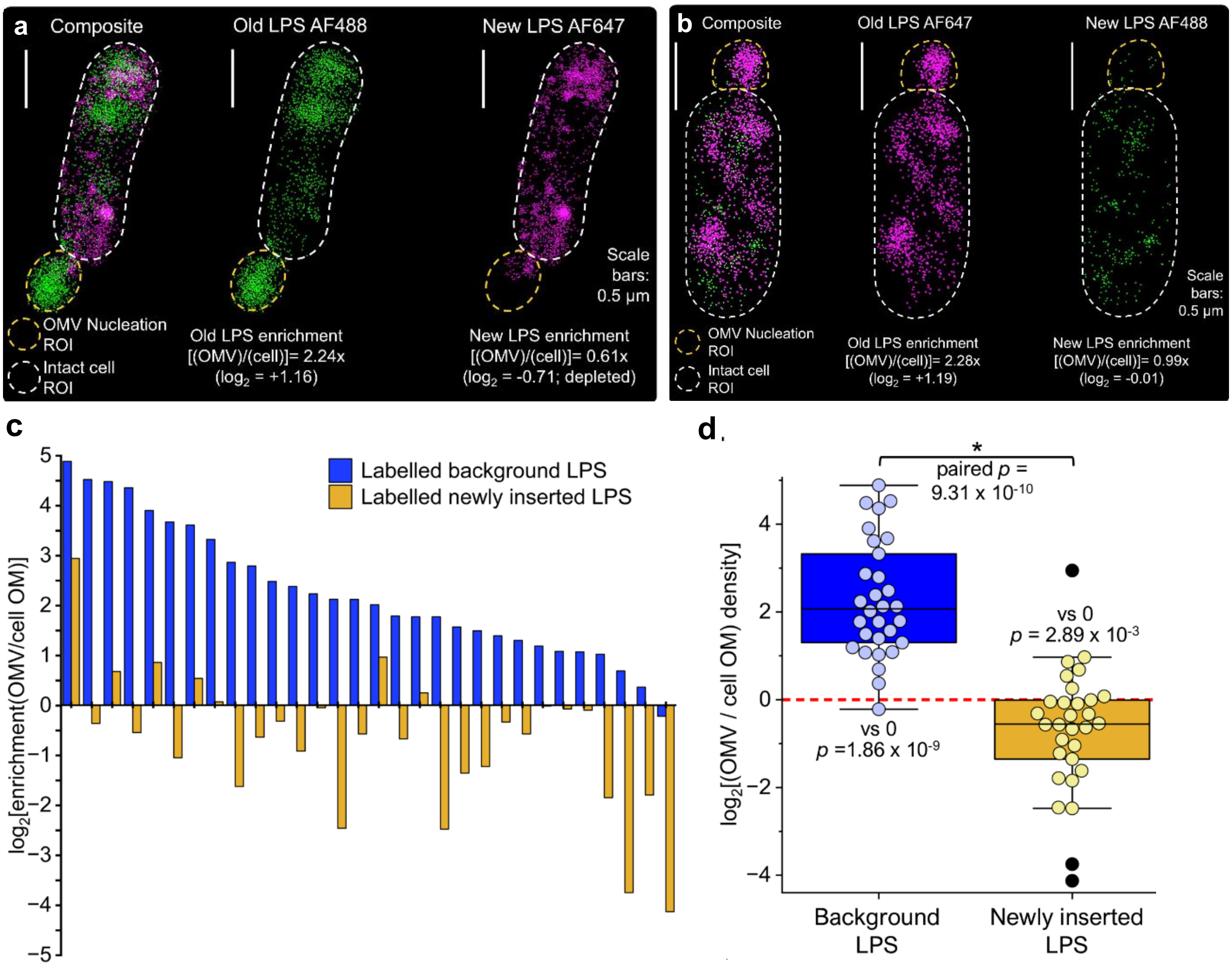

Supplementary Figure 13. Outer membrane vesicles produced from *E. coli* BW25113 cells are enriched in background, pre-existing LPS and depleted in newly inserted LPS relative to the cell outer membrane.

a, b. Representative two-colour dSTORM reconstructions of *E. coli* cells with differentially labelled background and newly inserted LPS in the process of OMV production (n = 30 cells from N = 3 biological replicates). The intact cell outer membrane ROI (white dashed outline) and OMV nucleation / bleb ROI (yellow dashed outline) were manually defined after dSTORM image reconstruction. Labelled background and newly inserted localisation densities (localisations µm^{-2}) were calculated for both ROIs and used to compute enrichment factors (S_c) for each LPS pool (old LPS and new LPS) in OMV ROIs relative to intact cell OM ROIs; $S_c = \frac{\rho_{c,OMV}}{\rho_{c,cell\ OM}}$ (reported as S_c and $\log_2(S_c)$).

Dye-swap labelling is shown in **(a)** and **(b)** to confirm that selective sorting is not dependent on fluorophore identity.

- **a. Background, pre-existing old LPS labelled with AF488 and newly inserted new LPS labelled with AF647.**
- **b. Background, pre-existing old LPS labelled with AF647 and newly inserted new LPS labelled with AF488.**
- **c. Rank-ordered per-cell enrichment values plotted as $\log_2(S)$.** Cells are sorted by background, pre-existing old labelled LPS enrichment (descending). Positive values (>0) indicate enrichment in the OMV ROI relative to intact cell outer membrane ROI; negative values (<0) indicate depletion. **Blue bars:** Background, pre-existing old LPS. **Yellow bars:** newly inserted, new LPS. n = 30 OMV-producing cells were analysed across N = 3 biological replicates.
- **d. Summary of enrichment distributions across all analysed blebbing events (n = 30 cells from N = 3 biological replicates).** Box plots show median values and interquartile ranges (IQRs); points show individual cells; red dashed line indicates $\log_2(S_c)$ = 0 (no enrichment). Background, pre-existing old labelled LPS is strongly enriched in OMV ROIs (median $\log_2(S_{old\ LPS})$ = +2.07, corresponding to a 4.20-fold enrichment). Labelled newly inserted new LPS is modestly depleted in OMV ROIs (median $\log_2(S_{new\ LPS})$ = -0.55, 0.68-fold depletion).
  Two-sided Wilcoxon signed-rank tests against 0: $p_{old\ LPS} = 3.73 \times 10^{-9}$, $p_{new\ LPS} = 5.78 \times 10^{-3}$. Paired two-sided Wilcoxon signed-rank on $\log_2(S_{old\ LPS}) - \log_2(S_{new\ LPS})$, $p = 1.86 \times 10^{-9}$ showing that background LPS is significantly enriched in OMVs compared to newly inserted LPS.

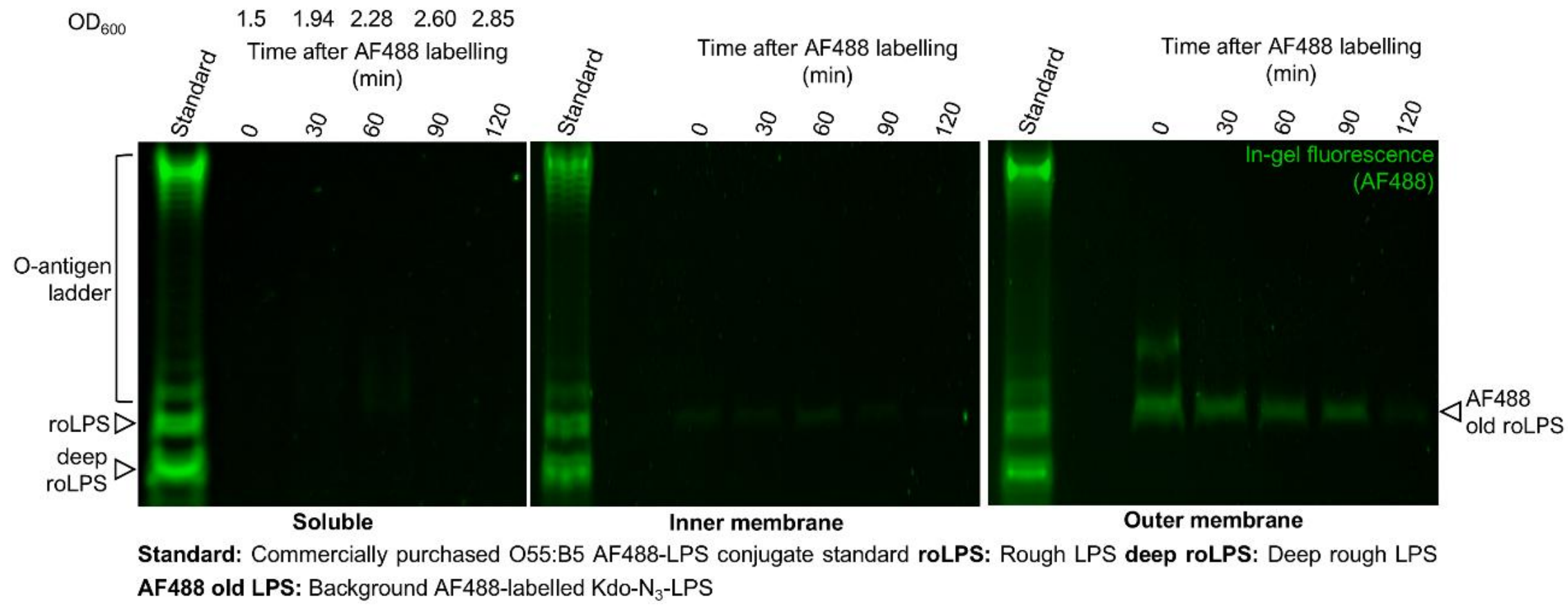

Supplementary Figure 14. Biochemical fractionation excludes soluble intracellular accumulation of fluorescently labelled background LPS over the live-cell 3D-SIM^2 imaging window (related to Fig. 5). *E. coli* BW25113 cells were metabolically labelled with Kdo–N_3, and the pre-existing (background/old) Kdo–N_3–LPS pool was fluorescently conjugated in vivo with DBCO–AF488 via strain-promoted azide(N_3)–alkyne cycloaddition (SPAAC). Labelling and washing steps were performed identically to the live-cell 3D-SIM^2 imaging workflow (Fig. 5) and specificity controls for fluorophore labelling are shown in Supplementary Fig. 15. The zero time point was defined as completion of AF488 labelling and washout of free DBCO–AF488, after which cultures were resuspended to an OD_{600}= 1.5 in prewarmed M9 CDM and incubated at 37 °C; samples were collected at 30 min intervals over 120-min time course as cultures continued to grow (OD_{600} at sampling shown), spanning and exceeding the live-cell imaging window (where cells were mounted at OD_{600} = 2.0). Cells were lysed and fractionated by ultracentrifugation into a soluble (cytoplasm/periplasm) fraction (Soluble, left gel image) and a total membrane pellet. Membranes were further partitioned by *N*-lauryl sarcosinate (Sarkosyl) extraction into inner membrane (IM; Sarkosyl-soluble, centre gel image) and outer membrane (OM; Sarkosyl-insoluble, right gel image) fractions. Equivalent cell fractions were analysed by TSDS-PAGE and imaged by in-gel AF488 fluorescence. A commercially purchased *E. coli* O55:B5 AF488–LPS conjugate was run as a fluorescent reference standard; the O-antigen ladder and approximate migration positions of rough LPS (roLPS) and deep-rough LPS (deep roLPS) are indicated. AF488-labelled background LPS is strongly enriched in the OM fraction and decreases over time, with no detectable time-dependent enrichment in the soluble fraction, confirming that background, fluorescently labelled LPS does not undergo intracellular internalisation and cytoplasmic recycling. A faint, time-invariant signal in the IM fraction is consistent with low-level cross-contamination inherent to detergent-based membrane separation rather than progressive internalisation.

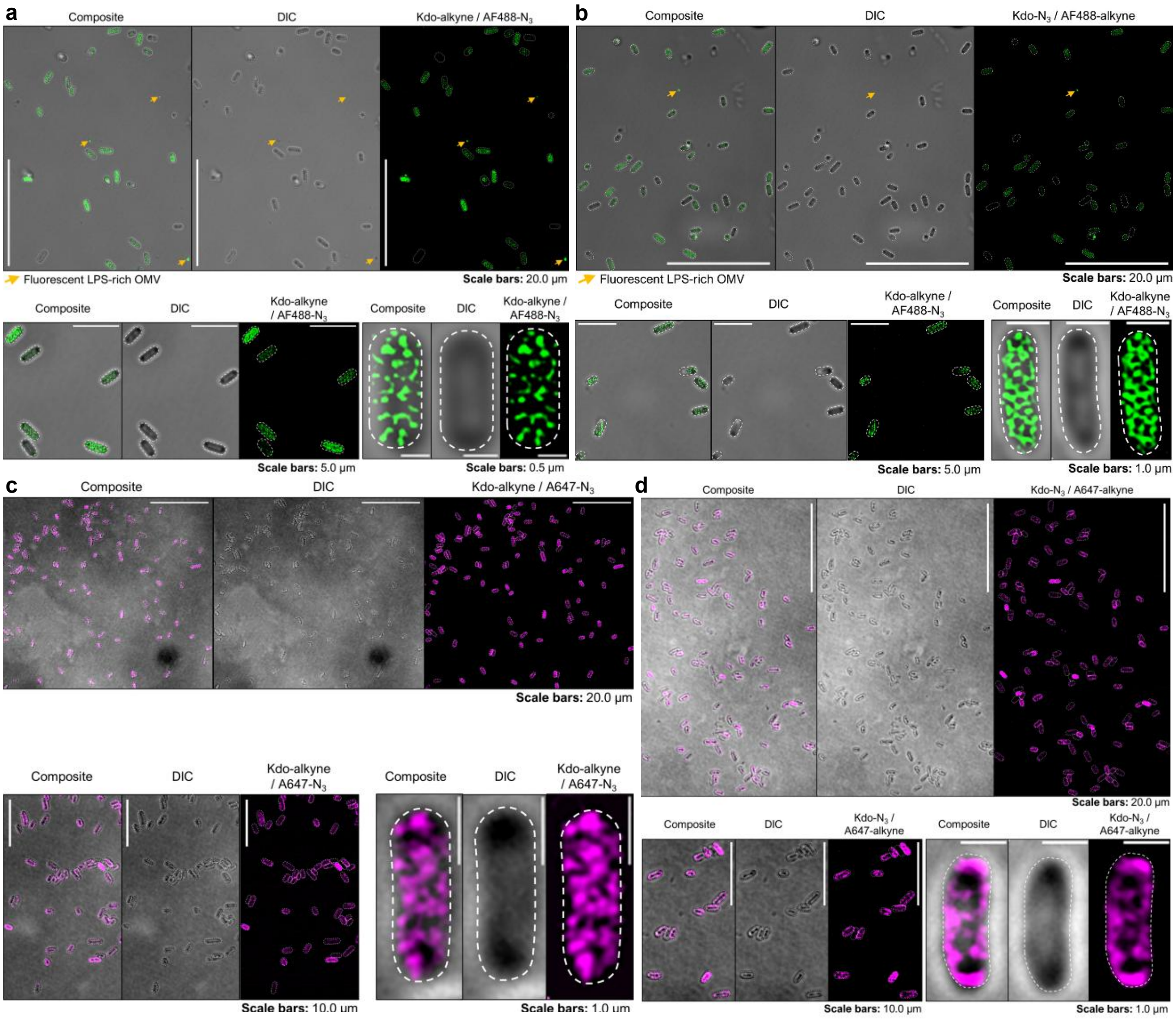
a
Composite
DIC
Kdo-alkyne / AF488-N3
Fluorescent LPS-rich OMV
Scale bars: 20.0 µm
Composite
DIC
Kdo-alkyne / AF488-N3
Scale bars: 5.0 µm
Composite
DIC
Kdo-alkyne / AF488-N3
Scale bars: 0.5 µm
b
Composite
DIC
Kdo-N3 / AF488-alkyne
Fluorescent LPS-rich OMV
Scale bars: 20.0 µm
Composite
DIC
Kdo-alkyne / AF488-N3
Scale bars: 5.0 µm
Composite
DIC
Kdo-alkyne / AF488-N3
Scale bars: 1.0 µm
c
Composite
DIC
Kdo-alkyne / A647-N3
Scale bars: 20.0 µm
Composite
DIC
Kdo-alkyne / A647-N3
Scale bars: 10.0 µm
Composite
DIC
Kdo-alkyne / A647-N3
Scale bars: 1.0 µm
d
Composite
DIC
Kdo-N3 / A647-alkyne
Scale bars: 20.0 µm
Composite
DIC
Kdo-N3 / A647-alkyne
Scale bars: 10.0 µm
Composite
DIC
Kdo-N3 / A647-alkyne
Scale bars: 1.0 µm

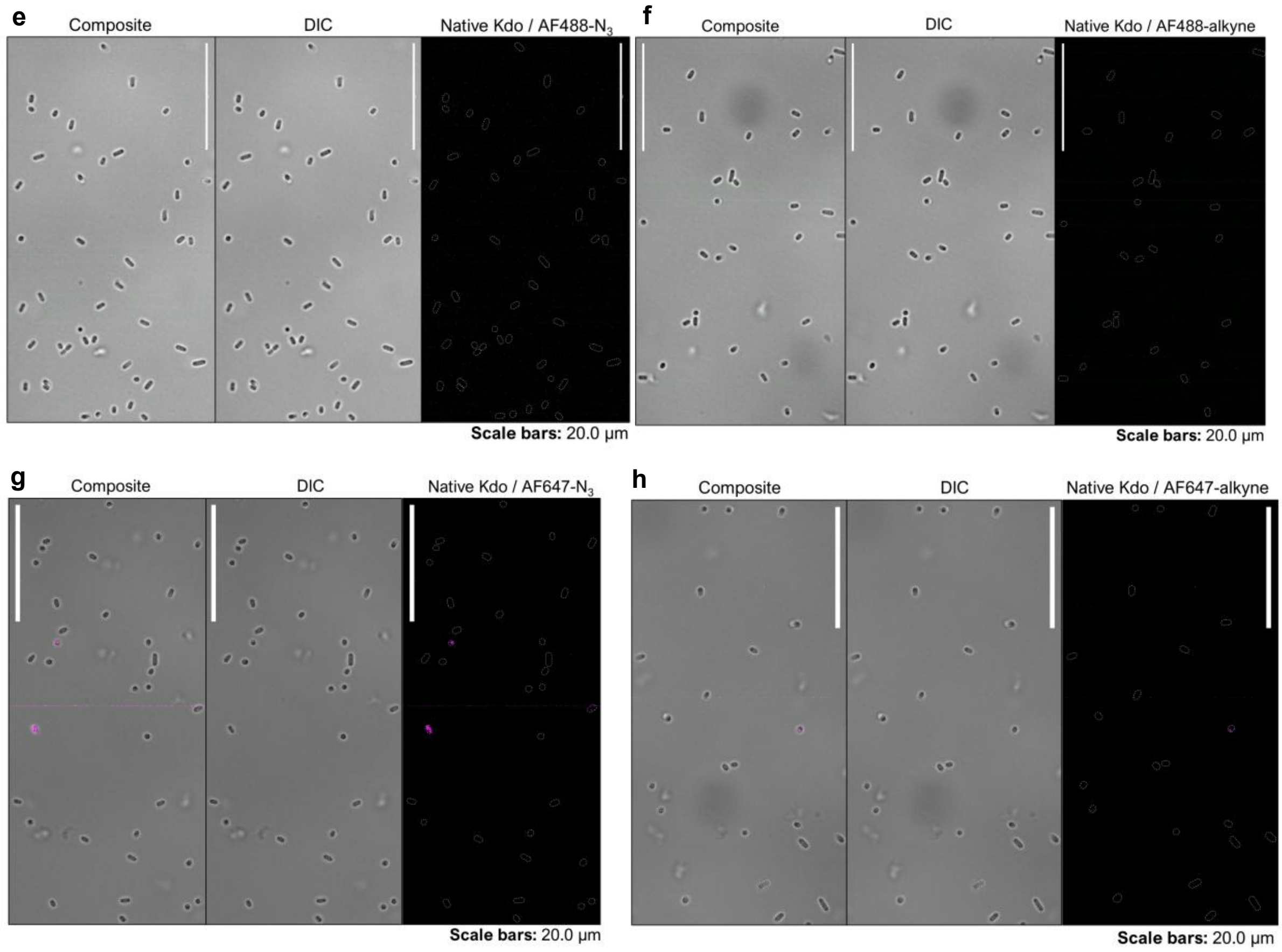

Supplementary Figure 15. Kdo-alkyne and Kdo-N_3 metabolic labelling enables specific in situ CuAAC fluorophore conjugation to Kdo-analogue containing LPS within the outer membranes of *E. coli* BW25113 cells with minimal non-specific staining.

Representative structured illumination microscopy (SIM) fluorescence images with corresponding differential interference contrast (DIC) images and composites (fluorescence overlaid on DIC) of wild-type *E. coli* BW25113 following in vivo metabolic incorporation of functionalised Kdo analogues into LPS and subsequent Cu(I)-catalysed azide(-N_3)–alkyne cycloaddition (CuAAC) with complementary functionalised fluorophores.

a-d. Labelling conditions: Cells cultured in M9 CDM charged with 4 mM Kdo-alkyne ***(a and c)*** or Kdo-N_3 ***(b and d)*** and reacted with AF488-N_3 ***(a)***, AF488-alkyne ***(b)***, AF647-N_3 ***(c),*** or AF647-alkyne ***(d)*** as indicated above each panel**.** Orange arrowheads indicate fluorescent LPS-rich OMV structures observed in the vicinity of cells.

e-h. Negative controls: Cells cultured in M9 CDM charged with 4 mM native Kdo (no bio-orthogonal handle) and subjected to identical CuAAC labelling protocols as A-D; AF488-N_3 ***(e)***, AF488-alkyne ***(f)*****,** AF647-N_3 ***(g)*****,** AF647-alkyne ***(h)*** demonstrating minimal background / non-specific staining in the absence of functionalised Kdo-analogue-containing LPS. Dashed outlines indicate cell boundaries. Imaging was performed across N = 3 biological replicates, with n > 100 cells per condition. Scale bars as indicated.

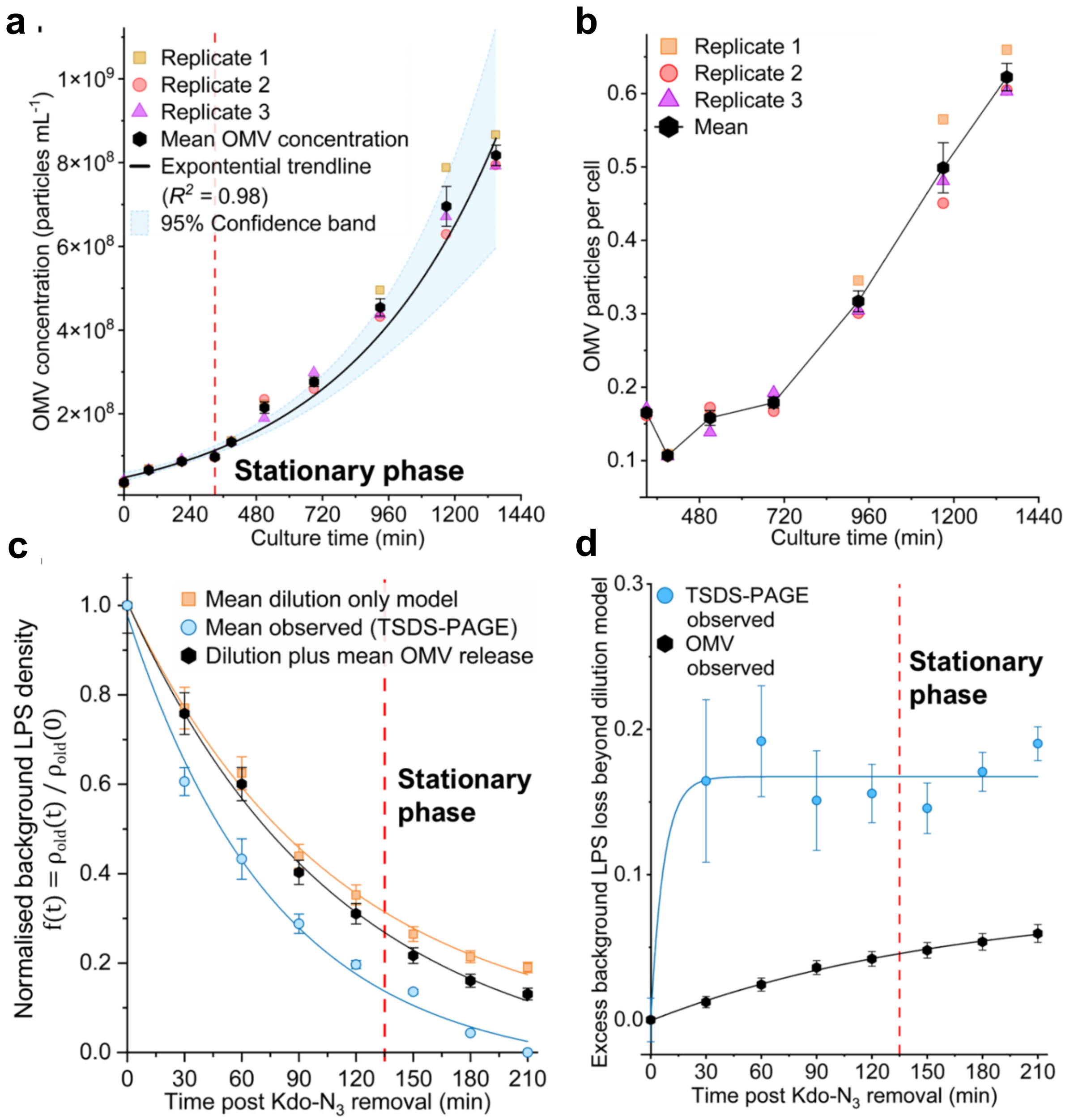

Supplementary Figure 16. NTA-calibrated OMV production accounts for a component of background-LPS turnover beyond dilution due to cell elongation and division.

- **a.** **Outer membrane vesicle (OMV) concentration measured by nanoparticle tracking analysis (NTA) from culture supernatants prepared using 0.45 µm clarification only (without 0.22 µm filtration step)**. Individual biological replicates are shown; black points indicate the mean. The black curve denotes an exponential fit to the mean (R^2=0.98) and the shaded region indicates the 95% confidence band. The vertical dashed line indicates the onset of stationary phase as defined by slowing of population growth as outlined in the main text.
- **b.** **OMV abundance expressed as particles per cell (OMV particles mL^{-1} divided by intact cells mL^{-1}) for each replicate and the mean in stationary phase culture.** Individual biological replicates are shown; black points indicate the mean.
- **c.** **Background (old) LPS density over time expressed as the normalised fraction remaining, $f(t) = \rho_{old}(t)/\rho_{old}(0)$, where ρ_{old} is background-labelled LPS signal per LPS-accessible OM area.** Red points show TSDS-PAGE–derived background LPS loss; orange points show the dilution-only expectation predicted from measured changes in total LPS-accessible OM area in the population; black points show a dilution-plus-OMV model obtained by subtracting the cumulative OMV-exported background pool (Methods). The vertical dashed line indicates the onset of stationary phase (same definition as in A).
- **d.** **Excess background LPS loss beyond dilution $\Delta f(t) = f_{\mathrm{dil}}(t) - f_{\mathrm{obs}}(t)$(red), compared with the modelled fraction of the initial background pool exported via OMVs, $f_{\mathrm{OMV}}(t)$(black).** Points show mean ± s.e.m.

Note: The OMV-derived export term is intentionally conservative because it is derived directly from measured NTA particle counts and size distributions from processed supernatants; any losses during sample handling (*e.g.* clarification/centrifugation and associated recovery inefficiencies) would reduce recovered particle counts and therefore would not inflate the estimated contribution.

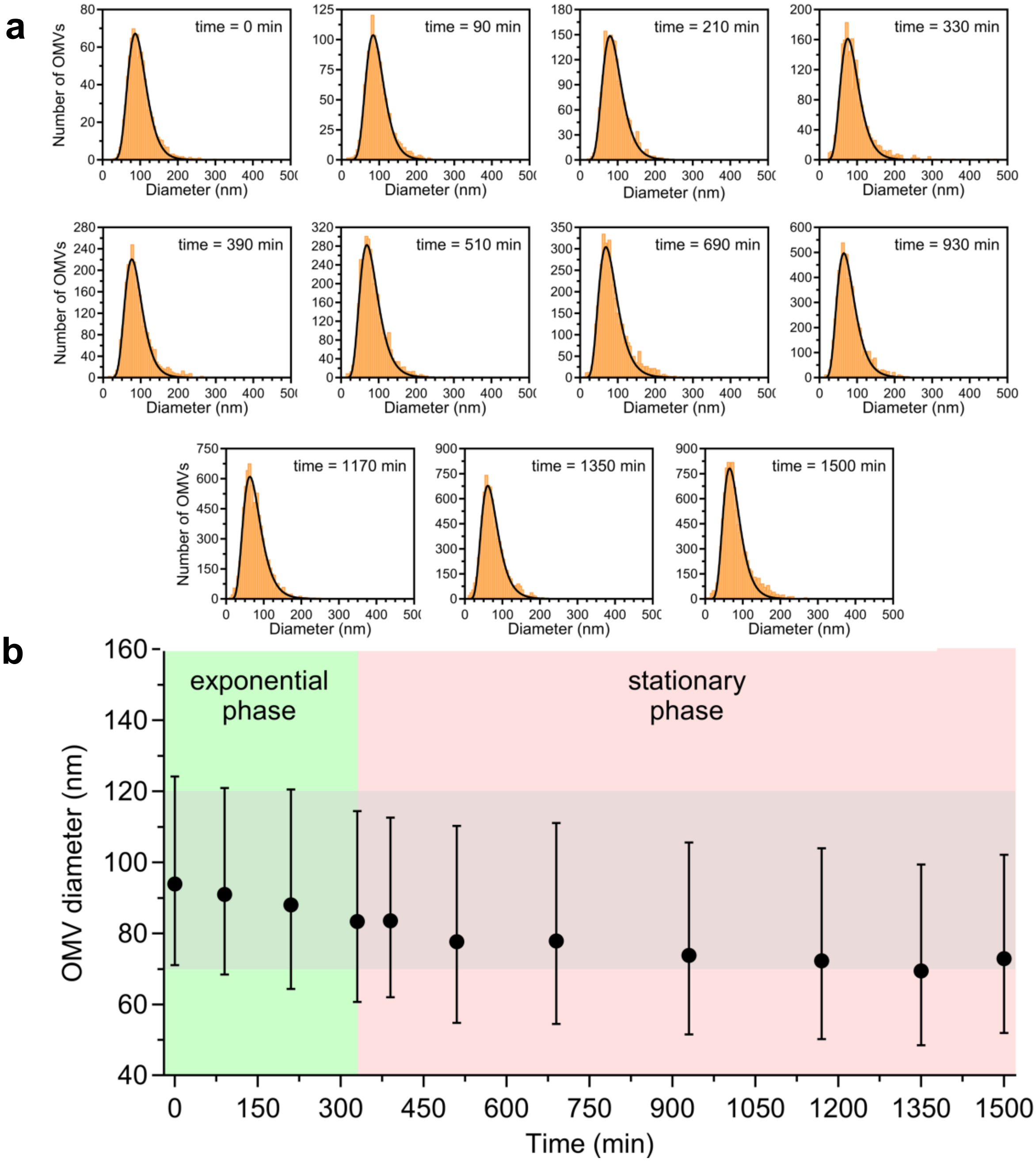

Supplementary Figure 17. Characterisation of OMVs produced by *E. coli* during exponential and stationary phases of growth in chemically defined medium (CDM) and purified using 0.45 µm pore size filtration.

a. Histograms of the diameters measured by nanoparticle tracking (NTA) for OMVs released by *E. coli* BW25113 cells cultured in M9 CDM at 37°C for different growth times (see top right corner of each histogram for growth time). OMVs were purified by filtration through a 0.45 µm pore size hydrophilic syringe filter **(see Methods section for description of the purification procedure)**. Each histogram was compiled using data from technical replicates (N = 3) done on a representative sample. The skewed size histograms at each timepoint were best described by a log-normal distribution function with mostly smaller vesicle sizes observed at each time point. Non-linear regression of the histograms with this distribution function yielded the following R^2 values (listed in chronological order): 0.97, 0.97, 0.97, 0.94, 0.95, 0.96, 0.93, 0.96, 0.96, 0.97 and 0.97.

b. The mean OMV diameters (± SD) obtained from the analysis of the size histogram for each time point. The grey shaded region indicates the predicted range of OMV diameters (d = 73-122 nm) from the OM buckling model for a range of experimentally determined OM thicknesses (= 7.5-21 nm, **see Methods section for mathematical description of the OM buckling model)**.

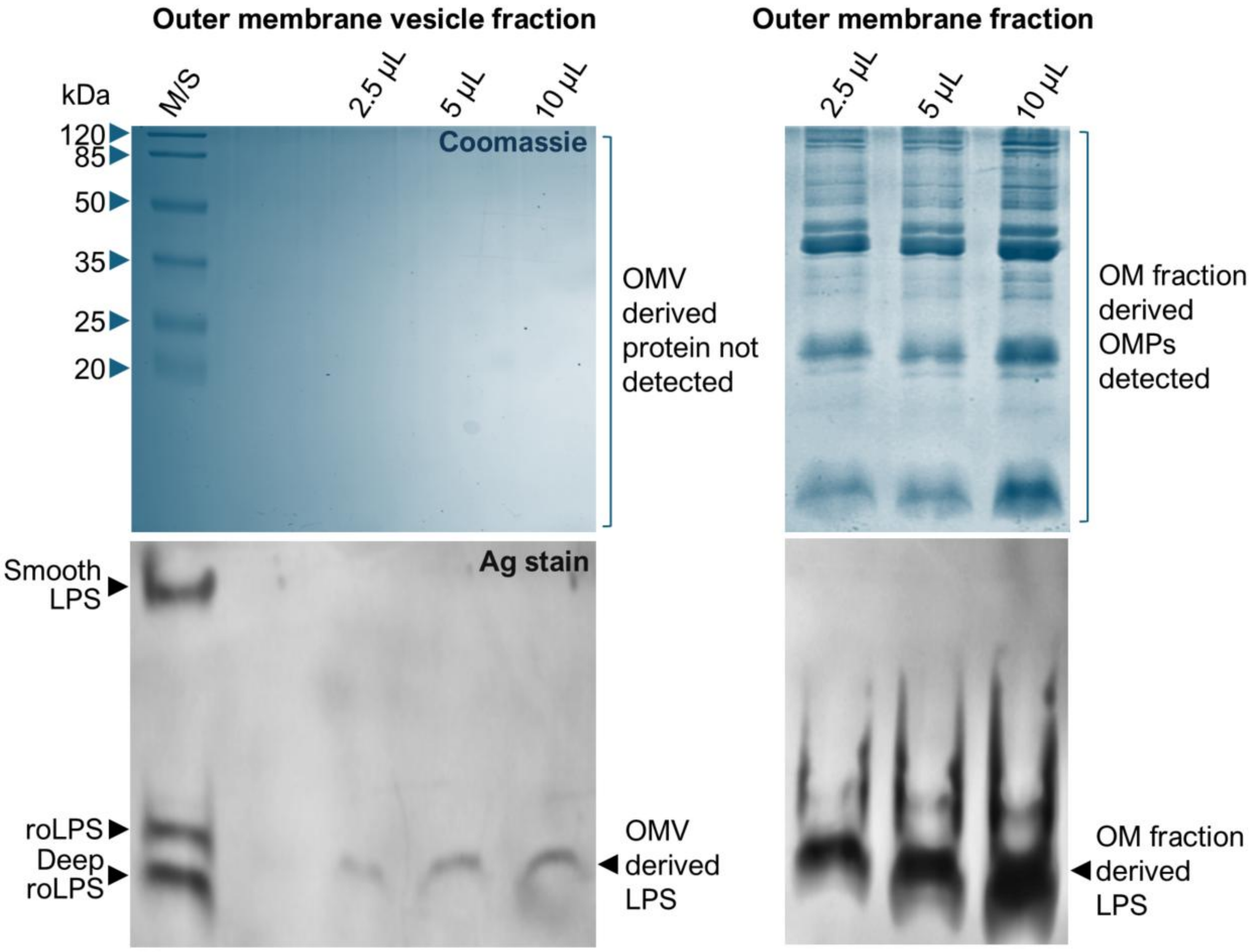

Supplementary Figure 18. *E. coli* BW25113 outer membrane vesicles (OMVs) are depleted in Coomassie-detectable protein content and enriched in LPS content relative to the bacterial outer membrane (OM). OM and OMV fractions were prepared from the same stationary phase culture (OD_{600} = 3.0) and resuspended in equivalent final volumes (100 µL each). OM fractions were generated from washed cell pellets by gentle sonication of the resuspended pellets in detergent-free solution, ultracentrifugation of the disrupted cells, and 1% (w/v) N-lauryl sarcosinate (Sarkosyl) detergent extraction of the resulting pellet to yield a Sarkosyl-insoluble OM pellet.

Protein (top panels). Aliquots of OM and OMV protein fractions were analysed by SDS-PAGE and stained with Coomassie brilliant blue. A mixture of outer membrane-derived proteins with different molecular weights were observed in the OM fraction, whereas the corresponding OMV lanes showed no Coomassie-detectable protein bands at cell density-equivalent gel loadings, indicating that OMVs produced under these conditions were strongly depleted in protein content (i.e., protein content is below the Coomassie detection limit in these samples).

LPS (bottom panels). Cell density-equivalent OM- and OMV-derived samples were treated with Proteinase K to remove residual protein and their LPS content was analysed by Tricine SDS-PAGE. LPS was visualised using a carbohydrate-sensitive silver stain (periodate oxidation followed by silver development). A commercial smooth LPS standard (O55:B5, 1 µg) was included as a reference and to indicate the resolving range of the gel.

Loading / culture equivalents. For each fraction, 2.5, 5.0, and 10.0 µL were loaded (left-to-right). Because each fraction was resuspended to 100 µL from a 10 mL input culture, these loads correspond to 0.25, 0.5, and 1.0 mL culture equivalents per lane, respectively (≈2.76×10^8, 5.52×10^8, and 1.10×10^9 CFU-equivalents using 1.104×10^9 CFU mL^{-1}, 1.10 x 10^{10} CFU per 10 mL culture at the point of harvesting (Supplementary Table 23)). For OMV-containing samples, culture-equivalents refer to the volume of supernatant processed to obtain the material loaded on the gel.

Together, these gels demonstrate that OMV fractions contain readily detectable LPS but are markedly depleted in Coomassie-detectable proteins relative to OM fractions, supporting an LPS-enriched and protein-depleted composition for the OMVs produced under these bacterial growth conditions.

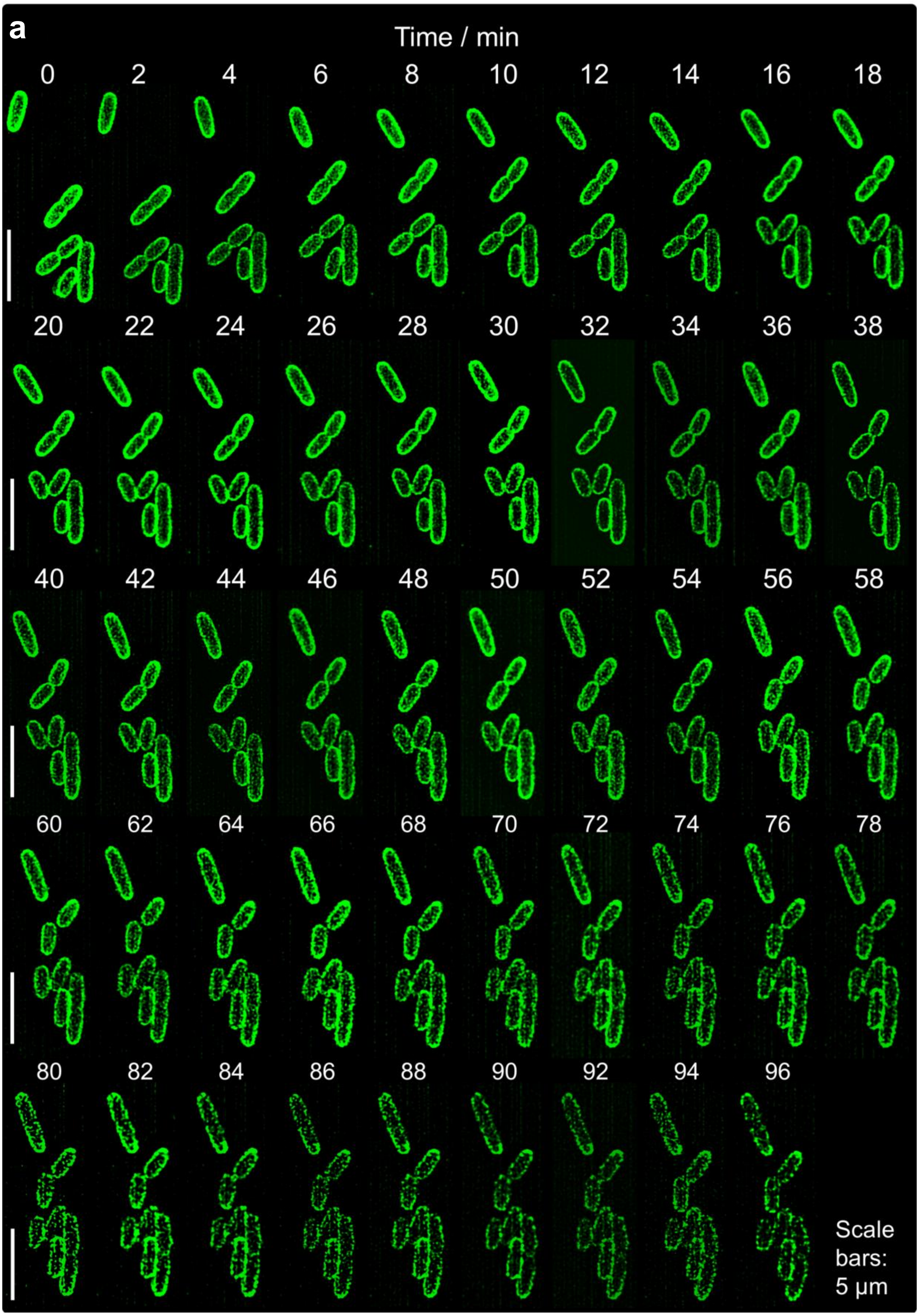
a
Time / min
0
2
4
6
8
10
12
14
16
18
20
22
24
26
28
30
32
34
36
38
40
42
44
46
48
50
52
54
56
58
60
62
64
66
68
70
72
74
76
78
80
82
84
86
88
90
92
94
96
Scale bars: 5 µm

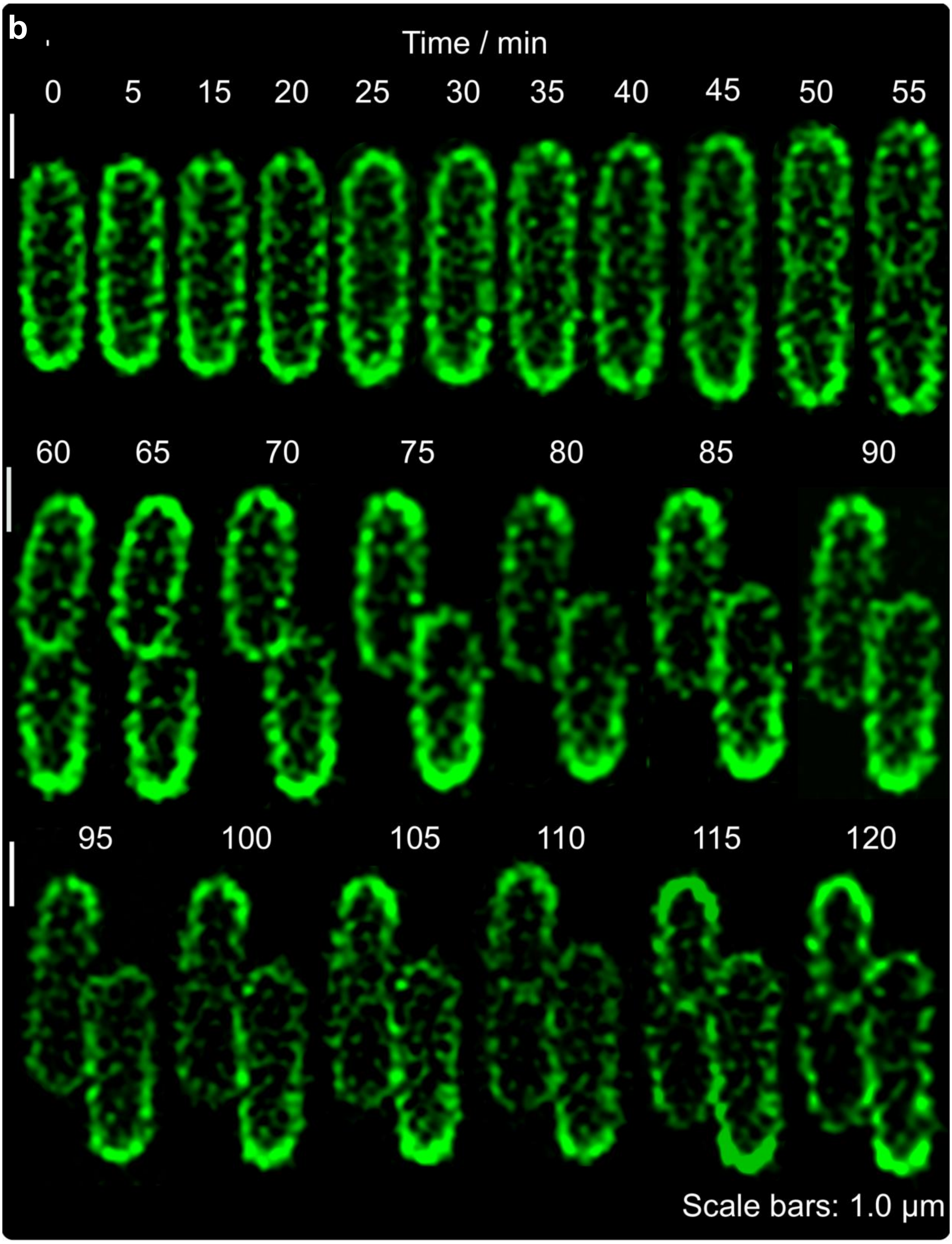
b
Time / min
0
5
15
20
25
30
35
40
45
50
55
60
65
70
75
80
85
90
95
100
105
110
115
120
Scale bars: 1.0 µm

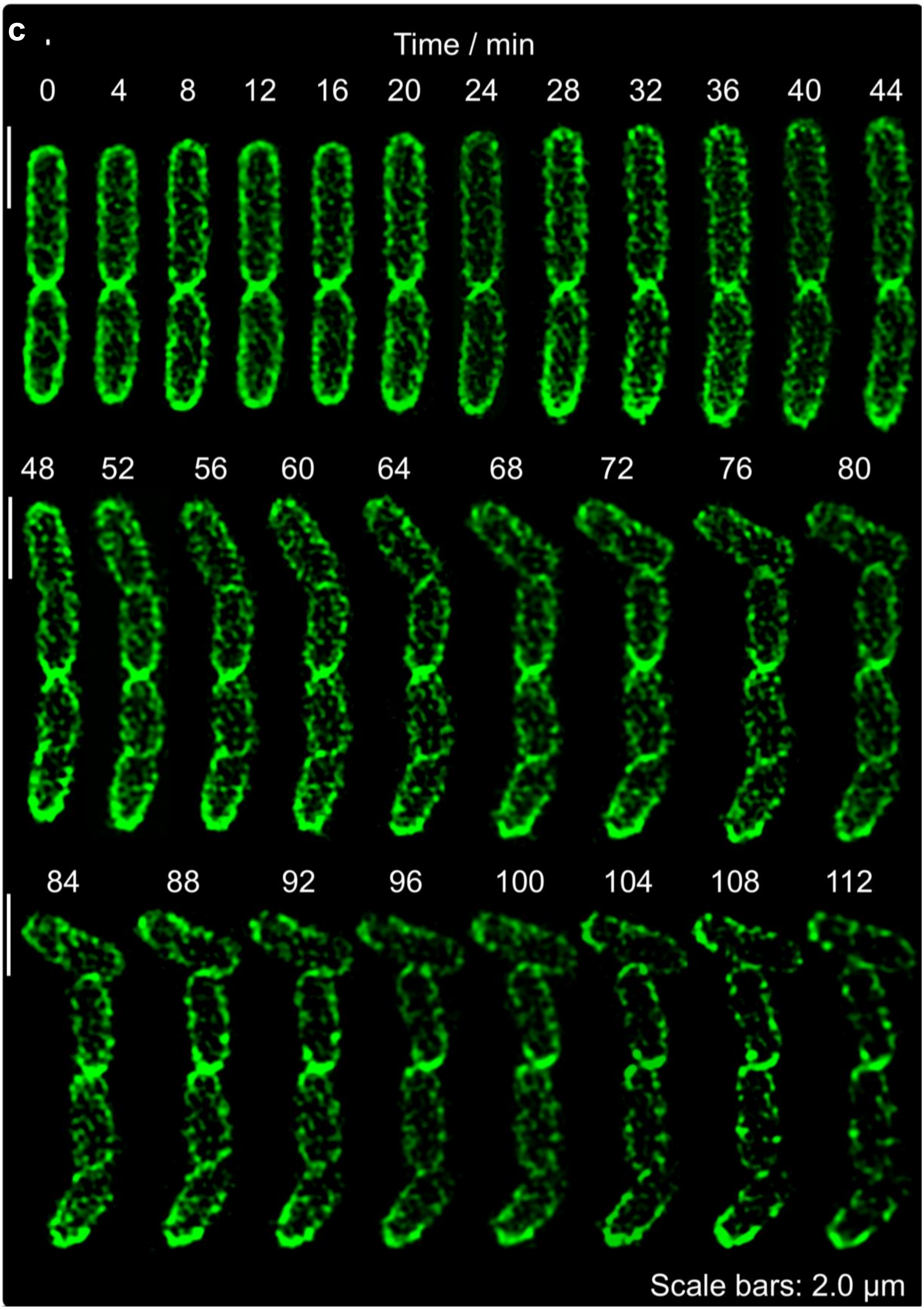
c
Time / min
0
4
8
12
16
20
24
28
32
36
40
44
48
52
56
60
64
68
72
76
80
84
88
92
96
100
104
108
112
Scale bars: 2.0 μm

Supplementary Figure 19. Extended 3D-SIM^2 time courses visualising the loss of background LPS fluorescence in growing and dividing cells. *E. coli* BW25113 cells containing metabolically labelled background Kdo-N_3-LPS were fluorescently labelled in vivo with DBCO-AF488 via strain-promoted azide-alkyne cycloaddition (SPAAC), mounted on supplemented M9 CDM / 2% (w/v) agarose pads, and imaged live at 37 °C by 3D-SIM^2. To extend the live-cell imaging window and reduce AF488 photobleaching, image acquisition settings were modified by reducing both the number of SIM phases from 13 to 9 and the 488 nm laser power.

- **a.** Representative field-of-view time course showing AF488-labelled background LPS in live *E. coli* cells over 96 min. Each frame is labelled with the image acquisition time in minutes. **Scale bars:** 5.0 µm.
- **b.** Representative single-cell time course showing OM-associated AF488-labelled background LPS over 120 min. Each frame is labelled with the image acquisition time in minutes. **Scale bars:** 1.0 µm.
- **c.** Representative time course showing AF488-labelled background LPS in two cells over 112 min. Each frame is labelled with the image acquisition time in minutes. **Scale bars:** 2.0 µm.

Across these extended time courses, background LPS fluorescence retained a punctate, clustered distribution, while the labelled signal from LPS-rich clusters gradually decreased over time. No time-dependent polar enrichment or coalescence of LPS-rich clusters was observed. These data extend the live-cell observations shown in Fig. 5 and Supplementary Fig. 5, and provide direct visual confirmation of the loss or dilution of the labelled background LPS pool, complementing the fixed-cell dSTORM and biochemical membrane fractionation analyses of background LPS turnover rate.

Supplementary Tables

Supplementary Table 1. Details of Gram-negative bacterial strains used in this study

Strain	Organism	Selection antibiotic	Notes	Ref.
**K-12 substr. BW25113**	*E. coli*	None	Parent strain of Keio collection. *lacI^q^ rrnB$_{T14}$ ΔlacZ$_{WJ16}$ hsdR514 ΔaraBAD$_{AH33}$ ΔrhaBAD$_{LD78}$*	1
**BW25113 pBAD-lptD***	*E. coli*	100 µg mL^{-1} AMP 35 µg mL^{-1} CHL	BW25113 strain co-transformed with pBAD-lptD* and pEVOL-pylRS plasmids. Produces recombinant LptD with ncAA propargyl-L-Lysine (Lysine analogue) at position D600 when provided with ncAA resulting from leaky *lptD** expression (L-arabinose was omitted to prevent over-production of LptD*).	1
**∆*ompA* pBAD-ompA***	*E. coli*	100 µg mL^{-1} AMP 35 µg mL^{-1} CHL 30 µg mL^{-1} KAN	Keio collection strain JW0940-KC with *ompA* gene replaced by kanamycin resistance cassette and transformed with pBAD-ompA* and pEVOL-pylRS plasmids. Produces recombinant OmpA with ncAA propargyl-L-Lysine (Lysine analogue) at position E89 when provided with the ncAA resulting from leaky *ompA** gene expression (L-arabinose was omitted to prevent over-production of OmpA*).	2

Antibiotic abbreviations: KAN = Kanamycin (Sigma-Aldrich), **AMP =** Ampicillin (Melford Laboratories Ltd.), **CHL =** Chloramphenicol (Sigma-Aldrich)

Supplementary Table 2. DNA primer sequences

Primer ID	Sequence (5' → 3')	Notes
lptD^{WT}-for	TTTGGGCTAACAGGAGGAATTACATATGAAAAAACGTATCCCCACTCTCC	*lptD* gene PCR amplification
lptD^{WT}-rev	GAGATGAGTTTTTGTTCTAGAAAGCTTACAAAGTGTTTTGATACGGCAGA	*lptD* gene PCR amplification
pBADcLIC-for	TCTGCCGTATCAAAACACTTTGTAAGCTTTCTAGAACAAAAACTCATCTC	pBADcLIC vector PCR amplification
pBADcLIC-rev	GGAGAGTGGGGATACGTTTTTTCATATGTAATTCCTCCTGTTAGCCCAAA	pBADcLIC vector PCR amplification
pBADcLIC-forS	ATGCCATAGCATTTTTATCC	pBAD-lptD sequencing
pBADcLIC-revS	GATTTAATCTGTATCAGG	pBAD-lptD sequencing
lptDmid-forS	TACTTTGAGTTCTACCTGCC	pBAD-lptD sequencing
lptDmid-revS	ATGCTGGAGTTACTGGTCGC	pBAD-lptD sequencing
lptD$^{[D600]}$-forM	CGATGACAACATAACATGGGAGAATTAGGACAAAACGGGTTCACTGGT	pBAD-lptD PCR mutagenesis
lptD$^{[D600]}$-revM	ACCAGTGAACCCGTTTTGTCCTAATTCTCCCATGTTATGTTGTCATCG	pBAD-lptD PCR mutagenesis
ompA^{WT}-for	GCTAACAGGAGGAATTAACCATGGATGAAAAAGACAGCTATCGCGATTG	*ompA* gene PCR amplification
ompA^{WT}-rev	GATGAGTTTTTGTTCTAGAAAGCTTCGTTAAGCCTGCGGCTGAGTTAC	*ompA* gene PCR amplification
pBAD-for-2	CGTTGTAACTCAGCCGCAGGCTTAACGAAGCTTTCTAGAACAAAAACTCATCTCAG	pBADcLIC vector PCR amplification
pBAD-rev-2	CAATCGCGATAGCTGTCTTTTTCATCCATGGTTAATTCCTCCTGTTAGCC	pBADcLIC vector PCR amplification
ompA$^{[E89]}$-for	CGTATGCCGTACAAAGGCAGCGTTTAGAACGGTGCATACAAAGCTCAGGGC	pBAD-ompA PCR mutagenesis
ompA$^{[E89]}$-rev	GCCCTGAGCTTTGTATGCACCGTTCTAAACGCTGCCTTTGTACGGCATACG	pBAD-ompA PCR mutagenesis

Supplementary Table 3. Composition of supplemented M9 chemically defined medium (pH 7.2)

Component	Concentration
Na_2HPO_4	48.0 mM
KH_2PO_4	22.0 mM
NaCl	8.6 mM
D-glucose	0.4% (w/v)
NH_4Cl	1.0 g L^{-1}
Casamino acids	0.05% (w/v)
$FeSO_4$	0.1 mM
$MgSO_4$	2.0 mM
$CaCl_2$	0.1 mM

Supplementary Table 4. Final concentrations of alkyne functionalised dyes (Click Chemistry Tools, Vector Laboratories, Inc.) used in CuAAC Click-iT reaction mixtures

AFDye	**Final concentration** (μM)
AF488	10
AF647	50

Supplementary Table 5. Final concentration of AF488-DBCO (Click Chemistry Tools, Vector Laboratories, Inc.) used in SPAAC reaction mixtures

AFDye DBCO type	**Final concentration** (μM)
AF488	25

Supplementary Table 6. Dual-colour CuAAC labelling order and dye combinations for LptD* and newly inserted LPS (Kdo-N_3) used to confirm that dye identity and labelling order do not affect two-colour dSTORM results

First labelled species / fluorescent dye	Second CuAAC step: labelled species / dye
LptD* / AF488-N_3	Newly inserted LPS / AF647-alkyne
Newly inserted LPS / AF647-alkyne	LptD* / AF488-N_3
LptD* / AF647-N_3	Newly inserted LPS / AF488-alkyne
Newly inserted LPS / AF488-alkyne	LptD* / AF647-N_3

Alkyne-functionalised dyes label Kdo-N_3, and azide-functionalised dyes label the alkyne handle on LptD*

Supplementary Table 7. Components in glucose oxidase (GluOx) / catalase (Cat) oxygen scavenging buffer system

Component	**Volume** (μL)
Degassed deionised H_2O	895
PBS pH 7.4 [40 x] (BioStatus Ltd.)	25
GluOx [10 mg mL^{-1}]	20
Cat [2 mg mL^{-1}]	20
Glucose [300 mg mL^{-1}]	40

Supplementary Table 8. Additional settings for dSTORM image acquisition with Zeiss Elyra 7 super-resolution imaging system

Dye	Laser beam splitter	Laser	Emission filter (nm)	xy scaling (μm pixel^{-1})	TIRF mirror angle	TIRF collimator
AF488	405/488/561/642	488 nm DPSS	BP420-480 + BP490-550	0.097	60°	230
AF647	405/488/561/642	561 nm DPSS	BP570-620 + LP655	0.097	60°	400

Supplementary Table 9. Typical dSTORM image filtering settings

Filter	AF488 dye	AF647 dye
Precision (nm)	1 – 40	1 – 40
Number of photons	1 – 1750	1 – 1250
Point squared function half width (nm)	60 – 200	100 – 300
Background variance	1 – 300	1 – 300
Chi-squared	0.4 – 1.2	0.4 – 1.2

Supplementary Table 10. Mean LptD* vs newly inserted LPS cross-correlation function data derived from analysis of dual-colour dSTORM outputs for individual cells (n = 30 cells, N = 3 biological replicates)

Radius (nm)	Mean *g(r)*	S.E.M. [Mean g(r)]
30	4.37	0.51
60	3.98	0.40
90	3.43	0.27
120	2.91	0.18
150	2.49	0.12
180	2.13	0.09
210	1.84	0.07
240	1.63	0.06
270	1.45	0.05
300	1.30	0.04
330	1.21	0.04
360	1.11	0.04
390	1.03	0.04
420	0.96	0.04
450	0.92	0.04
480	0.87	0.04
510	0.83	0.04
540	0.81	0.04
570	0.78	0.04
600	0.75	0.04
630	0.73	0.05

Supplementary Table 11. Results from two-tailed Mann-Whitney U-test assessing the significance of variation between LptD* vs newly inserted LPS Pearson correlation coefficient (PCC) values for polar, mid-cell and entire OM regions of *E. coli* BW25113 cells imaged via two-colour dSTORM after sequential dual-colour CuAAC labelling (two sequential CuAAC reactions, one dye per step, with wash steps between reactions) of LptD* and newly inserted LPS

Condition 1	Condition 2	n_1	n_2	Median PCC_1	Median PCC_2	*p*	U	Z
Pole 1	Mid-cell	30	30	0.49	0.34	0.32	518	0.99
Pole 1	Pole 2	30	30	0.49	0.52	0.46	399	-0.75
Pole 1	Entire OM	30	30	0.49	0.37	0.24	530	1.18
Pole 2	Mid-cell	30	30	0.52	0.34	0.10	561	1.63
Pole 2	Entire OM	30	30	0.52	0.37	0.07	574	1.83
Mid-cell	Entire OM	30	30	0.34	0.37	0.97	447	-0.04

Supplementary Table 12. Dual-colour CuAAC labelling order and dye combinations for background (Kdo-alkyne) and newly inserted (Kdo-N_3) LPS used to confirm that dye identity and labelling order do not affect two-colour dSTORM results.

First CuAAC step: labelled species / dye	Second CuAAC step: labelled species / dye
Newly inserted LPS / AF488-alkyne	Background LPS / AF647-N_3
Background LPS / AF647-N_3	Newly inserted LPS / AF488-alkyne
Newly inserted LPS / AF647-alkyne	Background LPS / AF488-N_3
Background LPS / AF488-N_3	Newly inserted LPS / AF647-alkyne

Alkyne-functionalised dyes label Kdo-N_3, and azide-functionalised dyes label Kdo-alkyne

Supplementary Table 13. DBSCAN-defined OM coverage by background and newly inserted LPS clusters and normalised cluster-area intersection over time

Time post Kdo-analogue exchange (min)	OM area covered by background LPS clusters (%)	S.E.M.	OM area covered by newly inserted LPS clusters (%)	S.E.M.	Normalised cluster-area intersection (mask intersection fraction)	S.E.M.
0	54.48	6.86	0.26	0.07	0.0027	0.0008
15	45.31	4.32	7.24	1.79	0.0635	0.0154
30	37.29	4.12	13.88	2.14	0.0977	0.0153
45	32.11	1.90	19.64	1.59	0.1280	0.0201
60	22.28	3.09	21.34	3.04	0.1440	0.0212
75	18.10	3.12	32.52	3.99	0.1281	0.0205
90	15.63	2.51	37.04	3.46	0.1164	0.0185
105	11.09	1.89	41.76	4.04	0.1051	0.0159
120	5.95	0.89	47.73	6.73	0.0869	0.0125
**Ref:** Figure 2e					**Ref:** Figure 2f	

Normalised cluster-area intersection was calculated as (area of intersection between independently segmented background- and newly inserted-LPS cluster regions) divided by (background cluster area + newly inserted cluster area)

**Supplementary Table 14.** Composition of 4x TSDS-PAGE sample loading buffer	
**Component**	**Final concentration**
Tris-Cl pH 6.8	0.25 M
Sodium dodecyl sulphate (SDS)	227 mM
Bromophenol blue	0.02 % (w/v)
Glycerol	4.3 M
**Note:** β-ME added to a final concentration of 2% (v/v) immediately before use.	

**Supplementary Table 15.** AF488 emission dSTORM image filtering settings	
**Filter**	**AF488 value range**
Precision / nm	1 – 40
Number of photons	1 – 1500
Point spread function half width / nm	60 – 220
Background variance	1 – 300
Chi squared	0.4 – 1.2

Supplementary Table 16. Mean background LPS turnover rates in the outer membrane of *E. coli* BW25113 cells derived from dSTORM analyses during Kdo-N_3 / native Kdo pulse-chase experiments. Data show the mean AF488–LPS emission density (μm^{-2}) and standard error (S.E. M.) at 30-minute time intervals following removal of Kdo–N_3 from the culture medium. Each biological replicate (N = 3) includes n ≥ 10 individual cells per time point. In all replicates, background LPS levels decay exponentially over time. The rate of turnover does not appear to be altered by the transition from late exponential to stationary phase.

**Biological replicate**	**1.**		**2.**		**3.**	
**Time post Kdo-N_3 removal (h)**	**Mean AF488-LPS emissions μm^{-2}**	**S.E.M.**	**Mean AF488-LPS emissions μm^{-2}**	**S.E.M.**	**Mean AF488-LPS emissions μm^{-2}**	**S.E.M.**
0.0	1232.39	33.21	1425.17	50.28	1436.30	42.03
0.5	1032.55	36.27	1146.42	12.07	1022.91	17.80
1.0	693.62	21.23	817.34	22.27	835.98	14.37
1.5	337.77	11.71	472.33	23.17	380.13	10.87
2.0	257.49	12.80	503.20	6.63	312.24	8.67
2.5	241.37	8.25	418.62	21.09	254.52	6.48
3.0	201.97	9.49	265.33	9.85	234.98	6.32
3.5	164.07	5.15	153.80	9.56	178.45	5.15

Supplementary Table 17. Mean LPS insertion rates in the OM of *E. coli* BW25113 cells derived from dSTORM analyses during native Kdo / Kdo-N_3 pulse-chase experiments. Data show the mean AF488–LPS emission density (μm^{-2}) and standard error (S.E.M.) at 30-minute time intervals following addition of Kdo–N_3 to the culture medium. Each biological replicate (N = 3) includes n ≥ 10 individual cells per time point. In all replicates, new LPS levels increase in a linear manner over time. The rate of new LPS insertion does not appear to be altered by the transition from late exponential to stationary phase.

**Biological replicate**	**1.**		**2.**		**3.**	
**Time post Kdo-N_3 addition (h)**	**Mean AF488-LPS emissions μm^{-2}**	**S.E.M.**	**Mean AF488-LPS emissions μm^{-2}**	**S.E.M.**	**Mean AF488-LPS emissions μm^{-2}**	**S.E.M.**
0.0	209.85	8.13	244.28	7.08	206.01	13.22
0.5	306.97	20.91	382.12	24.63	273.46	28.30
1.0	555.61	27.56	535.04	21.62	451.67	25.19
1.5	732.44	61.67	714.16	16.45	741.65	44.98
2.0	965.05	36.97	887.01	27.06	833.49	30.79
2.5	1021.37	25.68	1096.33	29.17	1089.09	49.11
3.0	1196.48	24.91	1228.20	29.30	1140.78	34.65
3.5	1258.06	37.57	1385.65	67.81	1386.24	63.32

Supplementary Table 18. Summary of DBSCAN statistics for background LPS

**Time (min)**	**Mean cluster number per μm^2 of OM**	**Mean cluster surface area (nm^2)**	**Cluster density (localisations μm^{-2})**
0	24	17578	20373.65
30	19	14696	20373.65
60	19	8749	8748.68
90	14	5244	7119.06
120	13	4303	6537.44
150	13	3869	6754.92
180	12	3504	6967.52
210	12	3442	5121.69

Supplementary Table 19. Summary of DBSCAN statistics for newly inserted LPS

**Time (min)**	**Mean cluster number per μm^2 of OM**	**Mean cluster surface area (nm^2)**	**Cluster density (localisations μm^{-2})**
30	14	5962	13182.84
60	14	6619	13876.97
90	15	10558	12902.23
120	19	10295	14765.60
150	25	9820	19430.04
180	30	10095	21730.29
210	34	9724	23264.76

Supplementary Table 20. Additional settings for lattice SIM image acquisition with Zeiss Elyra 7 super-resolution imaging system

Experiment	Laser beam splitter	Laser	Emission filter (nm)	laser power (%)	Exposure (ms)	*xy* scaling (µm)	*z* scaling (µm)	Grating period (nm)	Phases
Standard live-cell 3D-SIM[2]	405/488/561 /642	488 nm DPSS	BP420 – 480 + BP490 - 550	3	50	0.031	0.110	718.33	13
Extended duration live cell 3D SIM[2]	405/488/561 /642	488 nm DPSS	BP420 – 480 + BP490 - 550	0.5-1.5	50	0.031	0.110	718.33	9

BP: Band pass. **LP:** Long Pass. **DPSS:** Diode pumped solid state. Images collected using a Plan-Apochromat 63x /1.4 (DIC M27) oil immersion objective lens. **Image size:** 10 µm x 10 µm x 2 µm, 16-bit

Supplementary Table 21. OMV lysis buffer composition. Prepared with HPLC-grade water. β-Mercaptoethanol added to a final concentration of 2% (v/v) immediately before use.

Component	Concentration
Sodium dodecyl sulfate (SDS)	2% (w/v)
Tris-Cl pH 6.5	60 mM
EDTA	1 mM

Supplementary Table 22. OD_{600} measurements for *E. coli* BW25113 cultured in M9 CDM during the Kdo–N_3/native Kdo pulse–chase time course. Stationary phase was defined operationally as the onset of the OD_{600} plateau (see Methods). Across replicates, stationary-phase entry occurred between 120–150 min under these conditions

Time (min)	Rep. 1 OD_{600}	Rep. 2 OD_{600}	Rep. 3 OD_{600}	Rep. 4 OD_{600}	Rep. 5 OD_{600}	Rep. 6 OD_{600}	Rep. 7 OD_{600}
0	0.50	0.48	0.55	0.49	0.52	0.47	0.49
30	0.65	0.605	0.7	0.67	0.62	0.61	0.59
60	0.85	0.9	0.95	1.00	0.82	0.80	0.79
90	1.31	1.35	1.4	1.45	1.25	1.23	1.21
120	1.75	1.80	1.90	2.00	1.70	1.82	1.69
150	2.10	2.15	2.30	2.40	2.05	2.20	2.00
180	2.30	2.35	2.55	2.70	2.32	2.50	2.25
210	2.44	2.50	2.70	2.90	2.60	2.70	2.44

Supplementary Table 23. Concentration of viable bacterial cells in culture as a function of time. The colony forming units (CFU) were calculated for each time point using serial dilutions of the bacterial culture and the plate count method. Bacterial cells were cultured in supplemented M9 CDM at 37 °C. The two separate bacterial cultures (ID numbers 1 and 2) used to determine CFU were grown under identical conditions.

**Time (min)**	**Growth phase**	**Culture ID number**	**OD_{600}**	**CFU / ml in original culture** ($\times 10^9$)	**(CFU/ml) / OD_{600}** ($\times 10^8$)
0	---	1	0.150	0.0424	2.83
60	exponential	1	0.309	0.032	1.04
90	exponential	1	0.441	0.096	2.18
150	exponential	1	0.696	0.142	2.05
210	exponential	1	1.34	0.296	2.21
270	late exponential	1	2.47	0.592	2.40
330	late exponential	1	3.07	0.752	2.45
390	stationary	1	3.05	1.104	3.62
450	stationary	1	3.23	1.072	3.32
510	stationary	2	2.94	0.928	3.16
570	stationary	2	3.05	0.944	3.10
630	stationary	2	2.99	0.912	3.05
690	stationary	2	2.95	1.048	3.55
1500	stationary	1	3.21	1.176	3.66

Supplementary Table 24. Per-cell localisation-density enrichment of background (old) and newly inserted (new) LPS in OMV nucleation (bleb) ROIs from analysis of two colour dSTORM images of cells captured in the process of OMV production

Event ID	Rep.	Old LPS dye	New LPS dye	Area cell ROI (µm²)	Area OMV ROI (µm²)	$N_{old\ LPS,cell}$	$\rho_{old\ LPS,cell}$ (µm⁻²)	$N_{old\ LPS,OMV}$	$\rho_{old\ LPS,OMV}$ (µm⁻²)	$S_{old\ LPS} = \rho_{old\ LPS,OMV} / \rho_{old\ LPS,cell}$	$\log2(S_{old\ LPS})$	$N_{new\ LPS,cell}$	$\rho_{new\ LPS,cell}$ (µm⁻²)	$N_{new\ LPS,OMV}$	$\rho_{new\ LPS,OMV}$ (µm⁻²)	$S_{new\ LPS} = \rho_{new\ LPS,OMV} / \rho_{new\ LPS,cell}$	$\log2(S_{new\ LPS})$	old LPS : new LPS ratio (cell ROI)	old LPS : new LPS ratio (OMV ROI)	ratio-of-ratios (OMV/cell)	$\Delta\log_2 S$ (old LPS − new LPS)	Rank by $\log_2 (S_{old\ LPS})$
14	2	AF488	AF647	1.14	0.07	1259	1104.4	2286	32657.1	29.57	4.89	2850	2500.0	1346	19228.6	7.691	2.943	0.4	1.7	3.8	1.94	1
13	1	AF647	AF488	0.70	0.02	2286	3265.7	1504	75200.0	23.03	4.53	1346	1922.9	30	1500.0	0.780	-0.358	1.7	50.1	29.5	4.88	2
28	3	AF647	AF488	1.86	0.1	198	106.5	238	2380.0	22.36	4.48	464	249.5	40	400.0	1.603	0.681	0.4	6.0	13.9	3.80	3
15	2	AF488	AF647	0.67	0.02	1226	1829.9	751	37550.0	20.52	4.36	2194	3274.6	45	2250.0	0.687	-0.541	0.6	16.7	29.9	4.90	4
25	2	AF488	AF647	1.62	0.14	344	212.3	445	3178.6	14.97	3.90	547	337.7	86	614.3	1.819	0.863	0.6	5.2	8.2	3.04	5
8	1	AF647	AF488	0.96	0.06	5039	5249.0	4024	67066.7	12.78	3.68	1981	2063.5	60	1000.0	0.485	-1.045	2.5	67.1	26.4	4.72	6
23	2	AF488	AF647	2.20	0.09	661	300.5	331	3677.8	12.24	3.61	1158	526.4	69	766.7	1.457	0.543	0.6	4.8	8.4	3.07	7
21	2	AF488	AF647	1.84	0.04	147	79.9	32	800.0	10.01	3.32	1442	783.7	33	825.0	1.053	0.074	0.1	1.0	9.5	3.25	8
11	1	AF647	AF488	1.00	0.04	2582	2582.0	753	18825.0	7.29	2.87	2690	2690.0	35	875.0	0.325	-1.620	1.0	21.5	22.4	4.49	9
18	1	AF647	AF488	1.46	0.13	1745	1195.2	1077	8284.6	6.93	2.79	8046	5511.0	462	3553.9	0.645	-0.633	0.2	2.3	10.8	3.43	10
29	3	AF647	AF488	1.20	0.13	251	209.2	152	1169.2	5.59	2.48	402	335.0	35	269.2	0.804	-0.315	0.6	4.3	7.0	2.80	11
9	1	AF647	AF488	0.95	0.08	6900	7263.2	3035	37937.5	5.22	2.38	893	940.0	40	500.0	0.532	-0.911	7.7	75.9	9.8	3.30	12
27	3	AF647	AF488	1.23	0.18	374	304.1	258	1433.3	4.71	2.24	282	229.3	40	222.2	0.969	-0.045	1.3	6.5	4.9	2.28	13
17	1	AF647	AF488	0.61	0.03	2209	3621.3	474	15800.0	4.36	2.13	2681	4395.1	24	800.0	0.182	-2.458	0.8	19.8	24.0	4.58	14
20	1	AF647	AF488	1.57	0.11	216	137.6	66	600.0	4.36	2.12	1905	1213.4	90	818.2	0.674	-0.569	0.1	0.7	6.5	2.69	15
19	1	AF647	AF488	2.21	0.08	1134	513.1	166	2075.0	4.04	2.02	7860	3556.6	557	6962.5	1.958	0.969	0.1	0.3	2.1	1.05	16
2	1	AF647	AF488	0.96	0.01	5429	5655.2	196	19600.0	3.47	1.79	1526	1589.6	10	1000.0	0.629	-0.669	3.6	19.6	5.5	2.46	17
22	1	AF647	AF488	1.09	0.08	1197	1098.2	301	3762.5	3.43	1.78	1244	1141.3	109	1362.5	1.194	0.256	1.0	2.8	2.9	1.52	18
12	1	AF647	AF488	0.59	0.04	1224	2074.6	284	7100.0	3.42	1.78	1887	3198.3	23	575.0	0.180	-2.476	0.6	12.4	19.0	4.25	19
1	1	AF647	AF488	0.93	0.15	6600	7096.8	3162	21080.0	2.97	1.57	888	954.8	56	373.3	0.391	-1.355	7.4	56.5	7.6	2.93	20
3	1	AF647	AF488	0.55	0.13	3740	6800.0	2493	19176.9	2.82	1.50	691	1256.4	70	538.5	0.429	-1.222	5.4	35.6	6.6	2.72	21
10	2	AF488	AF647	0.93	0.03	365	392.5	31	1033.3	2.63	1.40	5260	5655.9	135	4500.0	0.796	-0.330	0.1	0.2	3.3	1.73	22
26	3	AF647	AF488	1.03	0.15	1131	1098.1	376	2506.7	2.28	1.19	325	315.5	47	313.3	0.993	-0.010	3.5	8.0	2.3	1.20	23
6	2	AF488	AF647	0.92	0.11	3738	4063.0	1002	9109.1	2.24	1.16	1746	1897.8	128	1163.6	0.613	-0.706	2.1	7.8	3.7	1.87	24
16	2	AF488	AF647	0.57	0.03	2086	3659.6	233	7766.7	2.12	1.09	2271	3984.2	114	3800.0	0.954	-0.068	0.9	2.0	2.2	1.15	25
5	2	AF488	AF647	0.55	0.06	2021	3674.5	464	7733.3	2.10	1.07	264	480.0	27	450.0	0.938	-0.093	7.7	17.2	2.2	1.17	26
30	3	AF647	AF488	2.24	0.14	1083	483.5	138	985.7	2.04	1.03	230	102.7	4	28.6	0.278	-1.846	4.7	34.5	7.3	2.87	27
4	1	AF647	AF488	0.73	0.12	2766	3789.0	734	6116.7	1.61	0.69	490	671.2	20	166.7	0.248	-2.010	5.6	36.7	6.5	2.70	28
24	3	AF647	AF488	0.86	0.06	843	980.2	76	1266.7	1.29	0.37	347	403.5	7	116.7	0.289	-1.790	2.4	10.9	4.5	2.16	29
7	2	AF488	AF647	0.66	0.03	2582	3912.1	101	3366.7	0.86	-0.22	2690	4075.8	7	233.3	0.057	-4.127	1.0	14.4	15.0	3.91	30

Summary: n = 30 cells (old LPS-647, new LPS-488 = 20; old LPS-488, new LPS-647 = 10), median $\log_2(S_{old\ LPS})$ = 2.07, IQR $\log_2(S_{old\ LPS})$ = 0.24 to 3.21, median $\log_2(S_{new\ LPS})$ = -0.55, IQR $\log_2(S_{new\ LPS})$ = -0.02 to 0.32, median $\Delta\log_2 S(old\ LPS - new\ LPS)$ = 2.84

Supplementary References

1 Datsenko, K. A. & Wanner, B. L. One-step inactivation of chromosomal genes in Escherichia coli K-12 using PCR products. *Proc. Nat. Acad. Sci. USA* **97**, 6640-6645 (2000).

2 Baba, T. *et al.* Construction of Escherichia coli K-12 in-frame, single-gene knockout mutants: the Keio collection. *Mol. Syst. Biol.* **2**, 2006.0008 (2006).
