# Supplementary material for: Lipopolysaccharide insertion and vesicle mediated turnover drive growth independent outer membrane adaptation in Escherichia coli
Source: Nat Commun. 2026 Aug 21;17:9983. doi: 10.1038/s41467-026-76886-6 (PMC13590524; doi:10.1038/s41467-026-76886-6)
Supplement: Supplementary file 2 [file 41467_2026_76886_MOESM2_ESM.pdf]

Description of Additional Supplementary Files

File Name: Supplementary Movies 1-3

Description: (timestamps embedded) compiled from time-lapse 3D SIM2 imaging experiment lasting 30 minutes showing changes in the distribution of background LPS in lower OM (1), mid-cell (2) and upper OM (3) optical sections of live, growing and dividing E. coli BW25113 cells. The clustered distributions of background LPS are maintained in the upper and lower OMs with no evidence of background LPS becoming segregated in the polar regions of the OM over time. No increase in background LPS fluorescent signal was observed within the cell boundaries at mid-cell depth indicating background LPS is not undergoing internalisation followed by cytoplasmic recycling. Kdo-N3-containing LPS was fluorescently labelled via Cu+-free strain-promoted azide-(-N3)-alkyne cycloaddition (SPAAC) ensuring cell viability was preserved. Scale bars: 5.0 µm

File Name: Supplementary Movie 4

Description: Video sequence comparing changes in newly inserted and background LPS distributions and abundances in the OM of E. coli for the insertion-trapping simulation (model 1, left) and phase separation simulation (model 2, right). Video is composed of simulation snapshots at 5 min intervals from 0 – 120 min post-initiation of new LPS insertion. Magenta: Newly inserted LPS. Green: Background LPS. Black / vacant spaces: LPS-inaccessible OMP-occupied regions (as described for panel A). White circles: OMV biogenesis events arising in LPS-dense regions according to the OM buckling model. Scale bars: 0.5 µm.
