# Supplementary material for: Lipopolysaccharide insertion and vesicle mediated turnover drive growth independent outer membrane adaptation in Escherichia coli
Source: Nat Commun. 2026 Aug 21;17:9983. doi: 10.1038/s41467-026-76886-6 (PMC13590524; doi:10.1038/s41467-026-76886-6)
Supplement: Supplementary file 3 [file 41467_2026_76886_MOESM7_ESM.pdf]

nature portfolio

Corresponding author(s): Joe Nabarro, Christoph Baumann, Martin Fascione

Last updated by author(s): Jul 14, 2026

Reporting Summary

Nature Portfolio wishes to improve the reproducibility of the work that we publish. This form provides structure for consistency and transparency in reporting. For further information on Nature Portfolio policies, see our Editorial Policies and the Editorial Policy Checklist.

Statistics

For all statistical analyses, confirm that the following items are present in the figure legend, table legend, main text, or Methods section.

n/a	Confirmed	
☐	☒	The exact sample size (*n*) for each experimental group/condition, given as a discrete number and unit of measurement
☐	☒	A statement on whether measurements were taken from distinct samples or whether the same sample was measured repeatedly
☐	☒	The statistical test(s) used AND whether they are one- or two-sided<br>*Only common tests should be described solely by name; describe more complex techniques in the Methods section.*
☐	☒	A description of all covariates tested
☐	☒	A description of any assumptions or corrections, such as tests of normality and adjustment for multiple comparisons
☐	☒	A full description of the statistical parameters including central tendency (e.g. means) or other basic estimates (e.g. regression coefficient) AND variation (e.g. standard deviation) or associated estimates of uncertainty (e.g. confidence intervals)
☐	☒	For null hypothesis testing, the test statistic (e.g. *F*, *t*, *r*) with confidence intervals, effect sizes, degrees of freedom and *P* value noted<br>*Give P values as exact values whenever suitable.*
☒	☐	For Bayesian analysis, information on the choice of priors and Markov chain Monte Carlo settings
☐	☒	For hierarchical and complex designs, identification of the appropriate level for tests and full reporting of outcomes
☐	☒	Estimates of effect sizes (e.g. Cohen's *d*, Pearson's *r*), indicating how they were calculated

Our web collection on statistics for biologists contains articles on many of the points above.

Software and code

Policy information about availability of computer code

Data collection	Commercial software:<br>ZEISS ZEN Black 3.0 SR FP2: Microscope control & acquisition for dSTORM; SMLM localisation processing, dSTORM channel localisation channel export and SIM^2 reconstructions<br>ZEISS ZEN Black 3.0 SR (SIM^2 module): 3D-SIM^2 z-stack processing (SIM^2 weak defaults)<br>ZEISS ZEN Blue 3.1: Generation of depth-coded 3D images<br>OriginPro 2024b (64-bit): Graph generation<br>ZetaView Software v8.06.01 SP1 (Particle Metrix): NTA video analysis and particle tracking for OMV quantification<br>Open source software:<br>FIJI / ImageJ v1.54h (with Bio-Formats plugin): Representative image preparation; SIM^2 cross-section generation (upper OM, mid-cell and lower OM).
Data analysis	Commercial Software:<br>Zeiss ZEN Black 3.0 (SR): SMLM processing of dSTORM timeseries, localisation fitting, grouping and export of individual channel localisation tables (.csv format) for downstream analyses.<br>GraphPad Prism v10.3.1: Non-linear regression of outer membrane vesicle size histograms (log-normal fits).<br>OriginPro 2024b (64-bit): Collation of per-cell outputs, generation of population-level summaries and graphs.<br>ZetaView software v8.06.01 SP1 (Particle Metrix): Nanoparticle Tracking Analysis video processing and particle size / concentration calculations.<br>MATLAB R2024b (64-bit): Execution environment for custom analysis scripts (see Custom code).

Python 3.10.12 with NumPy 1.26.0, shapely 2.0.3, scikit-learn 1.5.0: Particle-based outer-membrane simulations (insertion-trapping vs. phase-separation), DBSCAN-based cluster-mask generation for newly inserted and background LPS, DICE overlap, and the coverage-normalised zero-lag overlap statistic g_NB(0). Simulation DBSCAN parameters matched to experiment (eplison = 30 nm, minPts = 7).
Custom Code (application described in methods, archived with README, inputs and outputs or presented in Extended Data):
MATLAB:
LptD_new_LPS_crosscorrelation: Two-colour radial cross-correlation g(r) between LptD and newly inserted LPS, PCA alignment, null randomisation, summary metrics and per-cell exports
LptD_LPS_Demograph_Generator: Demograph generation (25 nm bins), per-cell normalisation and centre alignment.
calculate_photon_weighted_PCCs: Photon-weighted Pearson correlation coefficients (regional and whole outer membrane).
MATLAB_DBSCAN_Background_or_New_LPS_dSTORM_individual_cell_code, new_lps_or_old_LPS_cluster_summary_graphs_code.txt, MATLAB_DBSCAN_Background_New_LPS_clusters_by_region: Half-rod geometry projection, DBSCAN (epilson = 30 nm, minPts = 7), individual cluster areas and density calculation, outer membrane region resolved maps.
Python:
Python_code_New_Old_LPS_DBSCAN: Two-colour DBSCAN masks, DICE and g_NB(0) calculations
Python_code_insertion_trapping_model_1: Insertion-trapping outer membrane simulation.
Python_code_phase_seperation_model_2: Phase separation outer membrane simulation

For manuscripts utilizing custom algorithms or software that are central to the research but not yet described in published literature, software must be made available to editors and reviewers. We strongly encourage code deposition in a community repository (e.g. GitHub). See the Nature Portfolio guidelines for submitting code & software for further information.

Data

Policy information about availability of data

All manuscripts must include a data availability statement. This statement should provide the following information, where applicable:

- Accession codes, unique identifiers, or web links for publicly available datasets
- A description of any restrictions on data availability
- For clinical datasets or third party data, please ensure that the statement adheres to our policy

All custom MATLAB and Python scripts used for dSTORM image analysis, spatial cross-correlation, cluster analysis, data interrogation and particle-based simulations are publicly available in the Persistent_LPS_insertion_OM_OMVs GitHub repository. The version used in this study is release v1.0.2 and has been permanently archived in Zenodo at https://doi.org/10.5281/zenodo.21326870. The release includes README and alias files, example input data, and representative analysis outputs required to run and interpret the code. Numerical inputs and outputs underlying the figures are also provided in the accompanying Figure Source Data and Analysis Workbook. The code is released under the MIT License. GitHub repository: https://github.com/joenabarro-OM/Persistent_LPS_insertion_OM_OMVs, Release v1.0.2: https://github.com/joenabarro-OM/Persistent_LPS_insertion_OM_OMVs/releases/tag/v1.0.2 Zenodo archive: https://doi.org/10.5281/zenodo.21326870

Research involving human participants, their data, or biological material

Policy information about studies with human participants or human data. See also policy information about sex, gender (identity/presentation), and sexual orientation and race, ethnicity and racism.

Reporting on sex and gender	N/A
Reporting on race, ethnicity, or other socially relevant groupings	N/A
Population characteristics	N/A
Recruitment	N/A
Ethics oversight	N/A

Note that full information on the approval of the study protocol must also be provided in the manuscript.

Field-specific reporting

Please select the one below that is the best fit for your research. If you are not sure, read the appropriate sections before making your selection.

☒ Life sciences ☐ Behavioural & social sciences ☐ Ecological, evolutionary & environmental sciences

For a reference copy of the document with all sections, see nature.com/documents/nr-reporting-summary-flat.pdf

Life sciences study design

All studies must disclose on these points even when the disclosure is negative.

Sample size

No formal statistical method was used to predetermine sample size. Sample sizes were selected based on established SMLM, SIM and nanoparticle tracking practice, pilot variability and the requirement to obtain reproducible cell-level estimates across independent biological replicates. For single-colour pulse–chase dSTORM time courses, at least 10 cells were analysed per time point in each of three independent

biological replicates, giving at least 30 cells per time point overall, with additional cells analysed at many time points. LptD*–new LPS spatial analyses included 30 cells across three independent biological replicates. Regional DBSCAN analyses included at least 10 cells per time point, with exact sample sizes provided in the corresponding figure legends and Figure Source Data and Analysis Workbook. NTA measurements comprised three technical measurements per sample, each sampling 11 positions over 60 frames under standardised acquisition settings. Growth curves included seven replicate cultures. The selected sample sizes produced reproducible trends across independent experiments, sufficiently precise cell-level estimates and consistently high-quality model fits for the effects examined.

Data exclusions

No acquired biological replicate or analysed cell was excluded from the reported analyses. Samples in which technical errors during preparation or imaging prevented acquisition did not generate analysable data and were not counted as experimental replicates. Localisation-level filtering was performed using predefined quality criteria described in the Methods and was applied uniformly across all datasets.

Replication

All planned biological replicates reported in the study were completed successfully and included in the analyses. Key experiments were performed across three independent biological replicates unless otherwise stated, with predefined per-cell sampling and technical replication where appropriate. For the single-colour pulse–chase dSTORM datasets, each time course was performed independently three times, with typically at least 10 cells analysed per time point in each biological replicate, giving at least 30 cells per time point overall and more at many time points. Per-replicate trajectories for background-LPS loss and new-LPS accumulation, together with summary values reported as mean ± s.e.m., are provided in Supplementary Fig. 3 and Supplementary Tables 16–17.

For the two-colour LptD*–new-LPS spatial analyses, 30 cells were analysed across three independent biological replicates. Per-cell spatial cross-correlation, g(r), and Pearson correlation coefficient measurements were calculated, with full mean ± s.e.m. g(r) profiles and regional statistical comparisons provided in Supplementary Tables 10–11. Reciprocal fluorophore-swap experiments produced closely comparable spatial-correlation profiles, supporting that the observed relationships were not dependent on fluorophore assignment.

Acquisition and primary processing were standardised across all biological replicates, using identical ZEN SMLM fitting, grouping and filtering settings, PCA-based cell alignment and fixed image-pixel dimensions. DBSCAN parameters (ε=30 nm; minimum points = 7) were predefined from localisation precision and nearest-neighbour distributions and applied unchanged across experimental datasets and simulation comparisons. The associated scripts, example inputs and analysis outputs are provided to enable independent rerunning of the analyses.

The principal single-cell microscopy findings were also supported by orthogonal measurements. The loss of background LPS and accumulation of newly inserted LPS observed by dSTORM were consistent with biochemical measurements of extracted LPS by Tricine SDS–PAGE, while live-cell 3D-SIM^2 time courses independently confirmed the persistence of discrete outer-membrane LPS regions without detectable intracellular accumulation. FRAP controls further showed that Kdo-azide and Kdo-alkyne labelling produced comparable LPS mobility measurements.

For nanoparticle-tracking analysis of OMVs, the instrument was calibrated using verified 100-nm standards, and each sample was measured in three technical replicates. Each measurement sampled 11 positions over 60 frames using fixed acquisition and analysis thresholds, with at least seven positions required to meet the predefined inclusion criteria. These procedures produced consistent OMV concentration and size estimates, and particle-size distributions were analysed using the same log-normal fitting procedure for all samples.

Randomization

Samples were allocated to predefined experimental groups according to bacterial strain, metabolic-labelling condition, fluorophore label position/type, treatment and pulse–chase time point. Random allocation was not applicable because the groups were determined by the experimental design rather than by assigning individual subjects to interventions. Potential experimental covariates were controlled using matched culture media, incubation conditions, growth-phase criteria, labelling procedures, acquisition settings and analysis pipelines. Comparative samples were processed in parallel wherever practicable, and identical inclusion criteria and predefined analysis parameters were applied across groups. Reciprocal dye assignments were used where relevant to control for fluorophore-dependent effects.

Blinding

Operator blinding during microscopy acquisition was not feasible because the experimental condition and time point had to be known to perform the pulse–chase and time-course procedures. For quantitative dSTORM analysis, exported per-cell localisation files were duplicated, assigned random alphanumeric identifiers and placed in a neutral folder structure that contained no condition or time-point information. Per-cell quantitative metrics were generated and saved while file identity was blinded. Files were unblinded only after these measurements had been finalised, before group-level plotting and statistical comparison. Identical SMLM fitting and filtering settings and predefined downstream analysis parameters, including DBSCAN parameters, were applied across datasets. Other assays were not formally blinded, but fixed acquisition protocols and standardised or automated analysis procedures were used to minimise operator bias.

Reporting for specific materials, systems and methods

We require information from authors about some types of materials, experimental systems and methods used in many studies. Here, indicate whether each material, system or method listed is relevant to your study. If you are not sure if a list item applies to your research, read the appropriate section before selecting a response.

Materials & experimental systems

n/a	Involved in the study
x	Antibodies
x	Eukaryotic cell lines
x	Palaeontology and archaeology
	x Animals and other organisms
x	Clinical data
x	Dual use research of concern
x	Plants

Methods

n/a	Involved in the study
x	ChIP-seq
x	Flow cytometry
x	MRI-based neuroimaging

Animals and other research organisms

Policy information about studies involving animals; ARRIVE guidelines recommended for reporting animal research, and Sex and Gender in Research

Laboratory animals	Escherichia coli K-12 substr. BW25113. E. coli BW25113 / pBADcLIC-lptD*/ pEVOL-pylRS. E coli BW25113 ompA- / pBADcLIC-ompA* / pEVOL-pylRS
Wild animals	N/A
Reporting on sex	N/A
Field-collected samples	N/A
Ethics oversight	University of York

Note that full information on the approval of the study protocol must also be provided in the manuscript.

Plants

Seed stocks	N/A
Novel plant genotypes	N/A
Authentication	N/A
