# Supplementary material for: Lipopolysaccharide insertion and vesicle mediated turnover drive growth independent outer membrane adaptation in Escherichia coli
Source: Nat Commun. 2026 Aug 21;17:9983. doi: 10.1038/s41467-026-76886-6 (PMC13590524; doi:10.1038/s41467-026-76886-6)
Supplement: Supplementary file 4 [file 41467_2026_76886_MOESM8_ESM.pdf]

nature portfolio

# Peer Review File

# Lipopolysaccharide insertion and vesicle mediated turnover drive growth independent outer membrane adaptation in Escherichia coli

Corresponding Author: Dr Joe Nabarro

**This file contains all reviewer reports in order by version, followed by all author rebuttals in order by version.**

Version 0:

Reviewer comments:

Reviewer #1

(Remarks to the Author)
In this work the authors use a stablished technique that allows the modification of Lipid A so that it can be followed by super resolution microscopy to first determine the insertion sites of new LPS molecules into the OM, finding insertion in patches distributed along the cell surface that correlated with the presence of the LPS assembly protein LptD. To determine the time dependent segregation of the inserted LPS the authors use two substrates modifiable by different chemistries in a pulse chase strategy, followed by labelling with two colors. This experiment showed that the two populations of LPS did not mix, in accordance with previously reported limited diffusion of the LPS and further supporting the insertion in random patches of the outer membrane. From these results the authors decide to examine the turnover of LPS molecules during exponential and stationary culture growth phases. From their results the authors conclude that LPS synthesis continues into stationary phase maintaining the separation between old and new LPS. The authors follow the old LPS in a single cell time lapse experiment under physiological conditions and find that the old LPS is not internalized and is not maintained at the poles, suggesting to them that is not being partitioned by cell growth and division. To explain the apparent contradiction between continuous LPS synthesis together with the disappearance of old LPS even in the absence of cell growth, the authors show that the old LPS is being preferentially lost in outer membrane vesicles.
The work presented in this manuscript is technically well done and the experiments related with the initial insertion and separation of LPS “populations” seems very sound. The following experiments regarding the continued synthesis and loss of LPS in OMV needs further work and extensive controls. I would suggest to the authors to split this work as necessary to better show the experiments that support their ideas and to better discuss previous works.
Given the extension of the manuscript many of the methodological strategies are hard to follow, it would be useful to include some of these details
The authors use of the “phase separation” idea to describe the absence of noticeable LPS mixing is confusing, explain how this idea is originally used and include the citations used after (line 276).
In reference 21 fusion of LPS patches is shown, in accordance with the phase separation model, please discuss this difference.
From lines 192 to 205 the authors describe again the absence of LPS diffusion by determining the overlapping regions between old and new LPS. As expected from the known low diffusion of the LPS, as the new one appears the overlap increases until the old one starts diluting by cell growth and the overlap diminishes. The authors decide to call this absence of diffusion the “insertion-trapping” mechanisms. However, there is no investigation into the mechanism, nor is this new or unexpected behavior.
In section that starts in line 217: The use of a similar technique to track the synthesis and insertion of new LPS has been recently reported, please cite and discuss 1
In figure 3B, there is a big change in the “background LPS loss” and the rate of “new LPS insertion” does not seem constant as the straight line suggests.
Line 225, the authors say that they followed LPS insertion and loss in a cell-by-cell basis, does this mean following single cells in time or more likely from the methods supplementary section determined in individual cells in a population?
The authors assume that the rate of appearance or loss of the label correlates with LPS synthesis. There is no proof that this is the case, much less in the transition to stationary phase. It has been shown that this technique differentially labels smooth and rough LPS and that the labelling is affected by the synthesis of Kdo by the cells, does any of these factors change during the different growth phases?

The culture conditions are not standard, startin a culture with an OD of 0.5 reduces the amount of available oxygen and reching stationary phase after just 1.5 hours is not usual. Could this be a factor influencing the production of OMVs?
Lines 277-290 Phase separation has been used in terms of protein rich and protein less domains in the OM, not in terms of LPS diffusion
Is conclusion of lines 291-301 not the same as above?
Lines 304-310, the old LPS has not been shown to be lost, only the signal coming from it. Introducing the idea of losing the LPS at this point seems leading to the conclusions that the authors are trying to probe.
Fig 4 C the number of clusters suddenly decreased between 60 and 90 min, probably coinciding with cell division?
It is not clear if the data presented in Fig. 5 is enough to discard internalization of LPS. Controls should be shown.
Lines 337-338. Why would the absence of polar concentration of LPS be evidence that binary partition is not occurring? The absence of old polar LPS could be caused by insertion of new LPS at the poles, as is suggested by this and other articles.
OMV are not made of only LPS, they normally contain the same proteins present in the OM, the authors seem to assume that the OMVs produced have no proteins and do not show if evidence of this.
How much LPS is really lost in the OMVs? Is this consistent with the synthesis of “excess” LPS.
OMV from other E. coli strains produce smaller OMVs than those observed in this work.
Reference 27 seems to be wrong
Reference 11 seems to be of a still not accepted manuscript; I am not sure this is acceptable and I would at least mention it the text.

(Remarks on code availability)

Reviewer #2

(Remarks to the Author)

Nabarro et al. investigated the spatiotemporal dynamics of LPS in the bacterial outer membrane of E. coli. For this purpose, LPS and the LPS transporter LptD were fluorescently labelled using sophisticated click chemistry techniques. Labelled LPS was recorded in fixed and living cells and in pulse-chased experiments with different super-resolution microscopy techniques including dSTORM and SIM. The results achieved are of very high quality and spectacular in some respects. A number of conclusions were drawn that will help to change our view of LPS metabolism and -dynamics. E.g., E. coli rearranges its outer membrane (OM) through continuous removal of pre-existing LPS and insertion of new LPS at discrete sites. Also, E. coli LPS in the OM is cleared by release through OM vesicles suggesting that this is the major way how Gram-negative bacteria remodel their surface architecture independent of their growth phase.

Major points

1. All images showing LPS and LptD rely solely on click-chemistry fluorescence labeling of either incorporated functionalized Kdo or modified LptD. Even though these methods are highly efficient, incomplete or unspecific labelling could compromise the results. Can the authors provide an independent depiction of LPS and LptD not requiring click-labelling, e.g., using antibody staining ? The antibody-stained and click-labelled LPS or LptD could then be checked for overlaps. Further, can the authors present data of wild type E. coli not treated with functionalized Kdo but only incubated with the fluorescent azide and alkyne compounds, demonstrating that the latter do not cause background/unspecific staining ?

2. The spatiotemporal relation of LptD and LPS suggest a functional relation, also when previous published work is considered. However, the data not provide proof that LptD under these conditions really affect LPS insertion or dynamics. Is it possible to (partly) inhibit LptD to see whether this affects LPS localization and/or dynamics?

Minor points

1. The elegant labelling system using click chemistry might be hard to understand for readers not familiar with this system. It could e.g., help to consistently use one nomenclature throughout the whole manuscript (e.g. avoid using sometimes N3 and sometimes azide). This includes “Keys” of Figures 1A and 2A.

2. LptD* is expressed from a vector within E. coli bacteria that still express endogenous LptD. Thus, there is likely an additional endogenous pool of LptD in the bacteria. To which extent might this influence the results, considering that only the vector-expressed LptD pool is shown? Also, could the presumably increased amount of LptD in the bacteria lead to changes in LPS insertion dynamics?

2. The authors show that preferentially background LPS is enriched in OMVs for LPS removal. Is there any data or are there any ideas on how the bacteria distinguish between new and background LPS? If yes it could be added to the discussion.

Figure S1A: Please indicate that you switched the dyes for both targets in the figure, not only by just switching the colors in the figure (for example by indicating the dyes that were used below the target).
Line 118/119: Please explain what the abbreviation BAM stands for.
Line 161/162: Figure numbering appears not correct, is Fig S2A meant?
Figure 2A; Key: Symbol for Kdo-azide is missing.

Line 234: Why would photobleaching be an issue here or matter at all and not in the other experiments? The same number of frames should be also acquired in all experiments for maintaining comparability throughout the manuscript. While Figure 3A shows a clear picture, the limited number of 5000 frames could theoretically result in a general underrepresentation of LPS.
Figure 5; Mid cell: Could you choose a different color for the images here as blue might not be visible well for the human eye.
Figure S5A-C: A timestamp should be inserted in the live-SIM movies.
Line 336/Figure 5: While the mentioned live-cell observations are consistent with the dSTORM data, can also the decrease of background LPS over time be observed in live-SIM in longer recordings?

(Remarks on code availability)

Reviewer #3

(Remarks to the Author)
This manuscript by Nabarro et al. uses super-resolution microscopy to identify that turnover of LPS in the outer membrane of E. coli is facilitated by the insertion of new LPS at discrete patches across the entire outer membrane and specifically release of old LPS in outer membrane vesicles. This turnover is shown to proceed well into stationary phase and is thus independent of cell growth, which challenges the null hypothesis that older LPS is passively diluted by the insertion of new LPS in a growth dependent manner.

The strength of the manuscript is the quality of the data. Outer membrane dynamics are an important aspect of bacterial physiology and the data reported here describe the relationship of these dynamics to cell growth than previous studies. The main weakness of the manuscript is that something very similar to the central model they propose was previously reported, and this study is not cited or discussed. As far as I know, the vesiculation during stationary phase is a novel result.

Major Comments:
-The authors should read, cite, and discuss the model presented by Ursell et al (doi:10.1371/journal.pcbi.1002680), which was based on a mathematical analysis of experimental data, similar to the present study. I believe that the “insertion trapping” model is essentially the same as the one presented there even if the resolution of the data in the current study is higher. Therefore, it would be helpful if the authors could present the previous model in the introduction, build on it (if possible) in the main text, and compare the two models in the discussion. There may be quantitative differences or advances that the current study could offer in light of the higher resolution. There may also be slightly different conclusions that arise due to differences in the mathematical models.

-Similarly, the authors propose liquid-liquid “phase separation” as a hypothetical organizing principle of the outer membrane to contrast their model, but despite the observation of limited separation of OMPs and LPS (Benn et al. PNAS 2021), it is well understood that the outer membrane is a growing solid material (neither OMPs or LPS diffuse), and therefore this seems to be a strawman argument, especially in light of the Ursell study.

-Figure 6: The vesiculation data is nice and potentially novel, but there is no quantitative assessment of it:

>How many vesicles per cell per unit time occur during stationary phase?
>Do this vesiculation rate quantitatively account for the rate of LPS loss as observed by the metabolic labeling?
>I don’t see any statistics of replicates or # of cells for Figures 6A-C

Minor comments:
-Figure 1E: Cells do not have right and left. Just use polar and non-polar.

-Figure 2: The analysis of “overlap” is not very informative with respect to physiological function. With STORM-level resolution, we expect very little overlap since LPS cannot actually be made on top of other LPS, so the only overlap comes from the noise in the measurement. I believe what we would like to understand is the dynamics of LptD/new LPS patches. How long is a single “patch” made for before the synthesis machinery moves on to synthesize a new patch, and how big do the patches get during this time?

-Line 305: “Kinetically-trapped mosaic of spatially segregated domains.” This is an overly convoluted statement. In biology, domains usually imply that there is something different about the domains. From the present analysis, there are no functional domains. The analysis reinforces the current understanding of the outer membrane as a growing solid – is there any difference between “kinetically trapped” and “solid”?

-Line 332: Replace “electromagnetic radiation” with “light”.

-Figure 3B vs 6F: stationary phase is defined very differently. Since it is an important concept in the model, it would be good to provide a single working definition of it and analyze all the data accordingly.

(Remarks on code availability)

Reviewer #4

(Remarks to the Author)
Christoph Baumann and collaborators present a communication titled "Persistent LPS insertion, spatial segregation and vesicle biogenesis drive growth-independent adaptation of the Escherichia coli outer membrane".

This manuscript presents the use of chemical biology tools, namely metabolic lipopolysaccharide labeling and genetic code expansion, to study the dynamics of lipopolysaccharide insertion, diffusion and release within the Outer Membrane of Gram-negative bacteria, using Escherichia coli as a relevant model and Super Resolution Microscopy (dSTORM) for nanometric imaging.

This very thorough and well conducted study combines all the available tools, including mathematical modeling, to better understand these processes which are fundamental to the study of Gram-negative bacteria, with potential long-range impact in terms of anti-bacterial strategies.

The authors show convincing evidence that LPS is inserted into the membrane in well-localized sites, that LPS does not diffuse laterally within this membrane, that new and old LPS are segregated locally within different patches, and that older LPS is released from the membrane via localized formation of Outer Membrane Vesicles, ensuring constant LPS renewal even when the bacterium is not elongating or dividing.

This study is very convincing, and, I believe, should be accepted in Nature Communications as soon as some minor remarks have been addressed.

The main concern relates to experiments depicted in Figure 1 and the "simultaneous New LPS &LptD* in situ fluorophore conjugation". The caption states: "Simultaneous CuAAC 'click' chemistry enabled orthogonal fluorescent tagging of LptD* and newly inserted LPS using azide- and alkyne-functionalised dye pairs". Strictly simultaneous CuAAC with the 2 fluorophores does not seem feasible, since the fluorophore-azide should react with the fluorophore alkyne. The supplementary Methods are not clear either on this aspect: "The washed cell pellet was then resuspended to an OD600 of 1.0 in 'Click-iT' mix charged with either AF488- or AF647-alkyne, transferred to a 2 mL microcentrifuge tube, and incubated at room temperature for 30 min on a rotary wheel (12 rpm, 80° incline relative to benchtop) protected from light." – Only AF488- or AF647-alkynes are mentioned, no azide and no sequential process – Could the authors clarify? Were these fluorophore conjugations realized stepwise? This is very important to describe a very clear protocol here.
The same applies to the experiment depicted in figure 2.

I am not an expert, but I thought that during the stationary phase, bacteria would continue dividing, but their division would be counter-balanced by bacterial death, limiting population growth. Do bacteria also stop dividing or slow down their division rate? Could the author add a short comment on this?

Figure 7, in the Particle-based simulations, what is the meaning of Black/vacant spaces? There should be some phospholipids present in that space, right?

Line 556: "a LPS molecule" instead of "an LPS molecule".

Some references are missing:
- For Kdo-N3 labeling of E. coli via SPAAC - https://doi.org/10.1371/journal.pone.0127700
- Molecular proof of Kdo-N3 incorporation in the LPS inner-core: https://doi.org/10.1074/jbc.M117.814962
- Supreresolution Imaging of E. coli, after metabolic lipopolysaccharide labeling using Kdo-N3: https://doi.org/10.1038/s41467-019-09901-8

(Remarks on code availability)

Reviewer #5

(Remarks to the Author)
I co-reviewed this manuscript with one of the reviewers who provided the listed reports. This is part of the Nature Communications initiative to facilitate training in peer review and to provide appropriate recognition for Early Career Researchers who co-review manuscripts.

(Remarks on code availability)

Version 1:

Reviewer comments:

Reviewer #2

(Remarks to the Author)
1. We asked for a simple control experiment where fixed bacteria should in addition to click-labelling be stained with antibodies against LPS or LptD to check whether and how either of these components when antibody-stained overlaps with its respective click labelled component. We did not ask for LPS and LptD colabelling, and therefore the effect of staining of one of these components cannot affect the localization or function of the other component?! Also, a linkage error of at most 25 nm (given that primary and secondary antibody are used), which could also be reduced to about 10nm in the case of primary antibody and secondary nanobody are used, will not have a significant impact when checking the overlap of the two signals (click-labelled and antibody labelled). Further, even if antibody might in some instances not bind to "shielded" antigen, every antibody-stained antigen should overlap with the corresponding click labelled antigen. The authors also argue that there are no working antibodies against LptD. Have they e.g., tried to get a hold of the  -LptD-AF488 Fabs as described in PMID: 31237236 and 40030021.

2. The authors have satisfactorily answered this point. In Fig. S15H they should correct the sequence of the single figures in the panel.

3.-10. The authors have satisfactorily answered these point and altered the ms accordingly.

11. The authors claim that the requested experiment, namely the visualization of the decrease of the background LPS is not possible by SIM because of faster photobleaching. However, they could decrease the temporal and/or spatial resolution in the experiment, for example by using a different microscopy technique, spinning disk, confocal, wide field etc. The mere decrease of background LPS should be detectable with any of these techniques and super high resolution is not really necessary for this.

(Remarks on code availability)

Reviewer #3

(Remarks to the Author)
The authors addressed each my concerns. This is a nice study that will be impactful in bacterial cell biology field.

(Remarks on code availability)

Reviewer #4

(Remarks to the Author)
I am pleased to note that the authors have taken the reviewers' comments, suggestions, and remarks extremely seriously. In my assessment, all the points I raised have been addressed in a thorough and convincing manner. The revisions reflect a thoughtful and rigorous response to the feedback provided.
I also evaluated the responses to Reviewer 1, and all concerns were similarly resolved. While the rebuttal does not explicitly discuss the comment regarding lines 192–205 of the original manuscript, the corresponding text has been amended to address it.
Based on the quality of the revisions, I now recommend that the manuscript be accepted for publication.

(Remarks on code availability)

Reviewer #5

(Remarks to the Author)
I co-reviewed this manuscript with one of the reviewers who provided the listed reports. This is part of the Nature Communications initiative to facilitate training in peer review and to provide appropriate recognition for Early Career Researchers who co-review manuscripts.

(Remarks on code availability)

Version 2:

Reviewer comments:

Reviewer #2

(Remarks to the Author)

I originally was not satisfied with answers to points 1 and 11 but I think these issues have been solved now.

1. Click labelling and antibody staining for LPS and/or LptD in parallel, to serve as a control for the click labelling:
The authors did not succeed to get valid data out of these experiments, but they apparently put in considerably effort and contacted various external parties for materials. They also strengthened their arguments that click labelling is in fact showing the physiological location of LPS. Thus, this issue is settled to my opinion and antibody-costaining simply cannot be accomplished within a reasonable timeframe because suitable antibodies appear not to exist.

11. Demonstrate the reduction in LPS background using SIM or another lower-resolution microscopy technique:
The authors have adjusted their SIM imaging and can now observe the reduction in background LPS using Live-SIM. These data are fine because they show a trend towards reduction of LPS and this point is settled to my opinion.

(Remarks on code availability)

**Open Access** This Peer Review File is licensed under a Creative Commons Attribution 4.0 International License, which permits use, sharing, adaptation, distribution and reproduction in any medium or format, as long as you give appropriate credit to the original author(s) and the source, provide a link to the Creative Commons license, and indicate if changes were made.
In cases where reviewers are anonymous, credit should be given to 'Anonymous Referee' and the source.
The images or other third party material in this Peer Review File are included in the article's Creative Commons license, unless indicated otherwise in a credit line to the material. If material is not included in the article's Creative Commons license and your intended use is not permitted by statutory regulation or exceeds the permitted use, you will need to obtain permission directly from the copyright holder.
To view a copy of this license, visit https://creativecommons.org/licenses/by/4.0/

**REVIEWER COMMENTS**

**Reviewer #1 (Remarks to the Author):**

In this work the authors use a stablished technique that allows the modification of Lipid A so that it can be followed by super resolution microscopy to first determine the insertion sites of new LPS molecules into the OM, finding insertion in patches distributed along the cell surface that correlated with the presence of the LPS assembly protein LptD. To determine the time dependent segregation of the inserted LPS the authors use two substrates modifiable by different chemistries in a pulse chase strategy, followed by labelling with two colours. This experiment showed that the two populations of LPS did not mix, in accordance with previously reported limited diffusion of the LPS and further supporting the insertion in random patches of the outer membrane. From these results the authors decide to examine the turnover of LPS molecules during exponential and stationary culture growth phases. From their results the authors conclude that LPS synthesis continues into stationary phase maintaining the separation between old and new LPS. The authors follow the old LPS in a single cell time lapse experiment under physiological conditions and find that the old LPS is not internalized and is not maintained at the poles, suggesting to them that is not being partitioned by cell growth and division. To explain the apparent contradiction between continuous LPS synthesis together with the disappearance of old LPS even in the absence of cell growth, the authors show that the old LPS is being preferentially lost in outer membrane vesicles.

The work presented in this manuscript is technically well done and the experiments related with the initial insertion and separation of LPS "populations" seems very sound. The following experiments regarding the continued synthesis and loss of LPS in OMV needs further work and extensive controls.

1. **I would suggest to the authors to split this work as necessary to better show the experiments that support their ideas and to better discuss previous works. Given the extension of the manuscript many of the methodological strategies are hard to follow, it would be useful to include some of these details**

**Response**

The editor [Redacted] has specifically instructed us not to split this work into separate manuscripts.

2. **The authors use of the "phase separation" idea to describe the absence of noticeable LPS mixing is confusing, explain how this idea is originally used and include the citations used after (line 276).**

**Response**

We revised the results section of the main text: **LPS-rich clusters remain spatially discrete and heterogeneously distributed, inconsistent with phase ordering in the OM** (lines 307 – 383),clearly defining 'phase separation' as compositionally distinct LPS-rich and OMP-rich domains, revising literature citations and clarifying that the discriminating prediction is domain coarsening, which we did not observe.

**Key text that addresses point**

**Lines 330 -340.** It has been proposed that organisation of the Gram-negative OM can involve phase separation or phase ordering, in which compositionally distinct regions (e.g., LPS-rich versus OMP-rich) emerge from thermodynamically favoured interactions under reduced membrane fluidity [33,41]. Here, we use 'phase separation' and 'phase ordering' in this compositional sense (rather than simply to denote restricted lateral diffusion). Because both LPS and OMPs exhibit very limited lateral mobility *in vivo*, any compositional ordering may be strongly diffusion-limited and therefore slow to relax on experimental timescales [1-3]. Nevertheless, an equilibrium-like ordering regime makes the clear, testable prediction that once compositionally distinct regions form, classical models of phase-ordering kinetics predict coarsening, in which smaller regions disappear while larger regions grow through coalescence and Ostwald ripening, yielding fewer, larger regions at later time points[10,11,32]. Nevertheless, an equilibrium-like ordering regime makes the clear, testable prediction that once compositionally distinct regions form, classical models of phase-ordering kinetics predict coarsening, in which smaller regions disappear while larger regions grow through coalescence and Ostwald ripening, yielding fewer, larger regions at later time points [42-48].

**Lines 360-364.** We observed no evidence of coarsening (in which smaller domains vanish as larger domains grow), as expected for Ostwald ripening or domain fusion [45-47]. For example, Benn *et al.* reported fusion and coalescence of LPS-rich patches under low-fluidity conditions compatible with an equilibrium-like phase-ordering regime [33,41]

**Lines 367-370.** This behaviour is compatible with a solid-like, diffusion-limited regime in which LPS-rich patches are stabilised rather than undergoing fusion-driven growth [10-13], arguing against equilibrium phase ordering as the dominant mechanism in our system.

**Lines 395-398** These results ***(Fig. 4 and Extended Data Fig. S4)*** argue against *E. coli* OM organisation being governed primarily by equilibrium phase ordering with domain coarsening. Instead, our data support a solid-like, diffusion-limited OM with persistent, insertion-patterned spatial heterogeneity..

### New relevant references


13 Ursell, T. S., Trepagnier, E. H., Huang, K. C. & Theriot, J. A. Analysis of Surface Protein Expression Reveals the Growth Pattern of the Gram-Negative Outer Membrane. *PLoS Comput. Biol.* **8**, e1002680 (2012).

40 Ester, M., Kriegel, H.-P., Sander, J. & Xu, X. in *Proceedings of the Second International Conference on Knowledge Discovery and Data Mining (KDD-96).* (ed E. Simoudis, Han, J. & Fayyad, U. M.) 226-231 (AAAI Press).

41 Gohrbandt, M. *et al.* Low membrane fluidity triggers lipid phase separation and protein segregation in living bacteria. *EMBO J.* **41**, e109800 (2022).

42 Elson, E. L., Fried, E., Dolbow, J. E. & Genin, G. M. Phase separation in biological membranes: integration of theory and experiment. *Annu. Rev. Biophys.* **39**, 207-226 (2010).

43 Bray, A. J. Theory of phase-ordering kinetics. *Adv. Phys.* **43**, 357-459 (1994).

44 Cugliandolo, L. F. Coarsening phenomena. *C. R. Phys.* **16**, 257-266 (2015).

45 Siggia, E. D. Late stages of spinodal decomposition in binary mixtures. *Phys. Rev. A* **20**, 595 (1979).

46 Lifshitz, I. M. & Slyozov, V. V. The kinetics of precipitation from supersaturated solid solutions. *J. Phys. Chem. Solids* **19**, 35-50 (1961).

47 Wagner, C. Theorie der alterung von niederschlägen durch umlösen (Ostwald-reifung). *Ber. Bunsenges. Phys. Chem.* **65**, 581-591 (1961).

48 Cahn, J. W. & Hilliard, J. E. Free energy of a nonuniform system. I. Interfacial free energy. *J. Chem. Phys.* **28**, 258-267 (1958).

**3. In reference 21 (now 33) fusion of LPS patches is shown, in accordance with the phase separation model, please discuss this difference.**

**Response**

We have added a comparison in the results section of the main text **LPS-rich clusters remain spatially discrete and heterogeneously distributed, inconsistent with phase ordering in the outer membrane** (lines 320– 406) explicitly mentioning the fusion/coalescence behaviour reported by Benn *et al.* (reference 33) and clarifying how this relates to our findings and interpretation.

**Key text that addresses point**

**Lines 360-370.** We observed no evidence of coarsening (in which smaller domains vanish as larger domains grow), as expected for Ostwald ripening or domain fusion [45-47]. For example, Benn *et al.* reported fusion and coalescence of LPS-rich patches under low-fluidity conditions compatible with an equilibrium-like phase-ordering regime [33,41]. In contrast, across our conditions and time window, both localisation-density-defined background and newly inserted LPS pools lacked any coarsening signature, indicating that LPS lateral mixing is strongly constrained and domain coalescence is suppressed. This behaviour is compatible with a solid-like, diffusion-limited regime in which LPS-rich patches are stabilised rather than undergoing fusion-driven growth [10-13], arguing against equilibrium phase ordering as the dominant mechanism in our system.

**Lines 395-404.** These results ***(Fig. 4 and Extended Data Fig. S4)*** argue against *E. coli* OM organisation being governed primarily by equilibrium phase ordering with domain coarsening. Instead, our data support a solid-like, diffusion-limited OM with persistent, insertion-patterned spatial heterogeneity. The Kdo-$N_3$-labelled background LPS pool (as measured by fluorescence/localisation density) progressively decreases without large-scale mixing or coarsening of LPS-rich regions ***(Fig. 4B)***, while newly inserted LPS accumulates in discrete, stable patches ***(Fig. 4A)***. This behaviour is consistent with insertion trapping, in which newly inserted LPS becomes effectively constrained upon incorporation rather than laterally redistributing to equilibrate the membrane. This restricted-mixing regime is directly observed for LPS [10-13,33]

**4. In section that starts in line 217: The use of a similar technique to track the synthesis and insertion of new LPS has been recently reported, please cite and discuss 1:**

**Response**

We have now cited and briefly discussed the recently reported Kdo-$N_3$ pulse–label/click strategy for tracking LPS synthesis and surface insertion in the section **Kdo-$N_3$ pulse–chase experiments reveal continuous insertion of new LPS and removal of background LPS in a growth-independent manner.** (lines 239 – 319).

**Key text that addresses point**

**Lines 244-254.** A related click-chemistry strategy was recently reported to estimate relative LPS synthesis rates in *E. coli* K-12 MG1655 by pulsing cells with Kdo-$N_3$ followed by copper-free click labelling and in-gel fluorescence quantification [14]. In that study, fluorescence microscopy outputs suggested that newly synthesised LPS initially appears as discrete surface foci at short pulse times, becoming more evenly distributed with longer pulses [14]. Here, we build on this strategy by coupling a pulse-chase design to single-cell dSTORM ***(Fig. 3, Extended Data Figs. S3A and S3B)*** and complementary population-scale biochemical readouts ***(Fig. 6D, Extended Data Fig. S6A and S6C)*** thereby enabling quantitative analysis of nanoscale new LPS insertion patterns and loss of pre-existing (background/old) LPS across the exponential-to-stationary phase of growth transition ***(Fig. 4 and Extended Data Fig. S4)***.

**Reference**

14 Shu, S. & Mi, W. Estimating the relative rates of lipopolysaccharide synthesis in Escherichia coli K-12 by click chemistry-mediated labeling. *PLoS One* **20**, e0325589 (2025).

**5. In figure 3B, there is a big change in the "background LPS loss" and the rate of "new LPS insertion" does not seem constant as the straight line suggests.**

**Response**

We have revised ***Figure 3B*** and ***Figure 3C*** (below) to show individual biological replicates alongside the replicate mean and to plot the mean trendline with a 95% confidence band, clarifying the fit and avoiding any implication of a strictly constant rate across the time course.

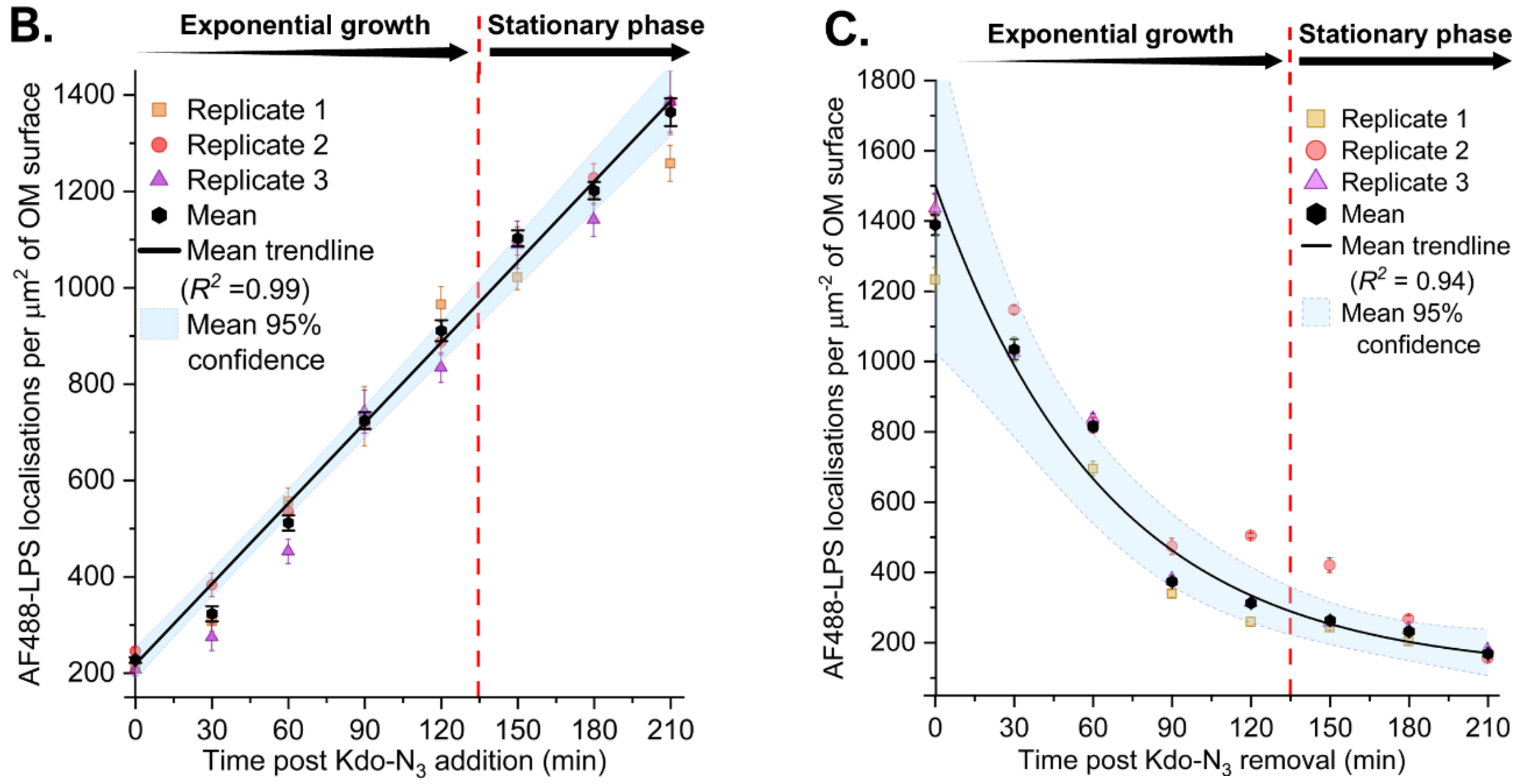


**Figure 3: Single-cell dSTORM Kdo-$N_3$ pulse–chase experiments reveal continuous insertion of metabolically labelled new LPS and removal of background LPS in a growth-independent manner.**

**B.** **Quantification of newly inserted (new), metabolically labelled LPS accumulation following Kdo-$N_3$ addition, plotted as AF488-LPS localisation density per µm² of OM surface area for individual biological replicates (coloured symbols) and the replicate mean (black hexagons).** The black line indicates the mean trendline with associated 95% confidence band (shaded); the red dashed line marks the approximate beginning of the transition from exponential growth to stationary phase (~135 min post Kdo-$N_3$ addition), derived from the $OD_{600}$ growth curve as the point where the increase in $OD_{600}$ begins to decelerate towards an asymptote ***(Extended Data Fig. S3C–D; Extended Data Tables S22–S23)***.

**C.** **Quantification of background, metabolically labelled LPS loss following Kdo-$N_3$ removal (chased with native Kdo), plotted as AF488-LPS localisation density per µm² of OM surface area for individual biological replicates (coloured symbols) and the replicate mean (black hexagons).** The black line indicates the mean trendline with 95% confidence band (shaded), the red dashed line marks the approximate beginning of the transition from exponential growth to stationary phase (~135 min post Kdo-$N_3$ addition), derived from the $OD_{600}$ growth curve as the point where the increase in $OD_{600}$ begins to decelerate towards an asymptote ***(Extended Data Fig. S3C–D; Extended Data Tables S22–S23)***.

For **B**. and **C**., n ≥ 30 cells per time point and N = 3 biological replicates per time point.

**6. Line 225, the authors say that they followed LPS insertion and loss in a cell-by-cell basis, does this mean following single cells in time or more likely from the methods supplementary section determined in individual cells in a population?**

**Response**

By cell-by-cell basis we mean that at each time point we quantified LPS signal in individual cells sampled from the population (rather than following the same single cells by time-lapse imaging), and we have adjusted the wording in this paragraph to make this explicit.

**Key text that addresses point**

**Lines 256-260.** We performed complementary pulse–chase experiments using Kdo-$N_3$ and native Kdo combined with dSTORM imaging. One experiment quantified accumulation of newly inserted LPS in the OM over time by analysing individual cells sampled from the population at each 30-minute time point, whilst the other quantified loss of pre-existing, background LPS from the OM of individual *E. coli* cells.

**7. The authors assume that the rate of appearance or loss of the label correlates with LPS synthesis. There is no proof that this is the case, much less in the transition to stationary phase. It has been shown that this technique differentially labels smooth and rough LPS and that the labelling is affected by the synthesis of Kdo by the cells, does any of these factors change during the different growth phases?**

**Response** (part 1). We agree that the appearance or loss of fluorescence reports the net appearance and removal of labelled LPS in the outer membrane (i.e., after biosynthesis and transport), and we therefore use localisation density (AF488-localisations per $\mu m^2$ of OM surface) as a proxy for relative OM insertion and turnover, rather than as an absolute readout of total LPS synthesis.

**Key text that addresses point Lines 267-283.** For these time-course measurements, we used identical imaging and processing settings across fixed acquisition window (5000 frames) at every time point, so that localisation density per OM area could be compared consistently as a relative proxy Kdo-$N_3$-containing, new LPS delivered to the OM. We note that a fixed 5000-frame acquisition may underrepresent absolute localisation yield, but this does not affect the comparability of localisation densities across the time course because every time point was acquired and processed under identical conditions. This fixed-window approach avoids time-dependent photophysical depletion and photobleaching effects that can compress dynamic range and bias comparisons across time points when acquisition length is varied or extended. Longer acquisitions were used elsewhere ***(Figs. 1 and 2)*** to maximise localisation yield for spatial patterning and colocalisation analyses, rather than for quantitative cross-condition density comparisons. Throughout, dSTORM localisation density ***(Fig. 3)*** and in-gel fluorescence ***(Figs. 6D and 6E)*** are used as relative proxies for the abundance of fluorescently labelled LPS; accordingly, we describe change as an increase (new LPS experiments) or decline (background/old) in metabolically- and fluorescently labelled LPS signal (newly inserted- or background LPS pool), rather than an absolute change in newly inserted / background LPS amount in cell OMs.

**Response** (part 2). The reported smooth-versus-rough differences in LPS labelling efficiency due to the increased steric hindrance associated with extended O-antigens of LPS is valid, however it is not applicable here because *E. coli* K-12 BW25113 is O-antigen deficient (produces rough LPS) as a consequence of well characterised mutations in the O-antigen biosynthetic locus in K-12 backgrounds (e.g., *rfb* and *wbbL*) and our TSDS-PAGE analyses show rough-LPS bands consistent with this genotype ***(Fig. S6C***, ***Extended Data Figs. S2C and S6C***, Stevenson *et al*., 1994). With respect to growth phase, although precursor pools and Kdo-pathway expression may vary, Kdo-$N_3$ was always supplied in large excess under defined conditions throughout, which should minimise sensitivity to changes in endogenous Kdo availability; moreover, the reproducible pulse–chase trends across biological replicates and the orthogonal bulk readouts suggest strongly that growth-phase-dependent incorporation efficiency is unlikely to explain the observed dynamics.

**Reference**


**i)** Stevenson, G. *et al.* Structure of the O antigen of *Escherichia coli* K-12 and the sequence of its **rfb** gene cluster. **J. Bacteriol.** *176*, 4144–4156 (1994). This study explicitly states E. coli K-12 has long been known not to produce O antigen, and identifies mutations in the rfb (O-antigen) gene cluster underlying the rough phenotype

**8. The culture conditions are not standard, starting a culture with an OD of 0.5 reduces the amount of available oxygen and reaching stationary phase after just 1.5 hours is not usual. Could this be a factor influencing the production of OMVs?**

**Response**

In our pulse–chase experiments, $OD_{600}$ = 0.5 denotes the mid-exponential phase at which metabolic labelling was initiated (monitoring new LPS insertion) or sampling was begun (monitoring background LPS turnover and new LPS vs background/old LPS OM distributions), not the inoculation density. All cultures were inoculated according to standard practice at $OD_{600}$ = 0.05 and grown under standardised, highly aerated conditions throughout. We initiated labelling at mid-exponential phase ($OD_{600}$ = 0.5) to ensure sufficient material for microscopy and biochemical analyses while keeping growth conditions and sampling consistent across replicates.

Importantly, OMV production was extensively quantified and characterised in separate assays starting from $OD_{600}$ = 0.05 ***(Fig. 6F, Extended Data Figs. S8, S11, S16 and S17)***, and the OMV production profile tracks the same exponential-to-stationary phase of growth transition, arguing against reduced oxygen availability at higher starting biomass as the primary driver of the observed OMV behaviour. Moreover, elevated rates of OMV production in *E. coli* haves been linked to significantly elevated envelope/oxidative stress physiology rather than moderately reduced oxygen availability. This is supported by multiple references (cited in manuscript)*:*

60 Schwechheimer, C. & Kuehn, M. J. Outer-membrane vesicles from Gram-negative bacteria: biogenesis and functions. *Nature Rev. Microbiol.* **13**, 605-619 (2015).

63 Kulp, A. J. *et al.* Genome-wide assessment of outer membrane vesicle production in Escherichia coli. *PLoS One* **10**, e0139200 (2015).

**Key text that addresses point**

**Lines 255 -257.** Cultures were inoculated at an $OD_{600}$ of 0.05 under standard shaking and aeration conditions; newly inserted LPS metabolic labelling was initiated by addition of Kdo-$N_3$ when cultures reached $OD_{600}$ = 0.5.

**9. Lines 277-290: Phase separation has been used in terms of protein rich and protein less domains in the OM, not in terms of LPS diffusion. Is conclusion of lines 291-301 not the same as above?**

**Response**

We have revised the section **LPS-rich clusters remain spatially discrete and heterogeneously distributed, inconsistent with phase ordering in the outer membrane** (lines 321-407) to more clearly define 'phase separation or phase ordering' in its original compositional sense (i.e., demixing into LPS-rich versus OMP-rich domains), rather than as restricted LPS diffusion, and we added an explicit statement that we tested for key discriminating predictors of this framework i.e. domain coarsening (domain coalescence and Ostwald ripening), in both newly inserted and background LPS cluster populations.

**Key text that addresses point**

**Lines 331-352.** It has been proposed that organisation of the Gram-negative OM can involve phase separation or phase ordering, in which compositionally distinct regions (e.g., LPS-rich versus OMP-rich) emerge from thermodynamically favoured interactions under reduced membrane fluidity [33,41]. Here, we use 'phase separation' and 'phase ordering' in this compositional sense (rather than simply to denote restricted lateral diffusion). Because both LPS and OMPs exhibit very limited lateral mobility *in vivo*, any compositional ordering may be strongly diffusion-limited and therefore slow to relax on experimental timescales [10,11,32]. Nevertheless, an equilibrium-like ordering regime makes the clear, testable prediction that once compositionally distinct regions form, classical models of phase-ordering kinetics predict coarsening, in which smaller regions disappear while larger regions grow through coalescence and Ostwald ripening, yielding fewer, larger regions at later time points [42-48]. Ursell *et al.* provided an early framework for interpreting growth-dependent patterning in the OM based on microscopy of an OMP reporter and modelling of this membrane as an incompressible 2D fluid [13]. More recent live-cell AFM and high-resolution imaging studies have described the *E. coli* OM as a spatially heterogeneous mixture of OMP-rich and LPS-rich regions and discussed this organisation in terms of phase separation or phase ordering [10,13,33,49]. We therefore tested for time-dependent coarsening signatures in both newly inserted and background LPS cluster populations ***(Figs. 4B and 4C, Extended Data Fig. S10)***. In our analysis of newly inserted LPS ***(Fig. 4B, Extended Data Figs. S10D and S10F)***, the number of clusters per cell increased steadily over the 210-min experiment, while the total area covered by LPS also expanded, yet the mean cluster size plateaued after approximately 90 min ***(Extended Data Figs. S10D and S10E)***.

**10. Lines 304-310: The old LPS has not been shown to be lost, only the signal coming from it. Introducing the idea of losing the LPS at this point seems leading to the conclusions that the authors are trying to probe.**

**Response**

We agree that in this section we report on the measured loss of the Kdo-$N_3$ associated AF488 fluorescence and localisation density rather than total LPS mass. We have therefore revised the text, referring explicitly to depletion of the Kdo-$N_3$-labelled background LPS pool (signal), avoiding phrasing that implies direct measurement of 'old LPS loss' at this point in the results section.

**Key text that addresses point:**

**Lines 277-283.** Throughout, dSTORM localisation density ***(Fig. 3)*** and in-gel fluorescence ***(Figs. 6D and 6E)*** are used as relative proxies for the abundance of fluorescently labelled LPS; accordingly, we describe change as an increase (new LPS experiments) or decline (background/old) in metabolically- and fluorescently labelled LPS signal (newly inserted- or background LPS pool), rather than an absolute change in newly inserted / background LPS amount in cell OMs.

**Lines 299-320.** In a second set of experiments, we monitored the depletion of the Kdo-$N_3$-containing, background ('old') LPS pool from the OM of individual cells. LPS in cells were pre-labelled extensively with Kdo-$N_3$ (metabolic labelling of pre-existing of 'background' LPS). After removal of excess Kdo-$N_3$ cells were transferred to fresh medium containing native Kdo in place of Kdo-$N_3$. The decline of the fluorescent signal from background LPS was similarly quantified by single-cell SRM imaging ***(Fig. 3B, Extended Data Fig. S3A and Extended Data Table S16)***, and also by bulk biochemical analysis of extracted LPS (using in-gel fluorescence as a proxy for the Kdo-$N_3$ background/old pool; ***Fig. 6D, Extended Data Figs. S6A and S6C)***. At the single-cell level, dSTORM imaging revealed a progressive decline in fluorescently labelled LPS density across the entire OM ***(Fig. 3B, Extended Data Figs. S3A. S4A-C and S4H)***. At the bulk-population level fluorescence analysis of Tricine SDS-PAGE LPS gels showed decline of the (fluorescent) background/old Kdo-$N_3$ LPS signal over time ***(Fig. 6, Extended Data Figs. S6A and S6C)***.

In both single-cell dSTORM and bulk biochemical assays fluorescent background/old LPS signal reduction persisted into stationary phase, despite cell elongation and division slowing significantly ***(Fig. 3B, Extended Data Figs. S3A, S6A and S16C)***. These results show that the decrease in Kdo-$N_3$ background/old LPS fluorescent signal over time cannot be explained by the dilution alone that results from multiplying cells as previously postulated [10] ***(Extended Data Fig. S16C)***. Notably, both single cell dSTORM- and bulk biochemically derived rates of labelled background LPS fluorescent signal decline are faster than expected from dilution alone, consistent with growth-independent depletion or turnover of the background LPS pool ***(Extended Data Fig. S16C)***.

**Figure 3B Legend.** Quantification of newly inserted (new), metabolically labelled LPS accumulation following Kdo-$N_3$ addition, plotted as AF488-LPS localisation density per µm² of OM surface area for individual biological replicates (coloured symbols) and the replicate mean (black hexagons).

**Figure 3C Legend.** Quantification of background, metabolically labelled LPS loss following Kdo-$N_3$ removal (chased with native Kdo), plotted as AF488-LPS localisation density per µm² of OM surface area for individual biological replicates (coloured symbols) and the replicate mean (black hexagons).

**11. Fig 4 C: The number of clusters suddenly decreased between 60 and 90 min, probably coinciding with cell division?**

**Response**

We have re-analysed and re-plotted the background-cluster data. In the revised ***Figure 4C*** we now fit the mean background cluster number with an exponential decay-to-asymptote model, and in new ***Extended Data Figs. S10A–C*** we show the same data with mean ± standard error (s.e.m.). and 95% confidence intervals for the fits. These analyses demonstrate that the 60- and 90-min time points lie on a smooth exponential trajectory within the confidence intervals, with no evidence for an abrupt step change or inflection between these time points. In addition, because the cluster metrics are normalised per µm² of OM surface area, changes in cell size and geometry associated with elongation and division are expected to have reduced impact on the reported cluster number.

Moreover, because the population is not synchronised and our cluster metrics are normalised per µm² of OM area, division events are expected to be distributed across the time course, making it unlikely that an asynchronous division process would generate a discrete drop between 60 and 90 min rather than the gradual decay we observe.

Corresponding references to the exponential fit and ***Extended Data Figure S10*** have been added to the Results section describing ***Figure 4C*** (**LPS-rich clusters remain spatially discrete and heterogeneously distributed, inconsistent with phase ordering in the outer membrane** lines 321 - 407), and incorporated in legend to ***Figure 4***.

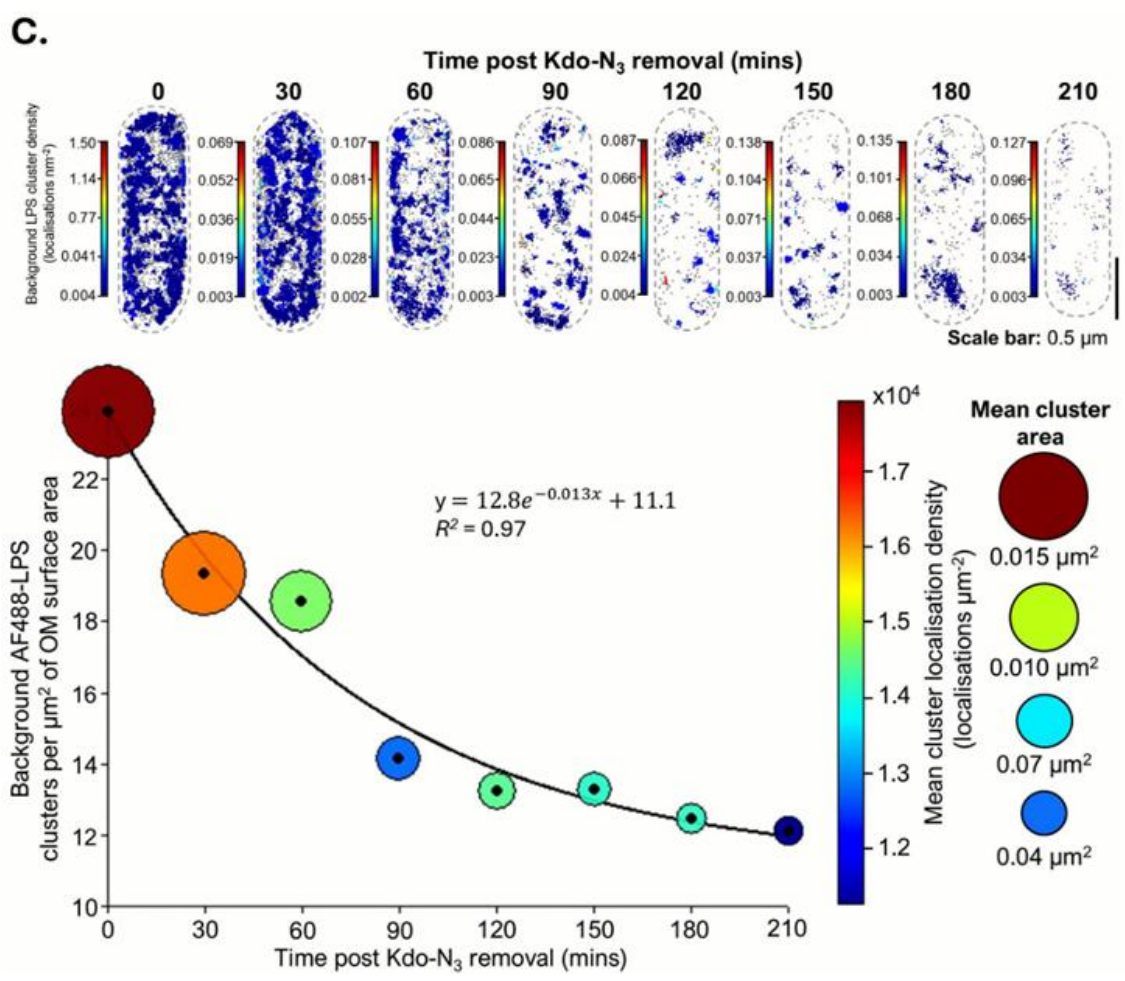


**Revised Figure 4C**

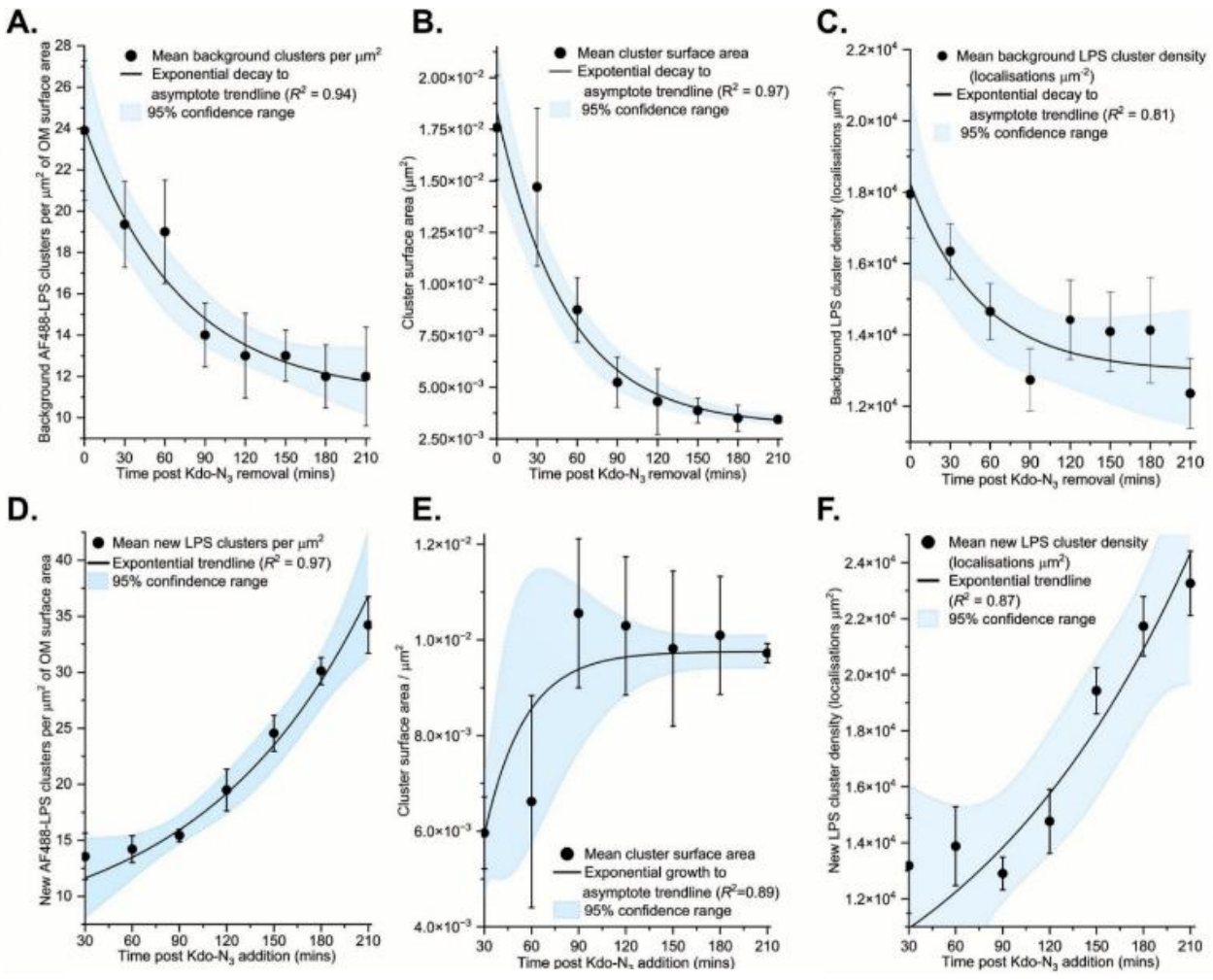


**New Extended Data Figure S10**

**Key text that addresses reviewer point**

**Lines 346-348.** We therefore tested for time-dependent coarsening signatures in both newly inserted and background LPS cluster populations ***(Figs. 4B and 4C, Extended Data Fig. S10)***.

**Lines 355-360.** Analysis of background LPS clusters reinforced this conclusion, with cluster number, mean cluster size, and density decreasing over the same period ***(Fig. 4C, Extended Data Figs. S10A-C)***. The 60- and 90-min time points lie on the fitted trajectory within the 95% confidence intervals, with no evidence of an abrupt step change in the observables ***(Fig. 4C, Extended Data Figs. S10A-C)***.

**Line 361-363.** We observed no evidence of coarsening (in which smaller domains vanish as larger domains grow), as expected for Ostwald ripening or domain fusion [45-47].

**Figure 4B Legend:**…The increase in cluster number is well described by an exponential growth-to-asymptote fit ($R^2$ = 0.98), consistent with continued LPS insertion after entry into stationary phase, without signatures of time-dependent domain coarsening

**Figure 4C Legend:**… The decrease in cluster number follows an exponential decay-to-asymptote ($R^2$ = 0.97), consistent with dilution of the pre-labelled LPS pool during exponential growth followed by continued loss during stationary phase with no evidence of time-dependent domain coarsening.

**Extended Data Figure S10 Legend.**…Mean values ± standard errors are plotted for each time point (n ≥ 30 cells per time point, N = 3 biological replicates), with exponential fits (black lines) and 95% confidence intervals (blue shaded regions).

**A.** … The data are well described by an exponential decay-to-asymptote fit ($R^2$ = 0.94).

Across all metrics, there is no evidence for abrupt step-like changes between time points; instead, the data follow smooth exponential trajectories consistent with gradual dilution/loss of background clusters and progressive accumulation of new clusters

**12. It is not clear if the data presented in Fig. 5 is enough to discard internalization of LPS. Controls should be shown**.

**Response**

We devised and carried out a control experiment to support the analysis of the data presented in ***Figure 5*** to verify that the apparent loss of background fluorescence could not be explained by intracellular internalisation, recycling or redistribution of labelled LPS. We carried out a biochemical fractionation control that tracks the subcellular localisation of fluorescently labelled background LPS across the entire post-labelling time window used in ***Figure 5***.

Specifically, we metabolically labelled LPS with Kdo–$N_3$ and fluorescently labelled the pre-existing ('background' or 'old') LPS pool *in vivo* using DBCO–AF488 (SPAAC; identical fluorescent labelling method used in live cell time course experiments presented in ***Figure 5 and Extended Data Fig. S5)***. After extensive washing to remove unreacted fluorophore, cultures were chased in analogue-free medium and sampled over time. Cells were then lysed via gentle (to ensure minimal inner and outer membrane fragmentation) sonication in the absence of any detergent that might conceivably affect LPS solubility. Lysates were then fractionated by ultracentrifugation into a soluble (cytoplasm/periplasm) fraction and a total (inner and outer) membrane pellet. The total membrane fraction was then subjected to *N*-lauryl sarcosinate (sarkosyl) extraction followed by ultracentrifugation to separate inner membrane (sarkosyl-soluble supernatant) and outer membrane (sarkosyl-insoluble pellet) fractions. Fractions were analysed by TSDS-PAGE with in-gel AF488 fluorescence detection.

The resulting data ***(Extended Data Fig. S14)*** show that AF488-labelled background Kdo-$N_3$-LPS is strongly enriched in the outer membrane fraction and decreases over time, in-line with our super-resolution imaging and TSDS-PAGE based turnover measurements ***(Figs. 3C, 4C, 6D, Extended Data Figs. S3A and S6A)***, while no time-dependent enrichment is detected in the soluble fraction. If labelled LPS were being internalised into the cytoplasm and/or periplasm, an increase in soluble AF488 signal would be expected. Instead, the signal in the soluble fraction remains at very low, background levels across the chase. A faint, time-invariant signal observed in the inner-membrane fraction is consistent with low-level cross-contamination inherent to detergent-based membrane separation rather than progressive internalisation that critically does not increase over time.

Additionally, we now explicitly present labelling specificity controls in a dedicated Extended Data figure ***(Extended Data Figure S15)***, demonstrating minimal non-specific staining in the absence of a functionalised Kdo-analogue, further addressing the 'controls should be shown' aspect of the comment. We have updated the manuscript to reference these new controls ***(Fig. 5 and Extended Data Figs. S14–S15)*** and added the corresponding description in the ***Methods*** section ***Extended Data S14 and S15*** legends.

**Key text that addresses reviewer point**

**Lines 418-420.** Alternatively, old LPS could conceivably be internalised into the cell and recycled internally or redistributed within the envelope (for example, via envelope-spanning processes rather than direct cytoplasmic recycling) [50].

**Lines 435-442.** To corroborate these imaging-based observations biochemically over the same period of growth (initiated at $OD_{600}$ = 1.5 and sampled for 120 min thereafter) ***(Fig. 5 and Extended Data Fig. S5)***, we fractionated identically labelled cultures into soluble, inner membrane and outer membrane compartments. We observed AF488-labelled background LPS predominantly in the outer membrane fraction, with no time-dependent enrichment in the soluble fraction ***(Extended Data Fig. S14)***. Together, these imaging and biochemical data indicate that the observed decline in background LPS signal is not explained by binary partitioning (as seen for OMPs) or by internal cytoplasmic recycling [32].

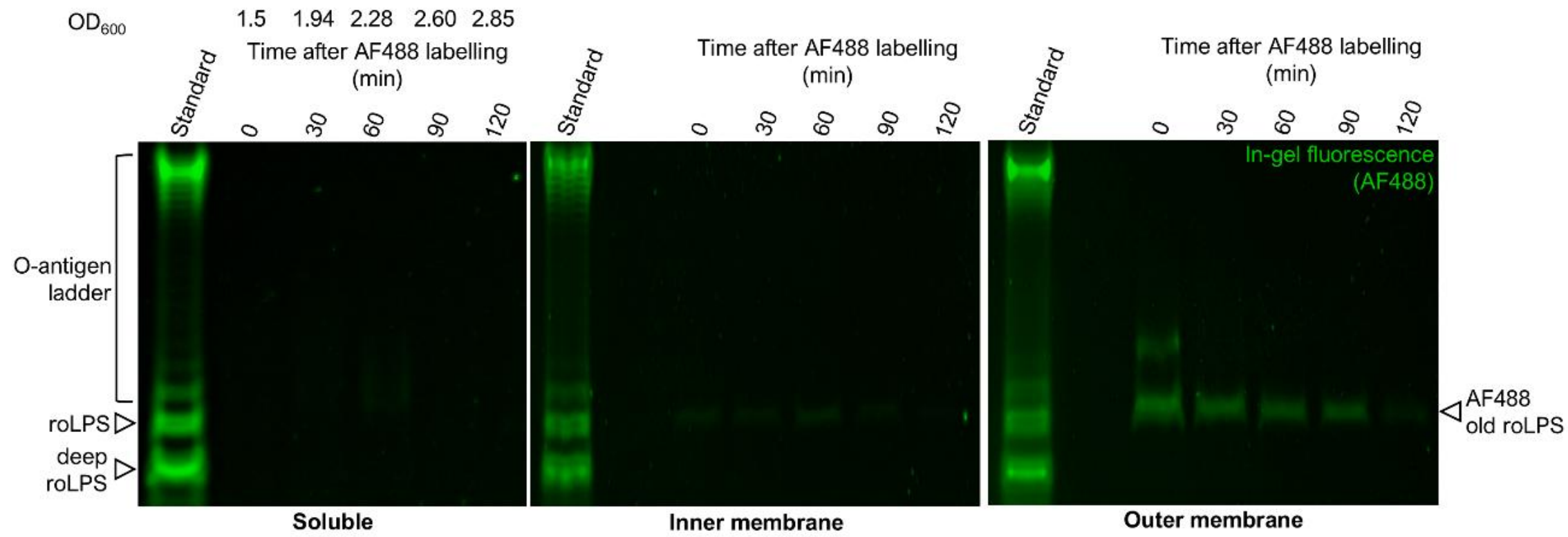


**Standard:** Commercially purchased O55:B5 AF488-LPS conjugate standard **roLPS:** Rough LPS **deep roLPS:** Deep rough LPS **AF488 old LPS:** Background AF488-labelled Kdo-$N_3$-LPS

**Extended data Figure S14**

**13. Lines 337-338. Why would the absence of polar concentration of LPS be evidence that binary partition is not occurring? The absence of old polar LPS could be caused by insertion of new LPS at the poles, as is suggested by this and other articles**.

**Response**

Our statement was intended to address the specific binary partition and polar sequestration model in which pre-existing outer membrane material becomes progressively enriched at the poles as cells elongate described by Rassam *et al.* (2015), which would predict an increase in relative polar enrichment of the pre-labelled (old/background) LPS pool over time; we do not observe such a trend. We agree that a lack of polar accumulation of old LPS could, in principle, also arise if new LPS is inserted at the poles and locally replaces and/or dilutes the labelled pool. We have revised the text to clarify this and to emphasise that our conclusion is based on the combined observations of **(i)** no net polar enrichment of the labelled background LPS and **(ii)** the spatially discrete insertion patterns of new LPS across the OM, rather than polar localisation alone.

**Reference**


32 Rassam, P. *et al.* Supramolecular assemblies underpin turnover of outer membrane proteins in bacteria. *Nature* **523**, 333-336 (2015).


**Key text that addresses reviewer point**

**i)** **Lines 372-375.** To test whether OM expansion during cell elongation driven redistribution produces a polar enriched pattern of older LPS, we categorised clusters from individual cell OMs into pole 1, mid-cell, and pole 2 regions based on their position along the longitudinal cell axis ***(Extended Data Fig. S4)***.

**ii)** **Lines 382-392.** Thus, we did not observe progressive enrichment of the pre-labelled background/old LPS pool at the poles over time, arguing against the specific binary-partition/polar-sequestration scenario in which new outer-membrane material is inserted preferentially away from the poles and pre-existing material is displaced poleward during elongation. However, we note that an absence of old-pool polar enrichment could also, in principle, arise if new LPS were inserted at the poles, locally diluting or replacing the labelled pool. Importantly, our two-colour pulse–chase data do not support preferential polar insertion of new LPS; instead, newly inserted LPS appears in discrete patches distributed along the longitudinal axis. This contrasts with growth driven redistribution frameworks derived from OMP reporters, in which spatially localised insertion together with restricted lateral mobility leads to growth driven redistribution and progressive accumulation of older OMPs at the poles [13,32].

14. **OMV are not made of only LPS, they normally contain the same proteins present in the OM, the authors seem to assume that the OMVs produced have no proteins and do not show if evidence of this.**

**Response**

We agree that OMVs are not composed solely of LPS and can contain outer membrane and periplasmic proteins. We have revised the manuscript text to remove any wording that could be read as implying that OMVs are 'protein-free'. The reviewer is correct that this should be demonstrated experimentally in our system. We therefore now include direct biochemical characterisation of OMVs produced by *E. coli* BW25113 under the same growth conditions used in the study, alongside matched cellular outer membrane (OM) fractions ***(Extended Data Figure S18)***. OM fractions were isolated by Sarkosyl extraction of total membranes to separate IM (Sarkosyl-soluble) and OM (Sarkosyl-insoluble), while OMVs were purified from cell-free supernatants by ultracentrifugation with a wash step to minimise soluble protein carryover. We then analysed global protein/OMP content by SDS-PAGE with Coomassie staining, and LPS content by TSDS-PAGE followed by silver staining. These new data show that our OMV preparation contains robust LPS signal, while global protein/OMP signal is markedly reduced (and below detection under the same loading scheme) in the OMV preparation compared to the OM fraction, when samples are loaded as matched culture equivalents ***(Extended Data Fig. S18)***. This directly addresses the reviewer's concern and supports our interpretation that under our culturing conditions OMVs are relatively enriched for LPS compared with bulk OM, without requiring any assumption that OMVs are entirely devoid of protein.

Finally, we note that the literature supports this interpretation. Previous studies have shown that OMVs are not necessarily a simple "mini-copy" of the OM, with multiple studies in *E. coli* reporting selective depletion of key OM–peptidoglycan tethering components (notably Braun's lipoprotein, Lpp) and that only a small fraction of abundant OMPs are released into vesicles, consistent with non-random cargo composition (McBroom *et al*., 2006, Schwechheimer *et al*., 2013, Kulp and Kuehn, 2010). We have incorporated these points in the revised text to clarify that our model focuses on relative LPS versus protein content in vesicles and does not assume OMVs lack proteins altogether.

**References**


**i)** Kulp, A. and Kuehn, M.J.. Biological functions and biogenesis of secreted bacterial outer membrane vesicles. *Annu. Rev. Microbiol.* **64**, 163–184 (2010).

**ii)** McBroom, A.J., Johnson, A.P., Vemulapalli, S. & Kuehn, M.J. Outer membrane vesicle production by Escherichia coli is independent of membrane instability. *J. Bacteriol.* **188**, 5385–5392 (2006).

**iii)** Schwechheimer, C., Sullivan, C. J. & Kuehn, M. J. Envelope control of outer membrane vesicle production in Gram-negative bacteria. *Biochemistry* **52**, 3031-3040 (2013).

**iv)** Wensink, J. and Witholt, B. Outer-membrane vesicles released by normally growing Escherichia coli contain very little lipoprotein. *Eur. J. Biochem.* **116**, 331–335 (1981).

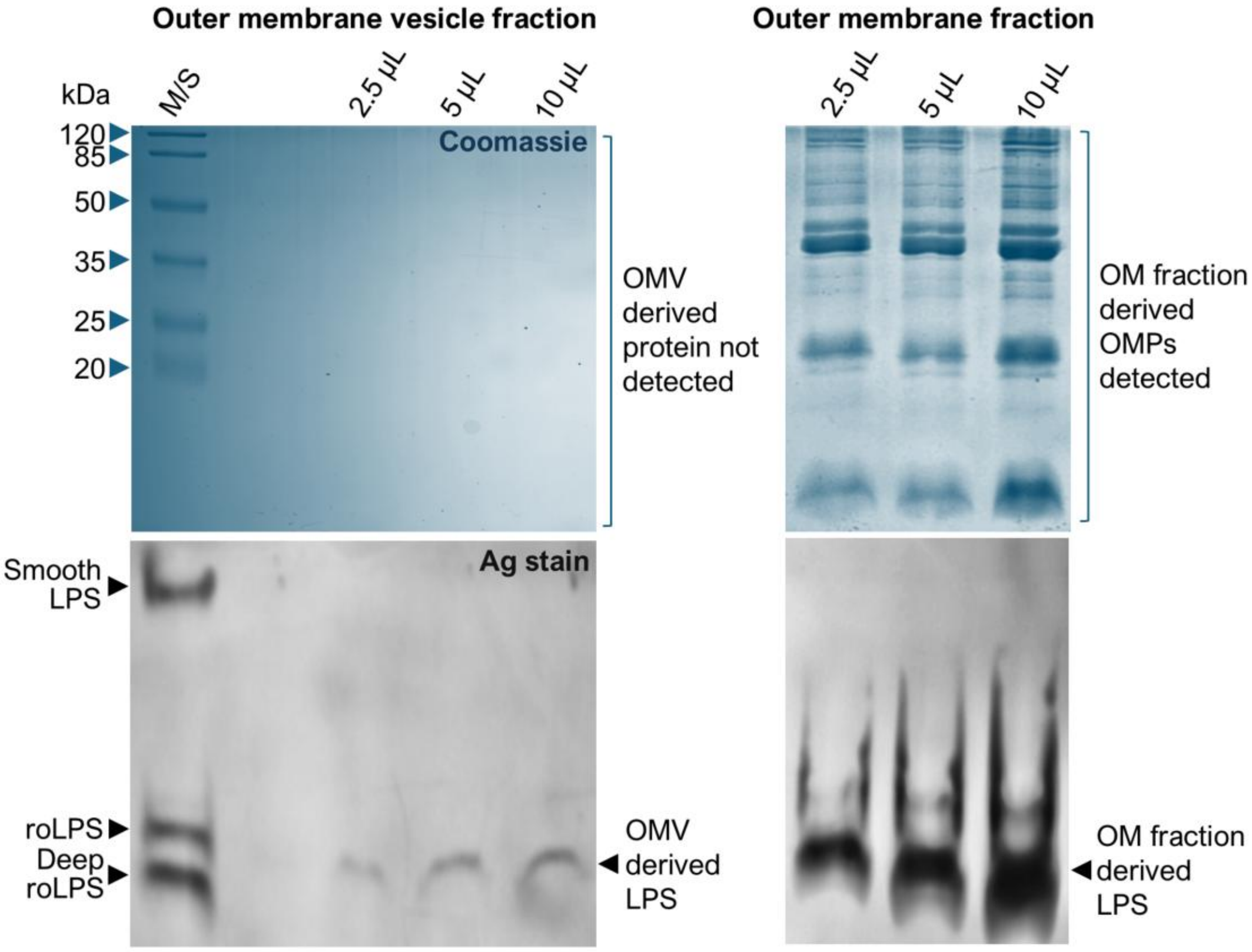


**Key**
**M.** Protein $M_w$ marker (top gel) **S.** *E. coli* O55:B5 LPS (1 µg, bottom gel)
**roLPS.** Rough LPS **Deep roLPS.** Deep rough LPS

**Extended Data Figure S18**

**Key text that addresses reviewer point**

**i)** **Lines 504 – 507.** Biochemical analysis of matched OM and OMV isolates confirmed OMV-associated LPS via TSDS-PAGE analysis followed by silver staining, whereas OMV-associated bulk protein signal was below the Coomassie detection limit at cell density-equivalent loadings ***(Extended Data Fig. S18)***.

**ii)** **Lines 581 - 584.** In-line with this framework, OMVs purified under our conditions were strongly depleted in Coomassie-detectable proteins while remaining LPS-rich relative to matched OM fractions ***(Extended Data Fig. S18)***.

**15. How much LPS is really lost in the OMVs? Is this consistent with the synthesis of "excess" LPS.**

**Response.** We agree that it is important to quantify the extent to which OMV production can account for the observed decline in the pre-labelled ('background/old') LPS pool, and to interpret this in the context of continued LPS insertion beyond that required for growth-associated OM expansion. We have therefore expanded the quantitative analysis at both the single-cell and population levels to directly address how much of the decline can be accounted for by OMV-associated export.

1. We used our LPS subpopulation selective labelling / dual colour dSTORM technologies to quantify OM bleb/buckle ROIs versus intact OM ROIs across a large dataset (n = 30, N = 3 independent biological replicates, both dye configurations). Critically, we show that OM bleb regions are enriched in the background-labelled pool and depleted in the newly inserted pool ***(Extended Data Fig. S13 and Extended Data Table S24)***. These data are consistent with vesiculation occurring preferentially from background pool enriched, LPS-dense regions, as expected under our OM buckling model ***(Fig. 7A)***, and support OMVs as a route for preferential export of the pre-existing labelled pool. This enrichment does not, by itself, imply an active 'old-versus-new' sorting mechanism.
2. At the population level, fractionation and in-gel fluorescence measurements show a reciprocal redistribution of the background-labelled signal from intact-cell OM fractions to an OMV fraction as cultures enter stationary phase ***(Figs. 6D–E; Extended Data Fig. S6C)***, coincident with increased OMV abundance by NTA ***(Fig. 6F; Extended Data Figs. S11C–D and S16A–B***; with size distributions in ***Extended Data Fig. S17)***.
3. To directly address 'how much' background LPS is exported by OMVs, we compare the TSDS-PAGE–derived decline of the background-labelled signal to a dilution-only expectation based on population growth and estimated OM expansion, then incorporate NTA-derived OMV abundance and size distributions to estimate OMV-associated export of the pre-labelled pool ***(Extended Data Figs. S16C–D)***. This analysis shows that the labelled background pool decreases faster than expected from dilution alone, and that OMV export can account for a substantial component of the excess decline beyond growth-driven dilution ***(Extended Data Figs. S16C–D)***. Accordingly, within conservative bounds, OMV-associated export accounts for a substantial fraction of the 'excess' decline beyond dilution-only expectations. We explicitly state that this modelling provides a conservative lower bound on OMV-mediated LPS loss, because supernatant processing and OMV isolation will unavoidably result in a reduction in recovered particle counts (e.g., filtration, high speed centrifugation and adsorption losses), which will decrease the inferred export term ***(Extended Data Figs. S16C–D).***
4. Finally, these export estimates are consistent with continued LPS insertion even as growth slows markedly: the newly inserted pool continues to accumulate at the OM during the exponential-to-stationary phase transition ***(Fig. 3)***, indicating that OM delivery/insertion is maintained beyond the requirements of elongation alone. Thus, OMV-associated export provides a quantitatively consistent sink that can accommodate continued ('excess') insertion under growth-limited conditions. Throughout, we therefore interpret localisation density and in-gel fluorescence as relative proxies for the abundance of the fluorescently labelled pool delivered to (or removed from) the OM, rather than as a direct readout of total LPS mass.

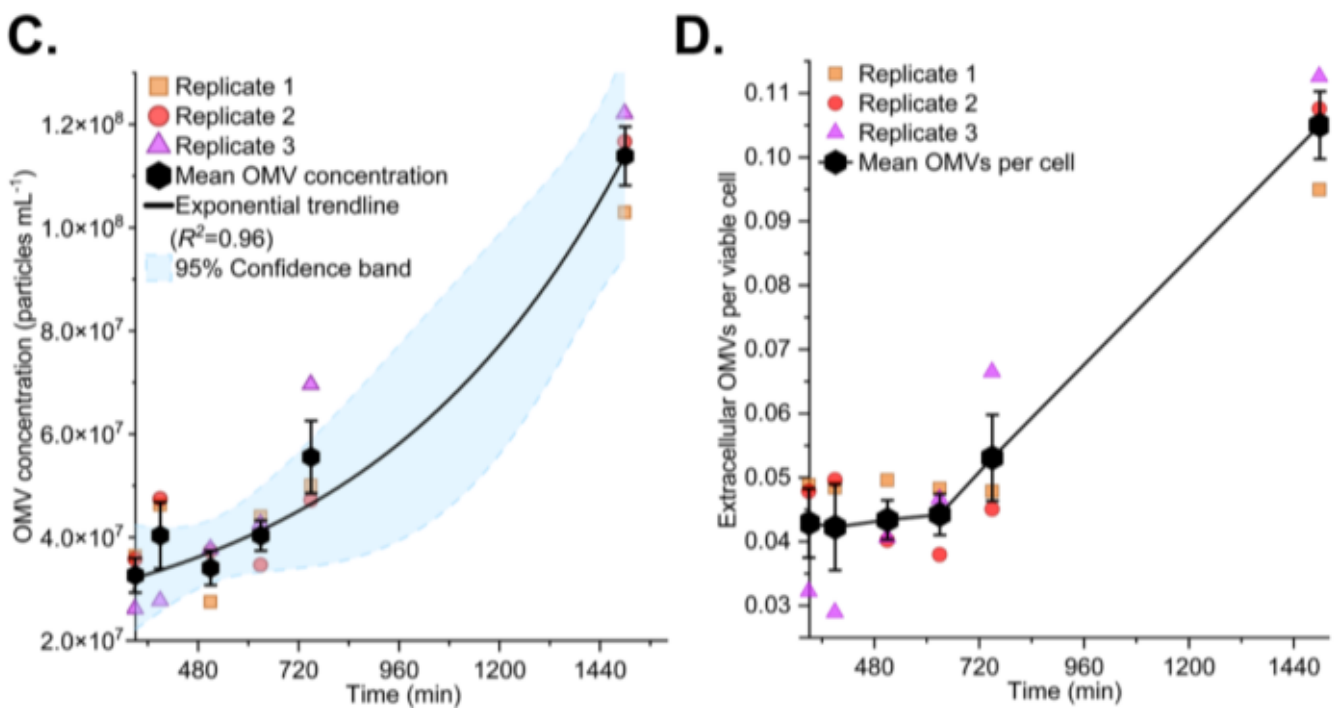


Extended Data Figures 11C and 11D

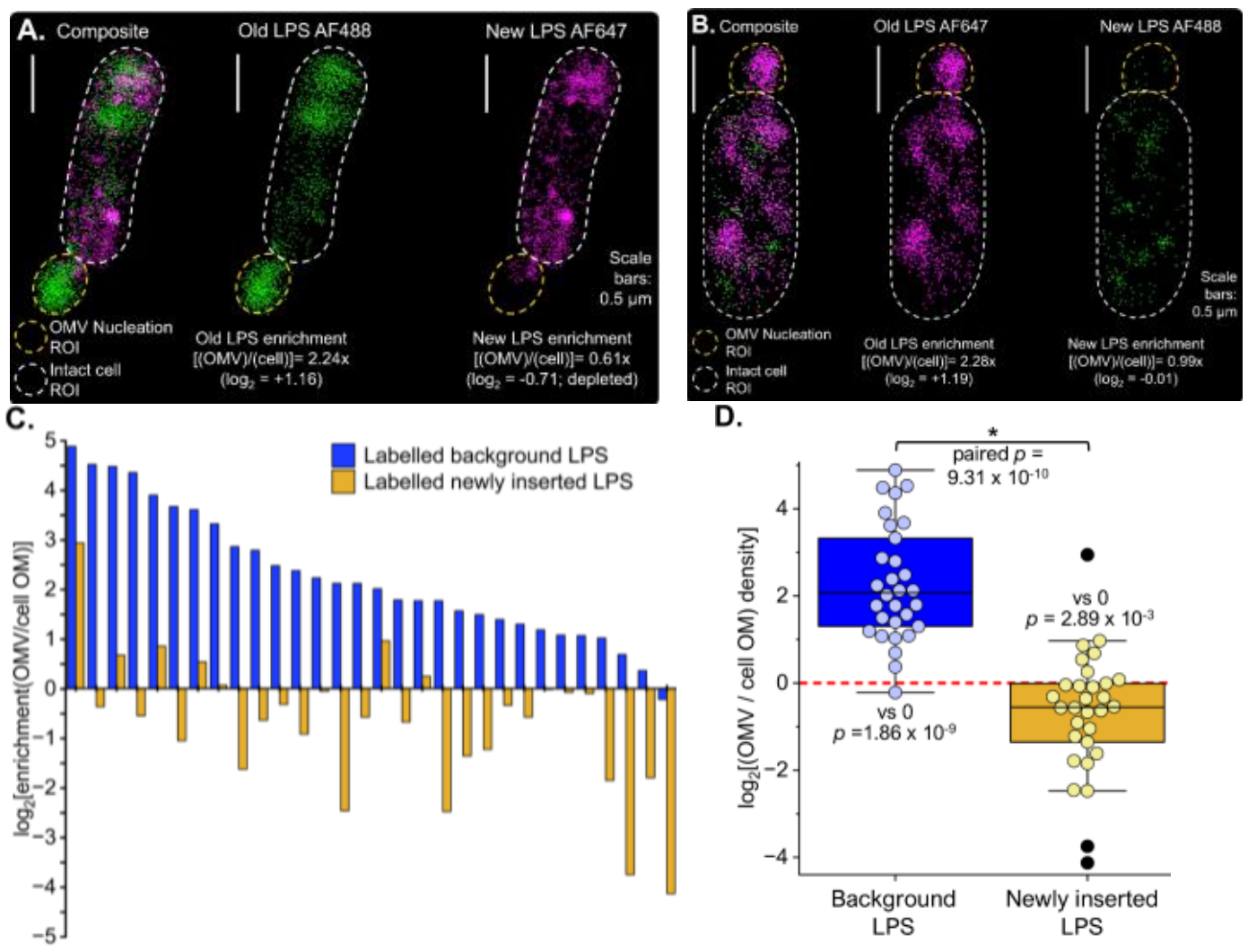


Extended Data Figure S13

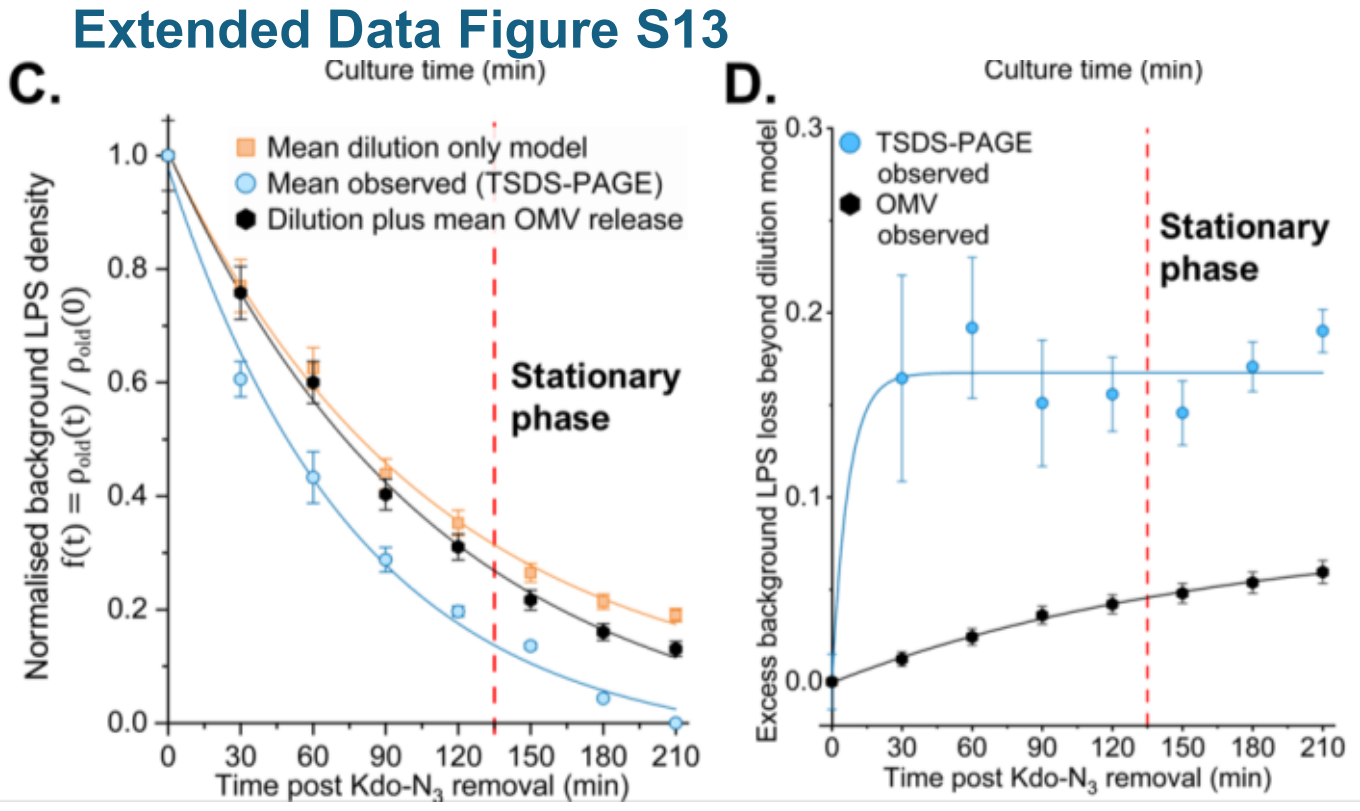


Extended Data Figure S16C and S16D

**Key text that addresses reviewer comment**

i) **Lines 489-496.** …***Extended Data Figure S13*** presents ROI-based localisation-density quantification of OMV/bleb protrusions versus intact-cell ROIs across a larger dataset (n = 30, N = 3 biologically independent experiments) and confirmed strong enrichment of old/background LPS in OMV ROIs (median $\log_2(S_{old\ LPS})$ = +2.07; 4.2-fold enrichment) and depletion of newly inserted LPS (median $\log_2(S_{new\ LPS})$ = –0.55; 0.68-fold depletion) relative to the surrounding OM ***(Extended Data Fig. S13 and Extended Data Table S24)***. Together, these data support a model in which stationary-phase cells preferentially shed OM regions that are enriched in older 'background' LPS via OMVs as part of growth-independent OM remodelling.

ii) **Lines 509-511.** In parallel, NTA measurements revealed a marked increase in OMV abundance as cultures transitioned into stationary phase ***(Fig. 6F and Extended Data Figs. S11C–D and S16A–B)***.

iii) **Lines 512-524.** To test whether the measured vesiculation rates can quantitatively account for the observed decline in background-labelled LPS fluorescence, we compared TSDS-PAGE–derived background LPS fluorescence decline with a dilution-only expectation calculated from population-level growth and OM expansion, and then utilised NTA-derived OMV abundance and size distributions to estimate OMV-associated export of the background LPS pool ***(Extended Data Fig. S16C–D)***. This analysis shows that the background LPS fluorescence signal decline measured by TSDS-PAGE is significantly faster than expected from dilution alone and that OMV export can account for a considerable component of the excess decline beyond cell multiplication driven dilution ***(Extended Data Fig. S16C–D)***. Because OMV recovery and particle counts can be reduced during supernatant processing and OMV isolation (e.g., resulting from filtration, high-speed prolonged centrifugation and adsorption losses), this estimate should be interpreted as a conservative lower bound on OMV-mediated export of the pre-labelled background LPS pool.

iv) **Lines 713-716.** By generating OMVs, the bacterium can shed portions of its OM that are enriched in background or 'old' LPS ***(Fig. 6C, Extended Data Fig. S13 and Extended Data Table S24)***, in line with prior observations in enteric bacteria [62,63,74,80]

v) **Lines 717-725.** Our data indicate that OMVs are enriched for the pre-labelled ('background/old') LPS pool, consistent with vesiculation contributing to background LPS turnover ***(Figs. 6A–D, Extended Data Figs. S12C-D;*** representative reconstructions; ***Extended Data Fig. S13;*** ROI-based quantification and ***Extended Data Table S24)***,but this enrichment does not imply exclusive packaging or an active 'old-versus-new' sorting mechanism.

**16. Reference 27 seems to be wrong**

**Response**

Reference 27 (now 39) has been corrected to the full citation of the original DBSCAN paper by Ester et al. (KDD-96), which is the algorithm used for our clustering analysis.

**Corrected reference:**

40 Ester, M., Kriegel, H.-P., Sander, J. & Xu, X. in *Proceedings of the Second International Conference on Knowledge Discovery and Data Mining (KDD-96).* (ed E. Simoudis, Han, J. & Fayyad, U. M.) 226-231 (AAAI Press).

**17. Reference 11 seems to be of a still not accepted manuscript; I am not sure this is acceptable and I would at least mention it the text.**

**Response**

This manuscript has now been published and reference 11 has been updated.

**Updated reference 11**

11 Nabarro, J. *et al.* Lipopolysaccharide confinement in the bacterial outer membrane is governed by interactions within the conserved Lipid A anchor. *EMBO J.* (2026). https://doi.org/10.1038/s44318-026-00711-5

**Reviewer #2 (Remarks to the Author):**

Nabarro et al. investigated the spatiotemporal dynamics of LPS in the bacterial outer membrane of *E. coli.* For this purpose, LPS and the LPS transporter LptD were fluorescently labelled using sophisticated click chemistry techniques. Labelled LPS was recorded in fixed and living cells and in pulse-chased experiments with different super-resolution microscopy techniques including dSTORM and SIM. The results achieved are of very high quality and spectacular in some respects. A number of conclusions were drawn that will help to change our view of LPS metabolism and -dynamics. E.g., *E. coli* rearranges its outer membrane (OM) through continuous removal of pre-existing LPS and insertion of new LPS at discrete sites. Also, *E. coli* LPS in the OM is cleared by release through OM vesicles suggesting that this is the major way how Gram-negative bacteria remodel their surface architecture independent of their growth phase.

**Major Points**

**1. All images showing LPS and LptD rely solely on click-chemistry fluorescence labelling of either incorporated functionalized Kdo or modified LptD. Even though these methods are highly efficient, incomplete or unspecific labelling could compromise the results. Can the authors provide an independent depiction of LPS and LptD not requiring click-labelling, e.g., using antibody staining? The antibody-stained and click-labelled LPS or LptD could then be checked for overlaps**.

**Response**

We agree with the reviewer that labelling specificity is critical. However, an antibody and lectin 'overlap verification experiment' is not a reliable orthogonal validation for the nanometre-scale spatial relationships quantified here by two-colour dSTORM. In particular, such staining can introduce:

**(i)** **Substantial linkage error**. Primary and secondary antibody stacks displace the fluorophore from the epitope by distances comparable to the spatial scales quantified in our experiments.

**Reference:** Früh, S. M. *et al.* Site-specifically-labeled antibodies for super-resolution microscopy reveal in situ linkage errors. *ACS nano* **15**, 12161-12170 (2021)

**(ii)** **Bivalency/multivalency-driven crosslinking and patching**. IgG antibodies and many lectins often reorganise membrane components during labelling**.**

**Reference:** Sograte-Idrissi, S. et al. Circumvention of common labelling artefacts using secondary nanobodies. Nanoscale **12**, 10226-10239 (2020).

**(iii)** **Heterogeneous surface accessibility effects at the Gram-negative outer leaflet that can bias apparent distributions.** Antigen exposure depends strongly on local LPS architecture and packing, so "non-overlap" can reflect shielding rather than true spatial separation.

**References:**

**(a)** Bentley, A. K., Philip. Effect of lipopolysaccharide structure on reactivity of antiporin monoclonal antibodies with the bacterial cell surface. *Journal of bacteriology* **170**, 1063-1068 (1988).

**(b)** Domínguez-Medina, C. C. et al. Outer membrane protein size and LPS O-antigen define protective antibody targeting to the Salmonella surface. *Nat. Comms.* **11**, 851 (2020).

For LptD, practical implementation is additionally constrained. Commercially available anti-LptD reagents are largely marketed/validated for denaturing applications (e.g., Western Blot/ELISA) rather than immunofluorescence microscopy of intact *E. coli* outer membranes. More fundamentally, LptD is embedded within the LPS-rich outer leaflet and functionally engages LPS during insertion, making it highly plausible that binding of a ~150 kDa IgG (and especially a secondary antibody stack) would sterically occlude nearby LPS and/or alter local organisation, confounding any attempt to interpret 'overlap' between anti-LptD staining and a large lectin-/ antibody-based LPS-binding probe.

**Instead, we addressed this concern using multiple independent, non-antibody specificity and robustness controls already included in the manuscript:**

**i)** **Functional/identity validation of labelled LptD*.** We show that ncAA-containing LptD* retains native LptD*/LptE complex formation (unheated vs heated SDS-PAGE), supporting correct folding/assembly of the labelled transporter ***(Extended Data Figure S1C)***. In addition, in our prior work, we demonstrated that ncAA-containing LptD* can restore outer membrane barrier function in an LptD-defective background *(imp4213)*, providing an orthogonal functional validation that ncAA incorporation does not abolish LptD function.

**Reference:** Nabarro, J. et al. Lipopolysaccharide confinement in the bacterial outer membrane is governed by interactions within the conserved Lipid A anchor. *The EMBO Journal* (2026). https://doi.org/10.1038/s44318-026-00711-5

ii) **Robustness of LptD–new LPS co-localisation to fluorophore choice:** Swapped dye configurations yield near-identical cross-correlation behaviour and consistent co-enrichment, arguing against dye-/channel-specific artefacts ***(Extended Data Figures 1A and 1D)*****.**

iii) **Specificity/neutrality of Kdo-analogue labelling of LPS**: Kdo-$N_3$ and Kdo-alkyne give equivalent incorporation/fluorescence and do not measurably perturb LPS behaviour (verified via FRAP-based mobility comparisons), and fluorescent TSDS-PAGE confirms signal in the Kdo-analogue containing, AF488-labelled LPS band ***(Extended Data Figures S2A-C and Extended Data Figures S15A-D).***

2. **Further, can the authors present data of wild type E. coli not treated with functionalized Kdo but only incubated with the fluorescent azide and alkyne compounds, demonstrating that the latter do not cause background/unspecific staining?**

**Response**

To directly address the possibility of non-specific staining by the click-reactive fluorophores, we have added dedicated labelling specificity controls in ***Extended Data Figure S15***. In these experiments, wild-type *E. coli* BW25113 cells were cultured under identical conditions but supplied with native Kdo (no bio-orthogonal handle, i.e. cells were not treated with Kdo-$N_3$ or Kdo-alkyne) and were then processed using the same fluorophore concentrations / CuAAC reaction conditions used for Kdo–$N_3$ / Kdo-alkyne metabolic labelling (including the corresponding dye-azide and dye-alkyne reagents, with identical washing conditions and imaging settings). Under these conditions, we observe minimal cell-associated fluorescence, demonstrating that the fluorescent azide/alkyne compounds do not cause significant 'background' or unspecific staining in the absence of the correspondingly functionalised Kdo analogue incorporation ***(Extended Data Fig. S15)***. These controls were performed across three biologically independent experiments, with >100 cells imaged per condition. We now cite these controls at the first introduction of the CuAAC labelling strategy in the main text (Results section: ***Spatial segregation of newly inserted and background LPS persists over time)***.

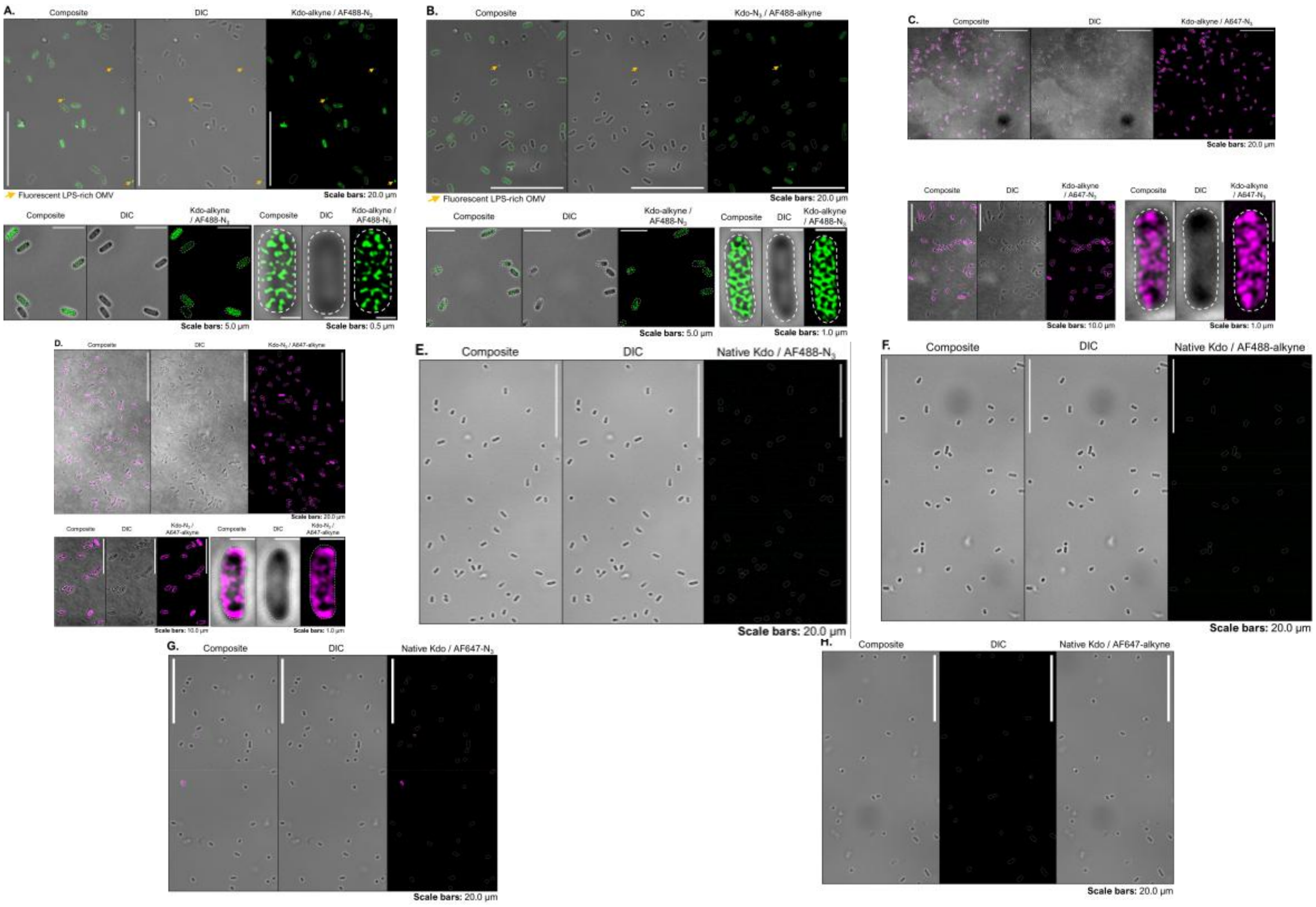


**Extended Data Figure S15**

**Key text that addresses reviewer comment. Lines 190– 194.** Control reactions in wild-type *E. coli* BW25113 cells cultured with native Kdo (no bio-orthogonal handle) and processed with the corresponding fluorescent azide(-$N_3$)/alkyne reagents under identical CuAAC conditions showed minimal non-specific cell-surface staining when imaged via 3D SIM[2] ***(Extended Data Fig. S15***, N = 3 biologically independent replicates, n > 100 cells imaged per condition).

**3. The spatiotemporal relation of LptD and LPS suggest a functional relation, also when previous published work is considered. However, the data not provide proof that LptD under these conditions really affect LPS insertion or dynamics. Is it possible to (partly) inhibit LptD to see whether this affects LPS localization and/or dynamics?**

**Response**

We agree that our two-colour imaging establishes a strong spatiotemporal association between LptD* and the appearance of newly inserted LPS, but this correlation alone does not constitute definitive proof that perturbing LptD directly tests whether LptD determines the observed insertion pattern or downstream LPS organisation. We have therefore ensured that the manuscript frames LptD* as a marker for discrete OM sites of new LPS delivery and interprets LptD*–labelled new LPS co-localisation as consistent with LptDE-mediated insertion rather than as perturbation-based causal evidence.

We chose not to perform partial inhibition or depletion of LptD as an additional experiment because this is not a clean or readily interpretable perturbation *in vivo*. LptD is essential, and established LptD depletion and defect strategies are known to cause rapid, pleiotropic outer-membrane defects (including barrier failure, compromised permeability, activation of envelope stress responses, and altered OM composition / LPS vs phospholipid asymmetry). Under such conditions, any changes in LPS localisation or dynamics would be difficult to attribute specifically to altered insertion-site behaviour rather than the indirect consequences of global envelope dysregulation (Wu *et al*., 2005, Ruiz *et al*., 2005, Ruiz *et al*., 2006, Sperandeo *et al*., 2008).

Importantly, substantial independent evidence already supports the functional link between LptDE and LPS delivery to the outer leaflet. Genetic and biochemical studies establish studies establish LptDE as the terminal OM translocon for LPS insertion, and LptD impairment disrupts OM biogenesis and permeability phenotypes consistent with impaired LPS transport, OM assembly and asymmetry maintenance (Wu *et al.*, 2005, Ruiz *et al.*, 2005, Ruiz *et al.*, 2006, Sperandeo *et al.*, 2008). Consistent with this, we verify in this manuscript that the ncAA-labelled LptD* construct used for imaging behaves as expected: LptD* forms the anticipated LptD*/LptE complex without evidence of gross assembly disruption (consistent with correct assembly) ***(Extended Data Fig. S1C)***. In addition, our prior dSTORM, fluorescence recovery after photobleaching (FRAP) and single particle tracking (SPT) study shows that the LptD-defective *imp4213* background exhibits markedly increased LPS lateral mobility and reduced antibiotic tolerance compared to wild type *E. coli* BW25113 (Nabarro *et al*., 2026). Finally we demonstrated that wildtype LPS lateral mobility and antibiotic resistance levels could be restored via the production of ncAA-containing LptD* in *imp4213*; further evidence that our LptD* construct was functional (Nabarro *et al*., 2026).

Together, these data fit the accepted consensus in the literature that LptD is mechanistically coupled to LPS delivery, while the present manuscript focuses on directly measuring **(i)** the nanoscale spatiotemporal relationship between LptD and newly inserted LPS, and **(ii)** whether newly inserted versus pre-existing LPS exhibits phase-ordering/coarsening or diffusion-limited, insertion-patterned organisation. Our key conclusions do not rely on invoking any additional LptD-dependent 'sorting' mechanism beyond the established essential role of LptDE in LPS transport and insertion.

We have:

**(i)** Clarified that the LptD–new LPS relationship is correlative where LptD*–new LPS co-patterning is introduced.

**(ii)** Added explicit text noting that the co-patterning supports LptDE-linked delivery sites but does not on its own prove causality.

**(iii)** Included citations to LptD depletion/defect literature and to our *imp4213*/rescue mobility measurements for functional context.

**References (for response above *not* manuscript)**


**i)** Wu, T., Malinverni, J., Ruiz, N., Kim, S., Silhavy, T. J. & Kahne, D. Identification of a multicomponent complex required for lipopolysaccharide transport to the outer membrane of *Escherichia coli*. *Cell* **121**, 235–245 (2005).

**ii)** Ruiz, N., Falcone, B., Kahne, D. & Silhavy, T. J. Chemical conditionality: a genetic strategy to probe organelle assembly. *ACS Chem. Biol.* **1**, 385–395 (2006)

**iii)** Sperandeo, P. *et al.* Functional analysis of the protein machinery required for transport of lipopolysaccharide to the outer membrane of *Escherichia coli*. *J. Bacteriol.* **190**, 4460–4469 (2008).

**iv)** Nabarro, J. *et al.* Lipopolysaccharide confinement in the bacterial outer membrane is governed by interactions within the conserved Lipid A anchor. *EMBO J.* (2026). doi:10.1038/s44318-026-00711-5


**Key text that addresses reviewer comment.**

**Lines 95-98.**… we implemented a novel dual labelling strategy in combination with two-colour SRM analysis enabling detection of newly inserted LPS and the OM LPS translocon LptD (LptDE), used here as a marker of putative LPS delivery/insertion sites [24] ***(Fig. 1A and Extended Data Fig. S1)***.

**Line 101-104.** We verified that the ncAA-labelled LptD* forms the expected LptD*/LptE complex and shows no evidence of gross assembly disruption ***(Extended Data Fig. S1C)***, supporting its use as a reporter for LptD-associated sites.

**Lines 129-131.** This co-patterning is in line with LptDE-mediated delivery, but does not by itself establish that LptD activity is the sole determinant of the observed insertion pattern.

**Lines 134-136.** Given that LptDE is the established OM translocon for LPS insertion, we interpret LptD* puncta as markers of discrete delivery sites.

**Minor points**

**1.** The elegant labelling system using click chemistry might be hard to understand for readers not familiar with this system. It could e.g., help to consistently use one nomenclature throughout the whole manuscript (e.g. avoid using sometimes N3 and sometimes azide). This includes "Keys" of Figures 1A and 2A.

**Response**

We have now standardised the nomenclature throughout the manuscript, Extended data, method and figure keys. On first use we define the full names of the probes [8-azido-3,8-dideoxy-D-manno-oct-2-ulosonic acid (Kdo-$N_3$) and 8-(4-pentynoylamido)-3-deoxy-D-manno-oct-2-ulosonic acid (Kdo-alkyne)] and subsequently refer to them consistently as Kdo-$N_3$ and Kdo-alkyne. In instances where it has been necessary to retain the word 'azide' for example 'Copper(I) mediated azide-alkyne cycloaddition (CuAAC) and strain promoted azide-alkyne cycloaddition (SPAAC) (-$N_3$) has been inserted after the word 'azide' to assist readers less familiar with this labelling system.

**2. LptD* is expressed from a vector within E. coli bacteria that still express endogenous LptD. Thus, there is likely an additional endogenous pool of LptD in the bacteria. To which extent might this influence the results, considering that only the vector-expressed LptD pool is shown? Also, could the presumably increased amount of LptD in the bacteria lead to changes in LPS insertion dynamics?**

**Response.** We agree that endogenous chromosomal LptD remains present and that LptD* therefore represents only the plasmid-expressed, ncAA-containing subpopulation. For this reason, lptD* was expressed from pBADcLIC under non-induced ('leaky') conditions to avoid LptD overproduction, which could otherwise perturb LPS transport. Importantly, our conclusions on growth-phase–decoupled LPS insertion and turnover **(Figs. 3–7)** were obtained from Kdo-$N_3$ pulse–chase assays performed in the parental BW25113 background (without LptD* production), so they are not dependent on LptD* abundance.

**Key text that addresses reviewer comment.**

**Lines 104-109.** As LptD is essential [28-31], cells necessarily retain the endogenous chromosomal LptD pool; thus, direct stochastic reconstruction microscopy (dSTORM) localisations report the plasmid-expressed ncAA-containing LptD* subpopulation. To minimise perturbation of total LptD abundance and LPS transport dynamics, *lptD** was expressed from a pBADcLIC vector under non-induced ('leaky') conditions (without L-arabinose), since induction would drive total LptD levels far above native.

**References** (showing that LptD is essential for *E. coli* viability)**:**


28 Braun, M. & Silhavy, T. J. Imp/OstA is required for cell envelope biogenesis in Escherichia coli. *Mol. Microbiol.* **45**, 1289-1302 (2002).

29 Chng, S.-S., Ruiz, N., Chimalakonda, G., Silhavy, T. J. & Kahne, D. Characterization of the two-protein complex in Escherichia coli responsible for lipopolysaccharide assembly at the outer membrane. *Proc. Natl Acad. Sci. USA* **107**, 5363-5368 (2010).

30 Wu, E. L. *et al.* E. coli outer membrane and interactions with OmpLA. *Biophys. J.* **106**, 2493-2502 (2014).

31 Wu, T. *et al.* Identification of a multicomponent complex required for outer membrane biogenesis in Escherichia coli. *Cell* **121**, 235-245 (2005).

**Inserted in methods for clarity**

i) **Bacterial strains and plasmids…**no L-arabinose was added in any experiment, lptD* and ompA* expression relied on basal araBAD promoter activity ('leaky' expression).

ii) **LptD fluorescent labelling via ncAA incorporation followed by CuAAC.** In the absence of L-arabinose, basal araBAD promoter activity drove low-level expression of the recombinant amber-stop codon-containing lptD gene (lptD*) ; addition of propargyl-L-Lysine enabled amber suppression by the pylRS and tRNAPyl pair and incorporation of the ncAA into full-length LptD with the ncAA integrated at one of four positions in its extracellular surface loops (LptD*). Because LptD is essential, cells retain an endogenous chromosomal LptD pool; therefore, dSTORM localisations reported only the plasmid-expressed ncAA-containing LptD* subpopulation.

**3. The authors show that preferentially background LPS is enriched in OMVs for LPS removal. Is there any data or are there any ideas on how the bacteria distinguish between new and background LPS? If yes it could be added to the discussion.**

**Response:** We agree that our data show OMVs are enriched for the pre-labelled ('background/old') LPS pool, but we do not interpret this as evidence of an active "old-versus-new" sorting/discrimination mechanism. We have therefore revised the Discussion to clarify this point and to propose a alternative in which enrichment arises passively from the persistent spatial segregation of newly inserted and background LPS ***(Figs. 1–4)*** coupled to envelope mechanics/bleb formation in our buckling model ***(Fig. 7; Extended Data Fig. S9)***, consistent with prior reports linking vesiculation to envelope remodelling and LPS remodelling-associated OMV production**.**

**References**


60 Schwechheimer, C. & Kuehn, M. J. Outer-membrane vesicles from Gram-negative bacteria: biogenesis and functions. *Nature Rev. Microbiol.* **13**, 605-619 (2015).

61 Bonnington, K. E. & Kuehn, M. J. Outer membrane vesicle production facilitates LPS remodeling and outer membrane maintenance in Salmonella during environmental transitions. *mBio* **7**, 10.1128/mbio. 01532-01516 (2016).

62 Elhenawy, W. *et al.* LPS remodeling triggers formation of outer membrane vesicles in Salmonella. *MBio* **7**, 10.1128/mbio. 00940-00916 (2016).


**Key text that addresses reviewer comment.**

**Line 717-743.** Our data indicate that OMVs are enriched for the pre-labelled ('background/old') LPS pool, consistent with vesiculation contributing to background LPS turnover ***(Figs. 6A–D, Extended Data Figs. S12C-D;*** representative reconstructions; ***Extended Data Fig. S13;*** ROI-based quantification and ***Extended Data Table S24)***,but this enrichment does not imply exclusive packaging or an active 'old-versus-new' sorting mechanism. At present, we have no evidence for the existence of cellular mechanisms capable of distinguishing between LPS cohorts based on their relative 'age'; instead an explanation consistent with our data is that this background LPS enrichment in OMVs emerges from OM spatial organisation and envelope mechanics. Newly inserted LPS is delivered at discrete LptD-associated insertion sites ***(Figs. 1B–E)*** and remains spatially confined and segregated from the pre-existing LPS pool ***(Figs. 2, 3A and 4B)***, which would bias the background LPS population to become relatively concentrated over time within LPS-rich regions that are spatially separated from highly tethered OMP-rich zones over time. In the context of our OM buckling model ***(Fig. 7A),*** such LPS-rich regions being comparatively less constrained would make them prone to membrane deformation and budding, providing a physical basis for preferential enrichment of the background LPS pool in released vesicles without requiring a means to actively discriminate between different LPS subpopulations ***(Fig. 7A)*** [5,6,32].

**4. Figure S1A: Please indicate that you switched the dyes for both targets in the figure, not only by just switching the colours in the figure (for example by indicating the dyes that were used below the target).**

**Response**

We have updated **Extended Data Fig. S1A** (see below) with annotations of the dye–target assignments for each reciprocal labelling configuration (LptD*–AF488 / new LPS–AF647 and LptD*–AF647 / new LPS–AF488), rather than indicating the swap only by colour. The figure and legend now make clear that both targets were dye-swapped, and that the observed co-localised puncta are independent of dye choice.

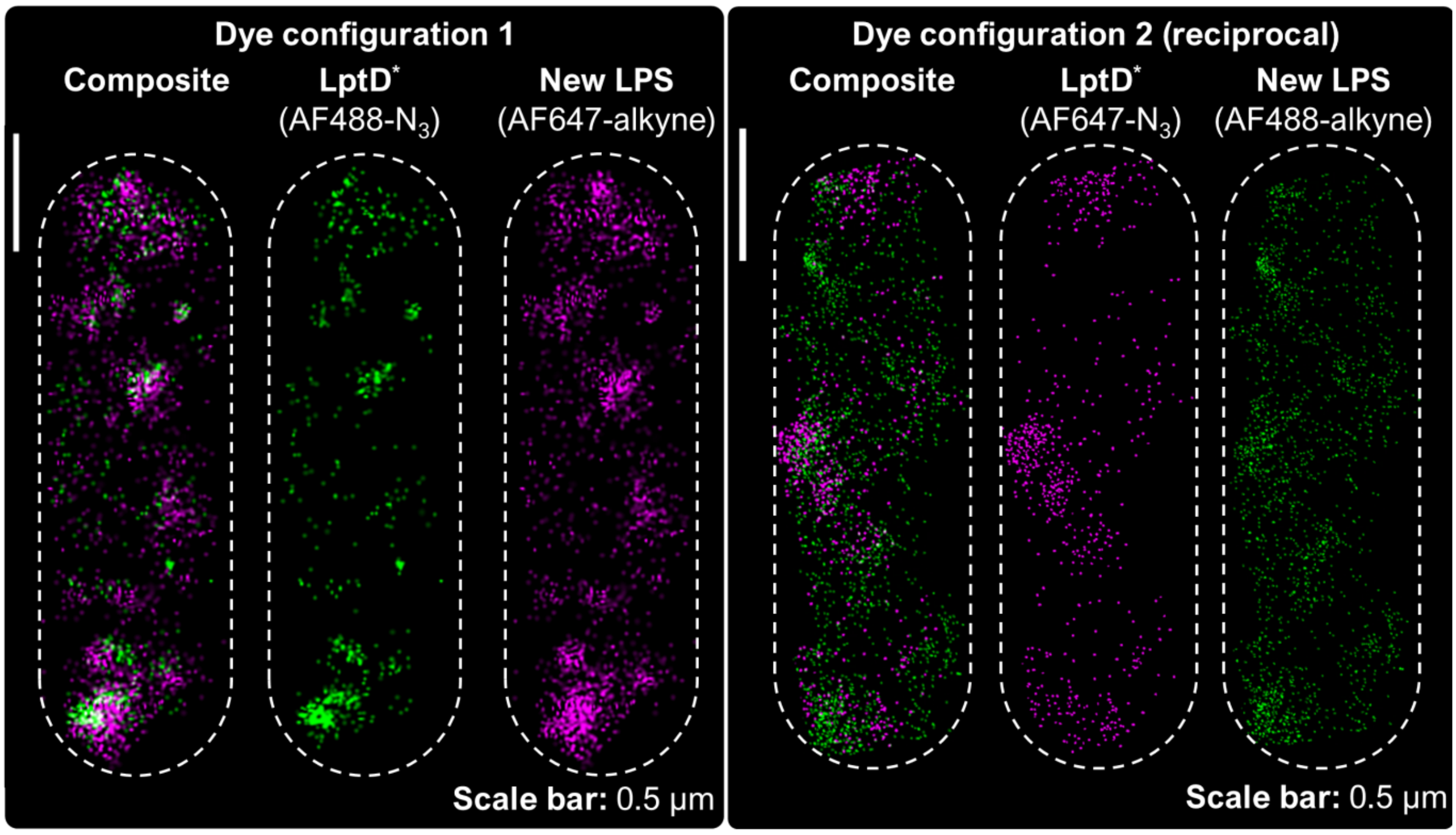


**Extended data Figure S1A, clearer dye-target assignments:**

**Legend: Reciprocal dye labelling confirms robust LptD–newly inserted LPS puncta independent of fluorophore identity.** Additional two-colour dSTORM images of E. coli outer membranes labelled for recombinant LptD* and newly inserted LPS 30 minutes after co-addition of N-propargyl-L-lysine and Kdo-$N_3$. LptD* was mutated with an amber stop codon replacing D600 in extracellular loop 9 and expressed via genetic code expansion, enabling site-specific conjugation to an azide($N_3$)-functionalised fluorophore via CuAAC. Newly inserted LPS was metabolically labelled with Kdo-$N_3$, enabling site-specific conjugation to an alkyne-functionalised fluorophore via CuAAC. In one dye configuration, LptD was labelled with AF488-$N_3$ and newly inserted LPS with AF647-alkyne (left); in the reciprocal configuration, LptD was labelled with AF647-$N_3$ and newly inserted LPS with AF488-alkyne (right) (dye–target assignments are indicated above each panel). Discrete, co-localised puncta are observed across the OM, independent of dye combination**. Scale bars:** 0.5 µm.

**5. Line 118/119: Please explain what the abbreviation BAM stands for.**

**Response**

We have now defined BAM on first use in the main text as the β-barrel assembly machinery (BAM), and retained the abbreviation thereafter.

**Key text that addresses reviewer comment.**

**Line 131-136.** The distribution of LptD*–LPS colocalisation sites across the OM indicates that LPS insertion occurs at discrete locations over the entire OM ***(Figs. 1D and 1E)***, rather than being concentrated within specific regions of the OM such as the division septum as is the case for β-barrel assembly machinery (BAM)-mediated OMP insertion [32].

**6. Line 161/162: Figure numbering appears not correct, is Fig S2A meant?**

**Response**

We corrected the figure callout in the Results section. The schematic referenced here is **Fig. 2A** (not an Extended Data panel), and we have ensured Extended Data figures are referenced consistently using the S# convention **(e.g., Extended Data Fig. S2A/S2C)** throughout the manuscript.

**Key text that addresses reviewer comment.**

**Line 182-184.** To probe spatiotemporal LPS dynamics, cells were pulse-labelled with a Kdo-alkyne probe to generate what we designated as the pre-existing or 'background' LPS population ***(Fig. 2A)*** [38]..

**7. Figure 2A; Key: Symbol for Kdo-azide is missing.**

**Response**

The key for Figure 2A has been corrected to include the symbol for Kdo-$N_3$, whilst also standardising Kdo-alkyne and Kdo-$N_3$ nomenclature in line with a previous reviewer's comment.

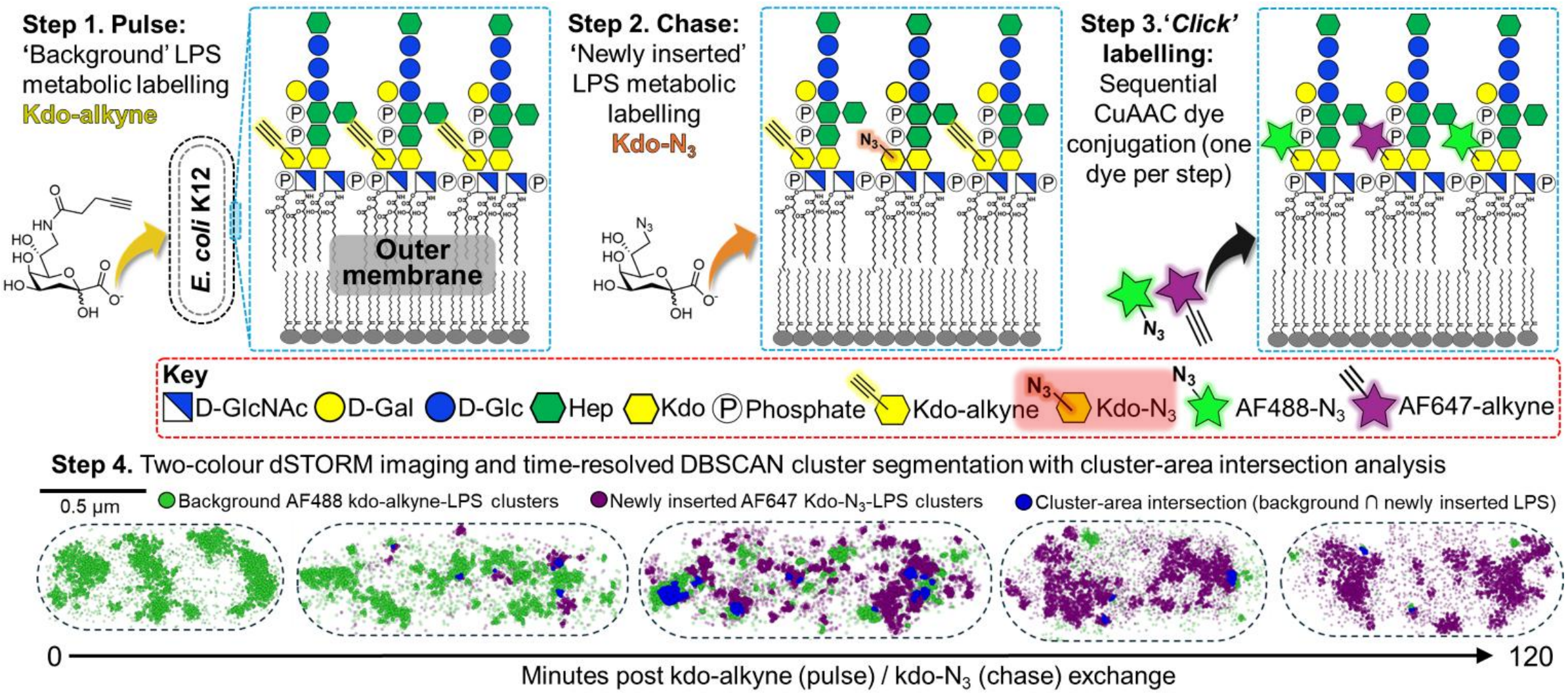


**Revised Figure 2A:** With the symbol for Kdo-$N_3$ (highlighted in red, absent in previous draft) inserted.

**8. Line 234: Why would photobleaching be an issue here or matter at all and not in the other experiments? The same number of frames should be also acquired in all experiments for maintaining comparability throughout the manuscript. While Figure 3A shows a clear picture, the limited number of 5000 frames could theoretically result in a general underrepresentation of LPS.**

**Response**

We agree that dSTORM localisation counts are not an absolute readout of total LPS, and we therefore use curvature-corrected localisation density as a relative proxy under strictly standardised conditions. For **Figure 3**, we deliberately fixed acquisition length (5000 frames) and standardised imaging/processing settings across all time points so that each cell OM is sampled identically. Because the Fig. 3 analysis makes quantitative comparisons of localisation density across time points, keeping the frame number fixed is essential to maintain comparability within this dataset. This is important because photophysical depletion/photobleaching is cumulative over time and can be non-linear; extending acquisitions can cause localisation yield to saturate as emitters bleach or enter long-lived dark states, which compresses dynamic range and can bias comparisons between low- and high-signal time points (Dempsey *et al.***,** 2011; Annibale *et al.***,** 2011)**.** We also note that, while a 5000-frame acquisition may under-sample the absolute number of localisations, any such undercount is systematic because it applies equally across all Fig. 3 time points acquired under identical conditions; our conclusions rely on relative changes measured under matched acquisition and reconstruction settings.

Longer acquisitions were used for the datasets underpinning ***Figures 1 and 2*** because those experiments focus on spatial patterning/colocalisation and benefit from maximising localisation yield, rather than comparing localisation densities quantitatively across conditions. Accordingly, we do not use datasets acquired with different frame numbers for direct quantitative localisation-density comparisons across experiments; density-based time-course conclusions are restricted to datasets acquired under identical acquisition lengths and processing parameters (e.g. ***Figure 3)***. We have revised the text in the Results section to signpost this to the reader for clarity.

**References**


**i)** Dempsey, G. T., Vaughan, J. C., Chen, K. H., Bates, M. & Zhuang, X. Evaluation of fluorophores for optimal performance in localization-based super-resolution imaging. *Nat. Methods* **8**, 1027–1036 (2011).**Localisation yield is not a simple linear function of imaging duration in dSTORM acquisitions; the fraction of fluorophores 'surviving' photobleaching decreases with time.**

**ii)** Annibale, P., Vanni, S., Scarselli, M., Rothlisberger, U. & Radenovic, A. Quantitative photo activated localization microscopy: unravelling the effects of photoblinking. *PLoS One* **6**, e22678 (2011). **Extension acquisitions can bias quantitative comparisons.**

**Key text that addresses reviewer comment.**

**Lines 267-283.** For these time-course measurements, we used identical imaging and processing settings across fixed acquisition window (5000 frames) at every time point, so that localisation density per OM area could be compared consistently as a relative proxy Kdo-$N_3$-containing, new LPS delivered to the OM. We note that a fixed 5000-frame acquisition may underrepresent absolute localisation yield, but this does not affect the comparability of localisation densities across the time course because every time point was acquired and processed under identical conditions. This fixed-window approach avoids time-dependent photophysical depletion and photobleaching effects that can compress dynamic range and bias comparisons across time points when acquisition length is varied or extended. Longer acquisitions were used elsewhere ***(Figs. 1 and 2)*** to maximise localisation yield for spatial patterning and colocalisation analyses, rather than for quantitative cross-condition density comparisons.

**9. Figure 5: Mid cell:** Could you choose a different colour for the images here as blue might not be visible well for the human eye.

**Response**

The LUT has been altered for mid-cell images to ensure they are clearly visible for the human eye. The LUT of the lower OM optical section has also been switched since magenta and red are analogous colours and therefore distort the composite images.

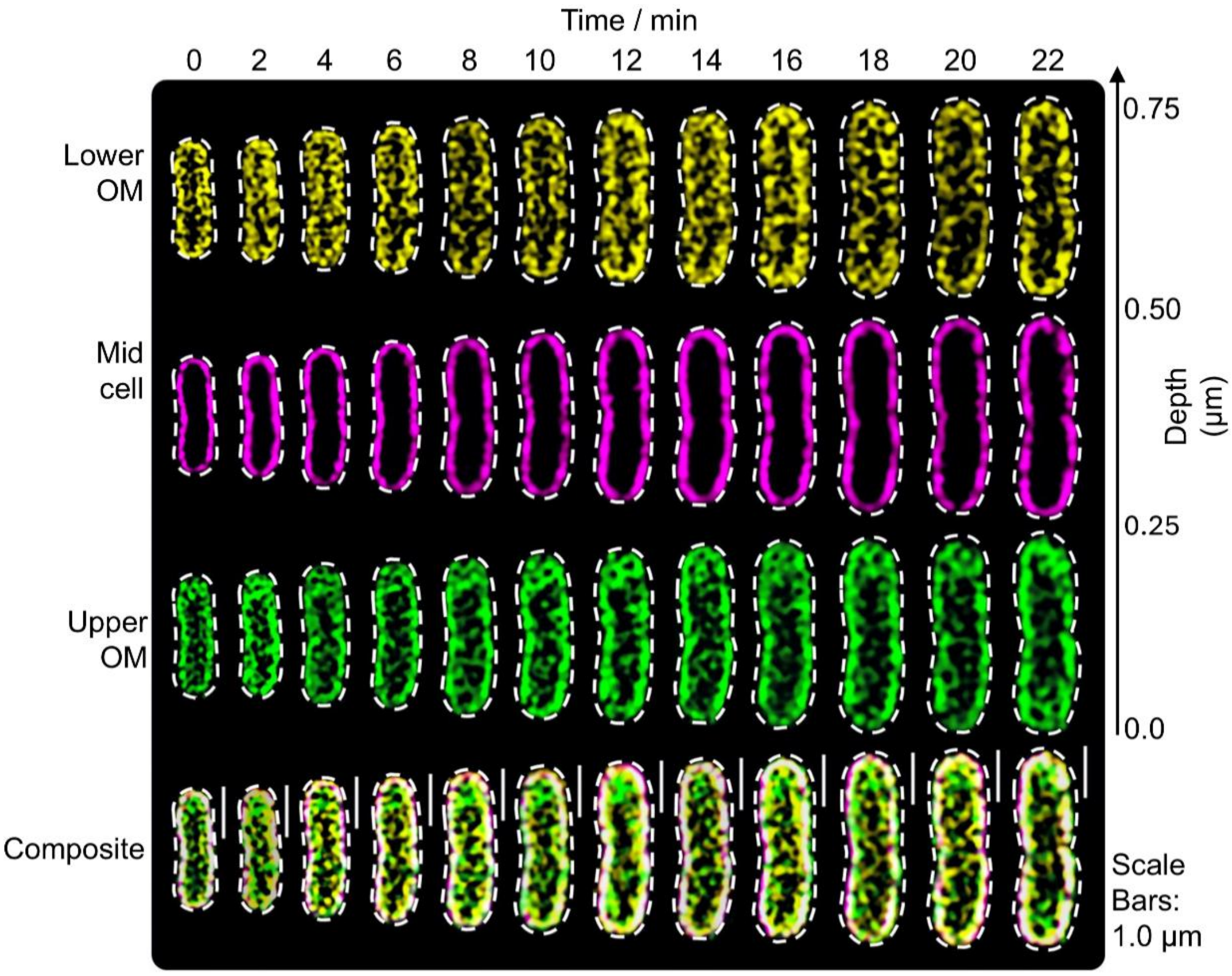


**New figure 5 with LUT changed to ensure they are clearly visible** *for the human eye*

**10. Figure S5A-C: A timestamp should be inserted in the live-SIM movies.**

**Response**

Timestamps have been added to live-SIM movies and the LUT has been altered for mid-cell video to ensure it is clearly visible for the human eye and matches those used in Figure 5 ensuring the reader can easily match optical section images in Figure 5 to Figure 5A-C videos (application of response to reviewer comment #9). In addition to being supplied in the extended data section, they are provided in a separate, additional ZIP folder: Extended_data_live_cell_SIM_background_LPS_videos

**Live-SIM movies containing timestamps**

I. Lower OM ***(Extended Data Figure S5A)***

Extended_Data_Figure_S5_A_video.mp4

II. Mid-cell ***(Extended Data Figure S5B)***

Extended_Data_Figure_S5_B_video.mp4

III. Upper OM ***(Extended Data Figure S5C)***

Extended_Data_Figure_S5_C_Video.mp4

**11. Line 336/Figure 5: While the mentioned live-cell observations are consistent with the dSTORM data, can also the decrease of background LPS over time be observed in live-SIM in longer recordings?**

**Response**

It is more difficult to quantify the decrease in background LPS over longer periods of time using the live-cell 3D-lattice SIM approach. Firstly, the cells must be maintained in an oxygenated medium which supports bacterial growth. These conditions are sub-optimal for fluorescence imaging, particularly when the higher laser excitation duty cycle required for dynamic lattice SIM imaging is used. This leads to faster photobleaching in the sample which in turn prevents longer time recordings from being possible. Secondly, the spatial resolution of 3D-lattice SIM is inferior (~60-80 nm in x,y plane) to that of dSTORM (~25 nm in x,y plane) which means resolving the finer detail of the LPS-rich regions would be impossible. Finally, the two-step reconstruction approach used in lattice SIM[2] can introduce image artifacts when the overall fluorescent signal is lower in the sample. For these reasons, we utilised 3D-lattice SIM exclusively for probing whether internalisation of old LPS occurred during cell elongation and to follow vesiculation at the cell surface. In parallel, we exploited the strengths of dSTORM imaging done on fixed cells to localise old and new LPS molecules with higher spatial resolution, and to follow LPS turnover over longer periods of time. This SRM imaging strategy exploits the different yet complementary strengths of lattice SIM and dSTORM to address different mechanistic questions related to LPS turnover in the bacterial OM. It is reassuring that the live-cell lattice SIM observations are consistent with the dSTORM data given the differences in sample preparation and image reconstruction employed for the two techniques.

**Reviewer #3 (Remarks to the Author):**

This manuscript by Nabarro et al. uses super-resolution microscopy to identify that turnover of LPS in the outer membrane of E. coli is facilitated by the insertion of new LPS at discrete patches across the entire outer membrane and specifically release of old LPS in outer membrane vesicles. This turnover is shown to proceed well into stationary phase and is thus independent of cell growth, which challenges the null hypothesis that older LPS is passively diluted by the insertion of new LPS in a growth dependent manner. The strength of the manuscript is the quality of the data. Outer membrane dynamics are an important aspect of bacterial physiology and the data reported here describe the relationship of these dynamics to cell growth than previous studies. The main weakness of the manuscript is that something very similar to the central model they propose was previously reported, and this study is not cited or discussed. As far as I know, the vesiculation during stationary phase is a novel result.

**Major Comments: 1.** The authors should read, cite, and discuss the model presented by Ursell et al (doi:10.1371/journal.pcbi.1002680), which was based on a mathematical analysis of experimental data, similar to the present study. I believe that the "insertion trapping" model is essentially the same as the one presented there even if the resolution of the data in the current study is higher. Therefore, it would be helpful if the authors could present the previous model in the introduction, build on it (if possible) in the main text, and compare the two models in the discussion. There may be quantitative differences or advances that the current study could offer in light of the higher resolution. There may also be slightly different conclusions that arise due to differences in the mathematical models.

**Response**

We have now cited this study and incorporated it explicitly into the manuscript in multiple places. First, we introduce the Ursell *et al*. work in the ***Introduction***, summarising their LamB reporter–based time-lapse analysis and mathematical modelling that inferred stochastic, spatially localised insertion along the cylindrical cell surface and, under restricted lateral mobility, growth-driven displacement leading to age-dependent enrichment of older outer-membrane material at the poles (Ursell *et al.* 2012). Second, we build on this framework in the ***Results*** by using our LPS readout to test a key qualitative prediction of growth-driven displacement models (progressive polar enrichment of the older labelled pool). We do not detect significant progressive enrichment of the labelled background ('old') LPS at the poles over the measured time window, and we now explicitly contrast this with the OMP reporter-based growth-driven redistribution model ***(Fig.4 and Extended Data Fig. S4)***. Third, we compare the Ursell *et al*. growth-dependent patterning model with our insertion-trapping model in the ***Results*** and ***Discussion***. We note the conceptual alignment with spatially localised insertion under restricted lateral mobility, but emphasise that our study provides major quantitative advances enabled by two-colour super-resolution imaging measurements with pulse–chase labelling of LPS in the outer leaflet. In particular, our simulations are constrained by experimentally quantified parameters (LPS insertion and loss kinetics and old–new segregation metrics) and incorporate growth-independent LPS removal via OMV shedding, extending models of growth-dependent redistribution of OM material ***(Fig. 7 and Extended Data Fig. S9)***.These additions address the reviewer's request to present, build on, and compare the Ursell *et al*. model while maintaining the focus of the manuscript on direct nanoscale measurement and mechanistic modelling of LPS turnover and OMV-mediated remodelling.

**Reference:**

Ursell, T. S., Trepagnier, E. H., Huang, K. C. & Theriot, J. A. Analysis of surface protein expression reveals the growth pattern of the Gram-negative outer membrane. *PLoS Comput. Biol.* **8**, e1002680 (2012).

**Key text that addresses reviewer comment.**

**Introduction > Lines 51-57.** In line with this growth-and-displacement mechanism, using a covalently labelled LamB trimeric maltoporin derivative as a surface reporter, Ursell *et al.* combined time lapse fluorescence microscopy with mathematical modelling to infer that *E. coli* OM expansion occurs through stochastic, spatially localised insertion of new material along the cylindrical cell surface [13]. Under restricted lateral mobility, growth driven displacement then predicts progressive enrichment of older outer membrane material at the poles [13].

**Results > Lines 389-392.** This contrasts with growth driven redistribution frameworks derived from OMP reporters, in which spatially localised insertion together with restricted lateral mobility leads to growth driven redistribution and progressive accumulation of older OMPs at the poles [13,32]..

**Results > Lines 632-636.** The organising rule is conceptually consistent with the stochastic, spatially localised insertion and growth-dependent patterning previously observed for LamB dynamics by Ursell *et al.* [13]. Here, it is constrained and tested using direct nanoscale measurements of LPS turnover and old/new LPS segregation, and it incorporates growth independent loss via OMV shedding.

**Discussion > Lines 763-771** Ursell *et al.* combined time-lapse fluorescence microscopy of an OMP reporter with mathematical modelling to infer that stochastic, spatially localised OMP insertion and growth-driven displacement can generate polar enrichment of older material when lateral mobility is restricted [13]. Our insertion trapping framework is conceptually consistent with localised insertion and constrained redistribution [11,13,32], but here it is constrained by experimentally quantified parameters and evaluated using two-colour single molecule super resolution measurements of LPS turnover and old and new LPS segregation in the outer leaflet, incorporating growth independent LPS loss through OMV shedding.

**2. Similarly, the authors propose liquid-liquid “phase separation” as a hypothetical organizing principle of the outer membrane to contrast their model, but despite the observation of limited separation of OMPs and LPS (Benn *et al.* PNAS 2021), it is well understood that the outer membrane is a growing solid material (neither OMPs or LPS diffuse), and therefore this seems to be a strawman argument, especially in light of the Ursell study.**

**Response**

We agree that on cellular timescales the *E. coli* OM is mechanically robust and strongly kinetically constrained, with very limited lateral mobility of both LPS and OMPs. Phase separation as an organising principle for LPS and OMPs was considered here because prior published work adopted it for the outer membrane – we have not constructed a strawman argument. We have revised the manuscript to clarify that our use of ‘phase separation’ or ‘phase ordering’ is not intended to imply a freely mixing, rapidly equilibrating liquid bilayer (or to claim liquid–liquid phase separation *in vivo*). Instead, we use the term in the compositional sense that has been adopted in recent experimental studies describing the *E. coli* OM as a mosaic of OMP-rich and LPS-rich regions (including live-cell AFM and high-resolution imaging work, e.g. Benn *et al.*, 2021, Webby *et al.*, 2022 and Kumar *et al.*, 2025), while also emphasising restricted mobility.

Importantly, our analysis does not rely on assuming liquid-like diffusion. Rather, we test a specific, falsifiable prediction of an equilibrium-like compositional ordering regime: time-dependent coarsening (domain growth via coalescence or Ostwald ripening) and increasing overlap between cohorts. In our two-colour pulse–chase labelling dSTORM data and cluster analyses, we do not observe coarsening signatures for either newly inserted or background LPS over the measured time window.

To ensure a like-for-like comparison, we have also clarified the modelling framework; the ‘phase ordering’ comparison is implemented under the same restricted lateral diffusion assumptions and identical experimentally derived turnover constraints as the insertion-trapping model, differing only in the organising rule (an effective short-range like–like interaction term, rather than insertion-site trapping). Under these matched constraints, the equilibrium-like ordering rule predicts increasing old–new overlap and domain growth that are not observed experimentally, whereas the insertion-trapping model reproduces key experimental hallmarks.

Finally, as requested in comment 1, we now cite and discuss the work of Ursell *et al*. and position our work in that context. Our insertion-trapping model is conceptually consistent with the spatially localised insertion and the constrained growth-dependent redistribution of an OMP reporter described by Ursell *et al*. This prior work demonstrated that growth-dependent patterning could manifest in the OM, resulting in older OMPs being partitioned to the opposite polar regions of daughter cells. Importantly, OMP insertion sites were shown subsequently by Rassam et al. (2015) to be restricted to the mid-cell region of an elongating bacterium which contributes to the observed old-new partitioning effect. In contrast to the model of Ursell *et al*. our insertion-trapping model can provide a plausible mechanistic explanation for how LPS turnover occurs in both growth-dependent and growth-independent (via OMV shedding) manners even when LPS insertion sites are located across the cell surface. We have also directly constrained and tested our new insertion-trapping model using two-

colour super-resolution imaging measurements of LPS turnover and old–new segregation in the outer leaflet during cell growth.

**References**

10 Kumar, S. *et al.* Immobile lipopolysaccharides and outer membrane proteins differentially segregate in growing Escherichia coli. *Proc. Natl Acad. Sci. USA* **122**, e2414725122 (2025).

33 Benn, G. *et al.* Phase separation in the outer membrane of Escherichia coli. *Proc. Natl Acad. Sci. USA* **118**, e2112237118 (2021).

49 Webby, M. N. *et al.* Lipids mediate supramolecular outer membrane protein assembly in bacteria. *Sci. Adv.* **8**, eadc9566 (2022). https://doi.org/10.1126/sciadv.adc9566

**Key text that addresses reviewer comment.**

**Lines 334-341** Here, we use 'phase separation' and 'phase ordering' in this compositional sense (rather than simply to denote restricted lateral diffusion). Because both LPS and OMPs exhibit very limited lateral mobility *in vivo*, any compositional ordering may be strongly diffusion-limited and therefore slow to relax on experimental timescales [10,11,32]. Nevertheless, an equilibrium-like ordering regime makes the clear, testable prediction that once compositionally distinct regions form, classical models of phase-ordering kinetics predict coarsening, in which smaller regions disappear while larger regions grow through coalescence and Ostwald ripening, yielding fewer, larger regions at later time points [42-48].

**References** (10, 33 and 33 conclude Lipid and OMP organisation in the OM is governed by phase separation whilst simultaneously acknowledging (or characterising) LPS and OMP restricted lateral mobility):

10 Kumar, S. *et al.* Immobile lipopolysaccharides and outer membrane proteins differentially segregate in growing Escherichia coli. *Proc. Natl Acad. Sci. USA* **122**, e2414725122 (2025).

33 Benn, G. *et al.* Phase separation in the outer membrane of Escherichia coli. *Proc. Natl Acad. Sci. USA* **118**, e2112237118 (2021).

42 Elson, E. L., Fried, E., Dolbow, J. E. & Genin, G. M. Phase separation in biological membranes: integration of theory and experiment. *Annu. Rev. Biophys.* **39**, 207-226 (2010).

43 Bray, A. J. Theory of phase-ordering kinetics. *Adv. Phys.* **43**, 357-459 (1994).

44 Cugliandolo, L. F. Coarsening phenomena. *C. R. Phys.* **16**, 257-266 (2015).

45 Siggia, E. D. Late stages of spinodal decomposition in binary mixtures. *Phys. Rev. A* **20**, 595 (1979).

46 Lifshitz, I. M. & Slyozov, V. V. The kinetics of precipitation from supersaturated solid solutions. *J. Phys. Chem. Solids* **19**, 35-50 (1961).

47 Wagner, C. Theorie der alterung von niederschlägen durch umlösen (Ostwald-reifung). *Ber. Bunsenges. Phys. Chem.* **65**, 581-591 (1961).

48 Cahn, J. W. & Hilliard, J. E. Free energy of a nonuniform system. I. Interfacial free energy. *J. Chem. Phys.* **28**, 258-267 (1958).49 Webby, M. N. *et al.* Lipids mediate supramolecular outer membrane protein assembly in bacteria. *Science Advances* **8**, eadc9566 (2022)

**3. Figure 6: The vesiculation data is nice and potentially novel, but there is no quantitative assessment of it. How many vesicles per cell per unit time occur during stationary phase?**

**Response**

We agree that a quantitative assessment strengthens the vesiculation analysis. We have therefore added a dedicated quantification of OMV accumulation during stationary phase using nanoparticle tracking analysis (NTA), together with time-matched normalisation to viable cell concentration (CFU equivalents) to report extracellular OMVs on a per-cell basis.

Specifically, we measured OMV particle concentration (particles $mL^{-1}$ at multiple stationary phase time points and determined viable cell concentration at matched time points by CFU plate counts ***(Extended Data Table S23)***. We then calculated OMVs per viable cell, as OMVs(t) / CFU(t), enabling comparison across stationary phase despite changes in culture density. These analyses are presented in new ***Extended Data Fig. S11C-D*** below (OMV concentration per mL, viable cell concentration, and OMVs per viable cell over time, including biological replicate variability). From the OMVs-per-viable-cell time course, we additionally report an apparent net extracellular accumulation rate ($\Delta$(OMVs/CFU)/$\Delta$t; a conservative lower bound): during stationary phase OMVs/CFU increases from ~0.044 at 390 min to ~0.053 at 750 min (~$1.5\times10^{-1}$ OMVs $cell^{-1}$ $h^{-1}$), and from ~0.053 at 750 min to ~0.105 at 1500 min (~$4.2\times10^{-3}$ OMVs $cell^{-1}$ $h^{-1}$) ***(Extended Data Fig. S11D).***

We note that because NTA quantifies extracellular particles in clarified, processed supernatants, this metric reflects net extracellular OMV accumulation per viable cell (production minus losses during handling and purification, e.g. resulting from 0.45 µm followed by 0.22 µm filtration) rather than an instantaneous single-cell production rate. The isolation and measurement workflow was stringently standardised across all time points, allowing robust relative quantification of vesiculation dynamics during stationary phase. We now reference this quantification in the main text alongside ***Fig. 6F*** and use it to support the conclusion that OMV release continues, and increases, during stationary phase.

**Key text that addresses reviewer comment.**

**Lines 532-537.** OMV particle concentrations per mL are shown in ***Extended Data Fig. S11C***, and normalisation to viable cell concentration (CFU equivalents) enables per-cell quantification of extracellular OMV accumulation during stationary phase ***(Extended Data Fig. S11B and S11D).*** From these data, an apparent net accumulation rate per viable cell can be estimated from the slope of the OMVs/CFU trace ***(Extended Data Fig. S11D).***

**Analysis method used for Extended Data Fig. S11:**

1. Determining the viable cell concentration of bacterial cultures (CFU equivalents)
2. Normalisation of Outer membrane vesicle concentration to viable cell concentration (CFU equivalents)

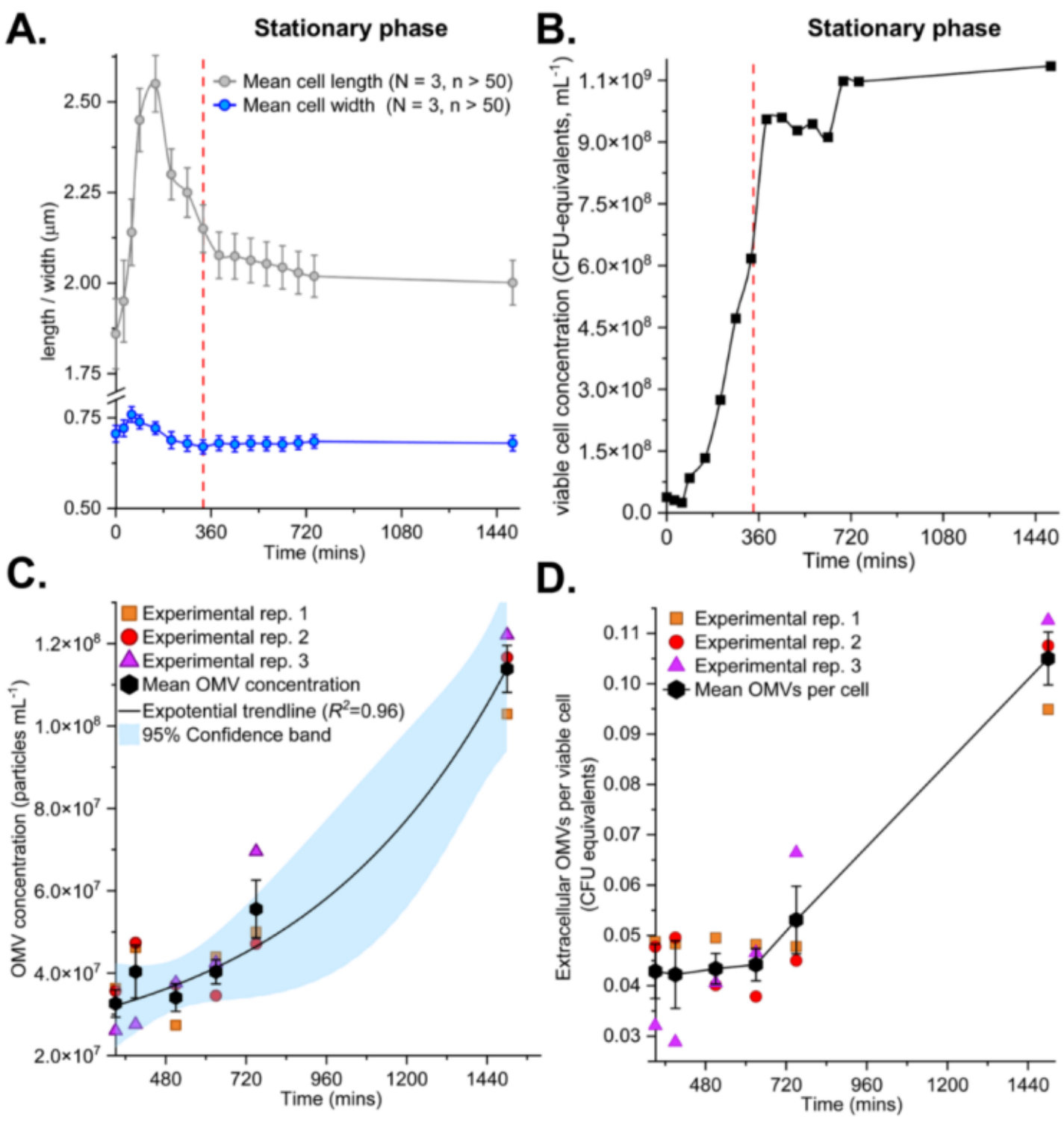


**New Extended Data Figure 11**

**4. Do this vesiculation rate quantitatively account for the rate of LPS loss as observed by the metabolic labelling?**

**Response**

We refer the reviewer to our response to point number 15 from reviewer 1 *'How much LPS is really lost in the OMVs? Is this consistent with the synthesis of "excess" LPS?'* in which we summarise the results and analyses from additional experiments carried out to answer these comments.

**5. I don't see any statistics of replicates or # of cells for Figures 6A-C**

**Response**

To address this, we have now:

i) Added n (= number of cells) and N (= independent biological replicates) reporting to the ***Figure 6A–C*** legend.

ii) Expanded the corresponding Results text ***(Outer membrane vesicle biogenesis contributes to LPS turnover in stationary phase E. coli cells)*** to state the replicate structure for live-cell 3D-SIM² (N = 3; ≥ 30 cells per replicate), to clarify that ***Figure 6A*** shows a single complete budding/release sequence (n = 1 scission event) while background-LPS–enriched OMV structures were repeatedly observed during time-course imaging, and to provide a conservative widefield OMV : cell ratio (14 structures adjacent to 72 cells)

iii) Reported n and N for the single-colour (n = 21, N = 3) and two-colour dSTORM datasets (n = 15, N = 3), including confirmation in dye swap experiments for the dual-colour enrichment.

iv) Added a new ***Extended Data Figure S12*** compiling additional live 3D-SIM² fields and expanded single- and dual-colour dSTORM examples.

v) Updated the Methods to explain how OMV-containing cells/fields were identified and to document the acquisition/analysis context for these datasets.

Together, we believe these changes provide clear replicate statistics and additional supporting information for ***Figures 6A–C***, while keeping the revisions proportionate to the referee's request.

**Adjusted legend for figures 6A-C**

**Figure 6: Preferential OMV-mediated clearance of background LPS from the OM of non-elongating, stationary phase *E. coli* cells.**

**A.** **Live-cell 3D-SIM² visualisation of OMV formation in a stationary phase *E. coli* cell presenting fluorescently labelled background LPS.** A depth-coded 3D projection from time-lapse imaging showing an AF488-LPS-enriched outer membrane vesicle budding from the cell surface and being released over a 10 min period (one complete budding-and-release sequence shown). Live cell 3D SIM$^2$ imaging was performed across N = 3 independent biological replicates (n ≥ 30 cells imaged per replicate). **Scale bars:** 1.0 µm. Additional examples of OMV-like structures captured during live cell 3D SIM$^2$ time course experiments are show in ***Extended Data Figs. S12A and S12B.***

**B.** **dSTORM images of stationary phase *E. coli* cells in the process of background LPS shedding via OMV biogenesis** (n = 21 cells, N = 3 independent biologically t replicates). **Scale bars:** 0.5 µm

**C.** **Background LPS is enriched in OMV-like protrusions during stationary phase.** Representative two-colour dSTORM images showing the spatial distribution of labelled, background (pre-existing) and labelled newly inserted LPS in the OM of *E. coli* cells during OMV formation. Background and newly inserted LPS were metabolically labelled with Kdo-alkyne and Kdo-$N_3$, respectively, using an *in vivo* pulse–chase method which enabled sequential CuAAC fluorescent labelling reactions (one dye per reaction) with complementary azide-($N_3$-) or alkyne-functionalised fluorophores. Background LPS (magenta in the left set of images and green in the right set of images) was enriched in the vesicle-like membrane protrusions (white arrows), while newly inserted LPS (green in the left set of images and magenta in the right set of images) was depleted. Images indicate spatial segregation of background and new LPS in the OM, with preferential incorporation of background LPS into membrane blebs in both dye orientations. This observation suggests stationary phase cells can employ OMV blebbing to facilitate removal of background LPS in the absence of cell elongation and division. This background-LPS enrichment of OMV-like protrusions was observed across all acquisitions and in both dye orientations ***(Extended Data Fig. S12D)*** (n = 15 cells, N = 3 independent biological replicates). Quantification of localisation-density enrichment in protrusion ROIs (OMV/bleb) versus intact cell ROIs across OMV-producing cells (n = 30, N = 3 independent biological replicates) is shown in ***Extended Data Fig. S13***, with per-cell values reported in ***Extended Data Table S24***. **Scale bars:** 0.5 µm.

**Key text that addresses reviewer comment.**

**Lines 457-467.** Using live-cell 3D-SIM², we directly visualised OMV biogenesis in stationary-phase cells presenting fluorescently labelled background LPS ***(Fig. 6A, Extended Data Figs. S12A and S12B)***. Across biologically independent live-cell imaging replicates (N = 3, ≥30 cells per replicate, ≥30 min time courses), we repeatedly observed the emergence of discrete, intensely fluorescent extracellular puncta and OMV-like structures adjacent to intact cells that exhibited substantially higher fluorescence than the contiguous OM signal, consistent with enrichment of labelled background LPS in vesicle-like structures ***(Extended Data Figs. S12A and S12B)***. In a representative widefield view, 14 visible OMV-like structures were observed in the vicinity of 72 intact cells (0.194 visible OMV-like structures per cell; ***Extended Data Fig. S12B)***, noting that this fluorescence-based count is a lower limit because the smallest vesicles fall below the practical resolution and sensitivity limits of fluorescence microscopy.

**Lines 470-473.** Capturing full scission events in 3D by live-cell super-resolution microscopy is technically challenging because vesiculation is transient and stochastic, and sufficient spatiotemporal resolution requires balancing illumination, acquisition speed and photophysics while maintaining cell viability.

**Line 474-496.** dSTORM imaging provided complementary support for OMV biogenesis and background-LPS shedding in stationary phase ***(Figs. 6B and 6C, Extended Data Figs. S12C and S12D)***. Unlike 3D-SIM², OMV-producing cells cannot be reliably identified during dSTORM acquisition, and vesicle-like protrusions and OMV-structures are typically confirmed only after localisation and reconstruction, necessitating screening of multiple cells and fields of view to capture vesiculation events. Despite this constraint, single-colour dSTORM frequently revealed LPS-rich puncta and vesicle-like protrusions consistent with nascent or recently released OMVs on stationary-phase cells (n = 21 cells, N = 3 biologically independent experiments, ***Fig. 6B and Extended Data Fig. S12C)***. Strikingly, in two-colour dSTORM experiments in which pre-existing background LPS and newly inserted LPS were differentially labelled, OMV-like protrusions were consistently enriched in labelled background LPS and depleted in labelled newly inserted LPS relative to the surrounding OM, and this enrichment was observed even after swapping the dye labels (n = 15 cells, N = 3 biologically independent experiments, ***Fig. 6C and Extended Data Fig. S12D)***.

**Added to methods section:**

1. **Time-lapse 3D SIM$^2$ imaging of live E. coli BW25113 cells with fluorescently labelled background LPS**

During live time-course imaging of stationary-phase cells presenting fluorescently labelled background Kdo-$N_3$-LPS, we repeatedly observed discrete, intensely fluorescent extracellular puncta/OMV-structures adjacent to intact cells, consistent with OMV-mediated preferential background-LPS clearance **(Extended Data Fig. S12A–B).** Imaging was performed across N ≥ 3 independent biological replicates with ≥ 30 cells imaged per replicate. Because many of the OMV dimensions fall below the effective diffraction-limited detection threshold in live-cell fluorescence imaging, we additionally provide a conservative, resolvable OMV : cell ratio from a representative widefield field-of-view (14 OMV structures adjacent to 72 intact cells; a ratio of 0.194 OMVs per cell; **Extended Data Fig. S12B)**.

2. **Two-colour dSTORM data acquisition with AF488- and AF647-labelled samples**

This two-colour acquisition workflow was also used to capture OMV-associated dual-colour examples comparing background versus newly inserted LPS **(Figure 6C and Extended Data Fig. S12D).**

3. **Processing of dSTORM data**

For OMV imaging by dSTORM, vesicle-like protrusions and extracellular puncta consistent with OMVs could not be reliably identified during acquisition and were typically confirmed only after localisation and reconstruction. OMV-containing examples were therefore obtained by screening multiple cells/fields, and the number of analysed cells (n) and independent biological replicates (N) are reported in the figure legends for **Figures 6B-C** and **Extended Data Figures S12C-D**.

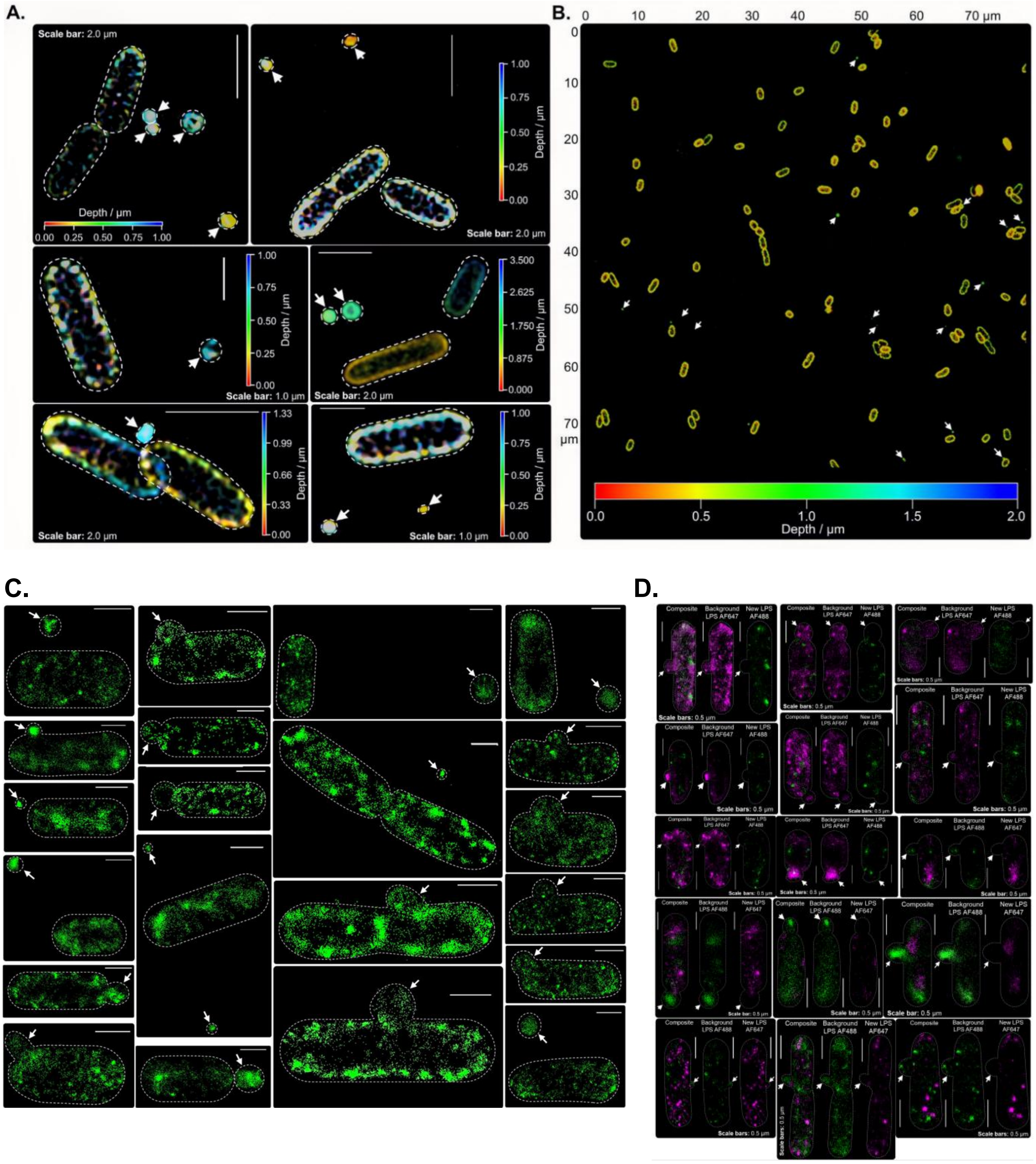


New Extended Data Figure S12.

**Minor comments**

**1. Figure 1E: Cells do not have right and left. Just use polar and non-polar.**

**Response**

Figure 1E and associated Extended Data Table S11 have been modified, replacing left pole with pole 1 and right pole with pole 2.

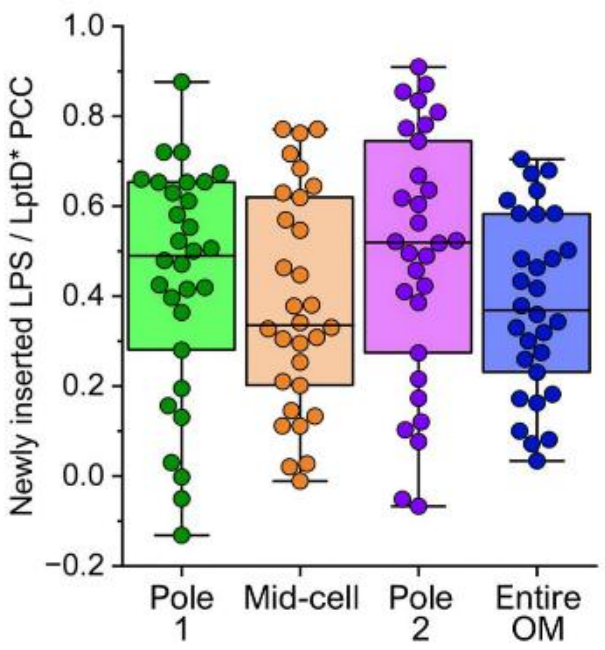


**Figure 1E** – with partial x-axis relabelling

**Extended Data Table S11:** Results from Mann-Whitney t-test assessing the significance of variation between LptD* vs newly inserted LPS Pearson correlation coefficient (PCC) values for polar, mid-cell and entire OM regions of *E. coli* BW25113 cells imaged via two-colour dSTORM after simultaneous dual fluorescent labelling of LptD* and newly inserted LPS

| Condition 1 | Condition 2 | $n_1$ | $n_2$ | Median $PCC_1$ | Median $PCC_2$ | *p* | U | Z |
|---|---|---|---|---|---|---|---|---|
| Pole 1 | Mid-cell | 30 | 30 | 0.49 | 0.34 | 0.32 | 518 | 0.99 |
| Pole 1 | Pole 2 | 30 | 30 | 0.49 | 0.52 | 0.46 | 399 | -0.75 |
| Pole 1 | Entire OM | 30 | 30 | 0.49 | 0.37 | 0.24 | 530 | 1.18 |
| Pole 2 | Mid-cell | 30 | 30 | 0.52 | 0.34 | 0.10 | 561 | 1.63 |
| Pole 2 | Entire OM | 30 | 30 | 0.52 | 0.37 | 0.07 | 574 | 1.83 |
| Mid-cell | Entire OM | 30 | 30 | 0.34 | 0.37 | 0.97 | 447 | -0.04 |

Likewise, ***Extended Data Figure S4A-F*** has also been modified replacing right  and left pole with polar region 1 and polar region 2, respectively.

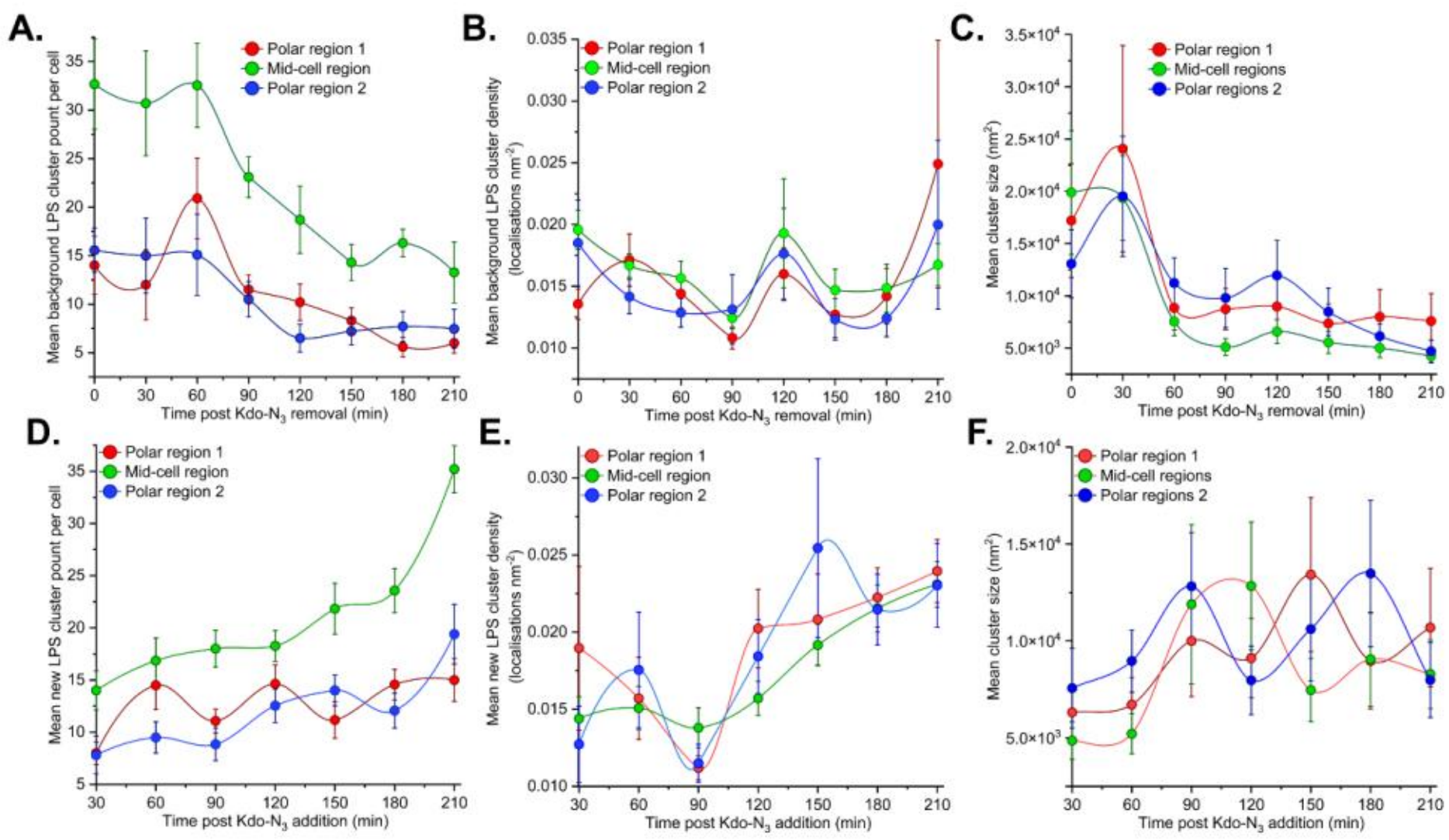

**2. Figure 2: The analysis of "overlap" is not very informative with respect to physiological function. With STORM-level resolution, we expect very little overlap since LPS cannot actually be made on top of other LPS, so the only overlap comes from the noise in the measurement**.

**Response**

We agree that new LPS cannot physically be inserted 'on top of' or directly 'below' pre-existing / old / background LPS molecules within the same leaflet. We have therefore revised the text and figure annotations to clarify that our analysis does not interpret this measurement as molecular co-occupancy. Instead, we segmented background- and newly inserted-LPS domains independently in each channel using DBSCAN and quantified a mask-based DBSCAN cluster-mask intersection (geometric intersection of the two DBSCAN-defined cluster regions, normalised by the combined cluster area). This provides a conservative, resolution-limited descriptor of domain proximity/co-occurrence at the analysis scale (set by $\varepsilon$ = 30 nm and localisation precision), rather than a claim that newly inserted LPS is deposited directly on top of or underneath pre-existing LPS. Consistent with the expectation of minimal mixing, this cluster-area intersection metric remains low across the time course ***(Fig. 2F; Extended Data Fig. S2E)***, and the principal conclusion of persistent spatial segregation is additionally supported by the consistently negative Pearson correlation coefficients ***(Fig. 2D)*** and dye-orientation controls ***(Fig. 2B–C; Extended Data Fig. S2D)***. To prevent misinterpretation, we have:

**i)** Relabelled ***Fig. 2F*** (*y*-axis and legend) and ***2A*** schematic annotations to use 'cluster-mask intersection'/'mask intersection fraction' terminology

**ii)** Removed over-interpretation of curve-fitting in the legend/text

**iii)** Added a brief clarification in the **Methods** defining the metric and its interpretation as a resolution-limited proximity/co-occurrence descriptor rather than molecular overlap. We also ensured the OM-boundary buffering parameter used for mask generation is reported consistently between code and Methods.

**Key text that addresses reviewer comment.**

**Lines 223-233.** To complement this coverage analysis with a cluster co-occurrence metric, we quantified the normalised DBSCAN cluster-area intersection (cluster-mask intersection fraction; geometric intersection of the two independently segmented cluster masks) for labelled background and newly inserted LPS ***(Fig. 2F and Extended Data Fig. S2E)***, calculated as the intersection area divided by the sum of the background- and newly inserted-cluster areas. Because this metric is defined at the cluster-mask/analysis scale (set by $\varepsilon$ and localisation precision), it reports cluster-mask 'co-occurrence' or 'proximity' rather than molecular co-occupancy. This cluster-area intersection metric remained low throughout the time course, showing only a modest transient maximum around 60 min before decreasing again, indicating persistent spatial segregation and limited cluster-mask co-occurrence ***(Fig. 2F)***.

### Methods > Two-colour dSTORM DBSCAN cluster segmentation and cluster-area intersection analysis

Spatial domains of background, (pre-existing) and newly inserted LPS were identified independently using a tailored Density-Based Spatial Clustering of Applications with Noise (DBSCAN) algorithm (ε = 30 nm, min_samples = 7) [25], widely adopted for co-localisation analysis of multi-channel SMLM data [26]. The ε value equals 1.5 × the median nearest neighbour distance between LPS localisations under our imaging conditions (median = 22 nm) and reflects the average precision associated with individual localisations (~30 nm), balancing sensitivity to genuine nanoscale domains with robustness to photon shot noise. A min_samples threshold of seven exceeds the upper quartile localisation count for single fluorophore bursts, limiting false positive clusters. Cluster polygons were clipped to the OM boundary and combined to obtain total old and new LPS cluster areas; their geometric intersection (background LPS ∩ newly inserted LPS) defined the cluster-area intersection (mask-intersection) region. Because ε is set on the order of the effective localisation precision, this mask-intersection metric reports cluster-mask co-occurrence/proximity at the analysis scale, rather than molecular co-occupancy. The script produces several output files. LPS_output_summary.csv contains numerical values for the OM area, individual and combined LPS areas, localisation counts within the mask-intersection region, and normalised coverage metrics; these underpin the quantitative time course displayed in **Figs. 2E** and **2F** and are reproduced in **Extended Data Table S13**. combined_overlap_map.png is a high resolution composite image showing all localisation points, their DBSCAN assigned clusters and the spatial overlap; the map provides morphological context for the representative cells provided in **Extended Data Fig. S2E** (Note that 'overlap' in these outputs refers to geometric overlap of independently segmented cluster masks at the analysis scale, not physical co-occupancy of LPS molecules)**.** Cluster size distributions are exported as old_lps_cluster_histogram.csv and new_lps_cluster_histogram.csv, enabling independent verification that old and new clusters are sampled from comparable size ranges. Examples of individual channel input dSTORM data for each time point (DBSCAN_new_old_LPS_dSTORM_input_folder), Python script (Python_code_New_Old_LPS_DBSCAN.txt) and associated READ.ME_Python_code_New_Old_LPS_DBSCAN file, example individual cell OM output data files (DBSCAN_new_old_LPS_outputs_folder) along with summary data output tables (DBSCAN_new_old_LPS_individual_cell_summary.xlsx and **Extended Data Table S13)** are provided as Supplementary Software and Supplementary Data, respectively.

**3. I believe what we would like to understand is the dynamics of LptD/new LPS patches. How long is a single "patch" made for before the synthesis machinery moves on to synthesize a new patch, and how big do the patches get during this time?**

**Response**

We agree this is an important mechanistic question. In the present study, our dual-colour dSTORM measurements quantify the spatial organisation, domain size and heterogeneity of LptD*–new LPS co-enriched insertion domains, providing a quantitative estimate of their characteristic lateral scale ($r_{1/2}$ max =181 nm) and showing that site activity is heterogeneous across the OM. We note that the time-resolved dynamics of individual Lpt engagement states have been addressed by recent single-molecule tracking work from the Kahne/Garner/Bernhardt teams, which supports highly dynamic, transient Lpt bridge states on the timescale of seconds**.** We have added a brief statement and citation in the Results section to contextualise our spatial findings within this established framework.

**Key text that addresses reviewer comment.**

**Lines 166-171.** These analyses constrain the spatial scale of LptD*–new LPS co-enriched insertion regions ($r_{1/2}$ max = 181 nm) and reveal heterogeneous site activity across the OM, supporting dynamic engagement of Lpt complexes. In keeping with this interpretation, recent single-molecule tracking of Lpt transport components supports highly dynamic bridge states that persist for a few seconds, providing a framework for future live-cell measurements of insertion-site dwell times and patch growth kinetics [36].

**Reference**

36 Törk, L., Moffatt, C. B., Bernhardt, T. G., Garner, E. C. & Kahne, D. Single-molecule dynamics show a transient lipopolysaccharide transport bridge. *Nature* **623**, 814-819 (2023).

**4. Line 305: "Kinetically-trapped mosaic of spatially segregated domains." This is an overly convoluted statement. In biology, domains usually imply that there is something different about the domains. From the present analysis, there are no functional domains. The analysis reinforces the current understanding of the outer membrane as a growing solid – is there any difference between "kinetically trapped" and "solid"?**

**Response** We agree that our original phrasing was unnecessarily complex and that the term "domains" could be misconstrued as implying functionally distinct compartments. We have revised the Results text to remove "mosaic" and "domains" terminology and instead describe the OM more clearly as diffusion-limited, with persistent, insertion-patterned spatial heterogeneity. This wording better reflects our measurements, which quantify spatial segregation and limited mixing/coarsening of LPS-rich regions over time, without implying specialised functional domains. The revised text also clarifies that by "solid-like" we mean an OM in which lateral diffusion is sufficiently restricted on experimental timescales that insertion patterns relax only slowly, consistent with an insertion-trapping model.

**Key text that addresses reviewer comment.**

**Lines 48-51**. A direct implication of this 'solid-like' behaviour is that OM expansion during growth cannot be achieved by rapid lateral equilibration, but instead must rely on spatially localised insertion of new material into an otherwise diffusion-limited outer leaflet.

**Lines 212-213.** … supporting a diffusion-limited organisation rather than an equilibrium-like compositional ordering regime in the *E. coli* OM over these timescales.

**Lines 335-341.** Because both LPS and OMPs exhibit very limited lateral mobility *in vivo*, any compositional ordering may be strongly diffusion-limited and therefore slow to relax on experimental timescales [10,11,32]. Nevertheless, an equilibrium-like ordering regime makes the clear, testable prediction that once compositionally distinct regions form, classical models of phase-ordering kinetics predict coarsening, in which smaller regions disappear while larger regions grow through coalescence and Ostwald ripening, yielding fewer, larger regions at later time points [42-48].

**Lines 365-371.** In contrast, across our conditions and time window, both localisation-density-defined background and newly inserted LPS pools lacked any coarsening signature, indicating that LPS lateral mixing is strongly constrained and domain coalescence is suppressed. This behaviour is compatible with a solid-like, diffusion-limited regime in which LPS-rich patches are stabilised rather than undergoing fusion-driven growth [10-13], arguing against equilibrium phase ordering as the dominant mechanism in our system.

**Lines 396-405.** These results ***(Fig. 4 and Extended Data Fig. S4)*** argue against *E. coli* OM organisation being governed primarily by equilibrium phase ordering with domain coarsening. Instead, our data support a solid-like, diffusion-limited OM with persistent, insertion-patterned spatial heterogeneity. The Kdo-$N_3$-labelled background LPS pool (as measured by fluorescence/localisation density) progressively decreases without large-scale mixing or coarsening of LPS-rich regions ***(Fig. 4B)***, while newly inserted LPS accumulates in discrete, stable patches ***(Fig. 4A)***. This behaviour is consistent with insertion trapping, in which newly inserted LPS becomes effectively constrained upon incorporation rather than laterally redistributing to equilibrate the membrane. This restricted-mixing regime is directly observed for LPS [10-13,33].

**Lines 686-687.** Instead, the OM maintains a solid-like, diffusion-limited organisation in which insertion-patterned LPS-rich patches are preserved over time.

**Lines 807-809.** This is inconsistent with Ostwald ripening and other phase separation phenomena, and is better described by a diffusion-limited, far-from-equilibrium process.

**5. Line 332: Replace "electromagnetic radiation" with "light".**

**Response**

This has been actioned as per reviewer instructions.

**Key text that addresses reviewer comment.**

**Lines 428-430.** Rates of cell elongation and division were assessed ***(Extended Data Fig. S5D)*** to ensure that growth was unaffected by labelling or light exposure under the imaging conditions used.

**6. Figure 3B vs 6F: stationary phase is defined very differently. Since it is an important concept in the model, it would be good to provide a single working definition of it and analyse all the data accordingly.**

**Response**

We agree that a single working definition of stationary phase improves clarity across datasets. We have therefore standardised growth-phase assignment throughout the manuscript using the same operational $OD_{600}$-based criteria for all experiments. Specifically, we now define the onset of stationary-phase as the transition point at which the $OD_{600}$ growth curve first departs from exponential increase and begins to decelerate toward an asymptote, determined from successive $OD_{600}$ measurements of the same culture (**Methods, 'Growth phase assignment').** We also clarify that the apparent difference in the absolute time-to-stationary phase between Fig. 3B and Fig. 6F arises from different initial culture densities required for the respective workflows (e.g. $OD_{600}$ ≈ 0.5 for pulse–chase labelling/imaging to ensure sufficient biomass versus $OD_{600}$ ≈ 0.1 for time courses with OMV sampling for NTA to capture the full growth trajectory). Importantly, despite these different starting points, phase of growth assignment uses the same $OD_{600}$-criteria in all cases**,** and we now direct readers to the underlying growth curves/tables supporting these assignments ***(Extended Data Fig. S3C–D and S11B; Extended Data Tables S22–S23)***.

**Methods > Growth phase assignment**

For all experiments, 'exponential', 'late exponential', and 'stationary phase' were assigned from the $OD_{600}$ growth curves constructed from $OD_{600}$ measurements of the sample culture, with stationary phase onset defined operationally as the transition point at which the $OD_{600}$ growth curve first departed from exponential increase and began to decelerate toward a plateau, as determined from successive $OD_{600}$ measurements of the same culture. Because some assays were initiated at different starting optical densities for practical reasons (e.g. $OD_{600}$ ≈ 0.5 for pulse–chase labelling/imaging versus $OD_{600}$ ≈ 0.05 for NTA), the absolute time-to-stationary differs between assays, but the phase assignment uses the same $OD_{600}$-plateau criterion (see ***Extended data figs. S3C-D, S11B and Extended data tables S22–S23)***. Over the analysed stationary-phase time window, viable cell counts (CFU $mL^{-1}$) remained approximately stable ***(Extended data fig. S11 and Extended data table S23)***, indicating minimal loss of viability under these conditions.

**Reviewer #4 (Remarks to the Author):**

Christoph Baumann and collaborators present a communication titled "Persistent LPS insertion, spatial segregation and vesicle biogenesis drive growth-independent adaptation of the Escherichia coli outer membrane".

This manuscript presents the use of chemical biology tools, namely metabolic lipopolysaccharide labelling and genetic code expansion, to study the dynamics of lipopolysaccharide insertion, diffusion and release within the Outer Membrane of Gram-negative bacteria, using Escherichia coli as a relevant model and Super Resolution Microscopy (dSTORM) for nanometric imaging.

This very thorough and well conducted study combines all the available tools, including mathematical modelling, to better understand these processes which are fundamental to the study of Gram-negative bacteria, with potential long-range impact in terms of anti-bacterial strategies.

The authors show convincing evidence that LPS is inserted into the membrane in well-localized sites, that LPS does not diffuse laterally within this membrane, that new and old LPS are segregated locally within different patches, and that older LPS is released from the membrane via localized formation of Outer Membrane Vesicles, ensuring constant LPS renewal even when the bacterium is not elongating or dividing.

This study is very convincing, and, I believe, should be accepted in Nature Communications as soon as some minor remarks have been addressed.

**Points to author**

**1. The main concern relates to experiments depicted in Figure 1 and the "simultaneous New LPS &LptD* in situ fluorophore conjugation". The caption states: "Simultaneous CuAAC 'click' chemistry enabled orthogonal fluorescent tagging of LptD* and newly inserted LPS using azide- and alkyne-functionalised dye pairs". Strictly simultaneous CuAAC with the 2 fluorophores does not seem feasible, since the fluorophore-azide should react with the fluorophore alkyne. The supplementary Methods are not clear either on this aspect: "The washed cell pellet was then resuspended to an OD600 of 1.0 in 'Click-iT' mix charged with either AF488- or AF647-alkyne, transferred to a 2 mL microcentrifuge tube, and incubated at room temperature for 30 min on a rotary wheel (12 rpm, 80° incline relative to benchtop) protected from light." – Only AF488- or AF647-alkynes are mentioned, no azide and no sequential process – Could the authors clarify? Were these fluorophore conjugations realized stepwise? This is very important to describe a very clear protocol here. The same applies to the experiment depicted in figure 2.**

**Response**

We agree that the wording ‘simultaneous CuAAC’ was imprecise. In our experiments, dual-colour labelling was performed stepwise using two sequential CuAAC reactions (one fluorophore type per reaction, with intermediate washes so azide($N_3$)- and alkyne-functionalised fluorophores were never present together in the same CuAAC mixture. We have therefore revised the manuscript to make the workflow explicit and unambiguous.

Specifically, we have:

**i)** Replaced ‘simultaneous CuAAC’ with ‘sequential CuAAC’ in the ***Figure 1*** legend and text and updated the schematic label accordingly.

**ii)** Rewrote the relevant Methods text for both the LptD* / newly inserted LPS dual-labelling workflow ***(Figure 1)*** and the Kdo-alkyne / Kdo-$N_3$ pulse–chase dual-LPS workflow ***(Figure 2)*** to state explicitly that two CuAAC reactions were carried out sequentially with wash steps between reactions.

**iii)** Clarified that labelling order and dye identity were alternated across independent replicate experiments to verify that neither dye identity nor labelling order influenced the observed spatial distributions ***(Extended Data Tables S6 and S12)***. Together, these changes address the reviewer’s concern and provide a clear, concise methodological description for both figures**.** We also note that ‘simultaneous’ is now used only in the sense of simultaneous two-colour visualisation after sequential labelling.

**Key text that addresses reviewer comment.**

**Lines 185-189.** Differential fluorescent labelling was performed by two sequential CuAAC reactions (one dye type per reaction, with washes between reactions) using distinct azide (-$N_3$) and alkyne functionalised fluorescent dyes, enabling simultaneous two-colour visualisation of both LPS subpopulations in separate fluorescence channels.

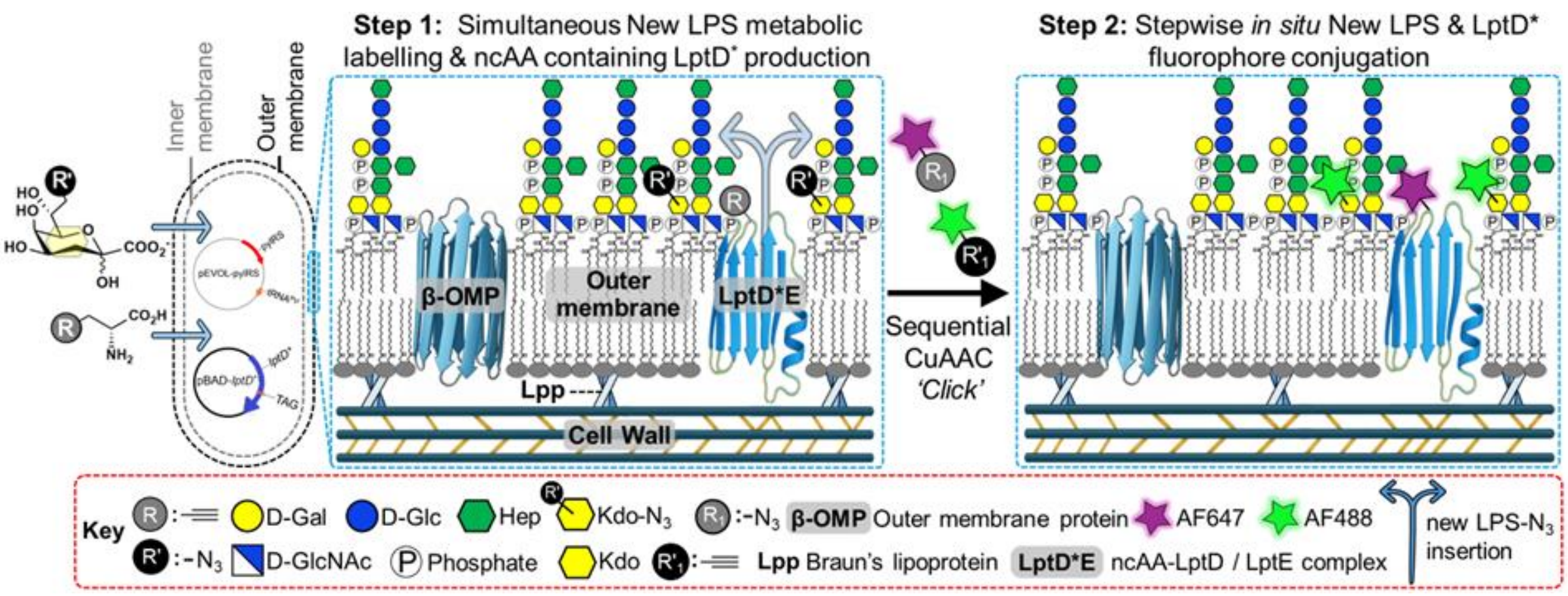


**Change to Figure 1A Legend.** Dual-colour differential fluorescent labelling was performed by two sequential CuAAC *(‘click’)* reactions (one dye type per reaction, with washing between steps between reactions), using an alkyne-functionalised dye to label Kdo-$N_3$–LPS and an azide (-$N_3$)-functionalised dye to label LptD*. Labelling order and dye identity were swapped between replicates ***(Extended Data Table S6)***.

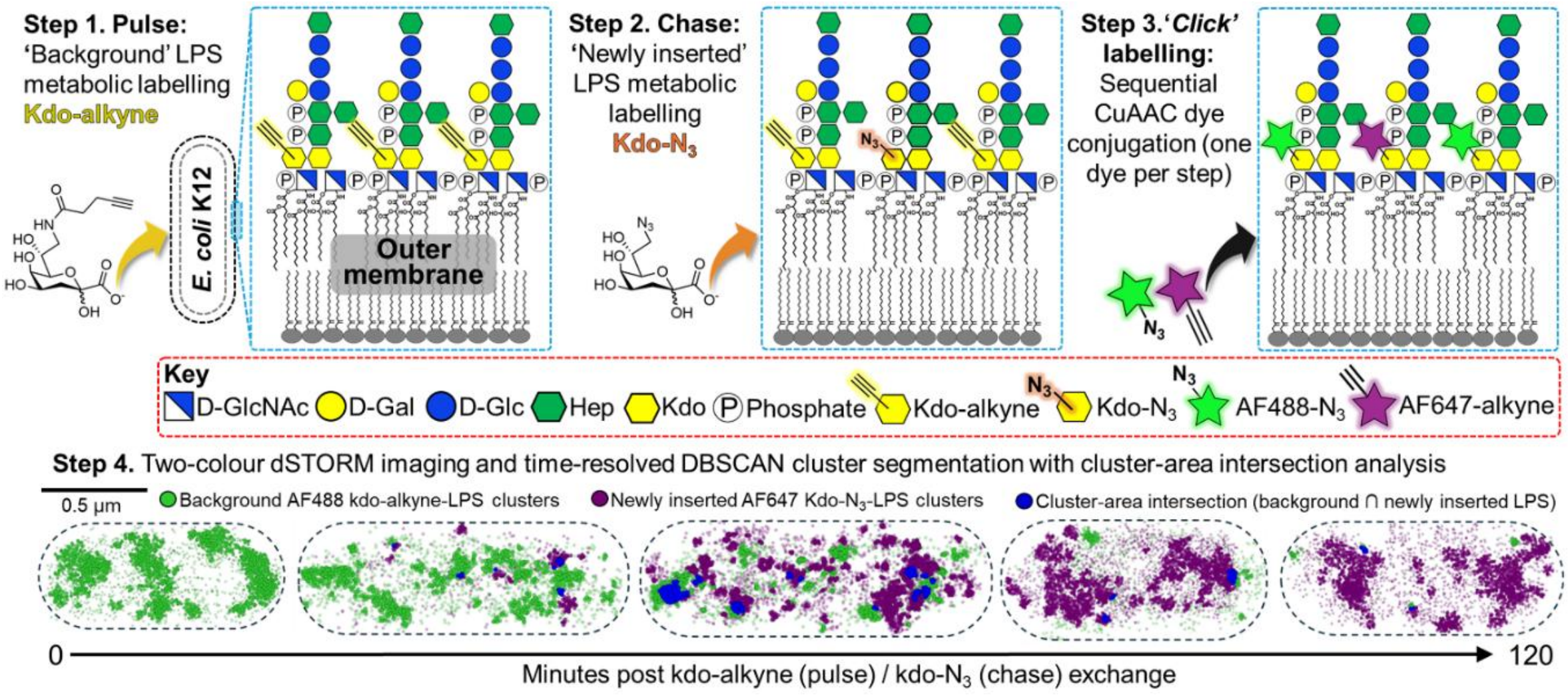


**Change to Figure 2A Legend** Kdo-$N_3$-LPS are differentially fluorescently labelled by two sequential CuAAC reactions (one dye type per reaction) using azide(-$N_3$)-functionalised dyes to label background Kdo-alkyne-LPS and alkyne-functionalised dyes to label newly inserted Kdo-$N_3$-LPS.

## Modified methods > Dual differential fluorescent labelling of LptD* and newly inserted Kdo-$N_3$-containing LPS

In the first step, cells were resuspended to an $OD_{600}$ of 1.0 in 'Click-iT' mix charged with a single complementary fluorophore (either an azide(-$N_3$) or alkyne-functionalised AF488/AF647 dye, matched to the relevant bio-orthogonal handle; Kdo-$N_3$-labelled LPS was labelled with an alkyne-functionalised dye and LptD* (propargyl-L-Lysine) was labelled with an azide(-$N_3$) functionalised dye), transferred to a 2 mL microcentrifuge tube, and incubated at room temperature for 30 min to ensure efficient CuAAC labelling on a rotary wheel (12 rpm, 80° incline relative to benchtop) protected from light. Cells were then pelleted and washed to remove unreacted dye before performing a second CuAAC reaction with the complementary dye to label the remaining handle. Cells were kept on ice in pre-chilled tubes/buffers except during the CuAAC incubations. In replicate experiments, the newly inserted LPS and LptD* labelling order was alternated, as were the dye combinations ***(Extended Data Table S6)***. This labelling strategy was implemented to confirm that the order in which species were labelled and the dye combinations used did not influence the apparent spatial distributions and relative concentrations of LptD* and newly inserted LPS ***(Fig. 1B and Extended Data Fig. S1A)***.

**Modified methods > Monitoring changes in background and newly inserted LPS distributions over time in the OM of *E. coli* BW25113 cells via Kdo-alkyne / Kdo-$N_3$ pulse-chase experiments followed by two-colour dSTORM characterisation**

Washed pellets from each time point were labelled by two sequential CuAAC reactions (one dye per step). For the first step, cells were resuspended to an OD600 of 1.0 in 'Click-iT' mix containing a single complementary fluorophore (either an alkyne-functionalised AF488/AF647 dye to label Kdo-$N_3$ (newly inserted LPS), or an azide (-$N_3$)-functionalised AF488/AF647 dye to label Kdo-alkyne (background LPS)), transferred to a 2 mL microcentrifuge tube, and incubated at room temperature for 30 min on a rotary wheel (12 rpm, 80° incline) protected from light. In replicate experiments, the newly inserted LPS and pre-existing, background LPS labelling order was alternated, as were the dye combinations **(Extended Data Table S12)**. This alternative labelling strategy was implemented in the experimental replicates to ensure that the order in which species were labelled and dye combination used did not influence the spatial distributions and relative concentrations of background and newly inserted LPS **(Figs. 2B and 2C)**.

**Modified extended data tables to make sequential labelling protocol clearer for reader**

**Extended Data Table S6.** Dual-colour CuAAC labelling order and dye combinations for LptD* and newly inserted LPS (Kdo-$N_3$) used to confirm that dye identity and labelling order do not affect two-colour dSTORM results

| **First CuAAC step:** **Labelled species / fluorescent dye** | **Second CuAAC step:** **labelled species / dye** |
|---|---|
| LptD* / AF488-$N_3$ | Newly inserted LPS / AF647-alkyne |
| Newly inserted LPS / AF647-alkyne | LptD* / AF488-$N_3$ |
| LptD* / AF647-$N_3$ | Newly inserted LPS / AF488-alkyne |
| Newly inserted LPS / AF488-alkyne | LptD* / AF647-$N_3$ |

Alkyne-functionalised dyes label Kdo-$N_3$, and $N_3$-functionalised dyes label the alkyne handle on LptD*

**Extended Data Table S12.** Dual-colour CuAAC labelling order and dye combinations for background (Kdo-alkyne) and newly inserted (Kdo-$N_3$) LPS used to confirm that dye identity and labelling order do not affect two-colour dSTORM results.

| **First CuAAC step: labelled species / dye** | **Second CuAAC step: labelled species / dye** |
|---|---|
| Newly inserted LPS / AF488-alkyne | Background LPS / AF647-$N_3$ |
| Background LPS / AF647-$N_3$ | Newly inserted LPS / AF488-alkyne |
| Newly inserted LPS / AF647-alkyne | Background LPS / AF488-$N_3$ |
| Background LPS / AF488-$N_3$ | Newly inserted LPS / AF647-alkyne |

Alkyne-functionalised dyes label Kdo-N3, and azide-functionalised dyes label Kdo-alkyne

**2. I am not an expert, but I thought that during the stationary phase, bacteria would continue dividing, but their division would be counter-balanced by bacterial death, limiting population growth. Do bacteria also stop dividing or slow down their division rate? Could the author add a short comment on this?**

**Response**

Stationary phase can reflect different physiological regimes depending on growth conditions and the time window analysed (e.g., reduced division balanced by death versus strongly reduced net growth with minimal loss of viability). In our study, we defined stationary phase operationally as the point at which the $OD_{600}$ growth curve first departed from exponential increase and began to decelerate toward an asymptote ***(Extended Data Fig. S3, Extended Data Tables S22 and S23)***. Importantly, over the stationary phase of growth time window analysed here, viable cell counts (CFU $mL^{-1}$) remained approximately stable ***(Extended Data Fig. S11, Extended Data Tables S22 and S23)***, indicating minimal loss of viability. We did not directly measure single-cell division rates in this work; however, the $OD_{600}$ asymptote together with a stable CFU value indicates that net population growth is negligible over this window. We have therefore added a short statement in the Results and Methods clarifying that, in this context, 'stationary phase' corresponds to strongly reduced net growth (i.e. greatly slowed population expansion) rather than population stagnation dominated by cell death.

**Key text that addresses reviewer comment.**

**Lines 447-450.** Over the time window analysed, CFU $mL^{-1}$ remained approximately stable ***(Extended Data Fig. S11, Extended Data Table S23)***, indicating minimal net loss of viability; thus, 'stationary phase' here reflects negligible net population growth rather than population stagnation dominated by cell death.

**Methods > Growth phase assignment.** Over the analysed stationary-phase time window, viable cell counts (CFU $mL^{-1}$) remained approximately stable ***(Extended data fig. S11 and Extended Data Table S23)***, indicating minimal loss of viability under these conditions. Accordingly, net population growth is negligible over this window (OD600 plateau), and we treat growth-associated dilution as minimal.

3. Figure 7: In the Particle-based simulations, what is the meaning of Black/vacant spaces? There should be some phospholipids present in that space, right?

**Response**

In the particle-based simulations, the black ('vacant') regions are not intended to represent empty membrane. Rather, they indicate areas not occupied by the simulated/visualised LPS particles, corresponding to non-LPS OM components that are not directly tracked in the simulation output. In our model, these regions represent OMP-rich (protein-dense) areas/obstacles that exclude LPS from the simulated map and therefore appear black in the rendered output. We have clarified this point in the Figure 7 legend and associated Methods text.

With respect to phospholipids, we agree that they are a major component of the inner leaflet, but the native Gram-negative OM is very asymmetric and the outer leaflet is composed almost entirely of LPS. The phospholipid content of the outer leaflet is expected to be extremely low in the native OM of wild-type *E. coli* cultured under standard laboratory conditions.

**Key text that addresses reviewer comment.**

**Figure 7B Legend... Black**: LPS-inaccessible, OMP-rich obstacle regions (~25% area) in the static mask; these pixels are rendered black because they are excluded from the simulated LPS particle field.

**Modified Methods / Simulation domain, mask and coarse-graining.**

The remaining 25% represented OMP-occupied, LPS-inaccessible excluded/obstacle regions (rendered as black in figures; sometimes referred to as "voids" in the simulation mask) consistent with OMP islands and the confined lateral mobility of OMPs and LPS in the E. coli OM [3,15].

**4. Line 556: "a LPS molecule" instead of "an LPS molecule".**

**Response.** This change has been made

**5. Some references are missing:**

**(i) For Kdo-$N_3$ labelling of E. coli via SPAAC: https://doi.org/10.1371/journal.pone.0127700**

**(ii) Molecular proof of Kdo-$N_3$ incorporation in the LPS inner core: https://doi.org/10.1074/jbc.M117.814962**

**(iii) Super-resolution imaging of *E. coli* after metabolic labelling using Kdo-$N_3$: https://doi.org/10.1038/s41467-019-09901-8**

**Response**

These references have now been cited at the appropriate points in the Results section of the main text.


27 Nilsson, I. *et al.* Molecular characterization and verification of azido-3, 8-dideoxy-d-manno-oct-2-ulosonic acid incorporation into bacterial lipopolysaccharide. *J. Biol. Chem.* **292**, 19840-19848 (2017).

37 Cabriel, C. *et al.* Combining 3D single molecule localization strategies for reproducible bioimaging. *Nat. Commun.* **10**, 1980 (2019).

58 Fugier, E. *et al.* Rapid and specific enrichment of culturable gram negative bacteria using non-lethal copper-free click chemistry coupled with magnetic beads separation. *PLoS One* **10**, e0127700 (2015)

**RESPONSE TO REVIEWER COMMENTS**

**Reviewer #2 (Remarks to the Author):**

**1. We asked for a simple control experiment where fixed bacteria should in addition to click-labelling be stained with antibodies against LPS or LptD to check whether and how either of these components when antibody-stained overlaps with its respective click labelled component. We did not ask for LPS and LptD colabelling, and therefore the effect of staining of one of these components cannot affect the localization or function of the other component?! Also, a linkage error of at most 25 nm (given that primary and secondary antibody are used), which could also be reduced to about 10nm in the case of primary antibody and secondary nanobody are used, will not have a significant impact when checking the overlap of the two signals (click-labelled and antibody labelled). Further, even if antibody might in some instances not bind to "shielded" antigen, every antibody-stained antigen should overlap with the corresponding click labelled antigen. The authors also argue that there are no working antibodies against LptD. Have they e.g., tried to get a hold of the LptD-AF488 Fabs as described in PMID: 31237236 and 40030021.**

**RESPONSE:**

We thank the reviewer for clarifying the requested control experiment. We now understand that the requested experiment was an antibody-versus-click comparison for each individual target, rather than a combined LPS / LptD antibody co-labelling experiment. We agree that such an experiment would provide a useful orthogonal validation of the click-labelling strategy, provided that suitable validated antibody / Fab reagents are available for intact, fixed *E. coli* K-12 BW25113 cells.

Following the reviewer's recommendation, we made targeted attempts to identify and obtain appropriate reagents for both proposed immunofluorescence controls. For LPS, we were unable to identify an antibody validated for reliable immunofluorescent labelling of the exposed native rough LPS structure at the surface of intact fixed *E. coli* K-12 BW25113 cells. Although commercial antibodies against rough *E. coli* LPS are available, the key requirement for this control is not simply recognition of purified or extracted LPS, but robust detection of surface-accessible LPS in the context of the intact bacterial outer membrane. We also consulted colleagues with expertise in *E. coli* LPS and outer-membrane biology, including Prof. M. van der Woude (University of York) and Prof. C. Kleanthous FRS (University of Oxford), but did not identify a suitable validated reagent for this application.

To our knowledge, there are no published examples of an antibody being used for fluorescence microscopy to label the native, surface-accessible oligosaccharide core of rough LPS in intact *E. coli* K-12 cells. Consequently, any candidate antibody would require substantial validation before it could serve as a meaningful orthogonal control for Kdo-$N_3$ labelling. We

therefore could not perform a reliable LPS antibody-versus-click comparison during the manuscript revision period.

We have instead ensured that the manuscript clearly documents the controls supporting the specificity of the Kdo-$N_3$ / Kdo-alkyne metabolic LPS labelling strategy. Kdo-based metabolic labelling of the LPS inner core is a well-established approach for visualising bacterial LPS in intact cells and has been used in multiple studies of LPS incorporation, localisation and dynamics *(Dumont et al., 2012; Nilsson et al., 2017; Kumar et al., 2025)*. This view was also supported by expert advice received during the revision period; when consulted about commercial LPS antibodies for intact-cell fluorescence labelling of rough LPS, Prof. C. Kleanthous FRS advised that his group does not use commercial LPS antibodies for cell labelling and considers click-based labelling to be superior for this application. In addition, the same Kdo-$N_3$ / click-labelling / dSTORM workflow used here was validated in our previous work in intact *E. coli* K-12 cells *(Nabarro et al., 2026)*, including controls demonstrating that fluorophore labelling was dependent on the Kdo-$N_3$ metabolic handle. In the present manuscript, we include further reciprocal dye controls, Kdo-$N_3$ / Kdo-alkyne comparison experiments, and mobility / organisation controls showing that Kdo-analogue incorporation and click-labelling do not measurably perturb OM organisation or homeostasis. These combined controls support the specificity and suitability of the metabolic labelling strategy for the analyses presented here.

We followed the recommendation of this reviewer and attempted to secure an aliquot of the Alexa Fluor 488-labelled monoclonal LptD Fab antibody used in Kumar *et al*. *(2025)*. The corresponding author’s group in Oxford could not provide us with the antibody / Fab when contacted. We then contacted the researcher at Genentech Inc. who supplied the Oxford group with the Alexa Fluor 488-labelled monoclonal LptD Fab antibody used in their published work. Dr. S. Rutherford confirmed that they did not currently have fluorescently labelled or unlabelled LptD antibody available to share. Therefore, despite attempting to obtain the relevant reagent directly from the groups associated with its generation and recent use, we were unable to source a suitable LptD antibody / Fab for the requested control experiment within the manuscript revision period.

In light of this, we note that while immunofluorescent visualisation would provide additional supporting evidence, the robustness of our LptD labelling strategy using a non-canonical amino acid approach is supported by prior functional and biochemical validation. Notably, in previously published work *(Nabarro et al., 2026)*, we validated non-canonical amino acid incorporation into recombinant *E. coli* LptD labelled in surface loop 9, termed LptD*, by demonstrating formation of a functional complex *in vivo* with LptE and restoration of OM barrier function and wild-type-like LPS lateral mobility in the LptD-defective *E. coli imp*4213 strain.

Alexa Fluor 488-labelled LptD* isolated from *E. coli* K-12 cell lysates formed a stable complex with LptE that could be observed by SDS-PAGE in unheated samples but was absent in heated samples *(Appendix Fig. S4B in Nabarro et al., 2026, and Extended Data Fig. S1C in this manuscript).* This standard approach for detecting LptD/LptE complex formation was developed

and used by the Silhavy and Kahne groups *(Chng et al., 2010; Freinkman et al., 2011)*, who also showed that incorporation of a non-canonical amino acid enabled mapping of LptD/LptE interactions by *in vivo* photo crosslinking *(Freinkman et al., 2011)*.

In Nabarro et al. *(2026)*, we also demonstrated that native OM barrier function and LPS lateral mobility could be restored in *imp*4213 *E. coli* cells expressing the *lptD** gene *(Appendix Fig. S4C and Fig. 3D, respectively, in Nabarro et al., 2026)*, but only when the bacterial culture was supplemented with the appropriate non-canonical amino acid (L-Lys(Boc)-OH or *N*-propargyl-L-Lys-OH). Furthermore, the distribution of LptD* observed here in the outer membrane of wild-type *E. coli* K-12 cells from mid-log culture (*Fig. 1B and Extended Data Fig. S1A in this manuscript)* is highly consistent with prior work using the Alexa Fluor 488-labelled monoclonal LptD Fab antibody for detection of wild-type LptD in the same bacterial strain by 3D-SIM *(Supplementary Fig. 12 in Kumar et al., 2025)*. We cited this published work in our manuscript and noted the close agreement between this independent Fab-based imaging approach and our non-canonical amino acid / click-labelling strategy.

We do not present this comparison as a substitute for the requested antibody-versus-click control. Rather, together with the biochemical evidence for LptD* / LptE complex formation and the prior functional complementation data, the consistency with published LptD-Fab imaging supports the use of LptD* as a reporter for LptD-associated OM sites in this study.

In summary, we acknowledge the value of the requested antibody-versus-click controls and made direct attempts to obtain suitable reagents for both LPS and LptD. We were unable to perform these experiments because we could not identify or obtain validated antibodies suitable for the required intact-cell immunofluorescence experiments within the manuscript revision period. We have therefore strengthened the manuscript by clarifying the existing validation supporting both labelling strategies, including Kdo-analogue-specific LPS labelling controls, reciprocal dye controls, prior validation of the Kdo-$N_3$ / click-labelling / dSTORM workflow, biochemical confirmation of LptD* / LptE complex formation, functional validation of LptD*, and consistency with recently published LptD-Fab imaging

**References**

Chng, S.-S., Ruiz, N., Chimalakonda, G., Silhavy, T. J. and Kahne, D. (2010) Characterization of the two-protein complex in *Escherichia coli* responsible for lipopolysaccharide assembly at the outer membrane. *Proc. Natl Acad. Sci. USA* 107: 5363-5368.

Dumont, A., Malleron, A., Awwad, M., Dukan, S. and Vauzeilles, B. (2012) Click-mediated labeling of bacterial membranes through metabolic modification of the lipopolysaccharide inner core. *Angewandte Chemie International Edition* 13: 3143-3146.

Freinkman, E., Chng S.-S., and Kahne, D. (2011) The complex that inserts lipopolysaccharide into the bacterial outer membrane forms a two-protein plug-and-barrel. *Proc Natl Acad Sci USA* 108: 2486-2491.

Kumar, S., Inns, P.G., Ward, S., Lagage, V., Wang, J., Kaminska, R., Booth, M.J., Uphoff, S., Cohen, E.A.K., Mamou, G. and Kleanthous C. (2025) Immobile lipopolysaccharides and outer membrane proteins differentially segregate in growing *Escherichia coli*. *Proc. Natl. Acad. Sci. USA* 122: e2414725122.

Nabarro, J., Leaman, R.M., Lenton, S., Mantion, L., Spears, R.J., Coles, M.C., Pushkin, D.O., Fascione, M.A. and Baumann, C.G. (2026) Lipopolysaccharide confinement in the bacterial outer membrane is governed by interactions within the conserved Lipid A anchor. *EMBO J.* 45: 2338-2369.

Nilsson, I., Grove, K., Dovala, D., Uehara, T., Lapointe, G. and Six, D.A. (2017) Molecular characterization and verification of azido-3, 8-dideoxy-*D*-*manno*-oct-2-ulosonic acid incorporation into bacterial lipopolysaccharide. *J. Biol. Chem.* 292: 19840-19848.

**2. The authors have satisfactorily answered this point. In Fig. S15H they should correct the sequence of the single figures in the panel.**

**RESPONSE:**

We are pleased that our revised manuscript and detailed response has satisfactorily addressed point 2. Thank you to this reviewer for spotting the formatting error in Extended Data Figure S15H. The order of the microscopy images in panel H of this supplementary figure has been corrected in the latest version of the revised manuscript.

**3.-10. The authors have satisfactorily answered these point and altered the ms accordingly.**

**RESPONSE:**

Once again, we appreciate the critical comments provided. We are pleased that our revised manuscript and detailed responses have addressed points 3-10.

**11. The authors claim that the requested experiment, namely the visualization of the decrease of the background LPS is not possible by SIM because of faster photobleaching. However, they could decrease the temporal and/or spatial resolution in the experiment, for example by using a different microscopy technique, spinning disk, confocal, wide field etc. The mere decrease of background LPS should be detectable with any of these techniques and super high resolution is not really necessary for this.**

**RESPONSE:**

We agree with the reviewer that direct live-cell visualisation of decreasing background LPS fluorescence would strengthen this point, and that this does not necessarily require the highest spatial or temporal resolution. We therefore performed additional live-cell time-course experiments specifically designed to extend the imaging window by reducing sample photobleaching.

Rather than switching to a different, lower resolution microscopy modality, we developed a modified reduced-photobleaching 3D-SIM$^2$ acquisition protocol. This allowed us to maintain methodological continuity with the live-cell datasets presented in ***Figure 5*** and ***Extended Data Figure S5***, while extending the imaging window sufficiently to visualise background LPS signal loss over time. Specifically, we reduced the number of SIM phases from 13 to 9 and used reduced 488 nm excitation laser power while retaining sufficient signal-to-noise to visualise outer membrane-associated Alexa Fluor 488-labelled background LPS. Under these reduced-photobleaching acquisition conditions, background LPS fluorescence was monitored in the outer membrane of live, fluorescently labelled *E. coli* cells for up to 120 min.

These new data are now included as ***Extended Data Figure S19*** (panel C is shown below). Across the extended time courses, the labelled background LPS signal remained predominantly outer membrane-associated and retained a punctate, clustered distribution, while the signal from these LPS-rich regions decreased gradually over time. No visible intracellular accumulation, time-dependent polar enrichment, or coalescence of LPS-rich clusters was observed, consistent with 3D-SIM$^2$ images in ***Figure 5***. These observations provide direct live-cell visualisation of background LPS fluorescence loss in individual growing and dividing cells over an extended time window, and are consistent with our fixed-cell dSTORM analyses ***(Figures 2 and 3)*** and biochemical membrane fractionation data (***Figure 6D and Extended Data Figure S6)***. We have revised the Results to describe these new live cell data and added ***Extended Data Figure S19***. We have also updated the Methods with a description of the modified 3D-SIM$^2$ acquisition conditions used for these experiments, including the reduction from 13 to 9 SIM phases and reduced 488 nm excitation laser power. We retain the fixed-cell dSTORM and biochemical fractionation analyses as the quantitative basis for estimating background LPS turnover rates, because long-duration live-cell fluorescence intensity measurements are affected by residual photobleaching, cell growth and division, focus/orientation changes, and cell movement during acquisition.

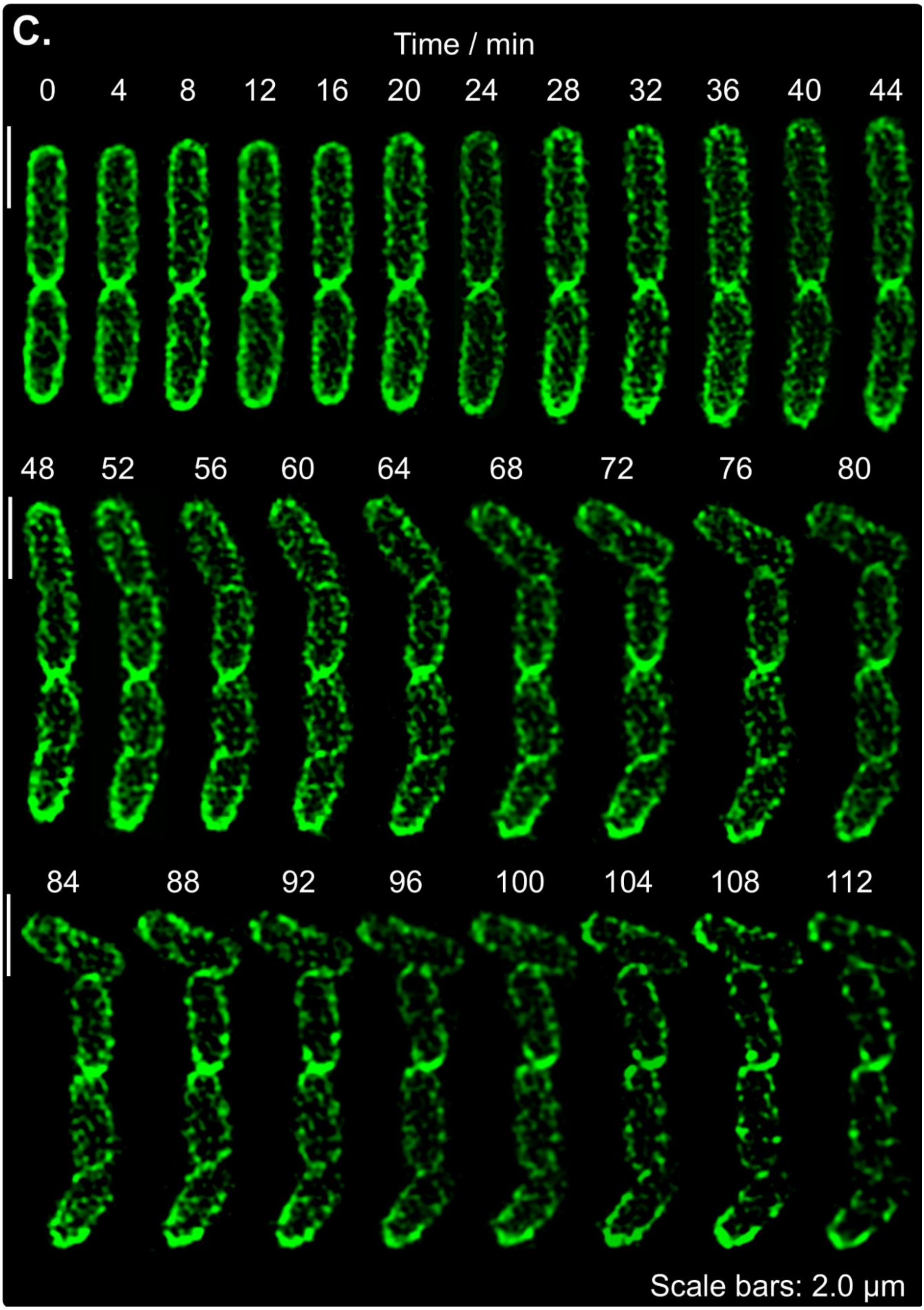


**Extended Data Figure S19C. Extended 3D-SIM[2] time courses visualising the loss of background LPS fluorescence in growing and dividing cells over 112 min.** *E. coli* BW25113 cells containing metabolically labelled background Kdo-$N_3$-LPS were fluorescently labelled *in vivo* with DBCO-AF488 via strain-promoted azide-alkyne cycloaddition (SPAAC), mounted on supplemented M9 CDM / 2% (w/v) agarose pads, and imaged live at 37 °C by 3D-SIM[2]. To extend the live-cell imaging window and reduce AF488 photobleaching, image acquisition settings were modified by reducing both the number of SIM phases from 13 to 9 and the 488 nm laser power. Each frame is labelled with the image acquisition time in minutes**. Scale bars:** 2.0 µm

**Reviewer #3** (Remarks to the Author):

The authors addressed each my concerns. This is a nice study that will be impactful in bacterial cell biology field.

**RESPONSE:**

We appreciate the critical comments provided by this reviewer. We are pleased that our revised manuscript and detailed response have addressed all their points.

**Reviewer #4** (Remarks to the Author):

I am pleased to note that the authors have taken the reviewers' comments, suggestions, and remarks extremely seriously. In my assessment, all the points I raised have been addressed in a thorough and convincing manner. The revisions reflect a thoughtful and rigorous response to the feedback provided.

I also evaluated the responses to Reviewer 1, and all concerns were similarly resolved. While the rebuttal does not explicitly discuss the comment regarding lines 192–205 of the original manuscript, the corresponding text has been amended to address it.

Based on the quality of the revisions, I now recommend that the manuscript be accepted for publication.

**RESPONSE:**

We appreciate the critical comments provided by this reviewer. We are pleased that our revised manuscript and detailed response have addressed all their points. We also very much appreciate this reviewer taking the time to carefully evaluate our direct responses to Reviewer 1, and for confirming that all points raised by Reviewer 1 have been satisfactorily addressed in our revised manuscript.

**Reviewer #5** (Remarks to the Author):

I co-reviewed this manuscript with one of the reviewers who provided the listed reports. This is part of the Nature Communications initiative to facilitate training in peer review and to provide appropriate recognition for Early Career Researchers who co-review manuscripts.

**RESPONSE:**

We appreciate the time taken by this ECR to peer review our manuscript.
